# Development of Stereocontrolled Palladium(II)-Catalyzed Domino Heck/Suzuki β,α-Diarylation Reactions with Chelating Vinyl Ethers and Arylboronic Acids

**DOI:** 10.1002/open.201100010

**Published:** 2012-02

**Authors:** Alejandro Trejos, Luke R Odell, Mats Larhed

**Affiliations:** [a] Division of Organic Pharmaceutical Chemistry, Department of Medicinal Chemistry, Uppsala UniversityBMC, Box 574, 751 23 Uppsala (Sweden)

**Keywords:** chirality, domino reactions, palladium, stereoselective catalysis, water effects

## Abstract

A stereoselective and 1,4-benzoquinone-mediated palladium(II)-catalyzed Heck/Suzuki domino reaction involving metal coordinating cyclic methylamino vinyl ethers and a number of electronically diverse arylboronic acids has been developed and studied. Diastereomeric ratios up to 39:1 and 78 % isolated yields were obtained. The stereoselectivity of the reaction was found to be highly dependent on the nature of the arylboronic acid and the amount of water present in the reaction mixture. Thus, a domino β,α-diarylation–reduction of chelating vinyl ethers can now be accomplished and stereochemically controlled, given that optimized conditions and an appropriate chiral auxiliary are used. To the best of our knowledge, this represents the first example of a stereoselective, oxidative Heck/Suzuki domino reaction in the literature.

## Introduction

C—C bond formations are paramount in organic synthesis and have captured the focus of chemists since the very beginning of modern organic chemistry.^1^ Palladium(0)- and palladium(II)-catalyzed coupling reactions have emerged as efficient and selective methods for the arylation and vinylation of a range of organopalladium precursors.^2^ Among the palladium(0)-catalyzed couplings, the Heck–Mizoroki and Suzuki–Miyaura reactions are two of the most prominent examples.^3^ This was recently recognized by the Royal Swedish Academy of Sciences who awarded Richard F. Heck, Ei-ichi Negishi and Akira Suzuki the 2010 Nobel prize in Chemistry.^4^

Recent advances^5^ in palladium(II)-catalyzed oxidative Heck reactions^6^ have allowed the use of an organometallic reactant as an alternative to aryl halides or pseudo halides. Regeneration of the catalytically active palladium(II) species is facilitated by employing a terminal reoxidant, usually metal salts, dioxygen or 1,4-benzoquinone (*p*-BQ). Two of the most common organometallic substrates used in the oxidative Heck reaction are aryl- or vinylboronic acids. Assuming that β-hydride elimination can be suppressed after the carbopalladation step, a subsequent transmetalation–reductive elimination sequence can occur, which would lead to an additional Suzuki-type arylation. The ability of palladium(II) to facilitate the addition of nucleophiles to alkenes is well described, and a variety of olefin difunctionalization reactions have been developed.^7^ However, domino difunctionalization reactions via a migratory insertion–transmetalation pathway are still relatively unexplored.^8^ The main challenge of the above-mentioned process is to identify structural features or conditions, where the carbopalladation occurs with high regioselectivity^9^ and in which β-hydride elimination can be suppressed, allowing the palladium(II) σ-species to transmetalate with, for example, an arylboronic acid or other substrates, and thereafter undergo reductive elimination.^10^

**Scheme 1:**
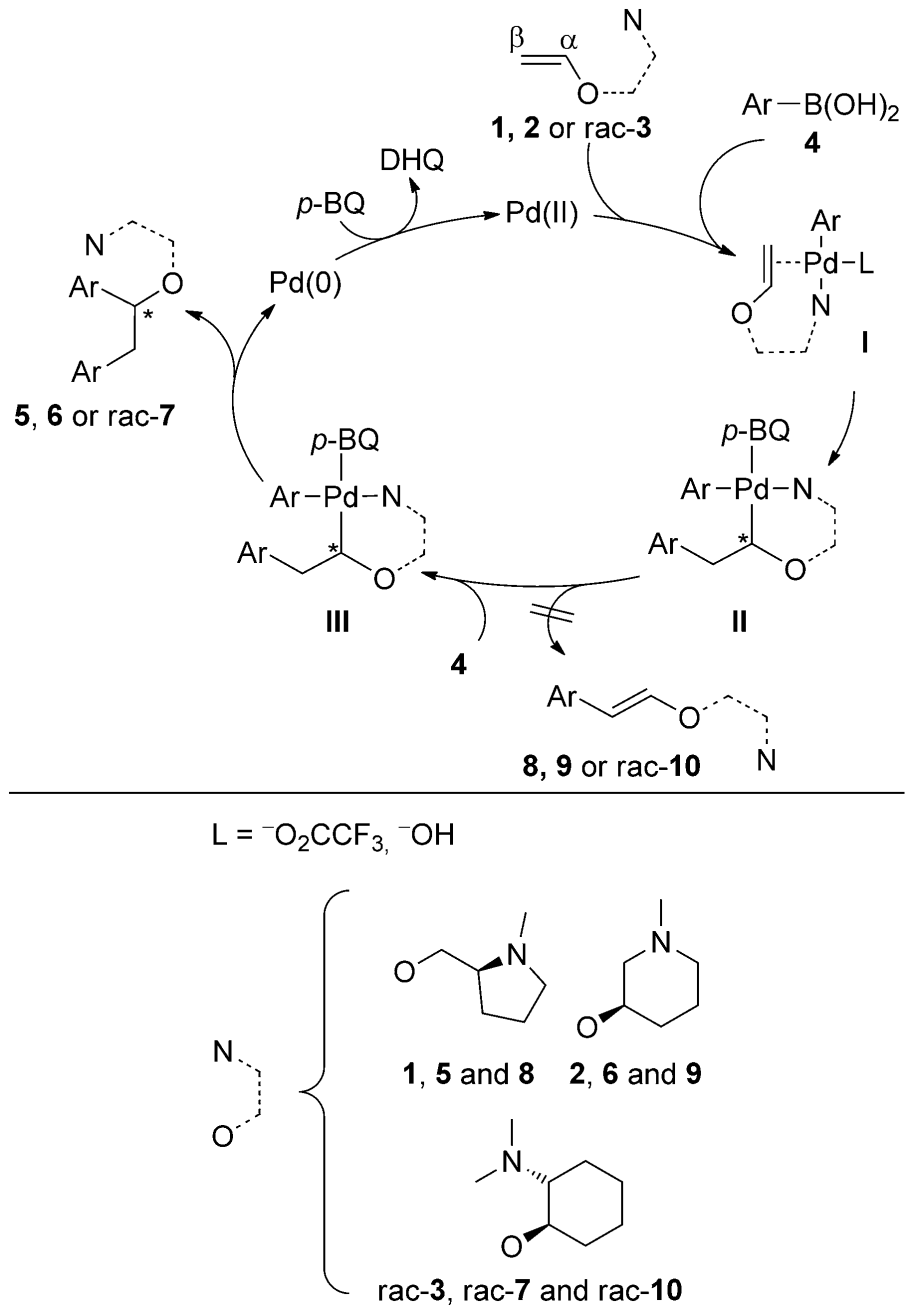
Simplified catalytic cycle for the domino oxidative Heck/Suzuki diarylation employing chiral chelating vinyl ethers.

Previously, our research group reported a novel palladium(II)-catalyzed domino Heck/Suzuki β,α-diarylation of an achiral dimethylaminoethyl-substituted vinyl ether using an excess of arylboronic acid in combination with *p*-BQ.10c For the domino Heck/Suzuki reaction, we proposed a mechanism involving a chelation-controlled carbopalladation step,^11^ which was supported by recent density functional theory (DFT) calculations, highlighting the crucial role of *p*-BQ in the catalytic process.^12^ Since the diarylation reaction requires an olefin equipped with a metal coordinating group, control of the stereochemical outcome of the reaction should be possible by choosing an appropriate chiral catalyst-directing moiety, allowing the generation of diastereomerically enriched σ-intermediate **II** (Scheme 1). Given that we have previously performed stereoselective palladium(0)-catalyzed Heck–Mizoroki reactions using a (*S*)-*N*-methyl-pyrrolidine-based chiral directing group,^13^ we were interested in exploring a similar approach in the Heck/Suzuki domino diarylation using chelating olefins **1**–**3** and arylboronic acids **4** (Scheme 1). Interestingly, the hydrochloride salts of this class of diarylated, amino-substituted ether products (**5**–**7**) have previously been reported to possess antihistamine activity, but the reported synthetic route requires long reaction times and stochiometric amounts of sodium metal.^14^

## Results and Discussion

**Scheme 2:**
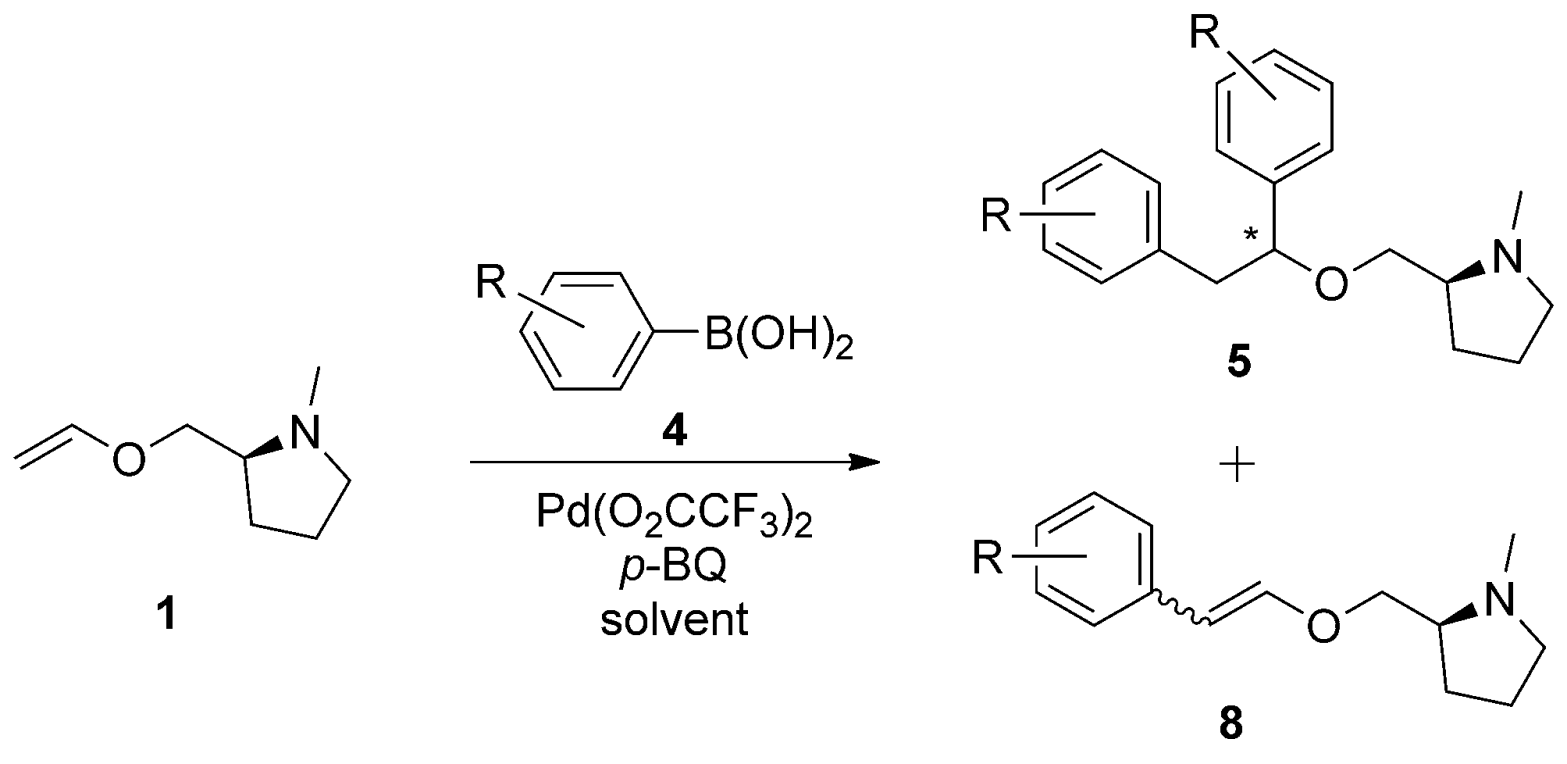
Reaction of 1 with different arylboronic acids 4 yielding domino product 5 and/or oxidative Heck product 8.

Based on our previous experience, the stereoselective domino reaction was first attempted with functionalized vinyl ether **1** (Scheme 2). In an initial test, one equivalent of olefin **1** was added to a vial containing three equivalents of boronic acid **4 a**, a slight excess of *p*-BQ (1.1 equiv) and catalytic amounts of Pd(O_2_CCF_3_)_2_ (0.05 equiv) in 1,4-dioxane (1.5 mL) at 40 °C (entry 1, Table 1). Analysis of the crude material by GC–MS and ^1^H NMR showed that the β,α-diarylated product **5 a** was successfully formed in the reaction with a diastereomeric ratio (*d.r*.) of 3.7:1 and a 5:1 ratio of compound **5** to **8**. In an attempt to improve the selectivity of the reaction, different solvents were screened. Disappointingly, polar solvents such as acetonitrile, *N*,*N*-dimethylformamide (DMF) or dimethyl sulfoxide (DMSO) increased the amount of monoarylated Heck product **8 a** (entries 2–4, Table 1). Toluene was also tested as the reaction medium, but the rate decreased dramatically and a significant amount of starting material **1** remained unreacted, even after 36 h at 40 °C. Increasing the equivalents of arylboronic acid seemed to decrease the required reaction time (c.f. entries 1, 13, 14, and 25–30, Table 1). When Pd(OAc)_2_ was used, the *d.r*. increased slightly. However, this was accompanied by a lower ratio of compound **5** to **8** and lower yields.

**Table 1:** Initial investigation of the Heck/Suzuki diarylation of vinyl ether 1 with 4-methoxyphenylboronic acid (4 a) and 4-acetylphenylboronic acid (4 b).^[a]^

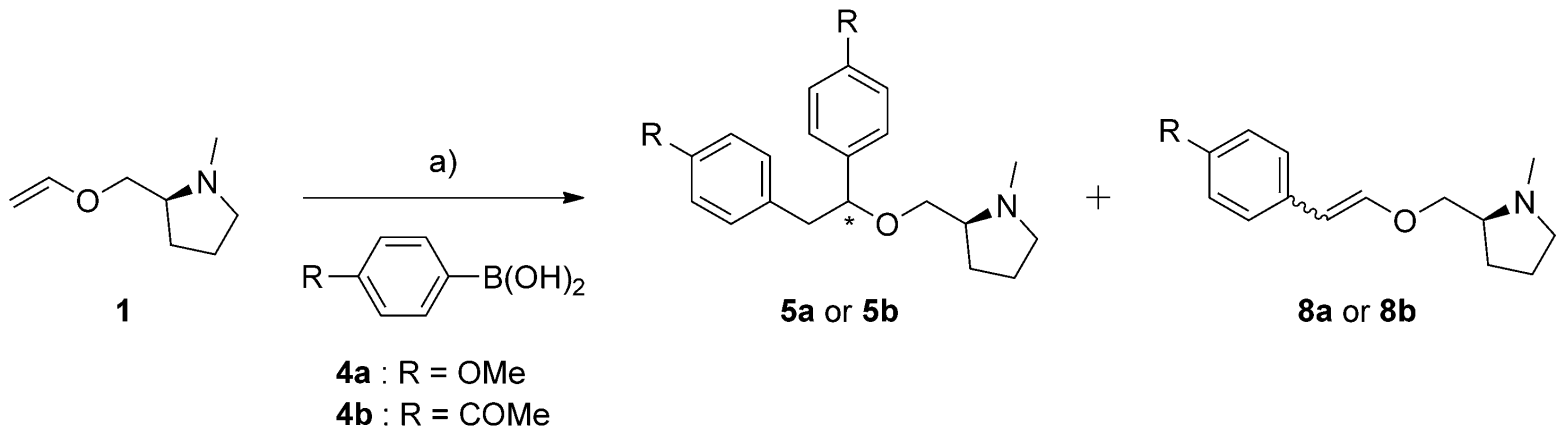										
Entry^[b]^	Temp [°C]	Pd(O_2_CCF_3_)_2_ [equiv]	Boronic acid	Time [h]	**4 a** or **b** [equiv]	Solvent [mL]	Ratio **5**:**8**^[c]^	*d.r*.^[d]^	Yield [%]^[d]^	Product
1	40	0.05	**4 a**	24	3	1,4-dioxane (1.5)	5:1	3.7:1	68	**5 a**
2	40	0.05	**4 a**	24	3	acetonitrile (1.5)	1.8:1	7.0:1	15	**5 a**
3	40	0.05	**4 a**	10	3	DMF (1.5)	1:1.2	4.2:1	26	**5 a**
4	40	0.05	**4 a**	8	3	DMSO (1.5)	1:1.5	9.0:1	12	**5 a**
5	40	0.05	**4 a**	36	3	toluene (1.5)	–^[e]^	8.2:1	28	**5 a**
6	40	0.05^[f]^	**4 a**	24	3	1,4-dioxane (1.5)	1.8:1	6.0:1	–^[g]^	**5 a**
7	25	0.025	**4 a**	36	3	1,4-dioxane (0.7)	9.4:1	7.3:1	51	**5 a**
8			**4 b**	36			14.4:1	4.7:1	53	**5 b**
9	40	0.025	**4 a**	24	4	1,4-dioxane (1.5)	5:1	39:1	38	**5 a**
10			**4 b**	24			100:1	3.5:1	76	**5 b**
11	60	0.025	**4 a**	8	5	1,4-dioxane (3.0)	19:1	19:1	28	**5 a**
12			**4 b**	6			43:1	3.7:1	88	**5 b**
13	40	0.05	**4 a**	o.n.^[h]^	5	1,4-dioxane (0.7)	5.1:1	27:1	57	**5 a**
14			**4 b**	10			35:1	2.6:1	88	**5 b**
15	60	0.05	**4 a**	7	3	1,4-dioxane (1.5)	6.9:1	4.0:1	50	**5 a**
16			**4 b**	5			100:1	1.3:1	41	**5 b**
17	25	0.05	**4 a**	36	4	1,4-dioxane (3.0)	9.1:1	19:1	36	**5 a**
18			**4 b**	24			100:1	1.9:1	77	**5 b**
19	60	0.1	**4 a**	5	4	1,4-dioxane (0.7)	26:1	2.2:1	43	**5 a**
20			**4 b**	3			100:1	1.6:1	29	**5 b**
21	25	0.1	**4 a**	o.n.^[h]^	5	1,4-dioxane (1.5)	14:1	6.1:1	58	**5 a**
22			**4 b**	8			13:1	3.2:1	69	**5 b**
23	40	0.1	**4 a**	o.n.^[h]^	3	1,4-dioxane (3.0)	1.9:1	1.9:1	32	**5 a**
24			**4 b**	o.n.^[h]^			21:1	1.8:1	53	**5 b**
25	40	0.05	**4 a**	20	4	1,4-dioxane (1.5)	11.5:1	13.3:1	67	**5 a**
26			**4 b**	20			35:1	2.6:1	83	**5 b**
27	40	0.05	**4 a**	20	4	1,4-dioxane (1.5)	12.3:1	19:1	59	**5 a**
28			**4 b**	20			45:1	2.5:1	67	**5 b**
29	40	0.05	**4 a**	20	4	1,4-dioxane (1.5)	12.7:1	8.9:1	62	**5 a**
30			**4 b**	20			18:1	3.7:1	82	**5 b**

[a] *Reagents and conditions*: a) Olefin **1** (0.21 mmol, 1 equiv), **4**, *p*-BQ (1.1 equiv), Pd(O_2_CCF_3_)_2_, solvent, Δ.

[b] Entries 25–30 are triplicates in the D-optimal reaction design.

[c] Determined by GC–MS analysis of the crude product.

[d] Determined by ^1^H NMR analysis of the crude product.

[e] Monoarylated product was not detected.

[f] Pd(OAc)_2_ was used.

[g] No data.

[h] Overnight (o.n.) reaction time (12–16 h).

Entries 7–30 in Table 1 represent a determinant (D)-optimal design set^15^ in which 4-methoxyphenylboronic acid (**4 a**) and 4-acetylphenylboronic acid (**4 b**) were used in order to assess differences in the reaction outcome due to electronic effects. Additionally, the following factors were evaluated in the screening process: solvent volume, arylboronic acid equivalents, palladium equivalents and temperature. Unfortunately, no statistically significant model was obtained for the ratio of compound **5** to **8**, however, two significant models were obtained for the stereoselectivity shown by both **4 a** (*R*^2^=0.81, *Q*^2^=0.46) and **4 b** (*R*^2^=0.84 and *Q*^2^=0.62), as well as for the yield of **5 b** (*R*^2^=0.78 and Q^2^=0.51). According to these findings, lower amounts of Pd(O_2_CCF_3_)_2_ and increased amounts of arylboronic acid **4** would benefit the stereochemical outcome of the reaction with respect to **5**. Lower temperatures showed a tendency to increase stereoselectivity, but the results were not statistically significant. The amount of solvent studied in these experiments proved to be insignificant for both the stereoselectivity and yield of **5**. Interestingly, the same factors controlling the stereoselectivity also appeared to influence the yield of **5 b**.^16^

The results shown in Table 1 also indicate that the stereoselectivity is strongly governed by electronic effects (e.g., entries 17 and 18). Thus, phenylboronic acid **4 c** was tested using conditions that, according to the screening design, should furnish high yields and good stereoselectivity, meaning low catalyst loading and an excess of phenylboronic acid (entry 1, Table 2). Surprisingly, the stereoselectivity of this reaction was poor, and the yield was moderate. Hence, we opted to test other conditions in which only the temperature was increased (entry 2, Table 2) or the temperature was increased and the amount of catalyst was decreased (entry 3, Table 2) and finally one example in which the temperature and the amount of arylboronic acid were increased (entry 4, Table 2). Unfortunately, none of these experiments yielded satisfactory stereoselectivities and yields.

**Table 2:** Heck/Suzuki domino diarylation of 4 c with 1 employing conditions based on the results obtained from Table 1.^[a]^

Entry	Temp [°C]	Pd(O_2_CCF_3_)_2_ [equiv]	**4 c** [equiv]	Time [h]	Ratio **5 c**:**8 c**	*d.r*.^[b]^	Yield [%^[b]^	Product
1	25	0.02	4	36	>100:1	1.4:1	52	**5 c**
2	40	0.02	4	24	>100:1	1.9:1	42	**5 c**
3^[c]^	40	0.01	4	72	>100:1	1.6:1	24	**5 c**
4	50	0.02	6	24	>100:1	1.4:1	43	**5 c**

[a] *Reagents and conditions*: Olefin **1** (0.21 mmol, 1 equiv), **4 c**, *p*-BQ (1.5 equiv), Pd(O_2_CCF_3_)_2_, 1,4-dioxane (1.5 mL).

[b] Determined by GC–MS and NMR analysis of the crude product.

[c] Reaction did not go to completion.

To ensure that the results obtained with **4 c** were not anomalous, the conditions depicted by entry 2 of Table 2 were tested with an array of different arylboronic acids. Nearly half of the tested arylboronic acids (Table S3, Supporting Information)^17^ produced only trace amounts of product **5**, and the remaining arylating substrates yielded diarylated products with low stereoselectivities. These results suggest that factors other than catalyst loading and the amount of boronic acid have a profound effect on the stereochemical outcome of the reaction.

### Water concentration studies

**Figure 1:**
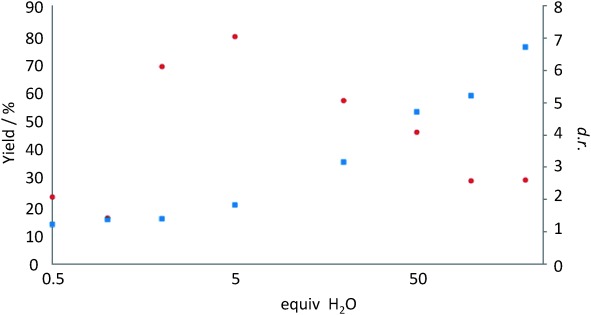
Influence of water content on the diastereoselectivity (*d.r*.: •) and yield (determined by NMR: ▪) of the chiral diphenylation of olefin 1 (see the Experimental Section for full details).

As shown in Table 1, diastereomeric ratios up to 39:1 could be achieved using **4 a** in low to moderate yields (entries 9, 11 and 17, Table 1). However, these results could not be reproduced using other arylboronic acids. The lack of reproducibility in reactions involving boronic acids has been linked to variations in water content,^18^ which is partially dependent on the solvent used for recrystallization.^19^ In the same manner, different batches of solvent used in the screening processes presumably contained varying water concentrations, which could have affected the reaction outcome. Previous work by our group has also illustrated the importance of water in an achiral version of the chelation-controlled Heck/Suzuki domino reaction.^12^ We therefore decided to investigate the effect of water content on the outcome of this reaction. The reactions were conducted under inert conditions using **1**, water-free 1,4-dioxane, recrystallized *p*-BQ (from absolute ethanol) and phenylboronic acid **4 c** (recrystallized from acetone or diethyl ether). The water content was then varied from 0.5 to 200 equivalents, keeping the total reaction volume constant at 1.5 mL. As depicted in Figure 1, an increased amount of water improved the diastereoselectivity up to a *d.r*. of 6.7:1 but reduced the product yield down to below 30 %. However, accompanied with improved diastereoselectivity, an increased water content also resulted in a decreased conversion of **1** and an increased formation of β-monophenylated Heck product **8 c**, which might account for the reduced yield of **5 c**.

**Scheme 3:**
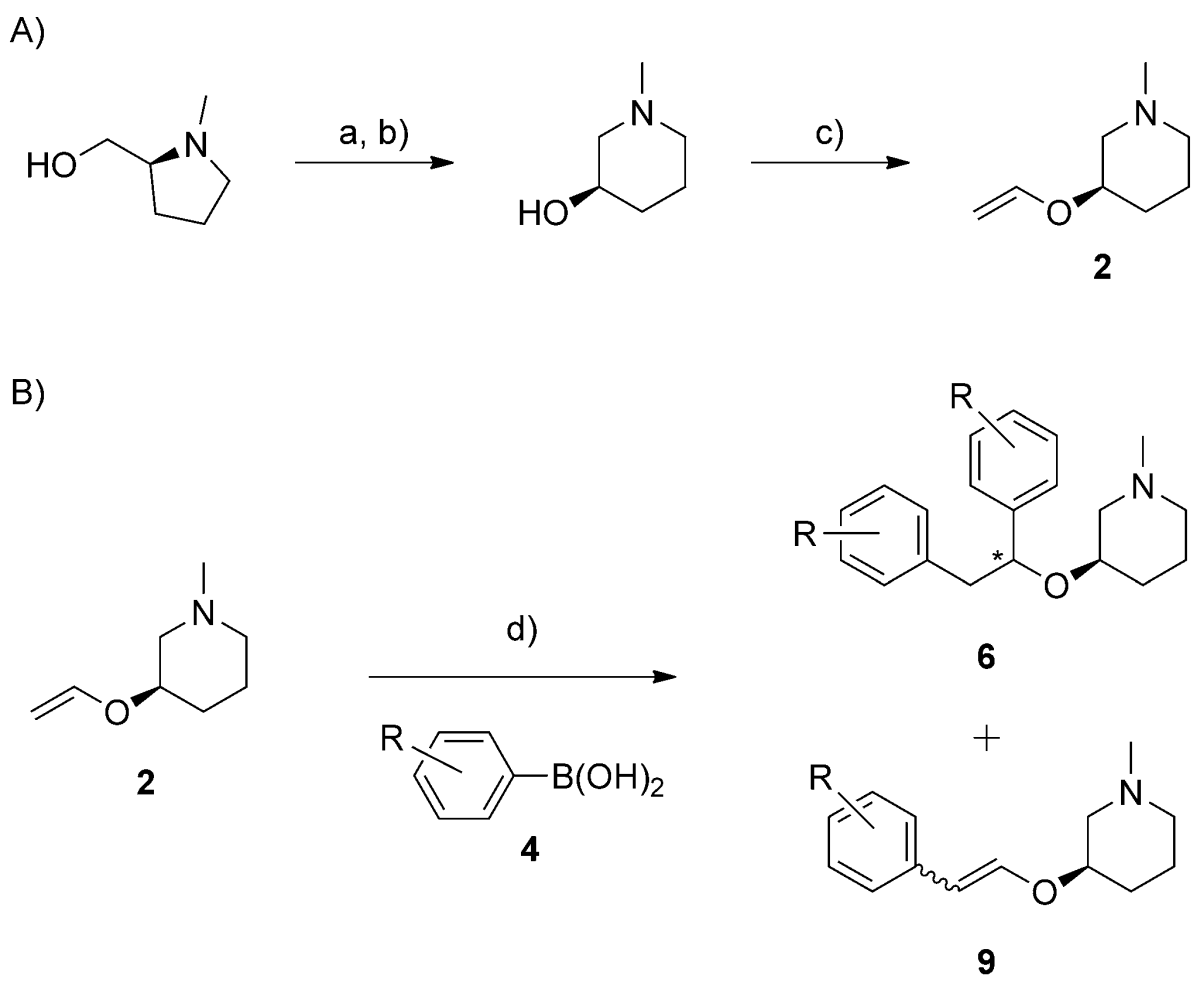
A) Synthesis of (*R*)-1-methyl-3-(vinyloxy)piperidine 2 from (*S*)-(1-methylpyrrolidin-2-yl)methanol (>49:1 *e.r*.). B) Reaction of 2 with different arylboronic acids 4 yielding domino product 6 and/or oxidative Heck product 9. *Reagents and conditions:* a) Trifluoroacetic anhydride, Et_3_N, dry THF, 0–20 °C; b) Aq NaOH (5 M), 55 %, >49:1 *e.r*.; c) Ethyl vinyl ether, 2,2′-bipyridine, Pd(O_2_CCF_3_)_2_, O_2_ (1 atm), 60 °C, 37 %, >49:1 *e.r*.; d) Pd(O_2_CCF_3_)_2_, *p*-BQ, H_2_O, 1,4-dioxane.

**Figure 2:**
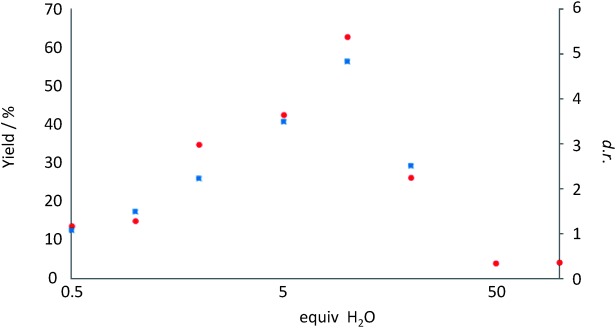
Influence of water content on the diastereoselectivity (*d.r*.: •) and yield (determined by NMR: ▪) of the chiral diphenylation of olefin 2 (see the Experimental Section for full details).

As we were unable to identify reaction conditions that simultaneously afforded high yields and stereoselectivities, we decided to examine the effect of a different catalyst-presenting group on the stereoselectivity. Enantiomer-enriched vinyl ether **2** (enantiomeric ratio (*e.r*.) ≥49:1) was synthesized via a stereoselective ring enlargement of (*S*)-(1-methylpyrrolidin-2-yl)methanol^20^ followed by palladium(II)-catalyzed vinylation of (*R*)-1-methylpiperidin-3-ol (Scheme 3 A). Compound **2** was then submitted to a series of test reactions using arylboronic acids **4 a**–**k** (Scheme 3 B). The results from these experiments showed that diastereomerically enriched products could be obtained, but, as was the case when **1** was used, the results varied significantly. Thus, the impact of the water concentration in the reaction between **2** and **4 c** was also explored. This system appeared to be even more sensitive to the amount of water, although the yields and stereoselectivities were comparable to the results obtained for compound **1** when approximately ten equivalents of water were used (Figure 2, *d.r*.: 5:1, 63 % yield). With greater amounts of water (50 and 100 equiv), the reaction yield dropped dramatically preventing the determination of *d.r*.

**Scheme 4:**
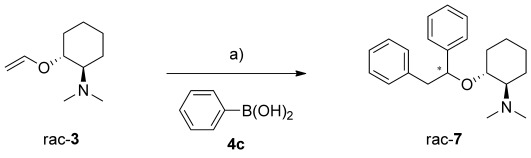
Reaction between racemic *trans-N*,*N*-dimethyl-2-(vinyloxy)cyclohexanamine (rac-3) and phenylboronic acid 4 c. *Reagents and conditions:* a) vinyl ether rac-3 (1 equiv), 4 (4 equiv), *p*-BQ (1.5 equiv), Pd(O_2_CCF_3_)_2_ (0.04 equiv), H_2_O (10 equiv), 1,4-dioxane (1.5 mL), 40 °C, 40 %, 2:1 *d.r*. (see the Experimental Section for further details).

Further attempts to find a more productive chiral auxiliary were undertaken, and racemic *trans-N,N*-dimethyl-2-(vinyloxy)cyclohexanamine (rac-**3**) was synthesized^21^ and tested with phenylboronic acid **4 c** employing the same conditions as in Table 3. Disappointingly, rac-**7** was obtained in only 40 % yield and with a *d.r.* of only 2:1 (Scheme 4). Further attempts to react rac-**3** with other arylboronic acids were discontinued at this point.

**Table 3:** Domino oxidative Heck/Suzuki reaction using vinyl ethers 1 and 2.^[a]^

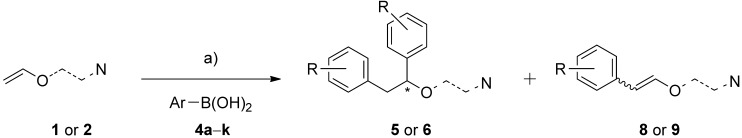								
Entry	Olefin	Ar—B(OH)_2_		Time [h]	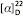 [°]^[b]^	*d.r*.^[c]^	Yield [%]^[d]^	Product
1	**1**	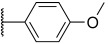	**4 a**	24	−22.6	2.9:1	52	**5 a**
2	**2**			36	4.5	2.2:1	12	**6 a**
3	**1**	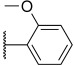	**4 d**	24	−16.6	1.4:1	24	**5 d**
4			**2**	–	–	–	–	**6 d**
5	**1**	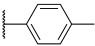	**4 e**	24	−29.1	2.3:1	56	**5 e**
6			**2**	36	7.1	3.0:1	63	**6 e**
7	**1**	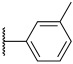	**4 f**	24	−33.9	2.0:1	63	**5 f**
8			**2**	36	−2.5	3.4:1	52	**6 f**
9	**1**	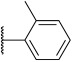	**4 g**	24	–	–	trace^[e]^	**5 g**
10			**2**	36	8.7	1.7:1	49	**6 g**
11	**1**	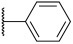	**4 c**	24	−38.3	2.5:1	78	**5 c**
12			**2**	36	−2.7	4.7:1	63	**6 c**
13	**1**	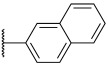	**4 h**	24	8.4	2.6:1	47	**5 h**
14			**2**	36	15.6	4.7:1	30	**6 h**
15	**1**	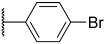	**4 i**	36	−7.3	3.4:1	52	**5 i**
16			**2**	36	15.8	3.0:1	35	**6 i**
17	**1**	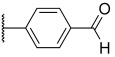	**4 j**	24	−13.5	3.0:1	64	**5 j**
18			**2**	–	–	–	–	**6 j**
19	**1**	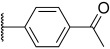	**4 b**	24	−11	4.0:1	62	**5 b**
20			**2**	–	–	1.8:1	<10^[f]^	**6 b**
21	**1**	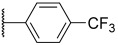	**4 k**	24	−29.0	4.7:1	41	**5 k**
22			**2**	36	−13.7	4.7:1	48	**6 k**

[a] *Reagents and conditions*: a) vinyl ether **1** or **2** (30 mg, 0.21 mmol), **4** (4 equiv), *p*-BQ (1.5 equiv), Pd(O_2_CCF_3_)_2_ (0.04 equiv), H_2_O (10 equiv), 1,4-dioxane (1.5 mL), 40 °C.

[b] Optical rotation (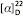) was performed in chloroform for the diastereochemically enriched product.

[c] Calculated by GC–MS and/or ^1^H NMR of the crude product.

[d] Isolated yields unless stated otherwise. Product purity >95 % according to GC–MS and ^1^H NMR.

[e] Product formation detected with GC–MS.

[f] Calculated from NMR analysis with DMF as an internal standard; not isolated.

### Scope and limitations

As can be seen from Figures 1 and 2, the amount of water required to obtain reasonable stereoselectivity and still retain useful yields is in the range of 10–20 equivalents for both olefins **1** and **2**. Accordingly, we decided to explore the scope and limitations of this reaction, using chelating olefins **1** and **2** and ten equivalents of water. The preparative results for the domino diarylation–reduction of **1** and **2** with different arylboronic acids **4** are presented in Table 3. Somewhat higher diastereoselectivity could be obtained using vinyl ether **2**, but the yields were generally lower and longer reaction times were required. Further, in a number of cases, **2** failed to give any detectable amount of product (c.f., entries 3, 4 and 17, 18, respectively, Table 3). The reaction between **1** and boronic acid **4 a** or **4 b** produced **5 a** or **5 b**, respectively, in moderate yields and stereoselectivities, whereas the corresponding reactions with **2** afforded **6 a** and **6 b** in very low yields and stereoselectivities (entries 1, 19, 2 and 20, respectively, Table 3). In addition, ^1^H NMR analysis of the crude showed a low diarylated (**6 b**) to monoarylated (**9 b**) product ratio of 1:1.4. Interestingly, sterically demanding *ortho*-substituted **4 d** produced **5 d** in low yield and stereoselectivity after reaction with **1**, but no product was detected when **2** was used as the starting material (entries 3 and 4, Table 3). In contrast, **4 g** did not give productive results when reacted with **1**, however, product **6 g** was obtained in reasonable yield and low stereoselectivity when **2** was used (entries 9 and 10, Table 3). In addition, the reactions of 1-naphthylboronic acid with **1** and **2** failed to produce any trace of product (data not shown), suggesting that the reaction is sensitive to steric effects. 4-Bromophenylboronic acid **4 i** provided useful results with both olefin **1** and **2**, yielding valuable diarylated products with further synthetic potential (entry 15 and 16, Table 3).

Additional experiments were performed to gain information on selectivity between arylboronic acids with different electronic properties and whether the addition of a chiral ligand could influence the stereoselectivity of the reaction. First, tolylboronic acid **4 e** and formylboronic acid **4 j** were added to a reaction vial in equimolar amounts (4 equiv) and reacted using the reaction conditions described in Table 3. The resulting crude mixture contained a mixture of four diarylated domino products in a 2:2:2:1 ratio.^22^ Secondly, the chiral ligand (*R*)-(+)-2,2′-bis(diphenylphosphino)-1,1′-binaphthyl (BINAP) was tested in the dominio reaction with **1** and **4 e**, since previous reports have shown that this ligand is capable of inducing chirality in oxidative Heck reaction products.^23^ The reaction was performed using the same reaction conditions as specified in Table 3, but with the addition of (*R*)-BINAP prior to the addition of Pd(OOCF_3_)_2_ under a nitrogen atmosphere. GC–MS and NMR analysis of the crude product showed that the reaction yielded primarily the α-monoarylated Heck product **8 e** and very small amounts of desired domino product **5 e** (ratio: 8:1).^24^ The chiral ligand (*R*)-BINAP can in this case act as a bidentate ligand, hindering coordination from the nitrogen and *p*-BQ, which completely impedes chelation and a second arylation from occurring.

The results obtained from these studies showed that useful stereoselectivity could be achieved in this palladium(II)-catalyzed Heck/Suzuki domino reaction, but that the outcome was highly sensitive to the amount of water present in the system, regardless of the choice of chelating olefin. This phenomenon might be a result of the polarity of the reaction media. Therefore, other solvents systems (acetonitrile/water; 1,4-dioxane/ionic liquids) were evaluated, but the 1,4-dioxane/water system consistently yielded the best results. Comparable diastereoselectivities were only obtained using acetonitrile/water mixtures, although the yields were substantially lower when this solvent combination was used, mainly due to an increased formation of the monoarylated oxidative Heck product.

### X-ray diffraction analysis

**Figure 3:**
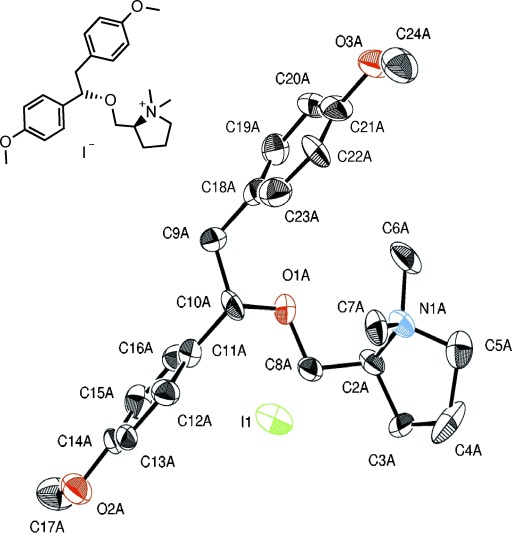
Crystals of (*S*,*S*)-11 were obtained by vapor diffusion crystallization. Thermal ellipsoids are drawn at the 50 % probability level and displays the preferred conformation (*S*,*S*)-11.

To determine the absolute configuration of the Heck/Suzuki domino product, the reaction of **1** and **4 a** yielding **5 a** in high selectivity (*d.r*. >19) was chosen for crystallization studies. After isolation of **5 a**, methyl iodide (0.5 mL) was used to methylate the amino group, providing the positively charged quaternary salt **11**. The salt was recrystallized by the vapor diffusion crystallization method using diethyl ether/isobutanol:ethyl acetate (10:1). Analysis by X-ray crystallography showed that the auxiliary-directed attachment of the internal anisyl group provided the (*S*)-configuration of the tertiary benzylic carbon in **11** (Figure 3). Starting from chiral vinyl ether **1**, this stereochemical outcome suggests a *Re*-face carbopalladation of complex **I** and subsequent formation of intermediate palladacycles **II** and **III** with high *d.r*. prior to reductive elimination to yield the dominant product (**5**) (Scheme 1).

## Conclusions

An investigation of the scope and limitations of a novel palladium(II)-catalyzed Heck/Suzuki β,α-diarylation of chiral catalyst-presenting vinyl ethers **1**–**3** has been described, providing 1,2-diaryl ethers **5**–**7** in diastereomeric ratios of up to 39:1 and 78 % isolated yield. Promising results show for the first time that a domino β,α-diarylation–reduction of chelating vinyl ethers can be stereochemically controlled, given the right conditions and an appropriate chiral auxiliary. In addition, the pivotal role of water in effecting the stereoselectivities has been investigated. We are currently exploring the use of computational calculations to design more efficient chiral auxiliaries.

## Experimental Section

**General**: Column chromatography was performed on silica gel 60 (40–63 μm, Merck). Analytical thin layer chromatography (TLC) was performed using aluminum sheets precoated with silica gel 60 F_254_. Chromatographic spots were visualized using UV detection and/or ethanolic ninhydrin solution (2 %), or ethanolic phosphomolybdic acid (5 %) followed by heating. Dry column vacuum chromatography (DCVC) was performed using silica gel 60 (40–63 μm, Merck). Preparative thin layer chromatography (PTLC) was performed using PLC silica gel 60 F_254_ (2 mm, Merck). Analytical GC–MS was performed on a Varian Saturn 3900/2100 system (Palo Alto, CA, USA) using a CP-SIL 5 or CP-SIL 8 CB low bleed capillary column (30 m × 0.25 mm) and electron impact (EI) ionization (70–300 °C, 20 °C min^−1^ or at 180–185 °C, 0.1 °C min^−1^). Analytical reverse-phase (RP)-HPLC–MS was performed on a Gilson HPLC system (Middleton, WI, USA) with a Finnigan AQA electrospray ionization (ESI) quadrupole mass spectrometer and using an Onyx Monolithic C18 4.6×50 mm column (Phenomenex, Torrance, CA, USA) with CH_3_CN/H_2_O in 0.05 % aq HCOOH as mobile phase at a flow rate of 4 mL min^−1^. Optical rotation was measured using a Perkin–Elmer 241 polarimeter (Na_λ_, 589 nm, Waltham, MA, USA). ^1^H and ^13^C NMR spectra were recorded on Varian Mercury Plus instruments (Palo Alto, CA, USA). Exact molecular masses were determined on a Micromass Q-Tof2 mass spectrometer equipped with an electrospray ion source. Collection of X-ray data for compound (*S*,*S*)-**11** was performed at the Latvian Institute of Organic Synthesis (Riga, Latvia). CCDC-851225 contain(s) the supplementary crystallographic data for this paper. These data can be obtained free of charge from The Cambridge Crystallographic Data Centre via http://www.ccdc.cam.ac.uk/data_request/cif. Screening design was planned with assistance of MODDE, version 9.0.0.0 (September 30, 2009), licensed by Umetrics AB.

**General procedure for the synthesis of diarylated products 5, 6 and rac-7**: Compound **4** (4 equiv), 1,4-benzoquinone (1.5 equiv), the appropriate vinyl ether (0.21 mmol **1** or **2**, or 0.18 mmol rac-**3**) and anhyd 1,4-dioxane (1.5 mL) were mixed in an 8 mL reaction vial. The mixture was homogenized by vigorous stirring, after which Pd(O_2_CCF_3_)_2_ (0.04 equiv) and H_2_O (10 equiv) were added. The vial was capped and heated in a metal heating block at 40 °C for 24 or 36 h. The crude material was filtered through a short Al_2_O_3_ column (2 cm Φ, 3 cm height) and eluted with 200 mL EtOAc/Et_3_N (10:1) or until no more product was detected (typically, 100 mL eluent was sufficient). The solvent was evaporated in vacuo, and the crude was analyzed by GC–MS and/or NMR spectroscopy to elucidate the diastereoselectivity. The product was thereafter purified by gradient elution using DCVC^25^ and isohexane/Et_2_O/Et_3_N as the eluent system (a maximum of 4 % Et_3_N was used).

**(*S*)-1-methyl-2-((vinyloxy)methyl)pyrrolidine (1)**: 1-Methylpiperidin-3-ol (5.00 g, 43.4 mmol) dissolved in ethyl vinyl ether (65 mL), 2,2′-bipyridyl (406 mg, 2.6 mmol) and Pd(OAc)2 (433 mg, 2.0 mmol) were added to a 250 mL three-necked flask and refluxed at 75 °C. Due to the volatile nature of the starting material, additional ethyl vinyl ether was added several times during the reaction to avoid complete evaporation as the reaction flask was kept open to drive the equilibrium towards product formation by evaporation of EtOH, which is formed as a by-product. Please note that when monitoring was not possible, the collection funnel was closed so that no starting material could evaporate. The mixture was monitored by GC–MS. When complete consumption of the starting material was detected (usually after reaction for 4–5 d), the temperature was decreased to RT, the ethyl vinyl ether was evaporated, and the crude was diluted with EtOAc (100 mL) and washed with 2 M aq NaOH (2×100 mL). The addition of brine was sometimes needed to assist phase separation. The organic phase was dried (K_2_CO_3_), filtered and concentrated in vacuo, and the crude was purified by bulb-to-bulb distillation (65 °C, 50 mbar) or silica gel chromatography (isohexane/Et_2_O/Et_3_N, 90:6:4) to give compound **1** as a colorless oil (2.80 g, 19.8 mmol, 46 %,): *R*_f_=0.2 (isohexane/EtOAc/Et_3_N, 90:6:4); 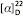
=−46.7° (*c*=8.5 in MeOH); ^1^H NMR (400 MHz, CDCl_3_, 20 °C, TMS): *δ*=6.05 (dd, ^3^*J*_H–H_=6.8, 14.4 Hz, 1 H, CH_2_C*H*), 4.18 (dd, ^2^*J*_H–H_=2.0 Hz, ^3^*J*_H–H_=14.4 Hz, 1 H, (*E*)-C*H*_2_CH), 3.98 (dd, ^2^*J*_H–H_=2.0 Hz, ^3^*J*_H–H_=6.8 Hz, 1 H, (*Z*)-C*H*_2_CH), 3.70 (dd, ^3^*J*_H–H_=5.1 Hz, ^2^*J*_H–H_=9.8 Hz, 1 H, OC*H*_2_CH), 3.16 (dd, *J*_H–H_=5.4 Hz, ^2^*J*_H–H_=9.8 Hz, 1 H, OC*H*_2_CH), 3.08 (m, 1 H, CH_2_C*H*CH_2_), 2.52–2.44 (m, 1 H, NC*H*_2_), 2.40 (s, 3 H, NC*H*_3_), 2.23 (m, 1 H, NC*H*_2_), 1.99–1.89 (m, 1 H, CHC*H*_2_), 1.83–1.61 ppm (m, 3 H, CHC*H*_2_ and CH_2_C*H*_2_); ^13^C NMR (100 MHz, CDCl_3_, 20 °C, TMS): δ=151.9 (CH_2_*C*H), 86.2 (*C*H_2_CH), 70.2 (O*C*H_2_), 64.2 (N*C*H), 57.7 (N*C*H_2_), 41.4 (N*C*H_3_), 28.5 (CH*C*H_2_), 22.8 ppm (CH_2_*C*H_2_); MS (EI, 70 eV): *m*/*z* (%): 142 (34) [*M*]^+^, 98 (35) [C_6_H_12_N]^+^, 84 (100) [C_5_H_10_N]^+^; HRMS (ESI): *m*/*z* [*M*+H]^+^ calcd for C_8_H_15_NO: 142.1232, found: 142.1237.

**(*R*)-1-methyl-3-(vinyloxy)piperidine (2)**: An oven-dried round bottom Schlenk flask containing ethyl vinyl ether (118 mL, 1.24 mol) was purged with O_2(*g*)_ and thereafter Pd(O_2_CCF_3_)_2_ (451.3 mg, 1.97 mmol) and 2,2′-bipyridine (310.0 mg, 1.97 mmol) were added under vigorous stirring. The mixture was stirred at 60 °C until all solids were dissolved and a clear bright yellow solution was obtained. To prevent acetal formation,^26^ Et_3_N (0.69 mL, 4.97 mmol) was added before the addition of piperidine alcohol (2.90 g, 24.8 mmol) by cannula. The reaction was refluxed for 72 h or until GC–MS showed no further improvement. Excess ethyl vinyl ether was removed in vacuo, and the crude material (reddish oil) was washed with 2 M aq NaOH (3×150 mL) and EtOAc (150 mL). The organic phase was evaporated in vacuo, and bulb-to-bulb distillation recovered unwashed/unreacted aminoalcohol (90 °C, 50 mbar) and gave purified compound **2** (65 °C, 58 mbar) as a colorless oil (1.31 g, 9.2 mmol, 37 % yield,): *R*_f_=0.2 (isohexane/EtOAc/Et_3_N, 90:6:4); 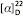
=+18.7° (*c*=7.9 in MeOH); ^1^H NMR (400 MHz, CD_3_OD, 20 °C, TMS): *δ*=6.31 (dd, ^3^*J*_H–H_*=*6.6, 14.1 Hz, 1 H, CH_2_C*H*), 4.29 (dd, ^2^*J*_H–H_*=*1.6 Hz, ^3^*J*_H–H_*=*14.1 Hz, 1 H, (*E*)-C*H*_2_CH), 3.98 (dd, ^2^*J*_H–H_*=*1.6 Hz, ^3^*J*_H–H_*=*6.6 Hz, 1 H, (*Z*)-C*H*_2_CH), 3.93–3.84 (m, 1 H, OC*H*), 2.74 (d, ^2^*J*_H–H_*=*11.7 Hz, 1 H, NC*H*_2_), 2.48 (d, ^2^*J*_H–H_*=*11.7 Hz, 1 H, NC*H*_2_), 2.26 (s, 3 H, NC*H*_3_), 2.18–2.05 (m, 2 H, NC*H*_2_), 1.90–1.72 (m, 2 H, CHC*H*_2_), 1.60–1.47 (m, 1 H, CH_2_C*H*_2_), 1.46–1.34 ppm (m, 1 H, CH_2_C*H*_2_); ^13^C NMR (100 MHz, CD_3_OD, 20 °C, TMS): *δ*=150.3 (CH_2_*C*H), 88.3 (*C*H_2_CH), 74.2 (O*C*H), 59.6 (*C*H_2_N), 55.3 (N*C*H2), 46.4 (N*C*H_3_), 29.0 (*C*H_2_), 22.8 ppm (*C*H_2_); MS (EI, 70 eV): *m*/*z* (%): 142 (100) [*M*]^+^, 98 (79) [C_6_H_12_N]^+^, 58 (21) [C_3_H_8_N]^+^; HRMS (ESI): *m*/*z* [*M*+H]^+^ calcd for C_8_H_15_NO: 142.1232, found: 142.1236.

***trans*****-*N***,***N*****-Dimethyl-2-(vinyloxy)cyclohexanamine (rac-3)**: Vinylacetate (10 mL), *trans*-2-(dimethylamino)cyclohexanol (600 mg, 4 mmol) and 6-methyl-2,2′-bipyridine (57.4 mg, 0.34 mmol) were added to a 20 mL microwave vial, and the mixture was vigorously stirred. Pd(O_2_CCF_3_)_2_ (69.6 mg, 0.21 mmol) was added, and the microwave vial was irradiated for 30 min at 100 °C. The crude was concentrated and filtered through a column of aluminum oxide (2 cm Φ, 4 cm height) with Et_2_O/Et_3_N (96:4, 200 mL). The acetylated by-product was separated from the vinylated product by DCVC using isohexane/Et_2_O/Et_3_N (100:0:0→80:26:4). After chromatography, the product was still contaminated with a small amount of 6-methyl-2,2′-bipyridine, and the product mixture was, therefore, purified by bulb-to-bulb distillation (70 °C, 60 mbar) yielding pure product rac-**3** as a colorless oil (206.0 mg, 1.2 mmol, 29 %): *R*_f_=0.2 (isohexane/EtOAc/Et_3_N, 80:16:4); ^1^H NMR (400 MHz, CDCl_3_, 25 °C, TMS): *δ*=6.35 (dd, ^3^*J*_H–H_*=*6.6, 14.1 Hz, 1 H, CH_2_C*H*), 4.29 (dd, ^2^*J*_H–H_*=*1.6 Hz, ^3^*J*_H–H_*=*14.1 Hz, 1 H, (*E*)-C*H*_2_CH), 3.99 (dd, ^2^*J*_H–H_*=*1.6 Hz, ^3^*J*_H–H_*=*6.6 Hz, 1 H, (*Z*)-C*H*_2_CH), 3.77–3.70 (m, 1 H, OC*H*), 2.54–2.48 (m, 1 H, NC*H*), 2.34 (s, 6 H, N(C*H*_3_)_2_), 2.17–2.10 (m, 1 H, OCHC*H*_2_), 1.92–1.81 (m, 2 H, OCHC*H*_2_ and NCHC*H*_2_), 1.76–1.68 (m, 1 H, NCHC*H*_2_), 1.34–1.13 ppm (m, 4 H, C*H*_2_C*H*_2_); ^13^C NMR (400 MHz, CDCl_3_, 25 °C, TMS): *δ*=150.8 (CH_2_*C*H), 87.7 (*C*H_2_CH), 78.4 (O*C*H), 66.0 (N*C*H), 41.0 (N(*C*H_3_)_2_), 31.2 (*C*H_2_), 24.9 (*C*H_2_), 24.7 (*C*H_2_), 24.1 ppm (*C*H_2_); MS (EI, 70 eV): *m*/*z* (%): 169 (34) [*M*]^+^, 126 (17) [C_8_H_16_N]^+^, 84 (100) [C_5_H_10_N]^+^; HRMS (ESI): *m*/*z* [M+H]^+^ calcd for C_10_H_19_NO: 170.1545, found: 170.1539.

**(2*S*)-2-((1,2-Bis(4-methoxyphenyl)ethoxy)methyl)-1-methylpyrrolidine (5 a)**: Prepared as described in the general procedure for the synthesis of diarylated products **5**, **6** and rac-**7**, but using 4-methoxyphenylboronic acid (**4 a**) as the arylating agent; the reaction was stirred for 24 h. Purification by DCVC afforded **5 a** as a yellow–brown oil (39 mg, 0.11 mmol, 52 % yield, 2.9:1 *d.r.*): *R*_f_=0.3 (isohexane/EtOAc/Et_3_N, 60:36:4): 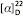
=−22.6° (*c*=11.4 in CHCl_3_); ^1^H NMR (400 MHz, CDCl_3_, 20 °C, TMS): *δ*=7.14 (d, ^3^*J*_H–H_=8.6 Hz, 2 H, Ar-H), 7.00 (d, ^3^*J*_H–H_=8.6 Hz, 2 H, Ar-H), 6.84 (d, ^3^*J*_H–H_=8.7 Hz, 2 H, Ar-H), 6.76 (d, ^3^*J*_H–H_=8.7 Hz, 2 H, Ar-H), 4.33 (dd, ^3^*J*_H–H_*=*6.0, 7.4 Hz, 1 H, ArC*H*), 3.80 (s, 3 H, Ar-OC*H*_3_), 3.77 (s, 3 H, Ar-OC*H*_3_), 3.28–3.18 (m, 2 H, Ar-C*H*_2_ and NC*H*), 2.95 (dd, ^3^*J*_H–H_*=*7.6 Hz, ^2^*J*_H–H_*=*13.7 Hz, 1 H, OC*H*_2_), 2.95–2.90 (m, 1 H, Ar-CH_2_), 2.78 (dd, *J=*5.8, 13.7 Hz, 1 H, OC*H*_2_), 2.41–2.30 (m, 1 H, NC*H*_2_), 2.31 (s, 3 H, NC*H*_3_), 2.25–2.15 (m, 1 H, NC*H*_2_), 1.86–1.75 (m, 1 H, CHC*H*_2_), 1.69–1.59 (m, 2 H, CHC*H*_2_ and CH_2_C*H*_2_), 1.43–1.32 ppm (m, 1 H, CH_2_C*H*_2_); ^13^C NMR (100 MHz, CDCl_3_, 20 °C, TMS): *δ*=159.0, 157.9, 134.0, 130.7, 130.5, 128.0, 113.6, 113.4, 83.7 (O*C*H), 71.8 (O*C*H_2_), 65.1 (N*C*H), 57.8 (Ar-O*C*H_3_), 55.2 (Ar-O*C*H_3_), 43.9 (N*C*H_3_), 41.5 (N*C*H_2_), 28.6 (*C*H_2_), 22.6 ppm (*C*H_2_); MS (EI, 70 eV): *m*/*z* (%): 356 (30) [*M*]^+^, 98 (15) [C_6_H_12_N]^+^, 84 (100) [C_5_H_10_N]^+^; HRMS (ESI): *m*/*z* [*M*+H]^+^ calcd for C_22_H_29_NO_3_: 356.2226, found: 356.2223.

**(*S*)-2-(((*S*)-1,2-Bis(4-methoxyphenyl)ethoxy)methyl)-1,1-dimethylpyrrolidin-1-ium iodide (11)**: MeI (0.5 mL) was added to a 4 mL glass vial containing **5 a** (26.8 mg, 75 μmol). The mixture was stirred for 3 h at 40 °C. When LC–MS showed total consumption of the starting material, MeI was evaporated leaving a yellow solution. The product was crystallized by vapor crystallization: hot isobutanol was used as the main solvent (∼0.6 mL) with a few drops of hot EtOAc. The vial was secured in a glass container partially filled with Et_2_O. The container was capped and left standing overnight. The next day, crystals were observed, and the container was transferred to the refrigerator were it was stored for an additional 2 d. When a satisfactory number of crystals were formed, the solvent was filtered, and the crystals were washed with Et_2_O and dried in vacuo to obtain product **11** as white crystals (14 mg, 0.028 mmol, 50 %).

## Supporting Information

Detailed facts of importance to specialist readers are published as ”Supporting Information”. Such documents are peer-reviewed, but not copy-edited or typeset. They are made available as submitted by the authors.
