# Supplementary material for: Development of Stereocontrolled Palladium(II)-Catalyzed Domino Heck/Suzuki β,α-Diarylation Reactions with Chelating Vinyl Ethers and Arylboronic Acids
Source: ChemistryOpen. 2012 Feb;1(1):49–56. doi: 10.1002/open.201100010 (PMC3922440; doi:10.1002/open.201100010)
Supplement: Supplementary file 1 [file open0001-0049-SD1.pdf]

ChemistryOPEN

Supporting Information

© Copyright Wiley-VCH Verlag GmbH & Co. KGaA, 69451 Weinheim, 2012

Development of Stereocontrolled Palladium(II)-Catalyzed Domino Heck/Suzuki β,α-Diarylation Reactions with Chelating Vinyl Ethers and Arylboronic Acids

Alejandro Trejos, Luke R. Odell, and Mats Larhed*[a]

open_201100010_sm_miscellaneous_information.pdf

General 2

Screening Design 3
Screening design using 4-methoxyphenylboronic acid (**4a**) 3
Screening design using 4-acetylphenylboronic acid (**4b**) 6

Table S3 9

Water concentration experiments 10
Experimental procedure for water concentration experiments 10

Determination of d.r. for product 5c by GC-MS analysis 11

Determination of d.r. for product 6c by ^{1}H NMR analysis 14

General procedure for synthesis of diarylated products 5, 6 and 7(rac). 17
(*S*)-1-methyl-2-((vinyloxy)methyl)pyrrolidine, product **1** 18
(*R*)-1-methyl-3-(vinyloxy)piperidine, product **2** 21
trans-N,N-Dimethyl-2-(vinyloxy)cyclohexanamine, product **3(rac)** 24
(2*S*)-2-((1,2-Bis(4-methoxyphenyl)ethoxy)methyl)-1-methylpyrrolidine, product **5a** 27
4,4'-(1-((-1-methylpyrrolidin-2-yl)methoxy)ethane-1,2-diyl)diacetylbenzene, product **5b** 30
(2*S*)-2-((1,2-Diphenylethoxy)methyl)-1-methylpyrrolidine, product **5c** 33
(2*S*)-2-((1,2-Bis(2-methoxyphenyl)ethoxy)methyl)-1-methylpyrrolidine, product **5d** 36
(2*S*)-2-((1,2-Di-p-tolylethoxy)methyl)-1-methylpyrrolidine, product **5e** 39
(2*S*)-2-((1,2-Di-m-tolylethoxy)methyl)-1-methylpyrrolidine, product **5f** 42
(2*S*)-2-((1,2-Di(naphthalen-2-yl)ethoxy)methyl)-1-methylpyrrolidine, product **5h** 45
(2*S*)-2-((1,2-Bis(4-bromophenyl)ethoxy)methyl)-1-methylpyrrolidine, product **5i** 48
4,4'-(1-((*S*)-1-Methylpyrrolidin-2-yl)methoxy)ethane-1,2-diyl)dibenzaldehyde, product **5j** 50
(2*S*)-2-((1,2-Bis(4-(trifluoromethyl)phenyl)ethoxy)methyl)-1-methylpyrrolidine, product **5k** 53
(3*S*)-3-(1,2-Bis(4-methoxyphenyl)ethoxy)-1-methylpiperidine, product **6a** 56
(3*S*)-3-(1,2-Diphenylethoxy)-1-methylpiperidine, product **6c** 59
(3*S*)-3-(1,2-Di-p-tolylethoxy)-1-methylpiperidine, product **6e** 62
(3*S*)-3-(1,2-Di-m-tolylethoxy)-1-methylpiperidine, product **6f** 65
(3*S*)-3-(1,2-Di-o-tolylethoxy)-1-methylpiperidine, product **6g** 68
(3*S*)-3-(1,2-Di(naphthalen-2-yl)ethoxy)-1-methylpiperidine, product **6h** 71
(3*S*)-3-(1,2-Bis(4-bromophenyl)ethoxy)-1-methylpiperidine, product **6i** 74
(3*S*)-3-(1,2-Bis(4-(trifluoromethyl)phenyl)ethoxy)-1-methylpiperidine, product **6k** 77
trans-2-(1,2-Diphenylethoxy)-*N*,*N*-dimethylcyclohexanamine, product **7(rac)** 80
(*S*)-2-(((*S*)-1,2-Bis(4-methoxyphenyl)ethoxy)methyl)-1,1-dimethylpyrrolidin-1-ium, product **10** 84
Experiments performed with addition of equimolar amounts of **4e** and **4j** 86
Experiments performed with addition of the chiral ligand (*R*)-BINAP 88

References 90

General

Column chromatography was performed on Merck silica gel 60 (40–63 µm). Analytical thin layer chromatography was performed using aluminium sheets precoated with silica gel 60 F_{254}. Chromatographic spots were visualized using UV-detection and/or ethanolic ninhydrin solution (2%) or ethanolic phosphomolybdic acid (5%) followed by heating. Dry Column Vacuum Chromatography (DCVC) was performed using silica gel 60 (40-63 µm, Merck). Preparative Thin Layer Chromatography (PTLC) was performed using Merck PLC Silica gel 60 F_{254}, 2 mm. Analytical GC-MS was performed on a Varian Saturn 3900/2100 system using a CP-SIL 5 CB Low Bleed (30 m x 0.25 mm) capillary column or CP-SIL 8 CB Low Bleed (30 m x 0.25 mm) capillary column and EI ionization (70-300°C, 30°C min^{-1} or at 180-190°C, 0.2°C min^{-1}). Analytical RPHPLC-MS was performed on a Gilson HPLC system with a Finnigan AQA ESI quadropole mass spectrometer with electrospray ionization and using an Onyx Monolithic C18 4.6 × 50mm (Phenomenex) with CH_3CN/H_2O in 0.05% aqueous HCOOH as mobile phase at a flow rate of 4 mL/min. Optical rotation was measured in a Perkin-Elmer 241 polarimeter (Na_{λ}, 589 nm). ^1H and ^{13}C NMR spectra were recorded on Varian Mercury Plus instruments; ^1H at 399.9 MHz and ^{13}C at 100.6 MHz. Exact molecular masses were determined on Micromass Q-Tof2 mass spectrometer equipped with an electrospray ion source. Collection of X-ray data for compound (*S,S*)-**11** was performed at the Latvian Institute of Organic Synthesis, Riga, Latvia. Screening design was planned with assistance of MODDE, version 9.0.0.0, sept 30 2009, licensed by Umetrics AB.

Screening Design

Screening design using 4-methoxyphenylboronic acid (4a)

(S)-1-Methyl-2-((vinyloxy)methyl)pyrrolidine **1** (30 mg, 0.21 mmol) was added to a reaction vial containing 4-methoxyphenylboronic acid (**4a**), 1,4-benzoquinone (*p*-BQ) (23.8 mg, 0.22 mmol) and 1,4-dioxane. The mixture was stirred until a clear solution was obtained. $Pd(O_2CCF_3)_2$ was thereafter added and the vial was sealed and heated in a metal heating block at the temperature stated in Table S1 for 24 h. The yield of **5a** was determined by ^1H NMR analysis of the crude mixture using DMF as internal standard. The d.r. was determined by GC-MS and ^1H NMR of the crude mixture.

Table S1 D-optimal design of experiments for the reaction between **1** and **4a**.

Exp No	Exp Name	Run Order	$Pd(O_2CCF_3)_2$ (Equiv)	1,4-dioxane (mL)	Temp (°C)	4a (Equiv)	Yield (%)	d.r.
1	N1	11	0.025	40	25	3	51	7.3
2	N2	2	0.025	80	40	4	38	39
3	N3	10	0.025	120	60	5	28	19
4	N4	3	0.05	40	40	5	57	27
5	N5	1	0.05	80	60	3	50	4
6	N6	12	0.05	120	25	4	36	19
7	N7	8	0.1	40	60	4	43	2.2
8	N8	4	0.1	80	25	5	58	6.1
9	N9	5	0.1	120	40	3	32	1.9
10	N10	7	0.05	80	40	4	67	13.3
11	N11	9	0.05	80	40	4	59	19
12	N12	6	0.05	80	40	4	62	8.9

The d.r. values were subjected to a logarithmical transformation in order to obtain a normal distribution of the response distribution.

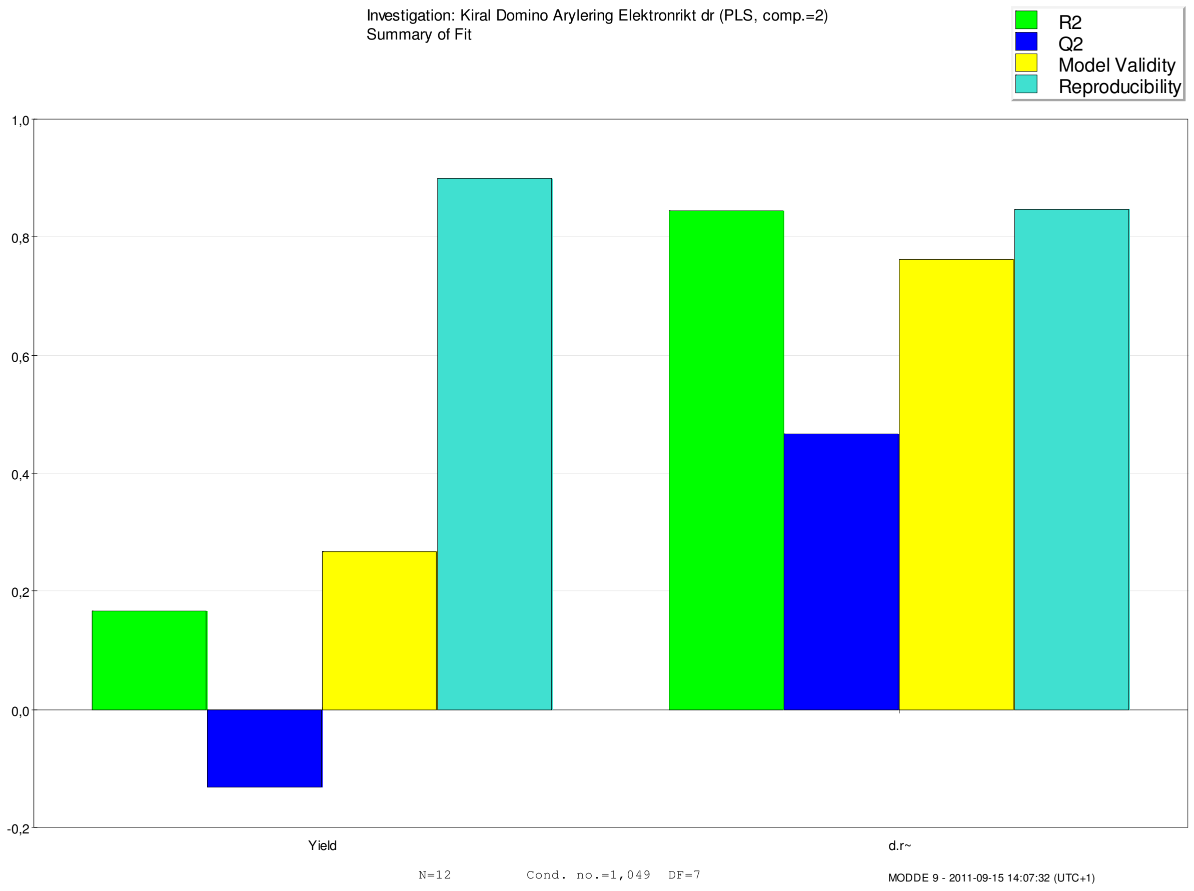

Figure S1 Summary of the basic model. R^2 = 0.16 and Q^2 = -0.13 for the yield of **5a** suggest that the model has no validity for predicting the principal components influencing the yield. R^2 = 0.81 and Q^2 = 0.46 for the d.r. of **5a** suggest that the model could be useful for explaining the principal components influencing the d.r. of the reaction.

The R^2 and Q^2 for the yield are below 0.5 and 0.1 (Figure S1) therefore the model is not valid for yield prediction of **5a**. Thus, only the d.r. was taken into account when the model was evaluated (R^2 = 0.81, Q^2 = 0.46).

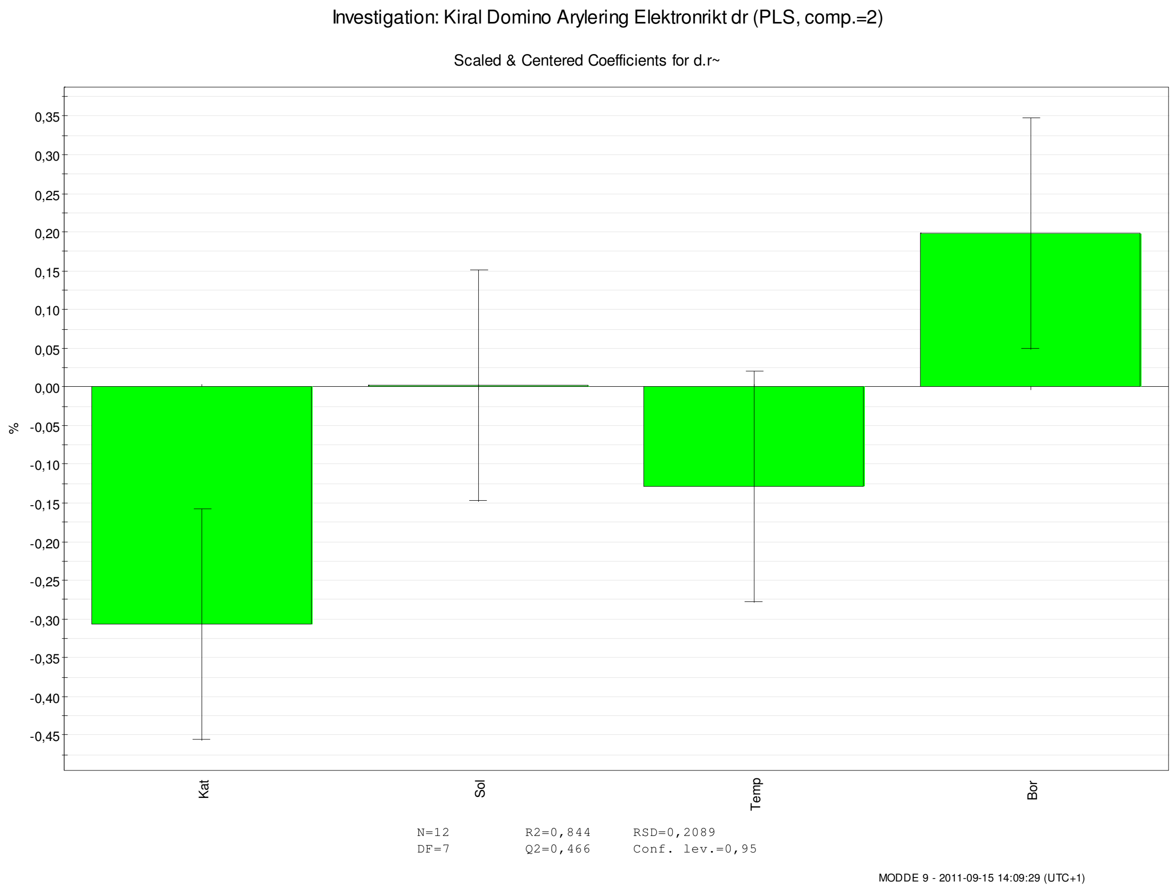

Figure S2 Coefficient plot showing the most important terms determining the d.r. of **5a** according to the model.

According to the coefficient plot (Figure S2), low amount of catalyst and increased amount of 4-methoxyboronic acid (**4a**) were beneficial for increasing the stereoselectivity. The temperature, according to this model, is not a statistically significant factor for the stereochemical outcome of the reaction within the studied domain. The amount of solvent is virtually a negligible factor for the stereoselectivity of the reaction.

Screening design using 4-acetylphenylboronic acid (4b)

(*S*)-1-Methyl-2-((vinyloxy)methyl)pyrrolidine **1** (30.0 mg, 0.21 mmol) was added to a reaction vial containing 4-acetylphenylboronic acid (**4b**), *p*-BQ (23.8 mg, 0.22 mmol) and 1,4-dioxane. The mixture was stirred until a clear solution was obtained. $Pd(O_2CCF_3)_2$ was thereafter added and the vial was then sealed and heated in a metal heating block at the temperature stated in Table S2 for 24 h. The yield of **5a** was determined by ^1H NMR analysis of the crude mixture using DMF as internal standard. The d.r. was determined by GC-MS and ^1H NMR of the crude mixture.

Table S2 D-optimal design of experiments for the reaction between 1 and 4b.

Exp No	Exp Name	Run Order	$Pd(O_2CCF_3)_2$ (Equiv)	1,4-dioxane (mL)	Temp (°C)	4b (Equiv)	Yield(%)	d.r.
1	N1	11	0.025	40	25	3	53	4.7
2	N2	2	0.025	80	40	4	76	3.5
3	N3	10	0.025	120	60	5	88	3.8
4	N4	3	0.05	40	40	5	88	2.6
5	N5	1	0.05	80	60	3	41	1.3
6	N6	12	0.05	120	25	4	77	1.9
7	N7	8	0.1	40	60	4	29	1.6
8	N8	4	0.1	80	25	5	69	3.2
9	N9	5	0.1	120	40	3	53	1.8
10	N10	7	0.05	80	40	4	83	2.6
11	N11	9	0.05	80	40	4	67	2.4
12	N12	6	0.05	80	40	4	82	3.8

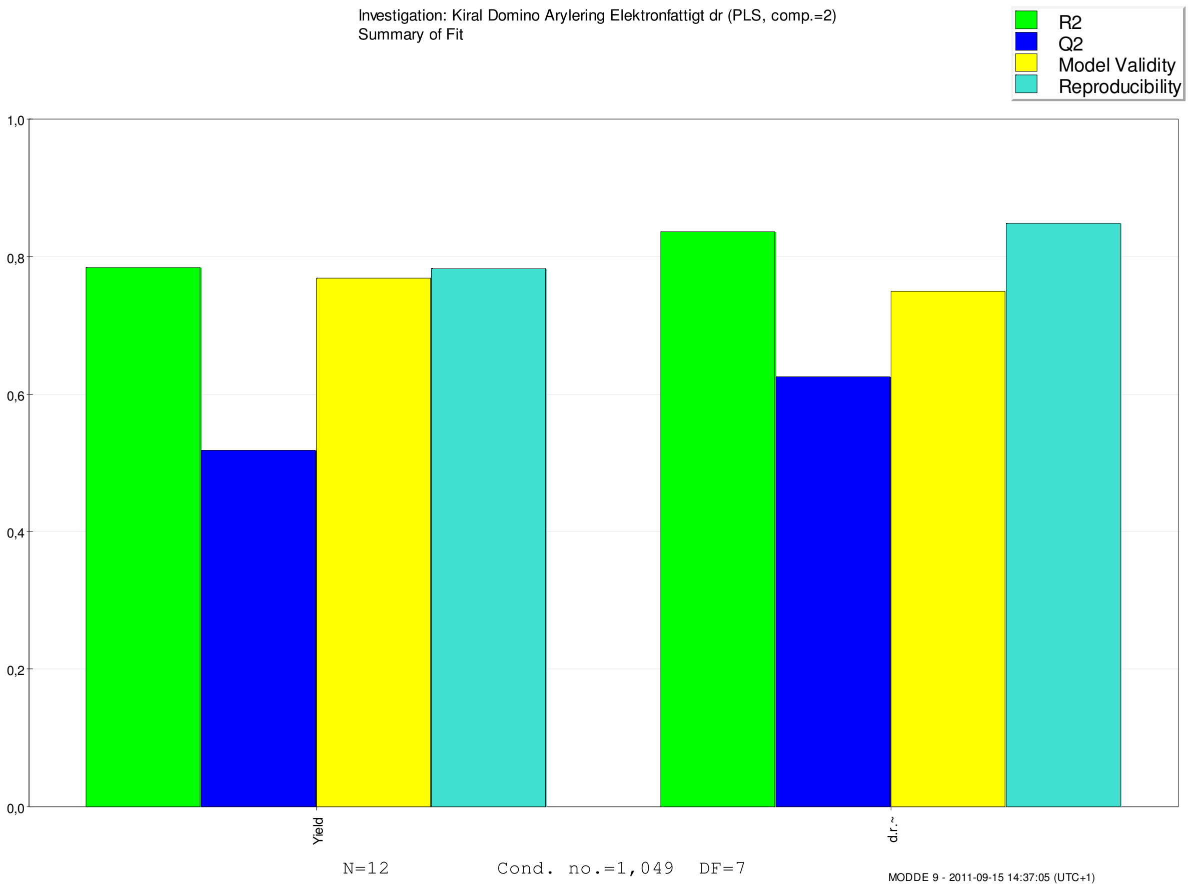

Figure S3 Summary of the basic model. R^2 = 0.78 and Q^2 = 0.51 for the yield of **5b** suggest that the model is valid and may be used for prediction of the principal components influencing the yield. R^2 = 0.84 and Q^2 = 0.62 for the d.r. of **5b** suggest that the model enjoys significance for explaining the principal components influencing the d.r. of the reaction.

The model (Figure S3) is significant for both explaining the principal components influencing the yields (R^2= 0.78, Q^2 = 0.51) and the d.r. (R^2 = 0.84, Q^2 = 0.62). Thus, both results will be discussed in the following figures.

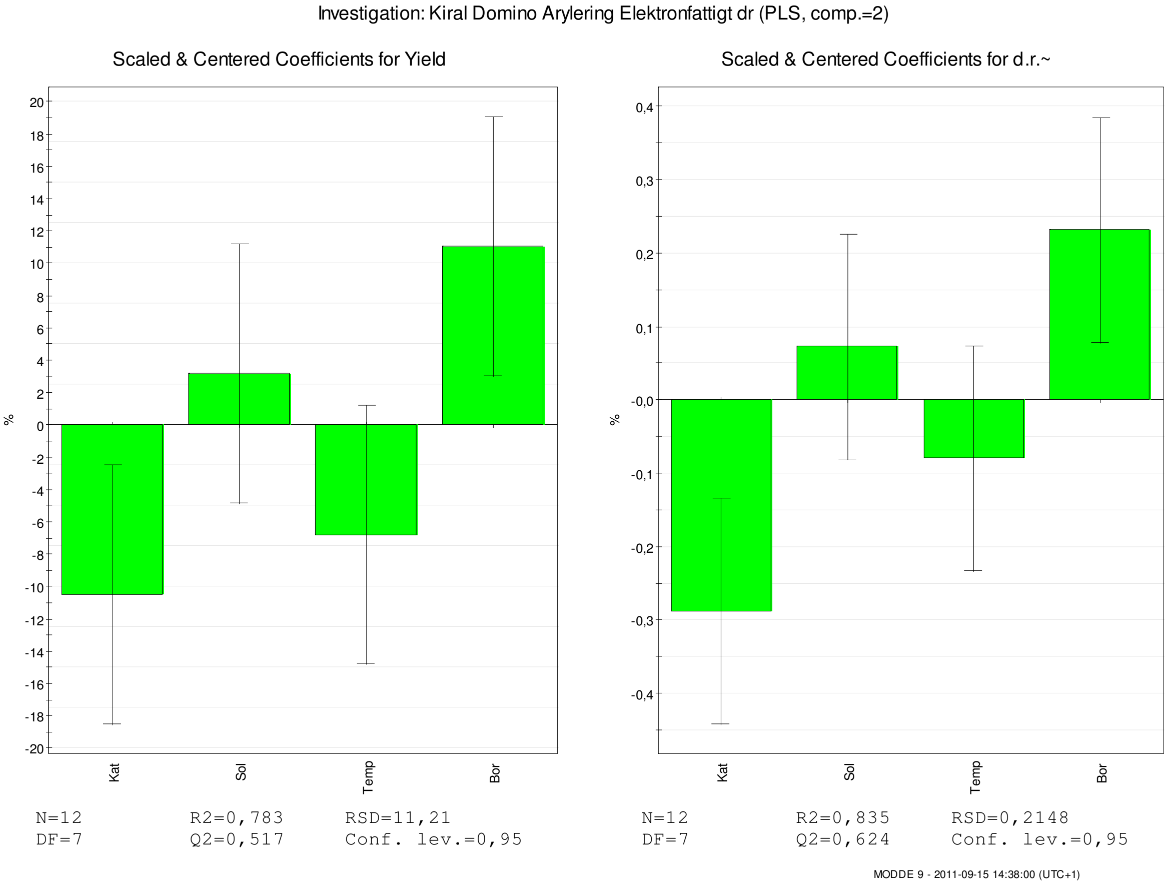

Figure S4 Coefficient plot showing the most important terms influencing the yields and d.r. according to the model.

As can be appreciated in Figure S4, lower amounts of catalyst and increased amounts of **4b** positively influence the yields and stereoselectivity in the reaction beteen **1** and **4b**, which is in accordance with the findings when electron-rich **4a** was reacted with **1**. The temperature and amount of solvent are not statistically relevant factors for the outcome of the reaction.

Table S3

Table S3 is a summary of the results obtained using different arylboronic acids (**4**) post screening design. As shown, the stereoselectivity and yields varied heavily and did not seem to correlate to the electronic nature of **4**. The temperature was increased from 25°C, as suggested by the model, to 40°C due to slow reaction rates and incomplete consumption of starting material **1** at 25°C.

Table S3 Results from the reaction between **1** and **4**.

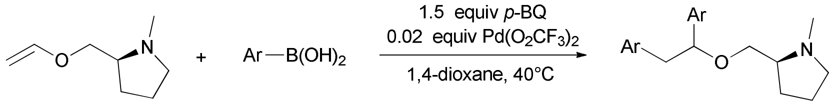

1 **4** **5**

Entry	Ar	Yield (%)[a]	d.r.[b]
1	4-$CH_3C_6H_4$	52	1.8:1
2	3-$CH_3C_6H_4$	30	1.4:1
3	4-$Me_2NC_6H_4$	Traces	n.d
4	2-$MeOC_6H_4$	Traces	n.d.
5	4-$F_3CC_6H_4$	47	1.6:1
6	2-naphthyl	34	7.3:1
7	1-naphthyl	Traces	n.d.
8	Ph	66	1.4:1
9	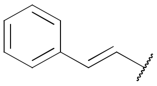	Traces	n.d.
10	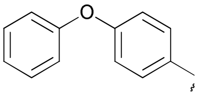	Traces	n.d.
11	4-$HOCC_6H_4$	44	1.6:1

(S)-1-Methyl-2-((vinyloxy)methyl)pyrrolidine **1** (0.21 mmol, 1 equiv), arylboronic acid **4** (4 equiv), *p*-BQ (1.5 eq), $Pd(O_2CCF_3)$ (0.02 equiv), 1,4-Dioxane (1.5 mL), temp (40°C). n.d. = no data. [a] Isolated yields. [b] Determined by ^{1}H NMR of the crude mixture or by GC-MS analysis of the crude mixture.

The varied results presented by this table show that there should be factors other than catalyst loading and amount of **4** in the reaction that influenced the stereochemical outcome of the reaction.

Water concentration experiments

Anhydrous 1,4-dioxane was obtained from Sigma-Aldrich and used without further purification. The reaction vials used in the experiments were dried overnight in a oven at 130°C and allowed to cool under vacuum prior to usage. All reactions were prepared in a N_2(g) atmosphere using a N_2(g)-flushed AtmosBag provided by Sigma-Aldrich. *p*-BQ and phenylboronic acid (**4c**) were re-crystallized in ethanol (99%) and diethyl ether respectively, and stored under vacuum before use. The palladium salt $Pd(O_2CCF_3)_2$ was obtained from Strem Chemicals and used as received.

Experimental procedure for water concentration experiments

An 8 mL reaction vial was charged with phenylboronic acid (**4c**, 103.6 mg, 0.84 mmol), *p*-BQ (34.7 mg, 0.32 mmol), (*S*)-1-methyl-2-((vinyloxy)methyl)pyrrolidine **1** or (*R*)-1-methyl-3-(vinyloxy)piperidine **2** (30 mg, 0.21 mmol) and 1,4-dioxane (the volume of used 1,4-dioxane varied depending on the amount of water used and the total volume was held constant at 1.5 mL). The mixture was homogenized by vigorous stirring, thereafter $Pd(O_2CCF_3)_2$ (2.8 mg, 8.4 µmol) and water (when needed) were added and the reaction mixture was heated in a metal heating block at 40°C for 24 h. The mixture was then diluted with EtOAc (20 mL), and washed with NaOH (1M, 3 x 15 mL). The aqueous phase was analyzed by LC-MS to ensure no product could be detected. The organic phase was dried with K_2CO_2, filtered and concentrated. The crude mixture was then analyzed by GC-MS and ^{1}H NMR (in $CDCl_3$, containing a known amount of DMF serving as an internal standard). For compound **5c**, the d.r. could be calculated by GC-MS analysis and confirmed by ^{1}H NMR. The separation between the GC-signals for the products formed by compound **2** (i.e. **6c**) were not satisfactory and the d.r. for compound **6c** could only be calculated by ^{1}H NMR analysis of the signals at 4.52 ppm and employing a pseudo weighting function until satisfactory baseline separation was achieved.

Determination of d.r. for product 5c by GC-MS analysis

Product 5c, (2S)-2-((1,2-Diphenylethoxy)methyl)-1-methylpyrrolidine, 0.5 equiv water

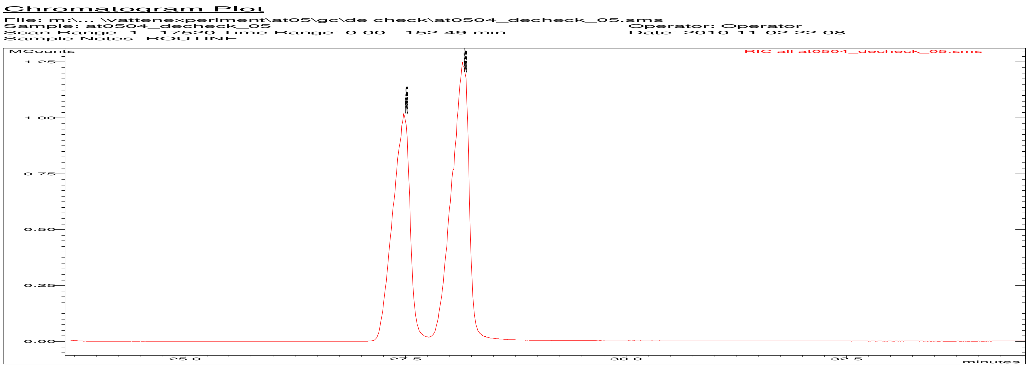

Product 5c, (2S)-2-((1,2-Diphenylethoxy)methyl)-1-methylpyrrolidine, 1 equiv water

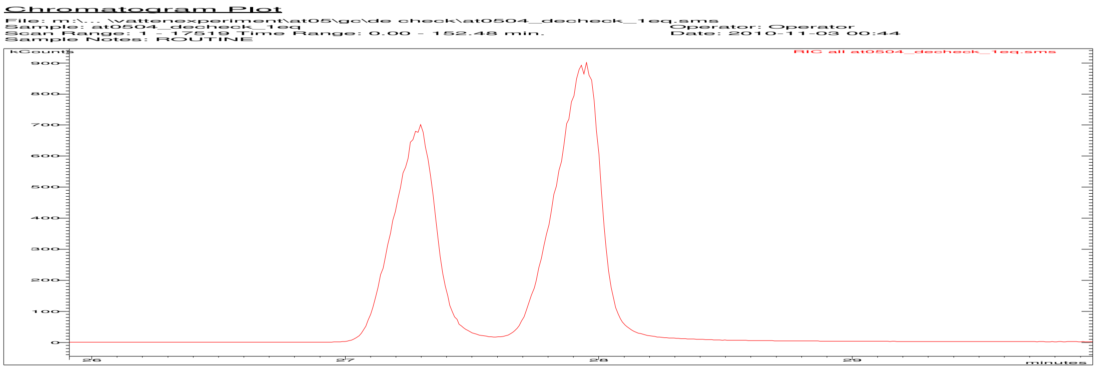

Product 5c, (2S)-2-((1,2-Diphenylethoxy)methyl)-1-methylpyrrolidine, 2 equiv water

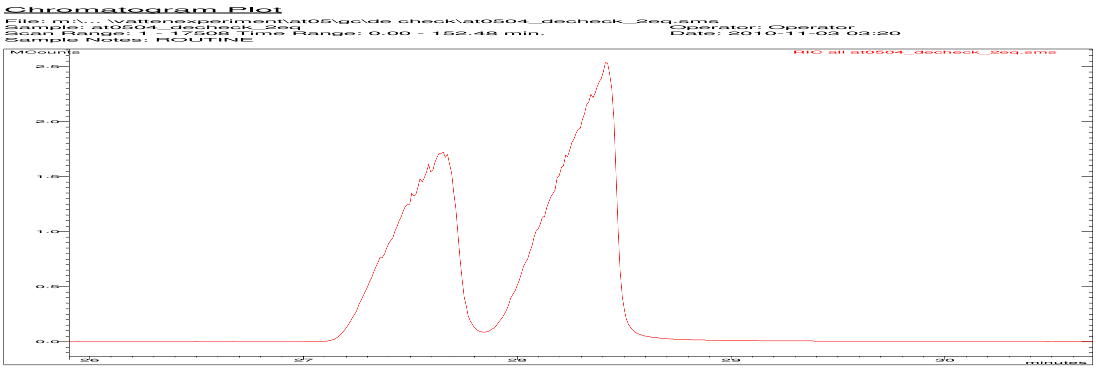

Product 5c, (2S)-2-((1,2-Diphenylethoxy)methyl)-1-methylpyrrolidine, 5 equiv water

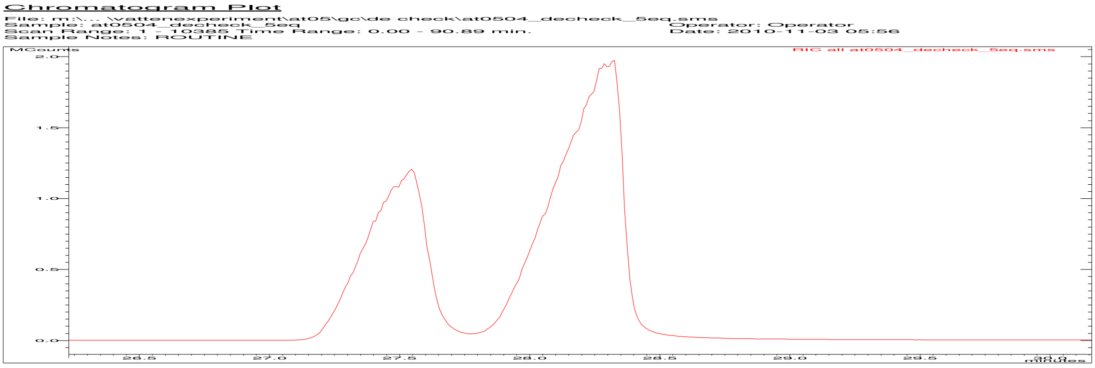

Product 5c, (2S)-2-((1,2-Diphenylethoxy)methyl)-1-methylpyrrolidine, 20 equiv water

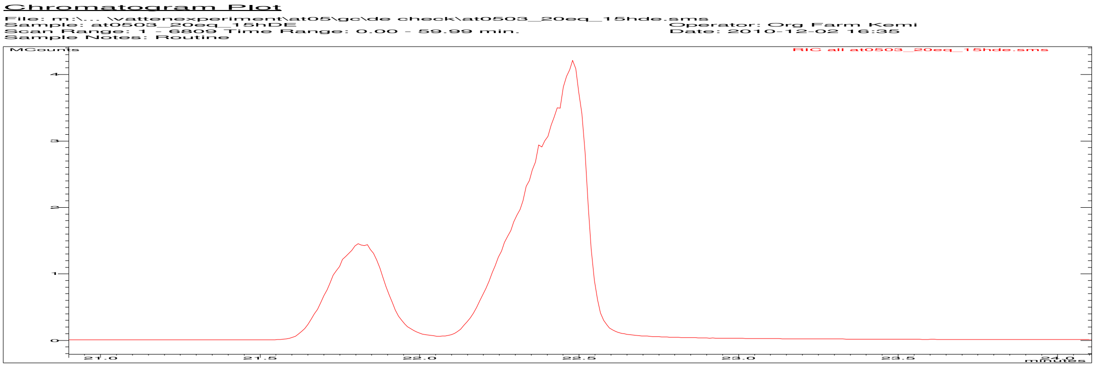

Product 5c, (2S)-2-((1,2-Diphenylethoxy)methyl)-1-methylpyrrolidine, 50 equiv water

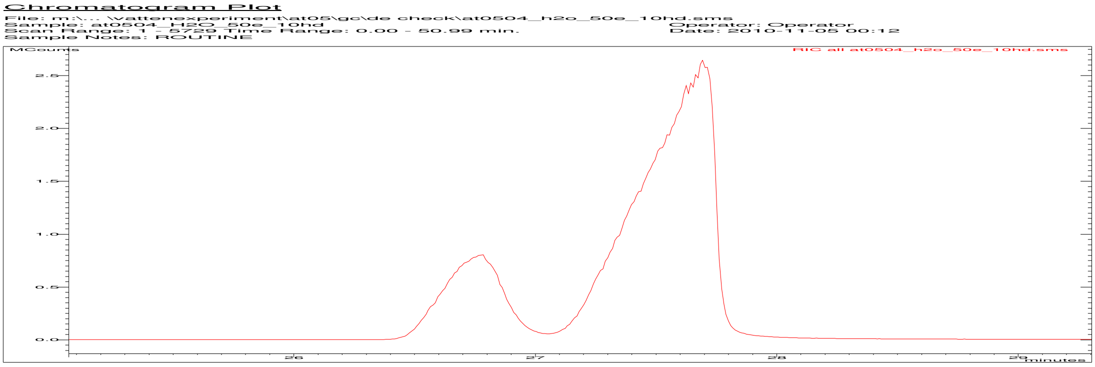

Product 5c, (2S)-2-((1,2-Diphenylethoxy)methyl)-1-methylpyrrolidine, 100 equiv water

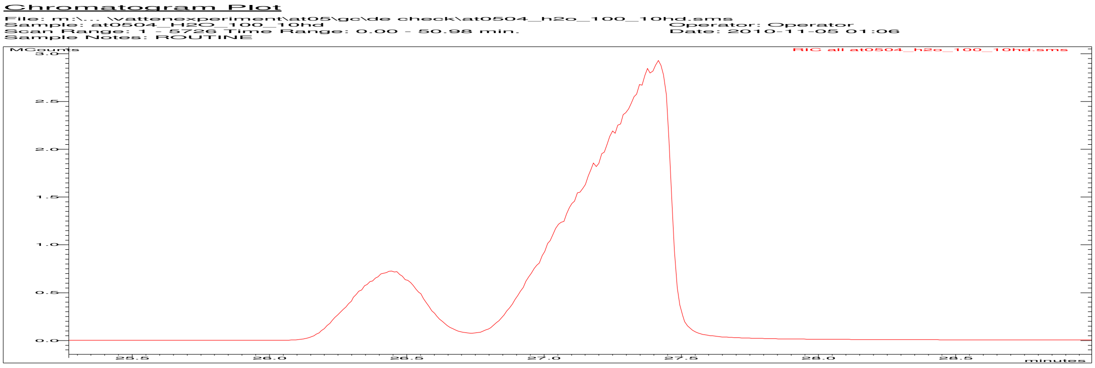

Product 5c, (2S)-2-((1,2-Diphenylethoxy)methyl)-1-methylpyrrolidine, 200 equiv water

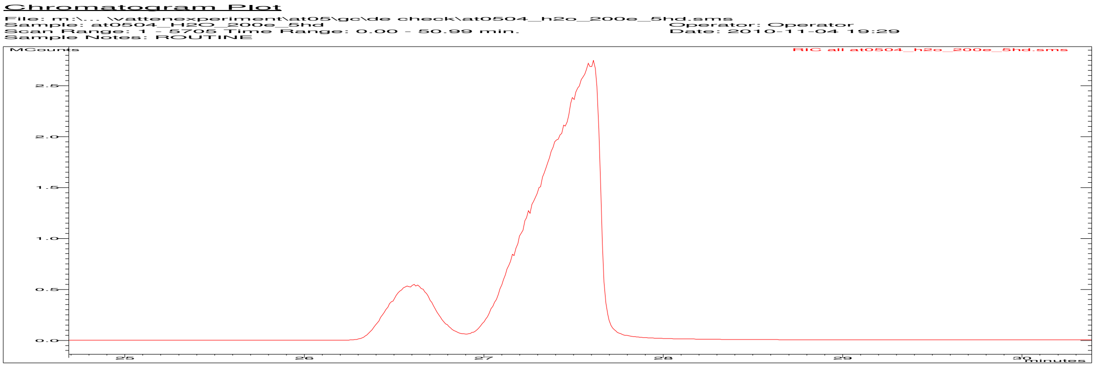

Determination of d.r. for product 6c by ^{1}H NMR analysis

Product 6c, (3R)-3-(1,2-Diphenylethoxy)-1-methylpiperidine, 0.5 equiv water

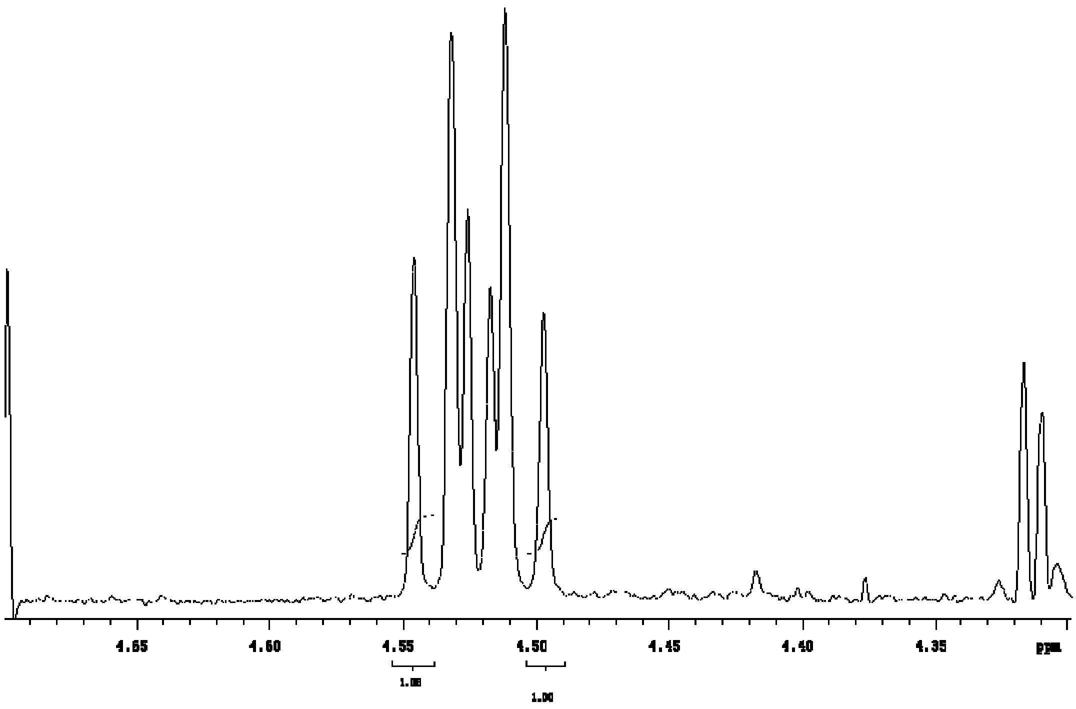

Product 6c, (3R)-3-(1,2-Diphenylethoxy)-1-methylpiperidine, 1 equiv water

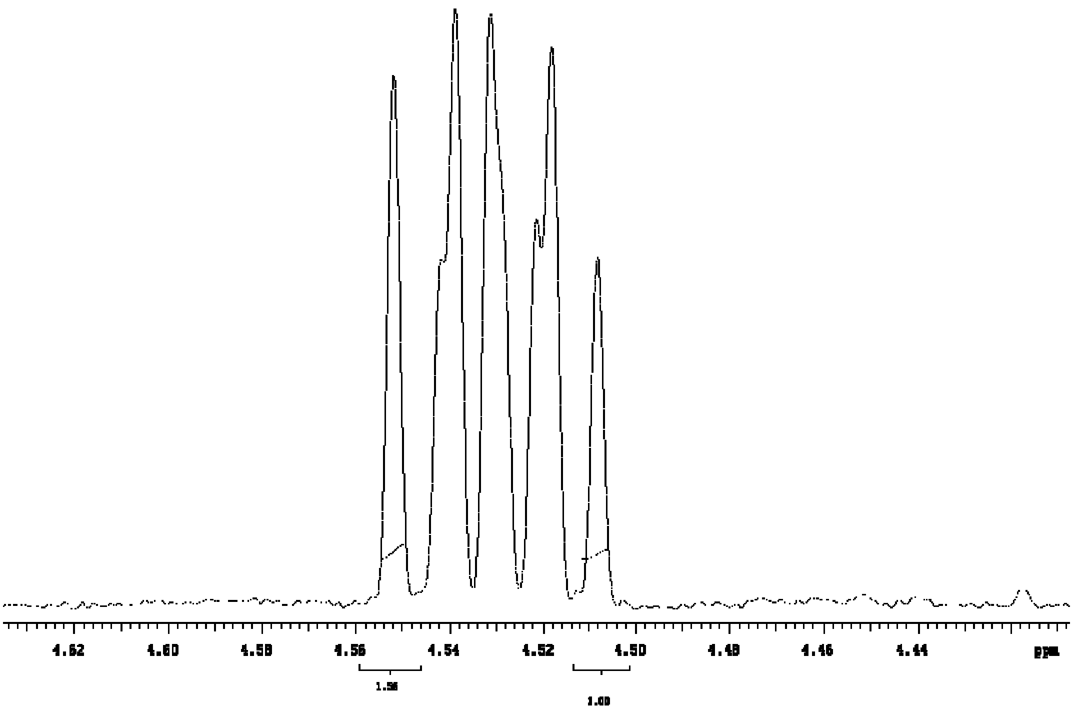

Product 6c, (3R)-3-(1,2-Diphenylethoxy)-1-methylpiperidine, 2 equiv water

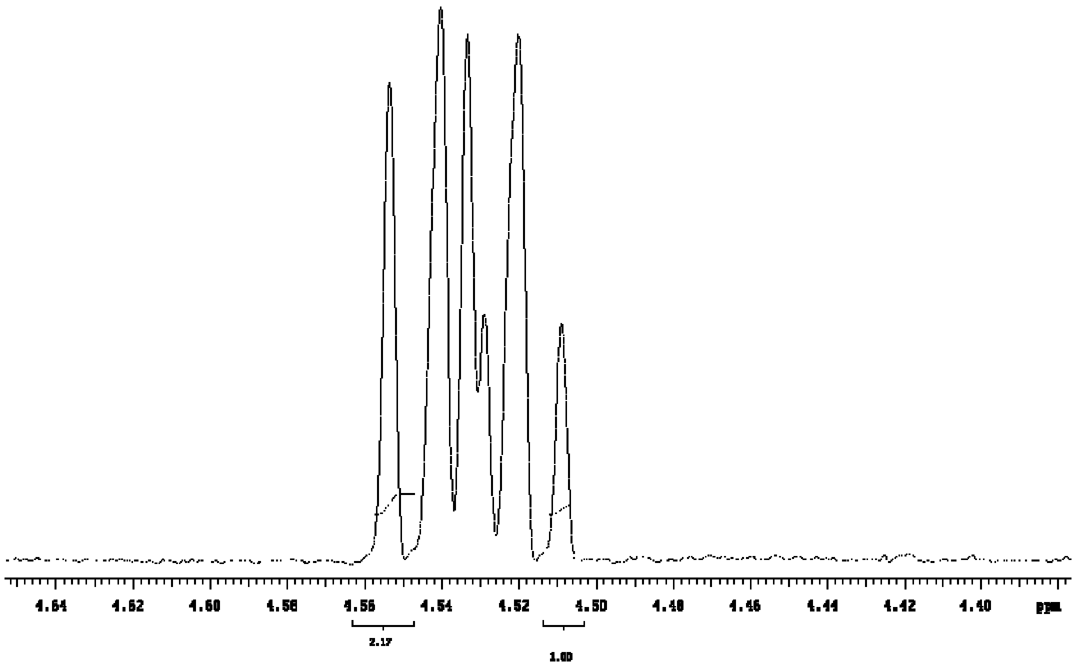

Product 6c, (3R)-3-(1,2-Diphenylethoxy)-1-methylpiperidine, 5 equiv water

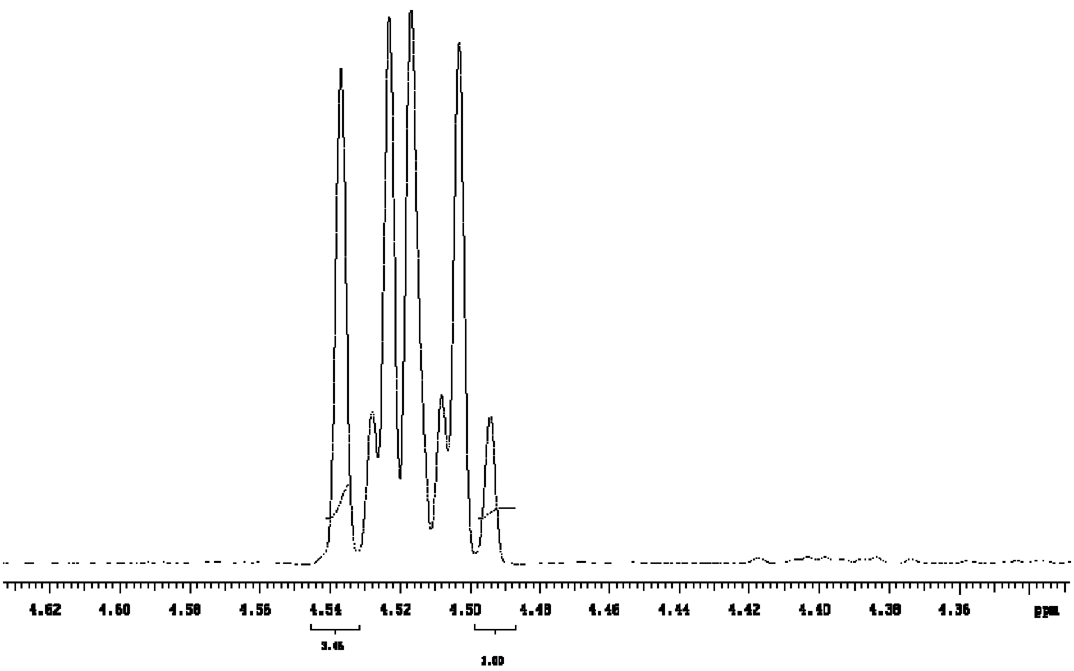

Product 6c, (3R)-3-(1,2-Diphenylethoxy)-1-methylpiperidine, 10 equiv water

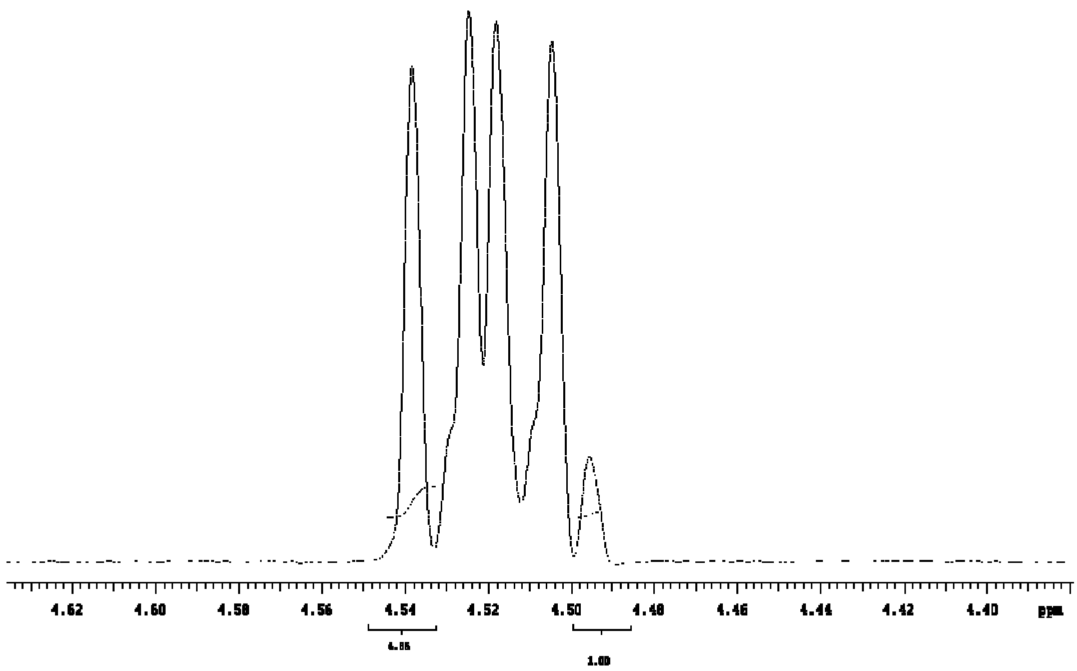

Product 6c, (3R)-3-(1,2-Diphenylethoxy)-1-methylpiperidine, 20 equiv water

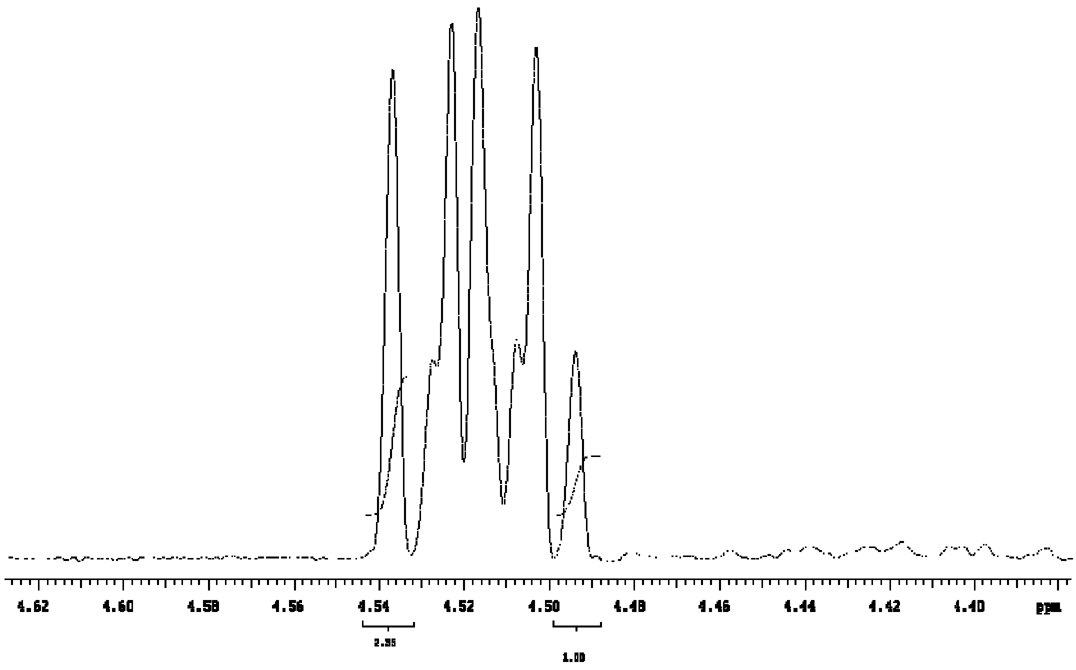

General procedure for synthesis of diarylated products 5, 6 and 7(rac).

An 8 mL reaction vial was charged with **4** (4 equiv), *p*-BQ (1.5 equiv), the appropriate vinyl ether (0.21 mmol for **1** and **2**, 0.18 mmol for **3(rac)**) and anhydrous 1,4-dioxane (1.5 mL). The mixture was homogenized by vigorous stirring, thereafter $Pd(O_2CCF_3)_2$ (0.04 equiv) and water (10 equiv) were added to the reaction mixture, the vial was capped and heated in a metal heating block at 40°C for 24 h or 36 h. The crude mixture was filtered through a short Al_2O_3-column (2 cm Φ, 3 cm height) and eluted with 200 mL EtOAc:Et_3N (10:1) or until no more product was detected (typically, 100 mL eluent was sufficient). The solvent was evaporated under reduced pressure and the crude mixture was analyzed by GC-MS and/or NMR-spectrometry to elucidate the diastereoselectivity. The product was thereafter purified by gradient elution Dry Column Vacuum Chromatography[1] (DCVC) using isohexane:diethyl ether:Et_3N as eluent system (a maximum of 4% Et_3N was used).

(S)-1-methyl-2-((vinyloxy)methyl)pyrrolidine, product 1

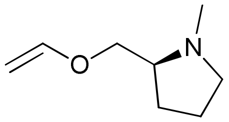

To a 250 mL three-necked-flask were added 1-methylpiperidin-3-ol (5.00 g, 43.4 mmol) dissolved in ethyl vinyl ether (65 mL), 2,2'-bipyridyl (406 mg, 2.6 mmol) and $Pd(OAc)_2$ (433 mg, 2.0 mmol) and refluxed at 75°C. Due to the volatile nature of the starting material, additional ethyl vinyl ether was added several times during the reaction to avoid complete evaporation of this starting reactant as the reaction flask was kept open to drive the equilibrium towards product formation by evaporation of EtOH that is formed as by-product (please note that when monitoring was not possible, the collection funnel was closed and no starting material could then evaporate). The mixture was monitored by GC-MS. When complete consumption of the starting material was noted (usually 4-5 days of reaction time), the temperature was decreased to rt, the ethyl vinyl ether was evaporated and the crude material was diluted with 100 mL of ethyl acetate and washed twice with 2M aqueous NaOH (addition of brine was noted to be convenient for phase separation). The organic phase was dried over K_2CO_3, concentrated under vacuum and the crude material was finally purified by bulb-to-bulb distillation (65°C, 50 mbar) or by silica gel chromatography (isohexane:diethyl ether:triethylamine 90:6:4) in 46% yield (2.80 g, 19.8 mmol). $[\alpha]_D^{22}$= -46.7° (c=8.5 in MeOH); ^{1}H NMR ($CDCl_3$, 20°C, TMS): δ=6.05 (dd, *J*=6.8, 14.4 Hz, 1H), 4.18 (dd, *J*=2.0, 14.4 Hz, 1H), 3.98 (dd, *J*=2.0, 6.8 Hz, 1H), 3.70 (dd, *J*=5.1, 9.8 Hz, 1H), 3.16 (dd, *J*=5.4, 9.8 Hz, 1H), 3.08 (m, 1H), 2.52-2.44 (m, 1H), 2.40 (s, 3H), 2.23 (m, 1H), 1.99-1.89 (m, 1H), 1.83-1.61 (m, 3H); ^{13}C NMR ($CDCl_3$, 20°C, TMS) *δ*=151.9, 86.2, 70.2, 64.2, 57.7, 41.4, 28.5, 22.8; MS (70 eV): *m/z* (%): 142 (34) [M^+], 98 (35) [$C_6H_{12}N^+$], 84 (100) [$C_5H_{10}N^+$]; HRMS (ESI): *m/z* calcd for $C_8H_{15}NO$ (M + H^+): 142.1232; Found: 142.1237.

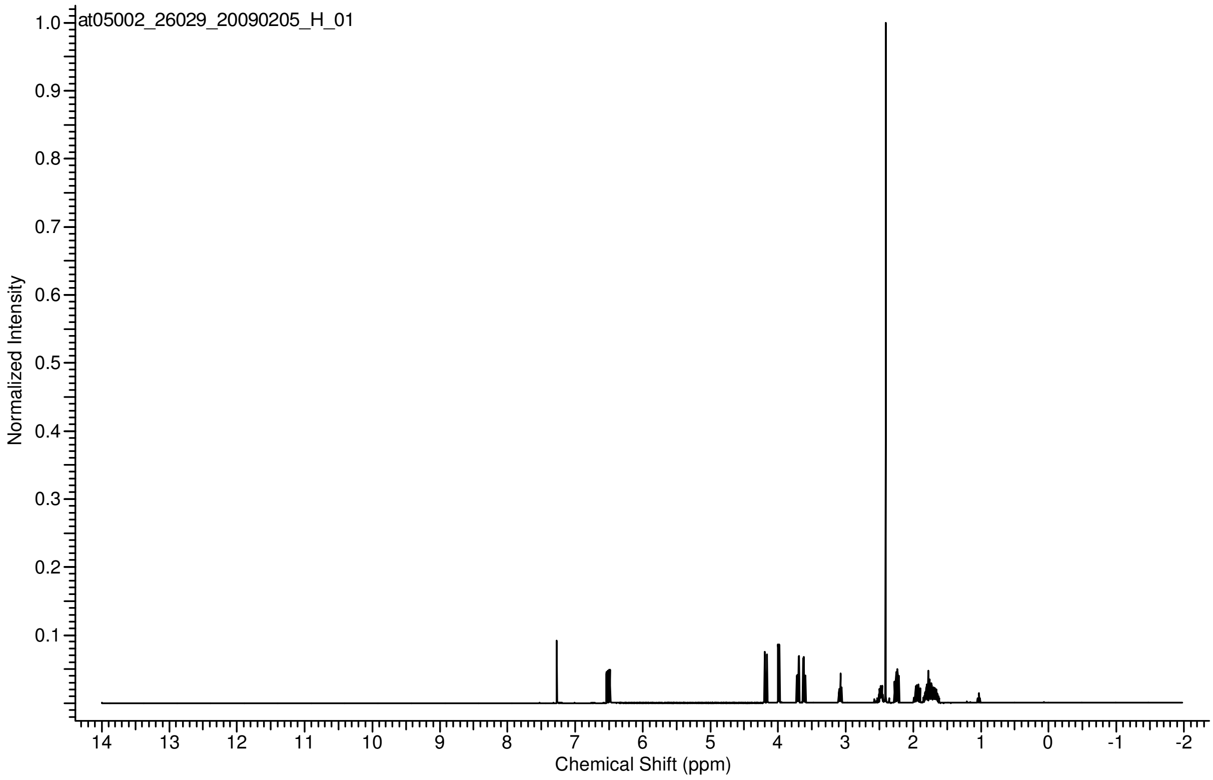
at05002_26029_20090205_H_01
Normalized Intensity
Chemical Shift (ppm)

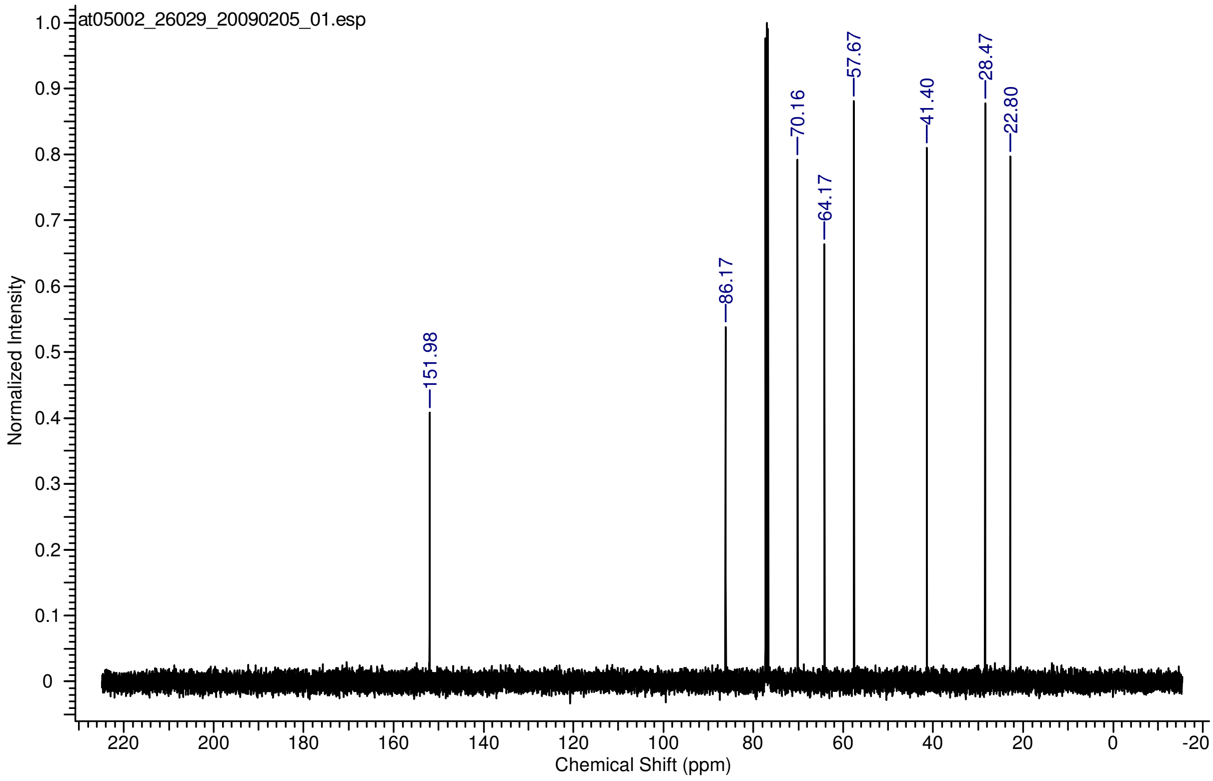
at05002_26029_20090205_01.esp
151.98
86.17
70.16
64.17
57.67
41.40
28.47
22.80
Normalized Intensity
Chemical Shift (ppm)

Chromatogram Plot

File: m:\... \at053 chiral heck-su\avslutning\gc\at05_26179_fr15.sms
Sample: at05_26179_fr15 Operator: Operator
Scan Range: 1 - 1162 Time Range: 0.00 - 11.16 min. Date: 09/09/2009 12:28
Sample Notes: ROUTINE

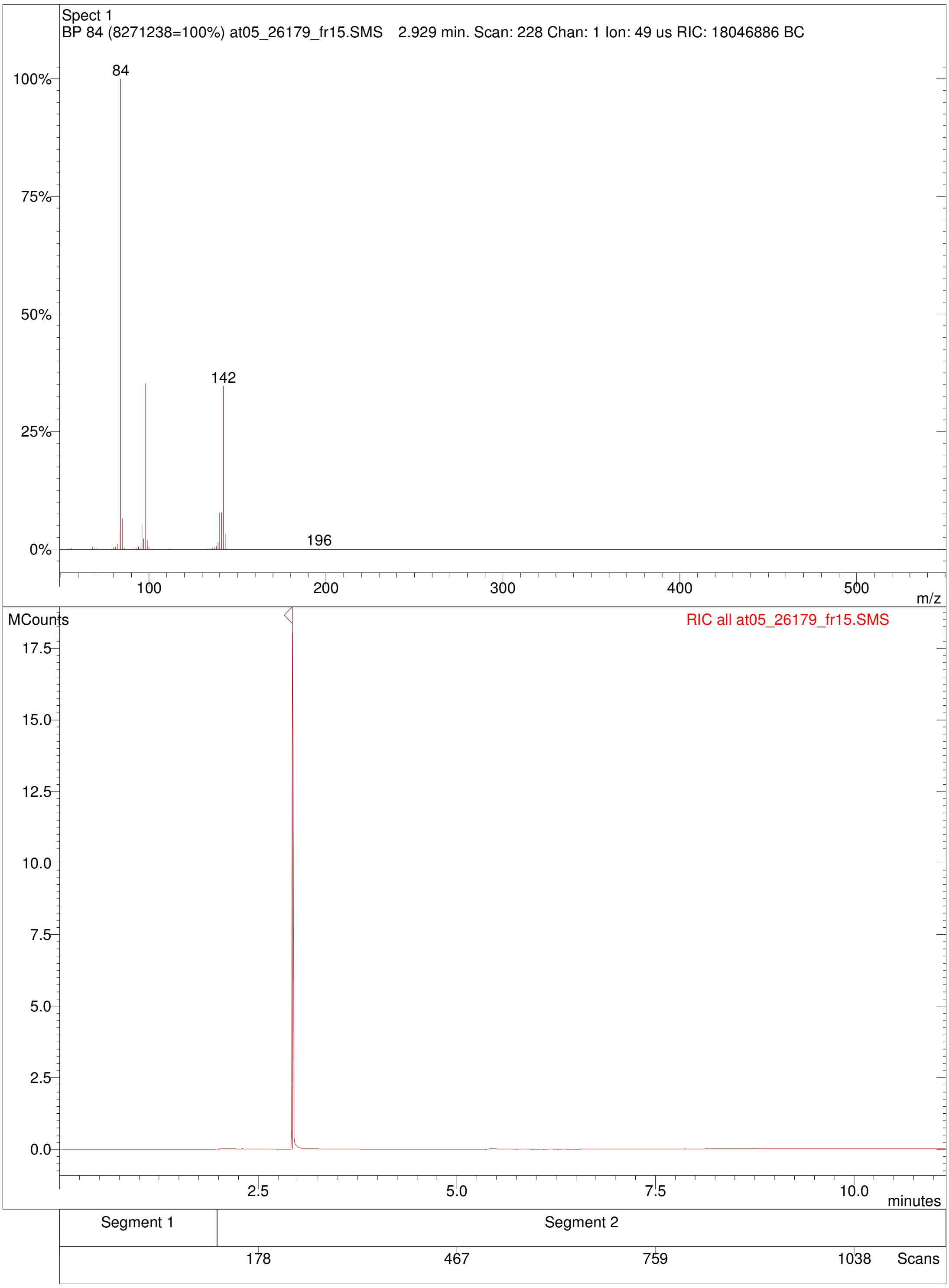

(R)-1-methyl-3-(vinyloxy)piperidine, product 2

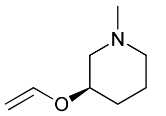

In an oven-dried round bottom Schlenk flask containing ethyl vinyl ether (118 mL, 1.24 mol) was purged with O_2 (g) and thereafter $Pd(O_2CCF_3)_2$ (451.3 mg, 1.97 mmol) and 2,2'-bipyridine (310.0 mg, 1.97 mmol) were added under vigorous stirring. The mixture was stirred at 60°C until all solids were dissolved and a clear bright yellow solution was obtained. To prevent acetal formation,[2] triethylamine (0.69 mL, 4.97 mmol) was added before addition of (*R*)-piperidine alcohol [3] (2.90 g, 24.8 mmol) by cannula. The reaction was refluxed for 72 h or until GC-MS showed no further improvement. The excess ethyl vinyl ether was removed under reduced pressure, the crude material (reddish oil) was washed with 2 M aqueous NaOH (3 x 150 mL) and EtOAc (150 mL). The organic phase was evaporated under reduced pressure and **2** was purified by bulb-to-bulb purification (65°C, 58 mbar) and isolated in 37% yield (1.31 g, 9.2 mmol). Unwashed/unreacted aminoalcohol could also be recovered (90°C, 50 mbar). $[\alpha]_D^{22}$= +18.7° (c=7.9, MeOH); ^{1}H NMR (CD_3OD, 20°C, TMS): δ=6.31 (ddd, *J*=1.3, 6.6, 14.1 Hz, 1H), 4.29 (dd, *J*=1.6, 14.1 Hz, 1H), 3.98 (dd, *J*=1.6,6.6 Hz, 1H), 3.93-3.84 (m, 1H), 2.74 (d, *J*=11.7 Hz, 1H), 2.48 (d, *J*=11.7 Hz, 1H), 2.26 (s, 3H), 2.18-2.05 (m, 2H), 1.90-1.72 (m, 2H), 1.60-1.47 (m, 1H), 1.46-1.34 (m, 1H); ^{13}C NMR (CD_3OD, 20°C, TMS): δ=150.3, 88.3, 74.2, 59.6, 55.3, 46.4, 29.0, 22.8; MS (70 eV): *m/z* (%): 142 (100) [M^+], 98 (79) [$C_6H_{12}N^+$], 58 (21) [$C_3H_8N^+$]; HRMS (ESI): *m/z* calcd for $C_8H_{15}NO$ ($M + H^+$): 142.1232; Found: 142.1236.

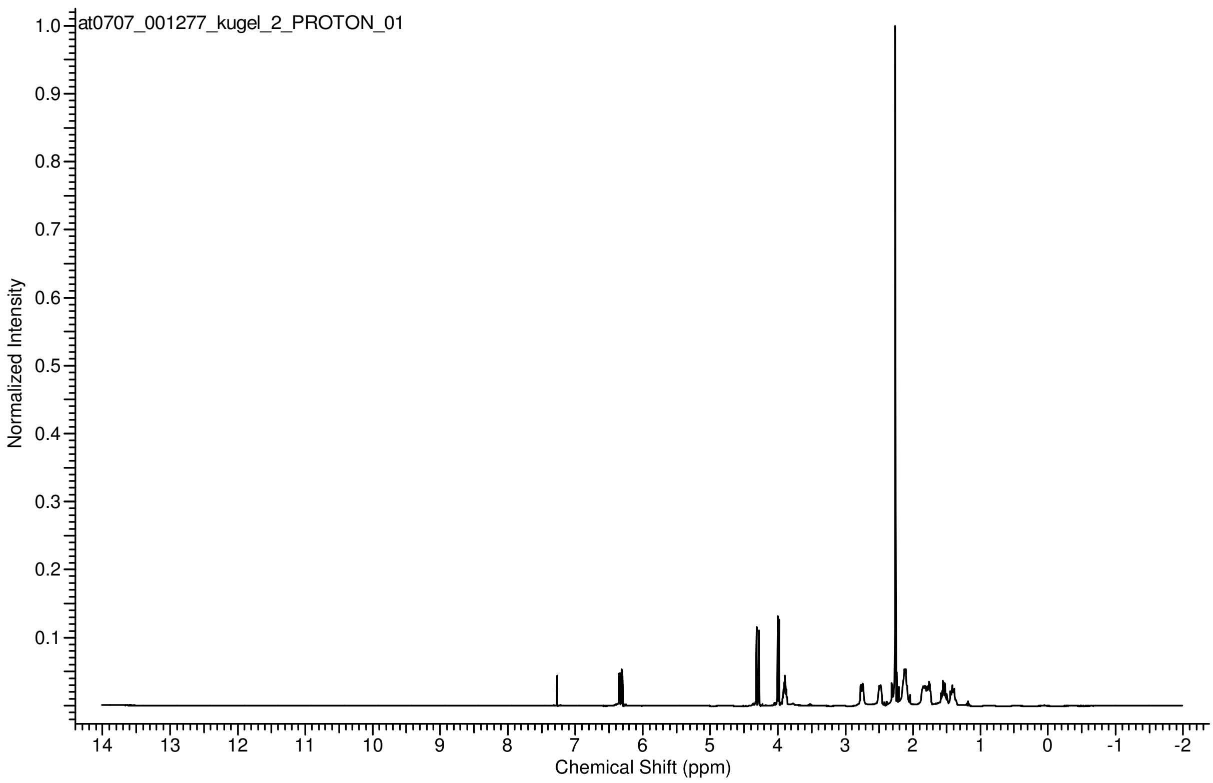
at0707_001277_kugel_2_PROTON_01
Normalized Intensity
Chemical Shift (ppm)

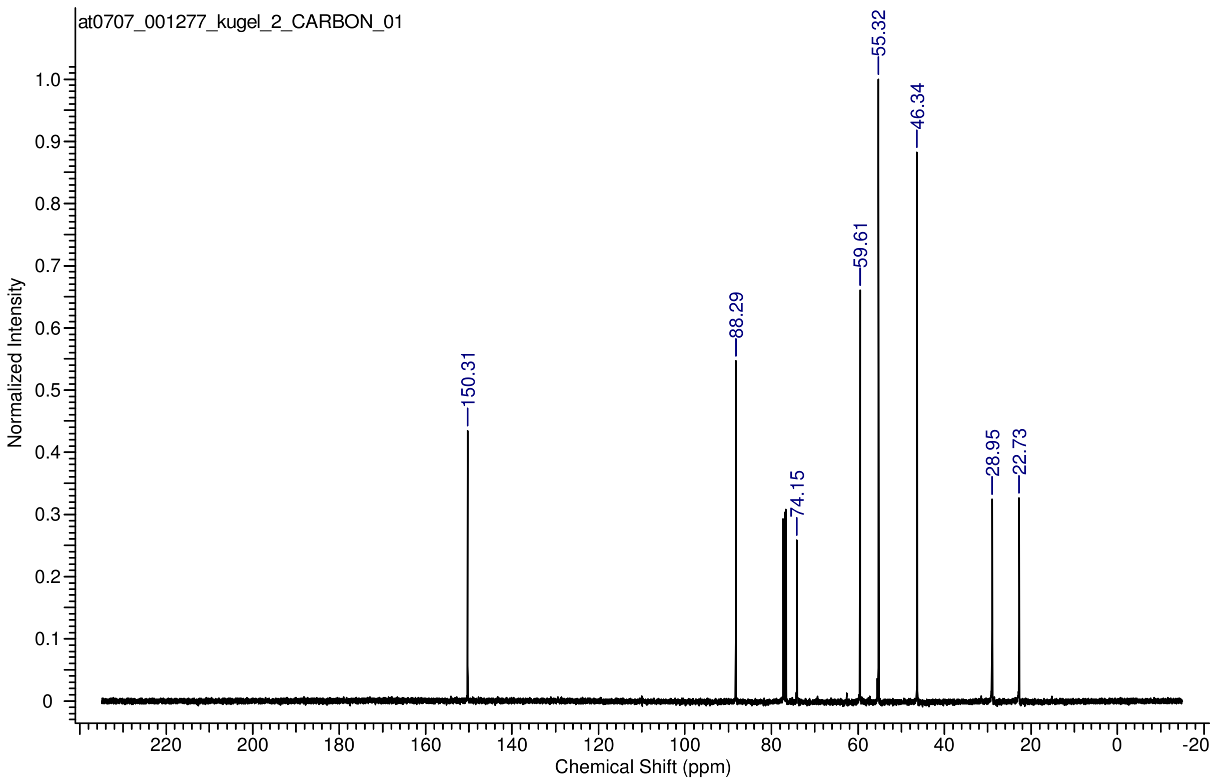
at0707_001277_kugel_2_CARBON_01
150.31
88.29
74.15
59.61
55.32
46.34
28.95
22.73
Normalized Intensity
Chemical Shift (ppm)

Chromatogram Plot

File: m:\... \pek\resterande gc_lc\at0707 012609\at0707_rac_extr04.sms
Sample: at0707_rac_extr04 Operator: Operator
Scan Range: 1 - 1177 Time Range: 0.00 - 11.16 min. Date: 2010-11-24 11:59
Sample Notes: ROUTINE

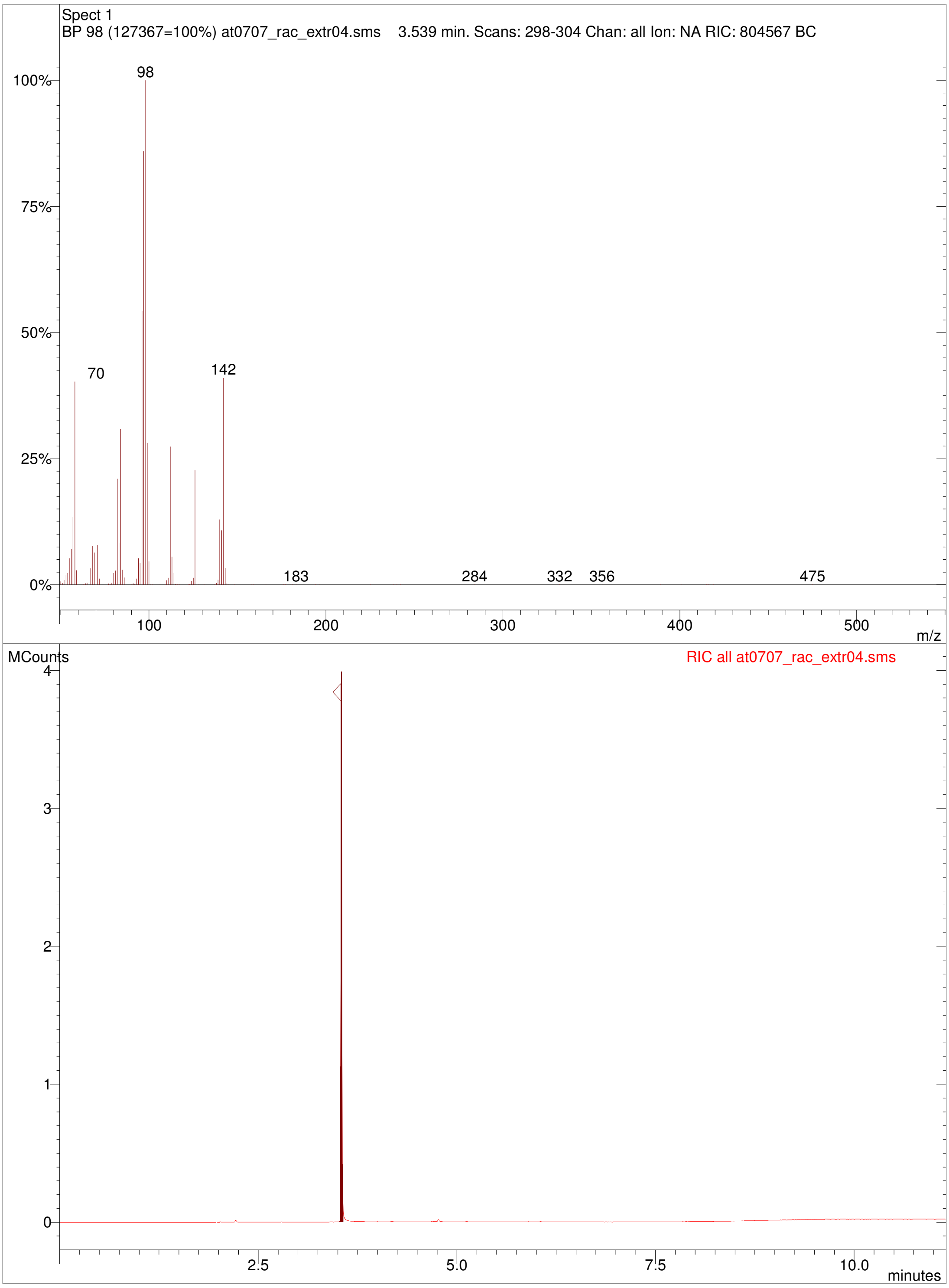

trans-N,N-Dimethyl-2-(vinyloxy)cyclohexanamine, product 3(rac)

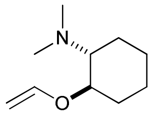

To a 20 mL microwave vial, vinylacetate (10 mL), *trans*-2-(dimethylamino)cyclohexanol (600 mg, 4 mmol) and 6-methyl-2,2'-bipyridine (57.4 mg, 0.34 mmol) were added and the mixture was vigorously stirred. $Pd(O_2CCF_3)_2$ (69.6 mg, 0.21 mmol) was thereafter added and the microwave vial was irradiated for 30 min at 100°C. The crude was concentrated and filtered through a column of aluminium oxide (2 cm Φ, 4 cm high) and eluted with 200 mL of diethyl ether:Et_3N (96:4). The acetylated by-product was separated from the vinylated product by DCVC, isohexane:diethyl ether:Et_3N (100:0:0 to 80:26:4). After chromatography, the product was still contaminated with a small amount of 6-methyl-2,2'-bipyridine and was therefore purified by bulb-to-bulb distillation (70°C, 60 mbar), yielding 206.0 mg pure product **3(rac)**, 29% yield. R_f=0.2 (Isohexane/EtOAc/Et_3N 80:16:4); ^1H NMR (400 MHz, $CDCl_3$, 25°C, TMS): δ=6.35 (dd, J=6.6, 14.1 Hz, 1H), 4.29 (dd, J=1.6, 14.1 Hz, 1H), 3.99 (dd, J=1.6, 6.6 Hz, 1H), 3.77-3.70 (m, 1H), 2.54-2.48 (m, 1H), 2.34 (s, 6H), 2.17-2.10 (m, 1H), 1.92-1.81 (m, 2H), 1.76-1.68 (m, 1H); 1.34-1.13 (m, 4H); ^{13}C NMR (400 MHz, $CDCl_3$, 25°C, TMS): δ=150.8, 87.7, 78.4, 66.0, 41.0, 31.2, 24.9, 24.7, 24.1; MS (70 eV): m/z (%): 169 (34) [M^+], 126 (17) [$C_8H_{16}N^+$], 84 (100) [$C_5H_{10}N^+$]; HRMS (ESI): m/z calcd for $C_{10}H_{19}NO$ ($M + H^+$): 170.1545; Found: 170.1539.

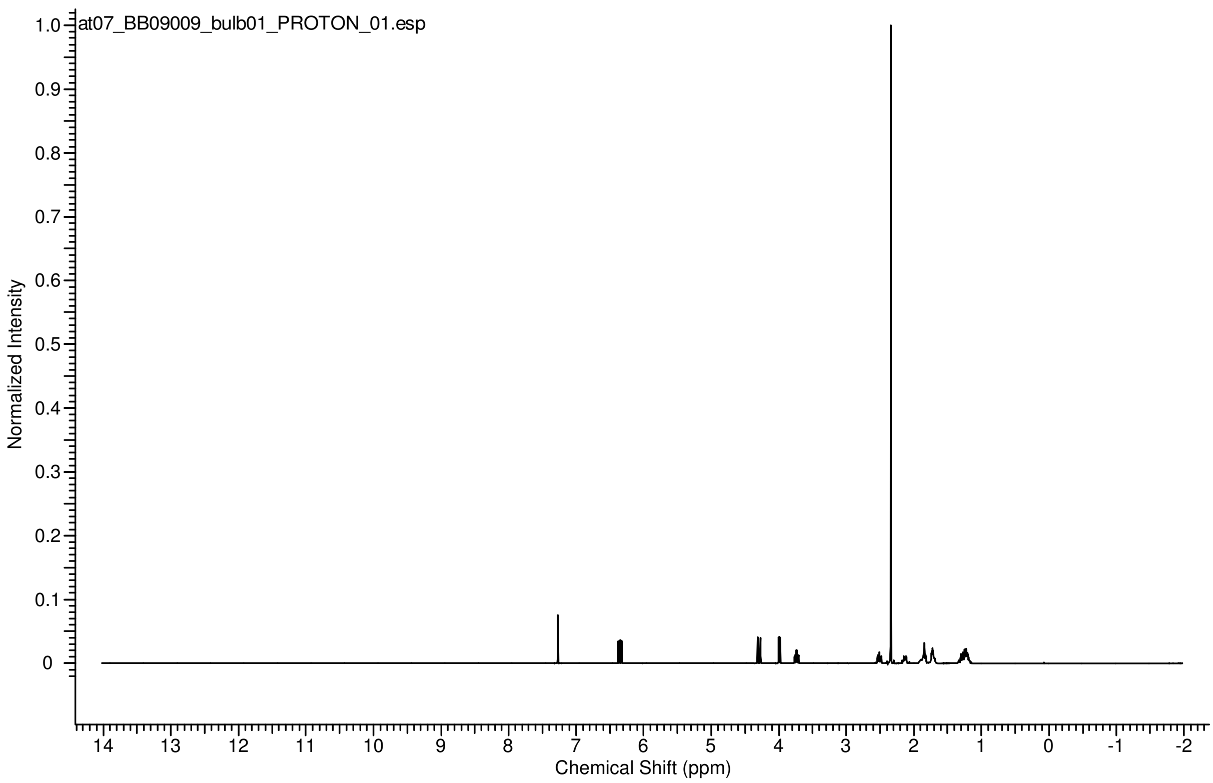
at07_BB09009_bulb01_PROTON_01.esp
Normalized Intensity
Chemical Shift (ppm)

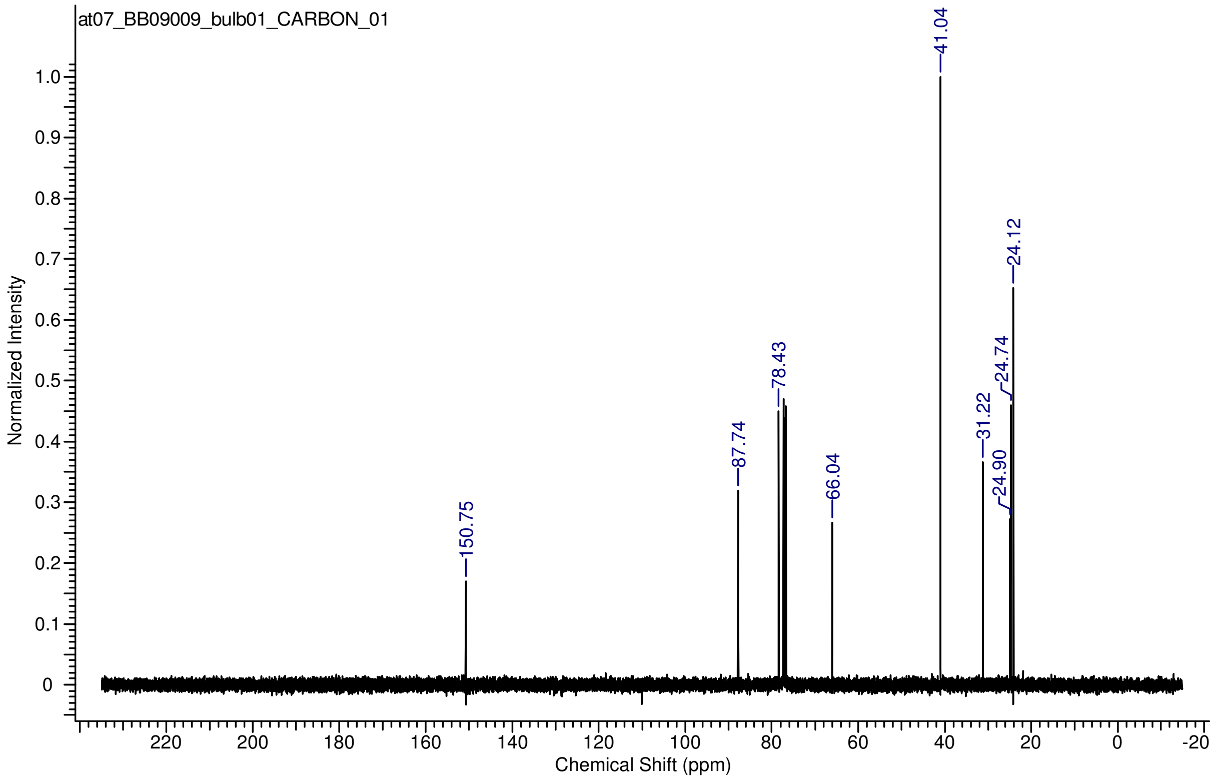
at07_BB09009_bulb01_CARBON_01
150.75
87.74
78.43
66.04
41.04
31.22
24.90
24.74
24.12
Normalized Intensity
Chemical Shift (ppm)

Chromatogram Plot

File: m:\... \at0713c vinylether sm\at07_bb9009_fr09_7-18-2011.sms
Sample: at07_BB9009_fr09 Operator: Org Farm Kemi
Scan Range: 1 - 1099 Time Range: 0.00 - 11.16 min. Date: 2011-07-18 16:09
Sample Notes: Routine

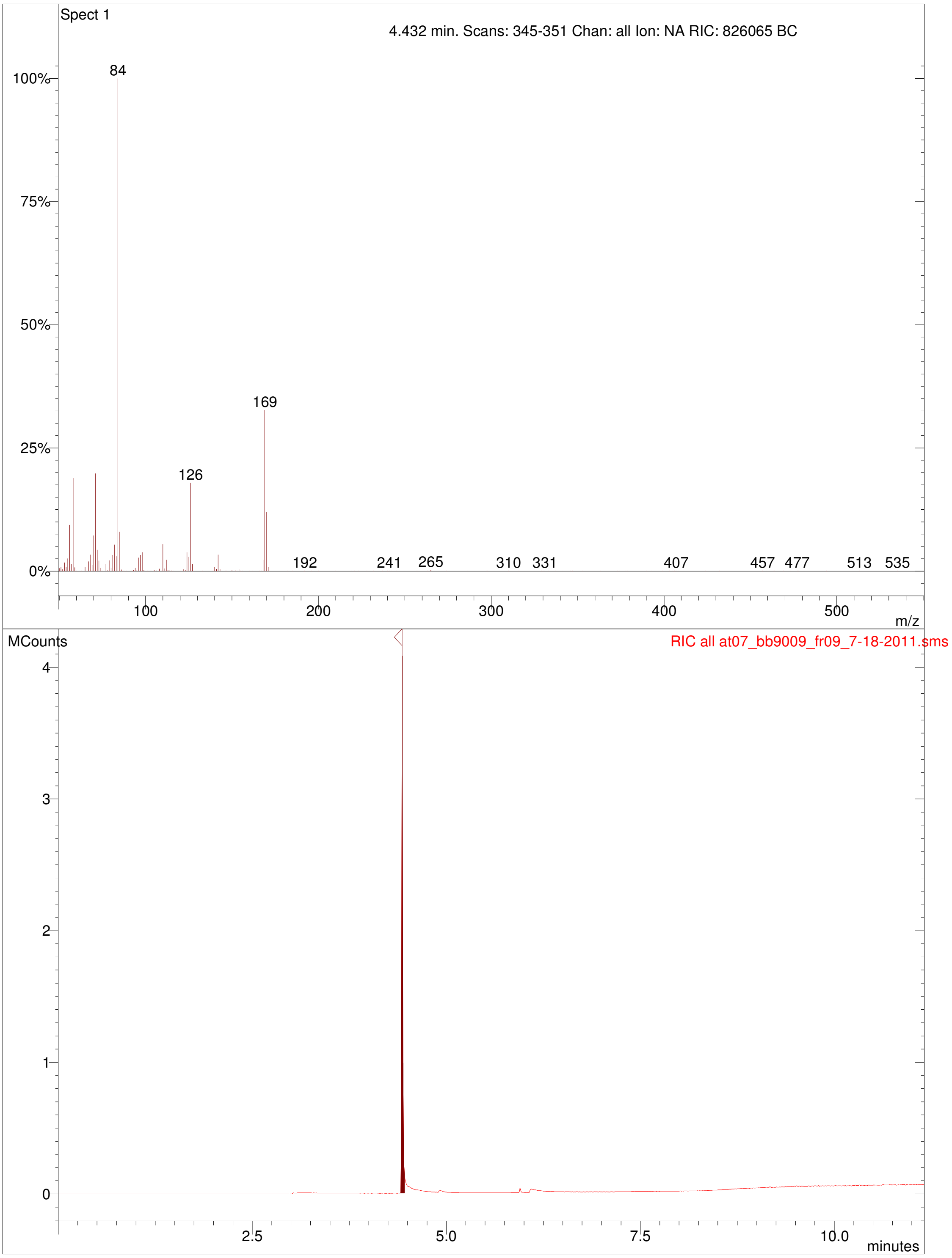

(2S)-2-((1,2-Bis(4-methoxyphenyl)ethoxy)methyl)-1-methylpyrrolidine, product 5a

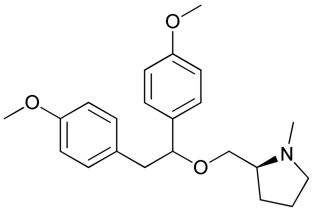

As described in the general procedure for synthesis of diarylated products **5**, **6** and **7(rac)**, but using 4-methoxyphenylboronic acid (**4a**) as the arylating agent. The reaction was stirred for 24 hours and purification by DCVC afforded **5a** in 52% yield (39 mg) as a yellow-brownish oil; 2.9:1 d.r; R_f=0.3 (*i*-hexane/EtOAc/Et_3N 60:36:4); $[\alpha]_D^{22}$= -22.6 (c=11.4 in $CHCl_3$); ^1H NMR ($CDCl_3$, major diastereomer, 20°C, TMS): δ=7.14 (d, *J*=8.6 Hz, 2H), 7.00 (d, *J*=8.6 Hz, 2H), 6.84 (d, *J*=8.7 Hz, 2H), 6.76 (d, *J*=8.7 Hz, 2H), 4.33 (dd, *J*=6.0, 7.4 Hz, 1H), 3.80 (s, 3H), 3.77 (s, 3H), 3.28-3.18 (m, 2H), 2.95 (dd, *J*=7.6, 13.7 Hz, 1H), 2.95-2.90 (m, 1H), 2.78 (dd, *J*=5.8, 13.7 Hz, 1H), 2.41-2.30 (m, 1H), 2.31 (s, 3H), 2.25-2.15 (m, 1H), 1.86-1.75 (m, 1H), 1.69-1.59 (m, 2H), 1.43-1.32 (m, 1H); ^{13}C NMR ($CDCl_3$, major diasteromer, 20°C, TMS): δ=159.0, 157.9, 134.0, 130.7, 130.5, 128.0, 113.6, 113.4, 83.7, 71.8, 65.1, 57.8, 55.2, 43.9, 41.5, 28.6, 22.6; ^1H NMR ($CDCl_3$, minor diastereomer, 20°C, TMS): δ=7.15-7.11 (d, *J*=8.0 Hz, 2H), 7.01 (d, *J*=8.0 Hz, 2H), 6.84 (d, *J*=8.4 Hz, 2H), 6.76 (d, *J*=8.4 Hz, 2H), 4.32 (dd, *J*=6.1, 7.4 Hz, 1H), 3.80 (s, 3H), 3.78 (s, 3H), 3.39 (dd, *J*=5.3, 9.6 Hz, 1H), 3.11-2.98 (m, 3H), 2.83-2.76 (m, 1H), 2.41-2.30 (m, 1H), 2.32 (s, 3H), 2.25-2.15 (m, 1H), 1.86-1.75 (m, 1H), 1.69-1.59 (m, 2H), 1.43-1.32 (m, 1H); ^{13}C NMR ($CDCl_3$, minor diastereomer, 20°C, TMS): δ=158.9, 157.9, 134.1, 130.8, 130.5, 127.9, 113.6, 113.3, 83.8, 71.8, 65.1, 57.6, 55.2, 43.9, 41.5, 28.8, 22.6; MS (70 eV): *m/z* (%): 356 (30) [M^+], 98 (15) [$C_6H_{12}N^+$], 84 (100) [$C_5H_{10}N^+$]; HRMS (ESI): *m/z* calcd for $C_{22}H_{29}NO_3$ (M + H^+): 356.2226; Found: 356.2223.

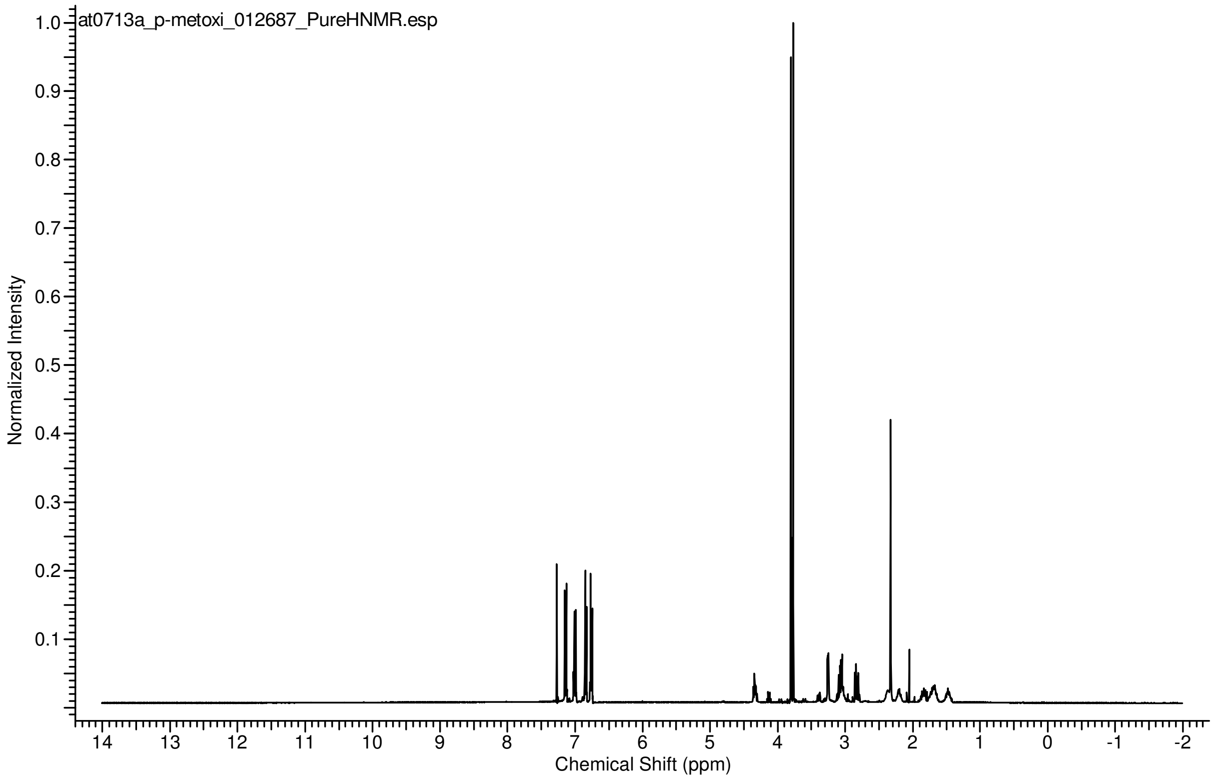
at0713a_p-metoxi_012687_PureHNMR.esp
Normalized Intensity
Chemical Shift (ppm)

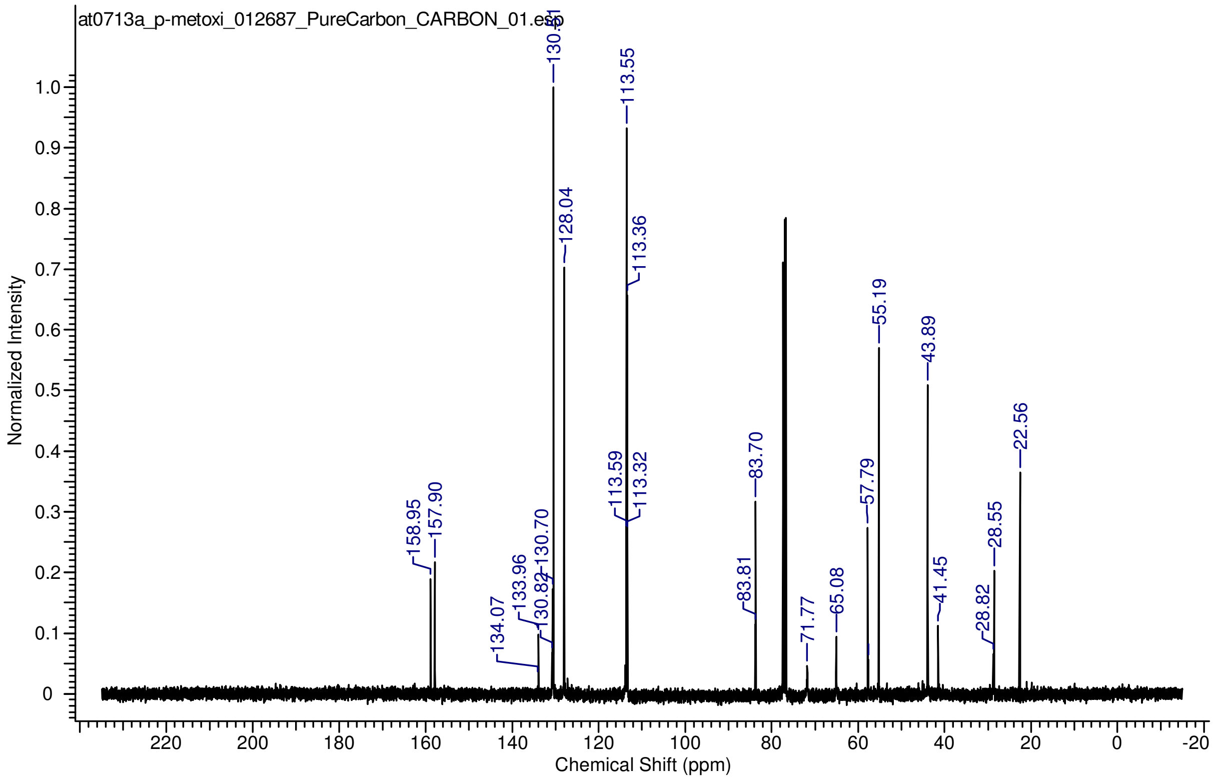
at0713a_p-metoxi_012687_PureCarbon_CARBON_01.esp
Normalized Intensity
Chemical Shift (ppm)
158.95
157.90
134.07
133.96
130.82
130.70
130.51
128.04
113.59
113.55
113.36
113.32
83.81
83.70
71.77
65.08
57.79
55.19
43.89
41.45
28.82
28.55
22.56

Chromatogram Plot

File: m:\... \at053 chiral heck-su\avslutning\gc\at05303_metoxi_fr14.sms
Sample: at0503_Metoxi_fr14 Operator: Operator
Scan Range: 1 - 1172 Time Range: 0.00 - 11.16 min. Date: 07/06/2010 16:47
Sample Notes: ROUTINE

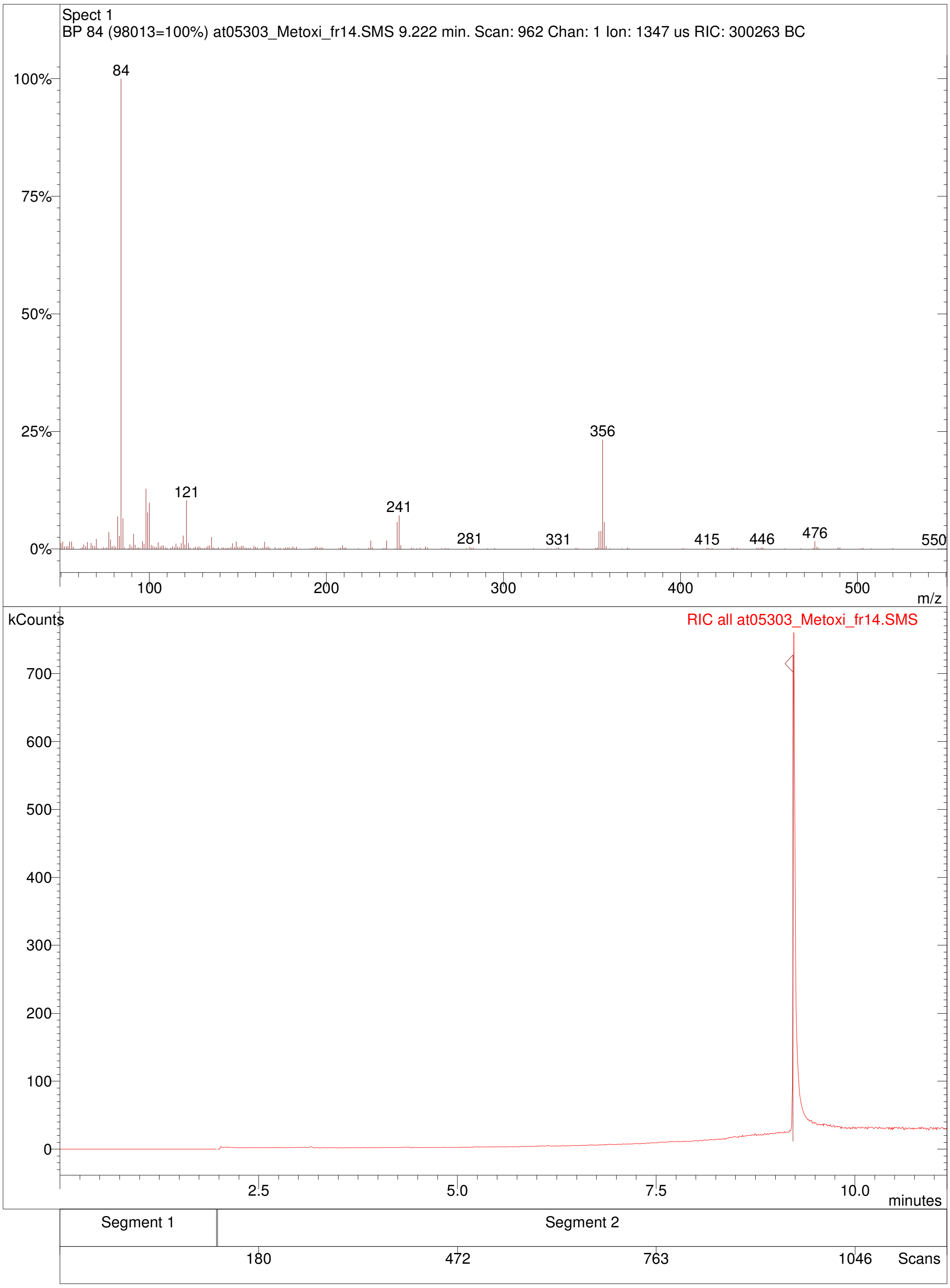

4,4'-(1-((-1-methylpyrrolidin-2-yl)methoxy)ethane-1,2-diyl)diacetylbenzene, product 5b

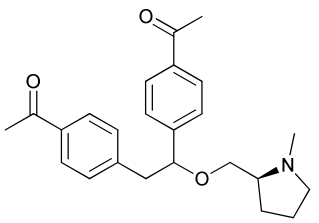

As described in the general procedure for synthesis of diarylated products **5**, **6** and **7(rac)**, but using 4-acetylphenylboronic acid (**4b**) as the arylating agent. The reaction was stirred for 24 hours and purification by DCVC afforded **5b** in 62% yield (50 mg) as a white paste; 4.0:1 d.r; R_f=0.2 (*i*-hexane/EtOAc/Et_3N 40:46:4); $[\alpha]_D^{22}$= -11.1 (c=16.1, Chloroform); ^{1}H NMR ($CDCl_3$, major diastereomer, 20°C, TMS): δ=7.89 (m, 2H), 7.81 (m, 2H), 7.31 (m, 2H), 7.17 (m, 2H), 4.55-4.46 (m, 1H), 3.30-3.20 (m, 2H), 3.16 (dd, *J*=7.4, 13.6 Hz, 1H), 3.05-2.93 (m, 1H), 2.95 (d, *J*=5.9, 13.6 Hz, 1H),), 2.57 (s, 3H), 2.55 (s, 3H), 2.37-2.28 (m, 1H), 2.329 (s, 3H), 2.22-2.11 (m, 1H), 1.87-1.77 (m, 1H), 1.74-1.58 (m, 2H), 1.49-1.38 (m, 1H); ^{13}C NMR ($CDCl_3$, major diastereomer, 20°C, TMS): δ=197.8, 197.7, 147.0, 143.5, 136.6, 136.5, 129.8, 128.4, 128.2, 126.9, 83.0, 72.4, 64.9, 57.8, 44.5, 41.5, 28.5, 22.6; ^{1}H NMR ($CDCl_3$, minor diastereomer, 20°C, TMS): δ=7.89 (m, 2H), 7.81 (m, 2H), 7.31 (m, 2H), 7.17 (m, 2H), 4.55-4.46 (m, 1H), 3.37 (dd, *J*=5.4, 9.5 Hz, 1H), 3.30-3.20 (m, 1H),3.15-3.10 (m, 1H), 3.05-2.93 (m, 2H), 2.96-2.91 (m, 1H), 2.58 (s, 3H), 2.55 (s, 3H), 2.37-2.28 (m, 1H), 2.30 (s, 3H), 2.22-2.11 (m, 1H), 1.87-1.77 (m, 1H), 1.74-1.58 (m, 2H), 1.49-1.38 (m, 1H); ^{13}C NMR ($CDCl_3$, minor diastereomer, 20°C, TMS): δ=197.9, 197.7, 147.1, 143.6, 136.7, 136.5, 129.9, 128.5, 128.1, 126.8, 83.1, 72.6, 65.0, 57.8, 44.5, 41.5, 28.8, 22.7; MS (70 eV): *m/z* (%): 380 (7) [M^+], 98 (10) [$C_6H_{12}N^+$], 84 (100) [$C_5H_{10}N^+$]; HRMS (ESI): *m/z* calcd for $C_{24}H_{29}NO_3$ ($M + H^+$): 380.2226; Found: 380.2230.

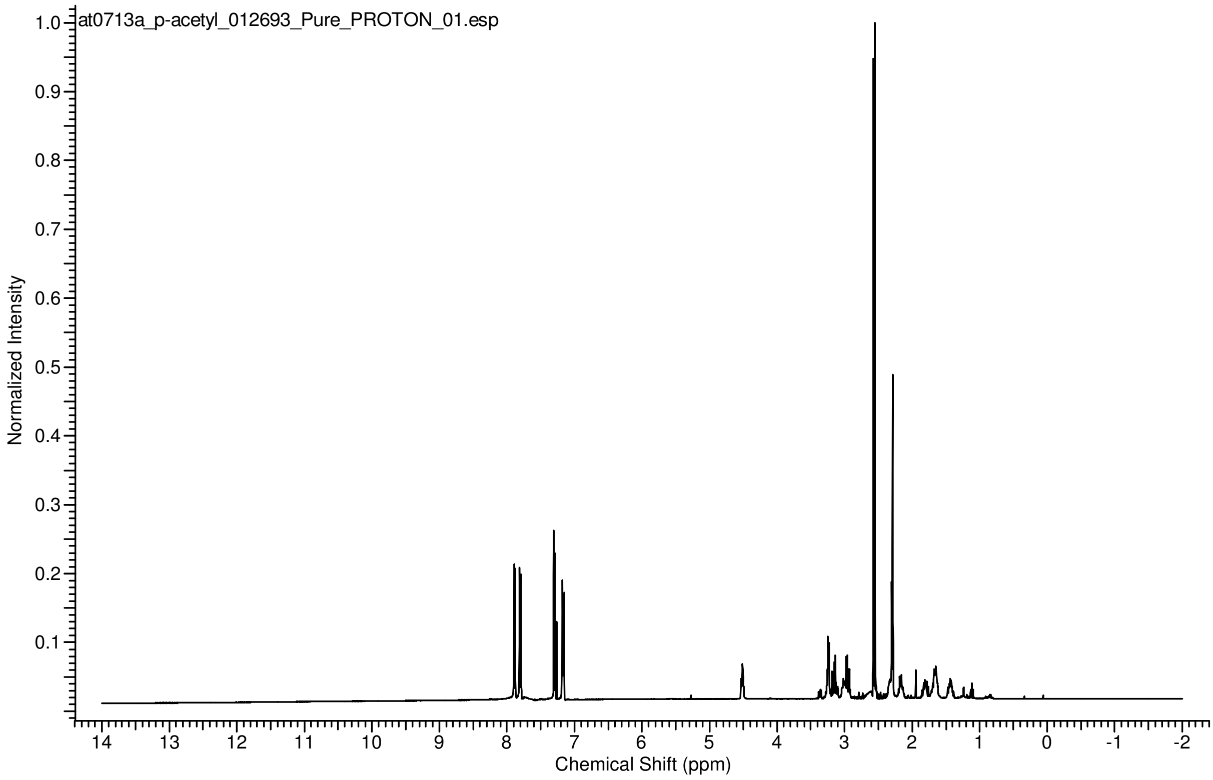
at0713a_p-acetyl_012693_Pure_PROTON_01.esp
Normalized Intensity
Chemical Shift (ppm)

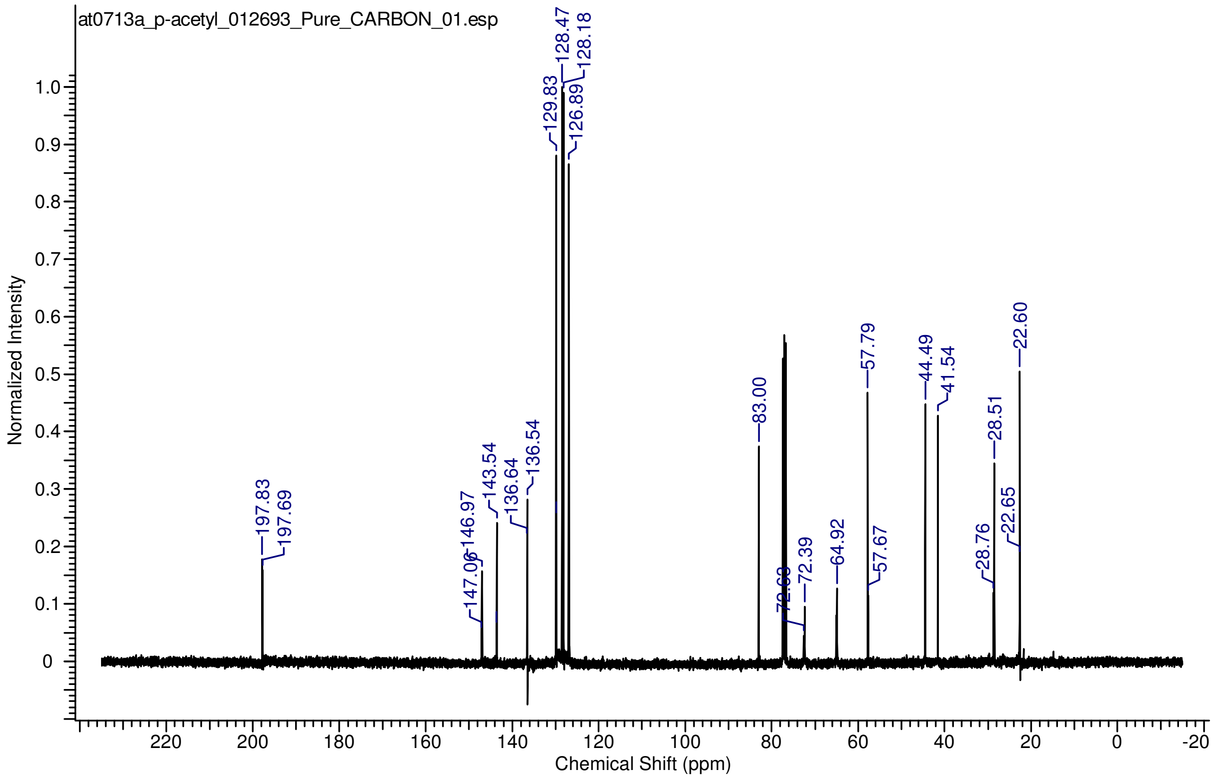
at0713a_p-acetyl_012693_Pure_CARBON_01.esp
Normalized Intensity
Chemical Shift (ppm)
197.83
197.69
147.06
146.97
143.54
136.64
136.54
129.83
128.47
128.18
126.89
83.00
72.39
64.92
57.79
57.67
44.49
41.54
28.76
28.51
22.65
22.60

Chromatogram Plot

File: f:\gcms & lc\at05302_001248_exor.sms
Sample: at05302_001248_EXOr
Operator: Operator
Scan Range: 1 - 1195 Time Range: 0.00 - 11.98 min.
Date: 02/09/2010 13:51
Sample Notes: ROUTINE

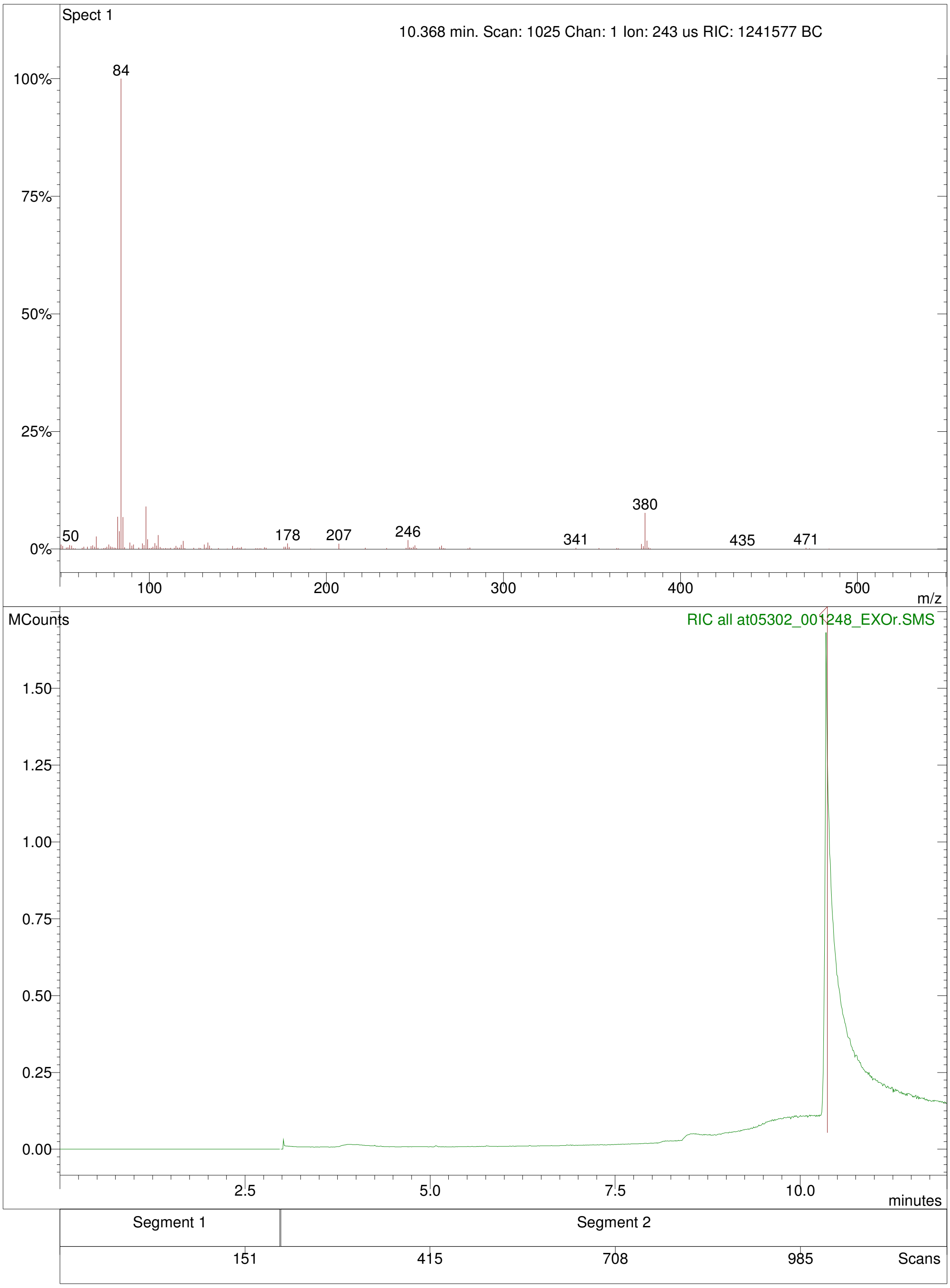

(2S)-2-((1,2-Diphenylethoxy)methyl)-1-methylpyrrolidine, product 5c

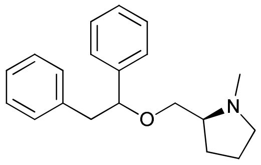

As described in the general procedure for synthesis of diarylated products **5**, **6** and **7(rac)**, but using phenylboronic acid (**4c**) as the arylating agent. The reaction was stirred for 24 hours and purification by DCVC afforded **5c** in 78% yield (49 mg) as a dark-yellowish oil; 2.5:1 d.r.; R_f=0.3 (Isohexane/EtOAc/Et_3N 70:24:6); $[\alpha]_D^{22}$= -38.3° (c=8.7 in $CHCl_3$); ^{1}H NMR ($CDCl_3$, major isomer, 25°C, TMS): δ=7.34-7.08 (m, 10H), 4.44 (dd, *J*=5.7, 7.6 Hz, 1H), 3.31-3.22 (m, 2H), 3.13 (dd, *J*=7.6, 13.6 Hz, 1H), 3.07-2.96 (m, 1H), 2.90 (dd, *J*=5.8, 13.6 Hz, 1H), 2.41-2.28 (m, 1H), 2.30 (s, 3H), 2.24-2.13 (m, 1H), 1.88-1.53 (m, 3H), 1.52-1.40 (m , 1H); ^{13}C NMR ($CDCl_3$, major isomer, 25°C, TMS): δ=141.9, 138.6, 129.6, 128.2, 127.9, 127.46, 126.7 , 126.0, 84.0, 72.0, 65.0, 57.8, 44.9, 41.4, 28.5, 22.5; ^{1}H NMR ($CDCl_3$, minor isomer, 25°C, TMS): δ=7.34-7.08 (m, 10H), 4.42 (dd, *J*=5.3, 7.6 Hz, 1H), 3.42 (dd, *J*=5.3, 9.5 Hz, 1H), 3.31-3.22 (m, 1H), 3.11 (dd, *J*=5.9, 9.5 Hz, 1H), 3.07-2.96 (m, 1H), 2.88 (dd, *J*=5.8, 13.7 Hz, 1H), 2.41-2.28 (m, 1H), 2.32 (s, 3H), 2.24-2.13 (m, 1H), 1.88-1.53 (m, 3H), 1.52-1.40 (m, 1H); ^{13}C NMR ($CDCl_3$, minor isomer, 25°C, TMS): δ=142.0, 138.7, 129.61, 128.24, 128.0, 127.48, 126.8, 126.0, 84.1, 72.0, 65.0, 57.6, 44.9, 41.4, 28.8, 22.6; MS (70 eV): *m/z* (%): 296 (5) [M^+], 98 (10) [$C_6H_{12}N^+$], 84 (100) [$C_5H_{10}N^+$];HRMS (ESI): *m/z* calcd for $C_{20}H_{25}NO$ (M + H^+): 296.2014; Found: 296.2012.

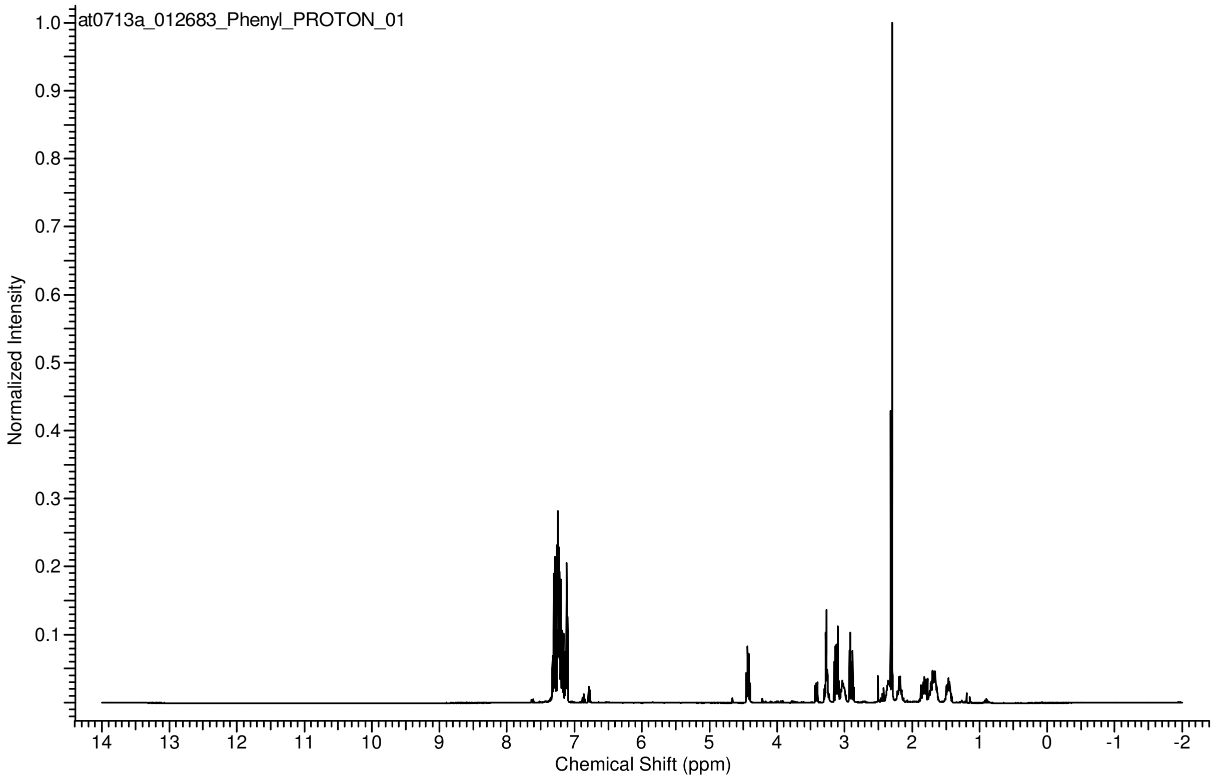
at0713a_012683_Phenyl_PROTON_01
Normalized Intensity
1.0
0.9
0.8
0.7
0.6
0.5
0.4
0.3
0.2
0.1
14 13 12 11 10 9 8 7 6 5 4 3 2 1 0 -1 -2
Chemical Shift (ppm)

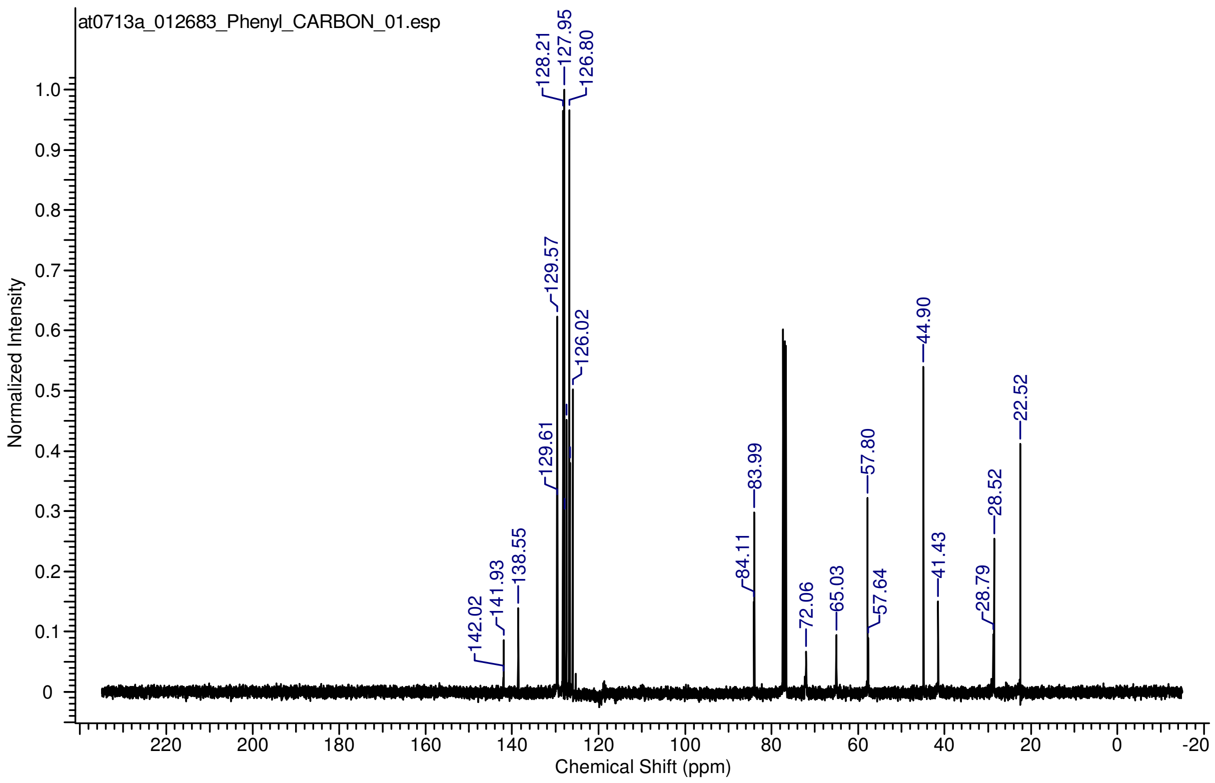
at0713a_012683_Phenyl_CARBON_01.esp
Normalized Intensity
1.0
0.9
0.8
0.7
0.6
0.5
0.4
0.3
0.2
0.1
0
142.02
141.93
138.55
129.61
129.57
128.21
127.95
126.80
126.02
84.11
83.99
72.06
65.03
57.80
57.64
44.90
41.43
28.79
28.52
22.52
220 200 180 160 140 120 100 80 60 40 20 0 -20
Chemical Shift (ppm)

Chromatogram Plot

File: m:\... \pek\resterande gc_lc\at0713a_012683\at0713a_012683_ph.sms
Sample: at0713a_012683_ph Operator: Operator
Scan Range: 1 - 1158 Time Range: 0.00 - 11.16 min. Date: 2011-02-16 15:56
Sample Notes: ROUTINE

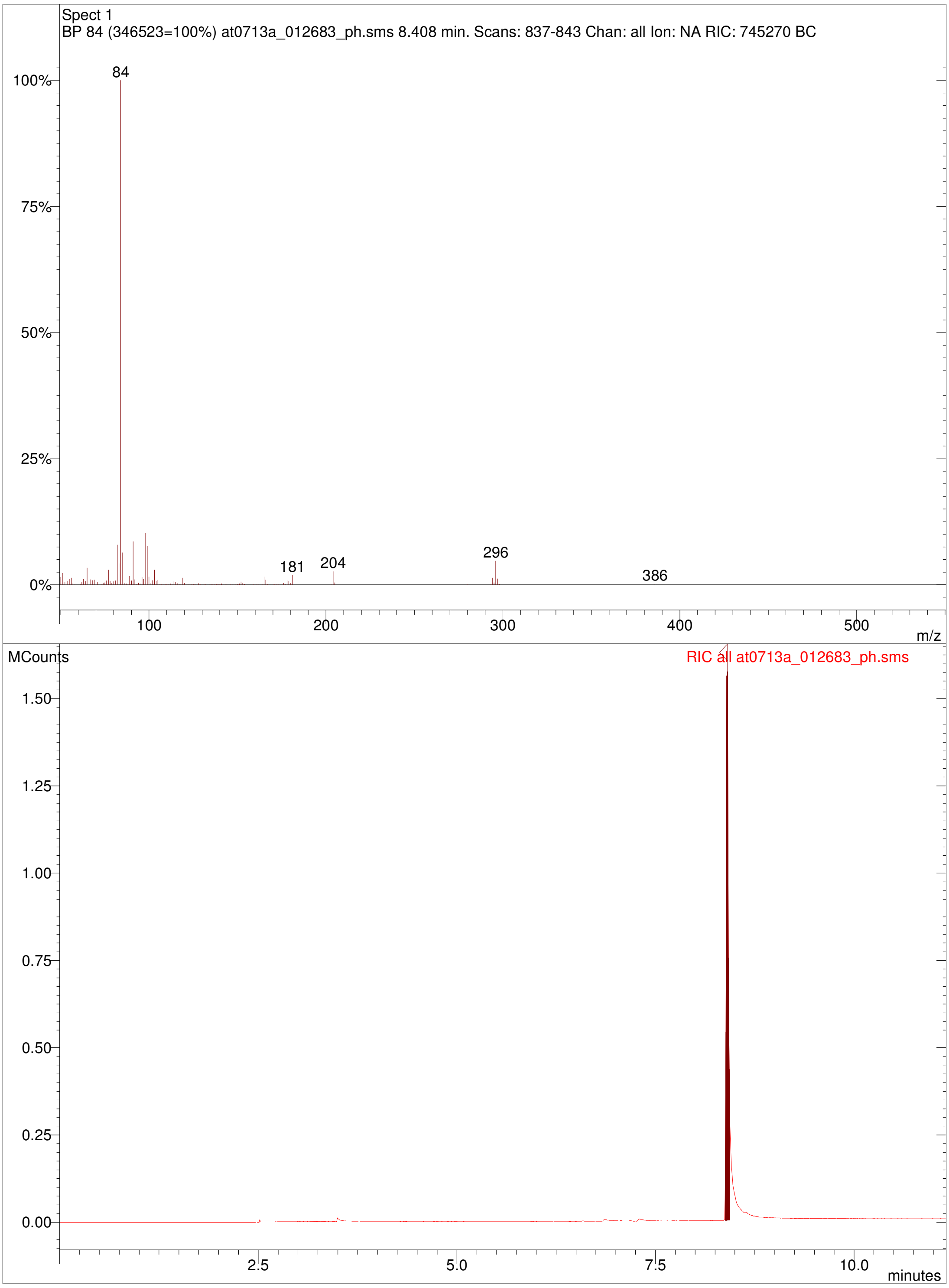

(2S)-2-((1,2-Bis(2-methoxyphenyl)ethoxy)methyl)-1-methylpyrrolidine, product 5d

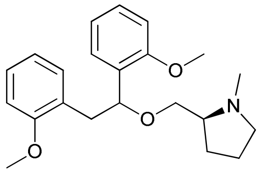

As described in the general procedure for synthesis of diarylated products **5**, **6** and **7(rac)**, but using 2-methoxyphenylboronic acid (**4d**) as the arylating agent. The reaction was stirred for 24 hours and purification by DCVC afforded **5d** in 24% yield (18 mg) as a pale-yellow oil; 1.4:1 d.r.; R_f=0.2 (*i*-hexane/EtOAc/Et_3N 70:26:4); $[\alpha]_D^{22}$= -16.6° (c =4.2 in $CHCl_3$); ^{1}H NMR ($CDCl_3$, major diastereomer, 20°C, TMS): δ=7.43 (dd, *J*=1.8, 7.6 Hz, 1H), 7.19 (ddd, *J*=0.7, 1.8, 7.3 Hz, 1H), 7.17-7.11 (m, 1H), 7.09 (dd, *J*=1.8, 7.9 Hz, 1H), 7.00-6.94 (m, 1H), 6.84-6.77 (m, 3H), 5.03 (dd, *J*=5.1, 7.8 Hz, 1H), 3.76 (s, 3H), 3.66 (s, 3H), 3.45 (dd, *J*=5.3, 9.5 Hz, 1H), 3.15-2.89 (m, 4H), 2.35-2.27 (m, 1H), 2.32 (s, 3H), 2.20-2.11 (m, 1H), 1.89-1.57 (m, 3H), 1.49-1.38 (m, 1H); ^{13}C NMR ($CDCl_3$, major diasteromer, 20°C, TMS): δ=157.8, 157.1, 131.2, 131.2, 127.8, 127.6, 127.0, 126.8, 120.6, 119.9, 110.2, 110.0, 75.7, 72.3, 65.0, 57.8, 55.3 41.4, 37.6, 28.6, 22.6; ^{1}H NMR ($CDCl_3$, minor diastereomer, 20°C, TMS): δ=7.42 (dd, *J*=1.8, 7.5 Hz, 1H), 7.21 (ddd, *J*=0.7, 1.8, 7.3 Hz, 1H), 7.17-7.11 (m, 1H), 7.06 (dd, *J*=1.7, 7.6 Hz, 1H), 7.00-6.94 (m, 1H), 6.84-6.77 (m, 3H), 5.06 (dd, *J*=5.3, 7.8 Hz, 1H), 3.77 (s, 3H), 3.68 (s, 3H), 3.32-3.24 (m, 1H), 3.28 (dd, *J*=4.9, 5.5 Hz, 1H), 3.15-2.89 (m, 3H), 2.35-2.27 (m, 1H), 2.30 (s, 3H), 2.20-2.11 (m, 1H), 1.89-1.57 (m, 3H), 1.49-1.38 (m, 1H);^{13}C NMR ($CDCl_3$, minor diastereomer, 20°C, TMS): δ=157.9, 157.0, 131.2, 131.1, 127.8, 127.5, 127.0, 126.8, 120.5, 119.9, 110.2, 110.0, 75.4, 72.7, 65.0, 57.7, 55.3, 41.4, 37.5, 28.9, 22.5; HRMS (ESI): *m/z* calcd for $C_{22}H_{29}NO$ (*M* + H^+): 356.2226; Found: 356.2229.

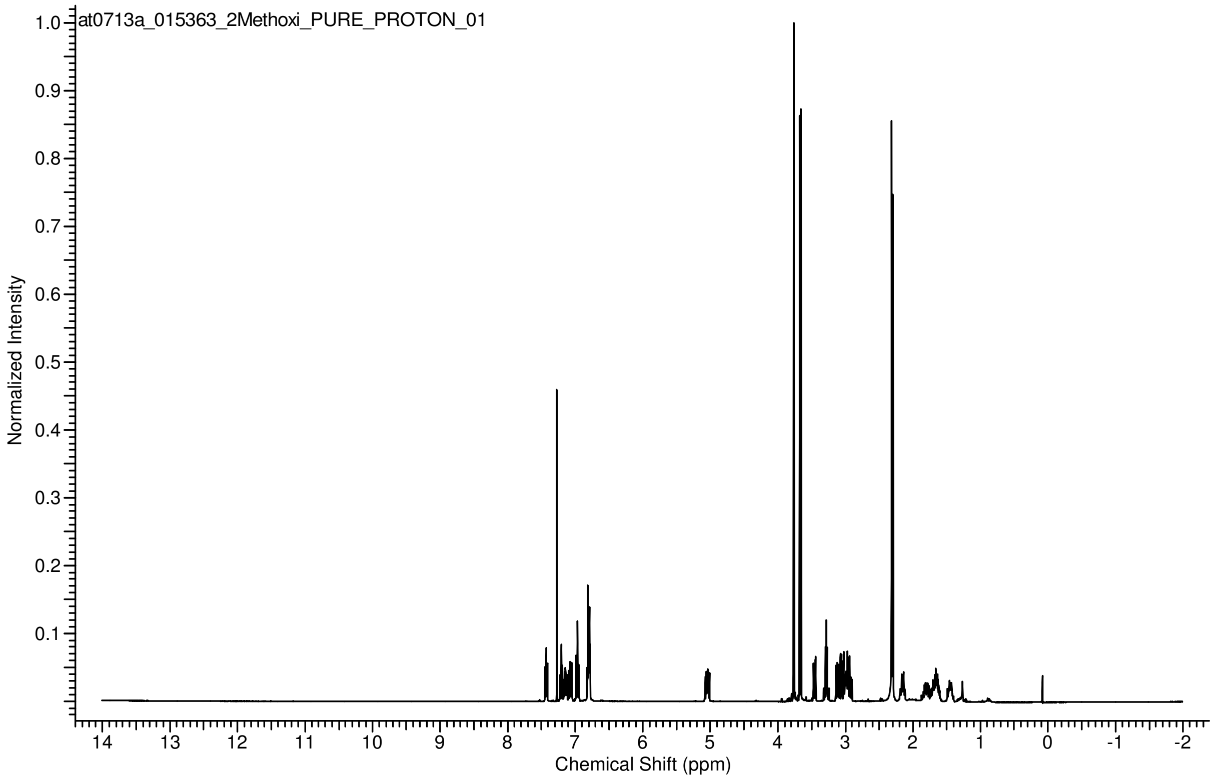
at0713a_015363_2Methoxi_PURE_PROTON_01
Normalized Intensity
Chemical Shift (ppm)

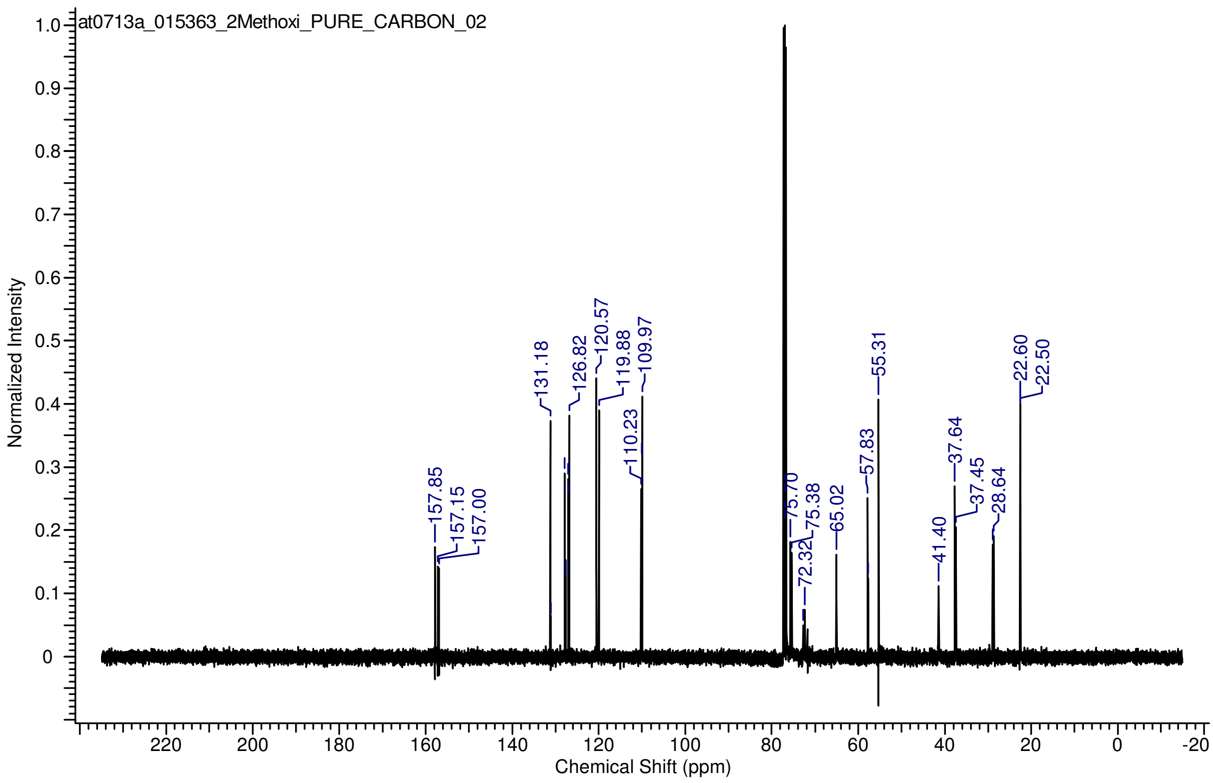
at0713a_015363_2Methoxi_PURE_CARBON_02
Normalized Intensity
Chemical Shift (ppm)
157.85
157.15
157.00
131.18
126.82
120.57
119.88
110.23
109.97
75.70
75.38
72.32
65.02
57.83
55.31
41.40
37.64
37.45
28.64
22.60
22.50

LC-MS Analysis Report

General Information

Sample ID:	at0713a_015363_PREP
Date & Time:	7/12/2011 2:27:01 PM
Data File:	D:\BMC\Users\alejandro\results\at07\11 v28\at0713a_015363_PREP_10774.D
Data Processing:	Mass spectrum report with integrated +/-TIC, UV chromatograms displayed

Chromatogram

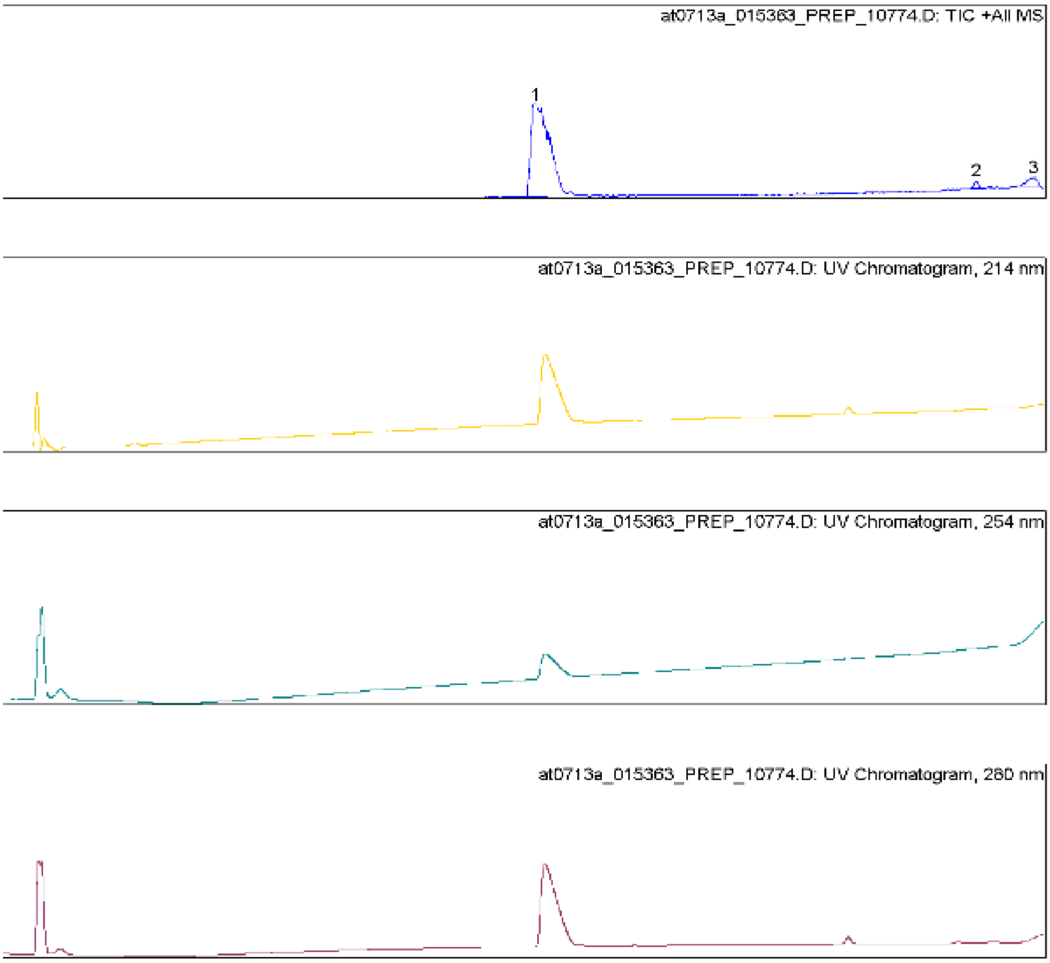

Cmpd 1, 2.82 min

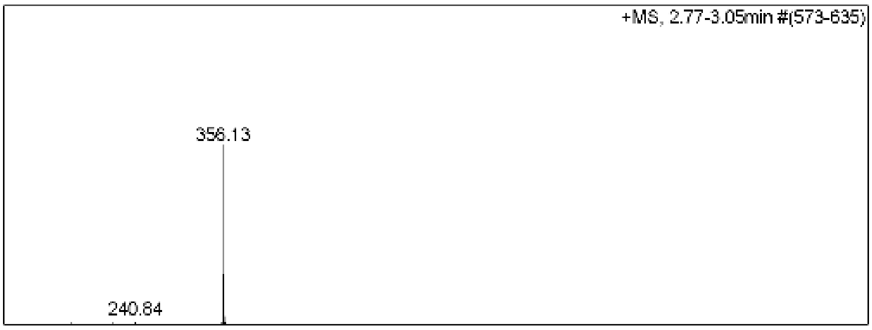

#	m/z
1	356.13
2	357.10
3	358.10

(2S)-2-((1,2-Di-p-tolylethoxy)methyl)-1-methylpyrrolidine, product 5e

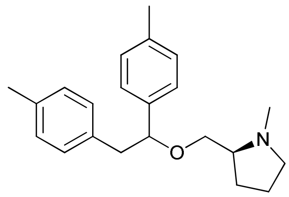

As described in the general procedure for synthesis of diarylated products **5**, **6** and **7(rac)**, but using *p*-tolylboronic acid (**4e)** as the arylating agent. The reaction was stirred for 24 hours and purification by DCVC afforded **5e** in 56% yield (39 mg) as a pale yellow oil; 2.3:1 d.r.; R_f=0.3 (*i*-hexane/EtOAc/Et_3N 70:26:4); $[\alpha]_D^{22}$= -29.1° (c=10.2 in $CHCl_3$); ^1H NMR ($CDCl_3$, major isomer, 25°C, TMS): δ=7.14-7.12 (m, 4H), 7.04-7.01 (m, 4H), 4.38 (dd, *J*=5.7, 7.8 Hz, 1H), 3.27-3.21 (m, 2H; overlapping signals), 3.08 (dd, *J*=7.5, 13.6 Hz, 1H), 3.05-2.98(m, 1H), 2.85 (dd, *J*=5.7, 13.76 Hz, 1H), 2.44-2.29 (m, 1H), 2.35 (s, 3H), 2.31 (s, 3H), 2.30 (s, 3H), 2.25-2.14 (m, 1H), 1.91-1.59 (m, 3H), 1.55-1.41 (m, 1H); ^{13}C NMR ($CDCl_3$, major isomer, 25°C, TMS): δ=139.0, 137.0, 135.6, 135.4, 129.4, 128.9. 128.6, 126.8, 83.9, 71.9, 65.1, 57.8, 44.4, 41.4, 28.5, 22.5, 21.1; ^1H NMR ($CDCl_3$, minor isomer, 25°C, TMS): δ=7.14-7.12 (m, 2H), 7.04-7.01 (m, 4H), 4.36 (dd, *J*=5.4, 7.8 Hz, 1H), 3.41 (dd, *J*=5.4, 9.6 Hz, 1H), 3.30-3.24 (m, 1H; overlapping signals), 3.13-3.07 (m, 1H; overlapping signals), 3.05-2.98(m, 1H), 2.83 (dd, *J*=5.4, 13.8 Hz, 1H), 2.44-2.29 (m, 1H), 2.35 (s, 3H), 2.33 (s, 3H), 2.30 (s, 3H), 2.25-2.14 (m, 1H), 1.91-1.59 (m, 3H), 1.55-1.41 (m, 1H); ^{13}C NMR ($CDCl_3$, minor isomer, 25°C, TMS): δ=139.1, 137.0, 135.7, 135.4, 129.5, 128.9, 128.6, 126.7, 84.0, 71.9, 65.0, 57.6, 44.4, 41.4, 28.8, 25.6, 21.0; MS (70 eV): *m/z* (%): 324 (15) [M^+], 98 (13) [$C_6H_{12}N^+$], 84 (100) [$C_5H_{10}N^+$];HRMS (ESI): *m/z* calcd for $C_{22}H_{29}NO$ (*M* + *1*) : 324.2327; Found: 324.2329.

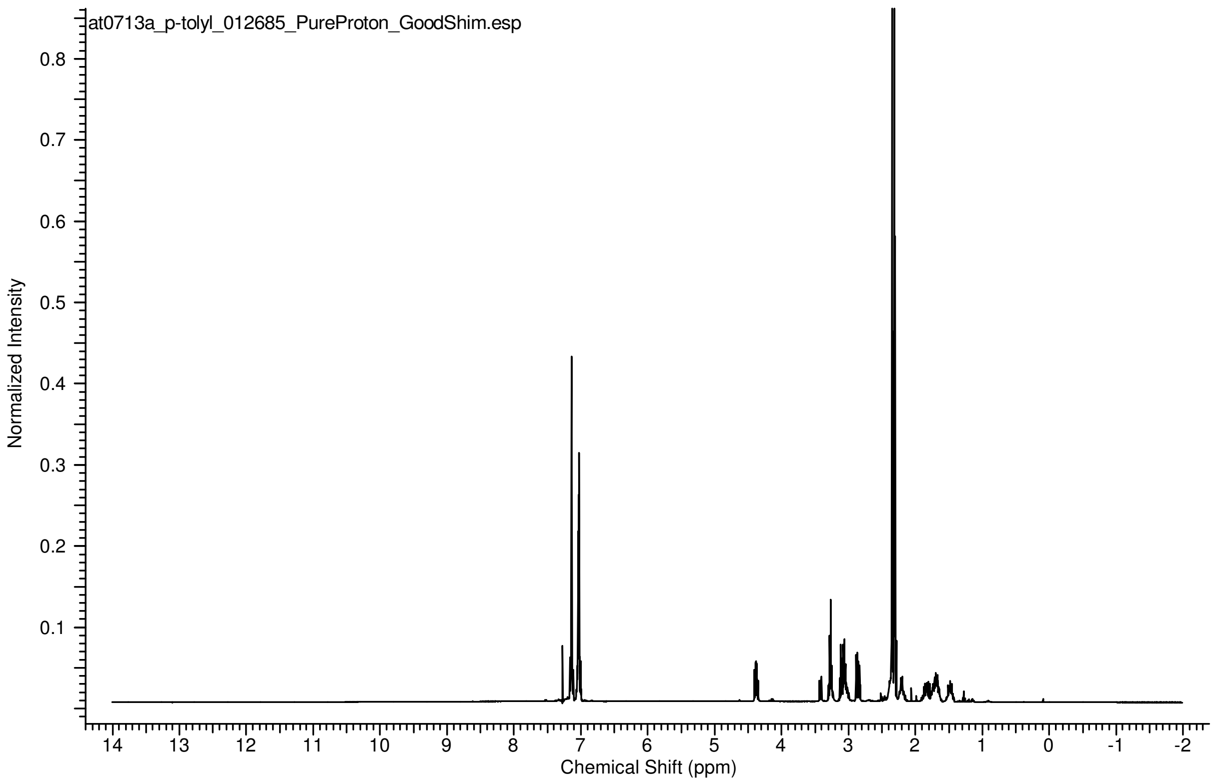
at0713a_p-tolyl_012685_PureProton_GoodShim.esp
Normalized Intensity
Chemical Shift (ppm)

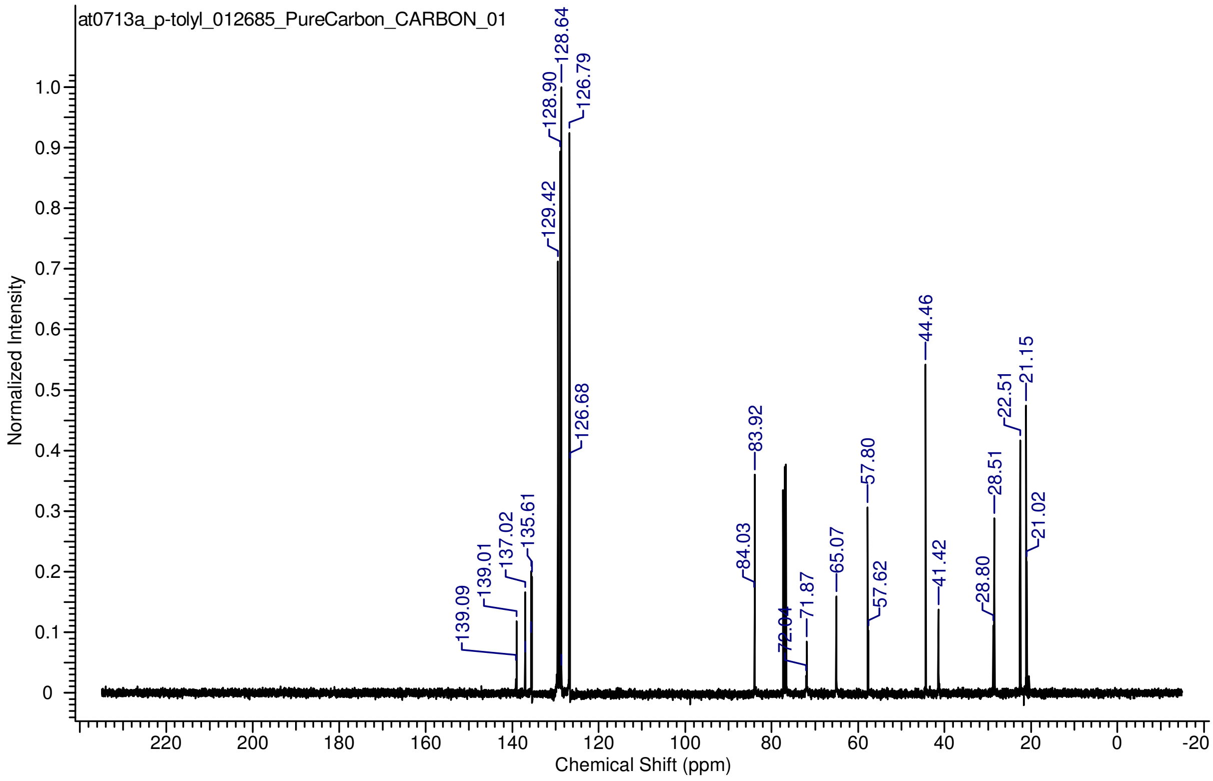
at0713a_p-tolyl_012685_PureCarbon_CARBON_01
Normalized Intensity
Chemical Shift (ppm)
139.09
139.01
137.02
135.61
129.42
128.90
128.64
126.79
126.68
84.03
83.92
72.04
71.87
65.07
57.80
57.62
44.46
41.42
28.80
28.51
22.51
21.15
21.02

Chromatogram Plot

File: m:\... \at053 chiral heck-su\avslutning\gc\is_000652_pch3.sms
Sample: IS_000652_pCH3 Operator: Operator
Scan Range: 1 - 1190 Time Range: 0.00 - 11.16 min. Date: 04/22/2010 21:40
Sample Notes: ROUTINE

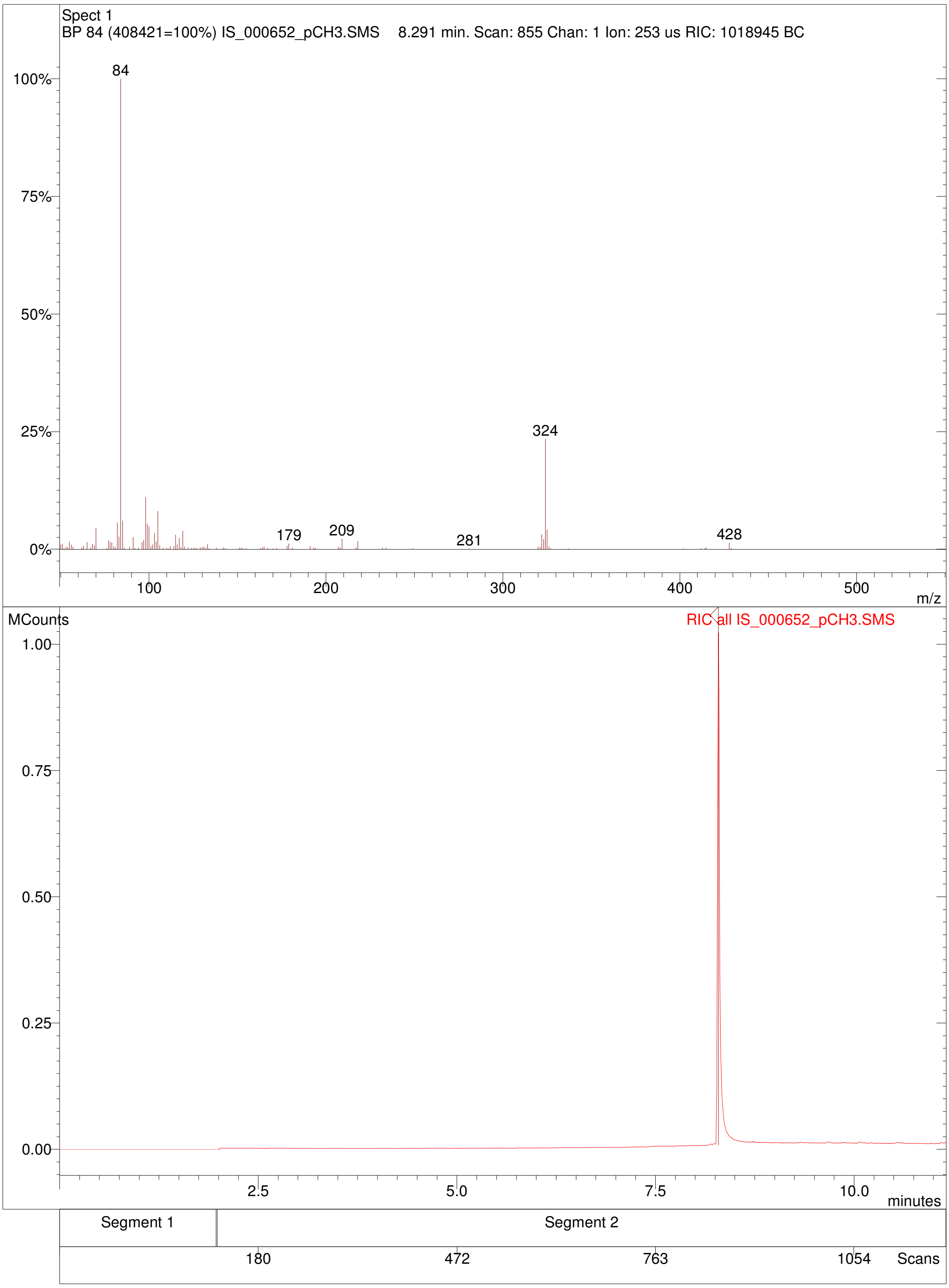

(2S)-2-((1,2-Di-m-tolylethoxy)methyl)-1-methylpyrrolidine, product 5f

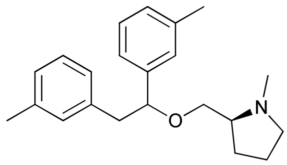

As described in the general procedure for synthesis of diarylated products **5**, **6** and **7(rac)**, but using *m*-tolylboronic acid (**4f**) as the arylating agent. The reaction was stirred for 24 hours and purification by DCVC afforded **5f** in 63% yield (43 mg) as a pale-yellow oil; 2.0:1 d.r.; R_f=0.3 (*i*-hexane/EtOAc/Et_3N 70:26:4); $[\alpha]^{22}_{D}$= -33.9° (c=12.2 in $CHCl_3$); ^1H NMR ($CDCl_3$, major isomer, 25°C, TMS): δ=7.22 (t, *J*=7.3 Hz, 1H), 7.17-7.06 (m, 4H), 7.02-6.98 (m, 2H; overlapping signals), 6.98-6.98 (m, 1H), 4.39 (dd, *J*=5.0, 8.2 Hz, 1H), 3.30 (dd, *J*=5.1, 9.6 Hz, 1H), 3.21 (dd, *J*=5.6, 9.6 Hz, 1H), 3.11-2.96 (m, 2H), 2.86 (dd, *J*=5.1, 13.6 Hz, 1H), 2.38-2.23 (m, 1H), 2.36 (s, 3H), 2.32 (s, 3H), 2.28 (s, 3H), 2.23-2.12 (m, 1H), 1.93-1.59 (m, 3H), 1.54-1.41 (m, 1H); ^{13}C NMR ($CDCl_3$ major isomer, 25°C, TMS): δ=142.2, 138.8, 137.8, 137.4, 130.4, 128.2, 128.1, 127.8, 127.4, 126.7, 126.5, 123.8, 84.1, 72.0, 65.0, 57.8, 44.9, 41.3, 28.5, 22.5, 21.4; ^1H NMR ($CDCl_3$ minor isomer, 25°C, TMS): δ=7.22 (t, *J*=7.3 Hz, 1H), 7.17-7.06 (m, 4H), 7.02-6.98 (m, 2H; overlapping signals), 6.98-6.98 (m, 1H), 4.37 (dd, *J*=4.8, 8.2 Hz, 1H), 3.43 (dd, *J*=5.1, 9.6 Hz, 1H), 3.11-2.96 (m, 3H), 2.84 (dd, *J*=5.0, 13.8 Hz, 1H), 2.38-2.23 (m, 1H), 2.36 (s, 3H), 2.32 (s, 3H), 2.31 (s, 3H), 2.23-2.12 (m, 1H), 1.93-1.59 (m, 3H), 1.54-1.41 (m, 1H); ^{13}C NMR ($CDCl_3$ minor isomer, 25°C, TMS): δ=142.2, 138.9, 137.8, 137.3, 130.5, 128.2, 128.1, 127.8, 127.4, 126.7, 126.6, 123.7, 84.2, 72.0, 65.0, 57.6, 44.9, 41.3, 28.8, 22.6, 21.3; MS (70 eV): *m/z* (%): 324 (13) [M^+], 98 (12) [$C_6H_{12}N^+$], 84 (100) [$C_5H_{10}N^+$]; HRMS (ESI): *m/z* calcd for $C_{22}H_{29}NO$ (M + H^+): 324.2327; Found: 324.2324.

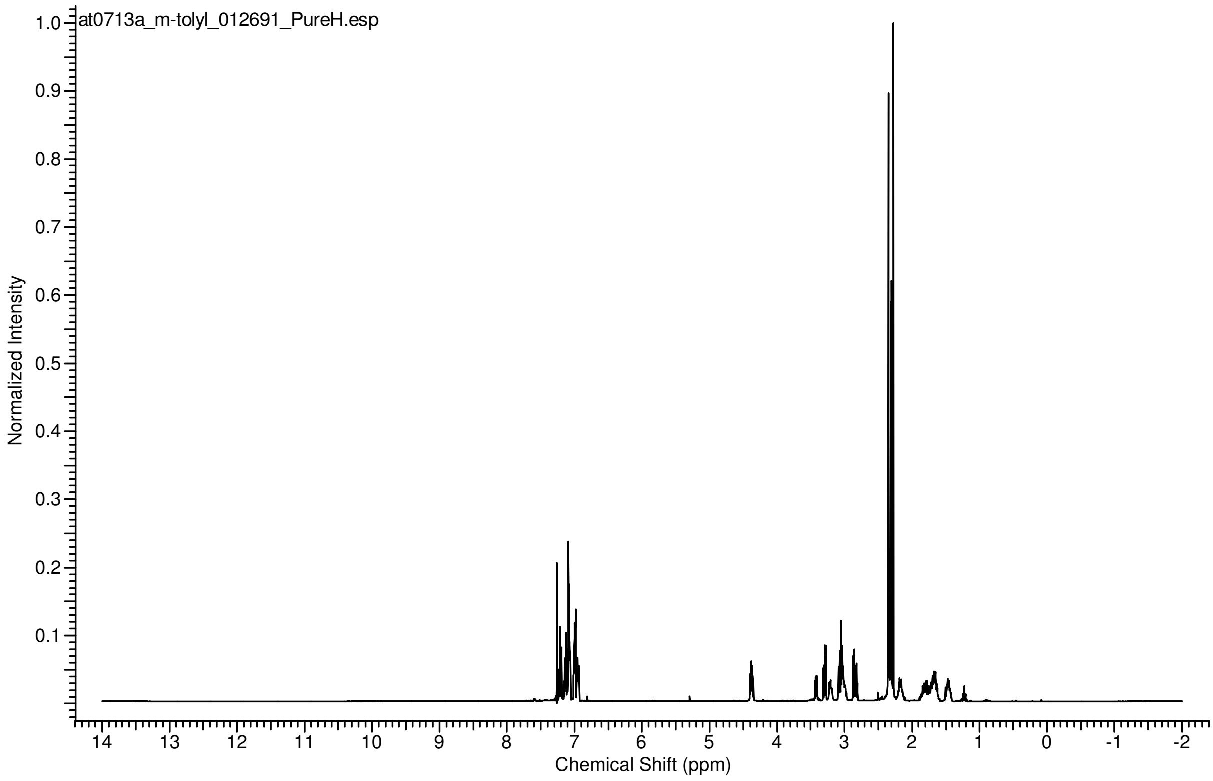
at0713a_m-tolyl_012691_PureH.esp
Normalized Intensity
Chemical Shift (ppm)

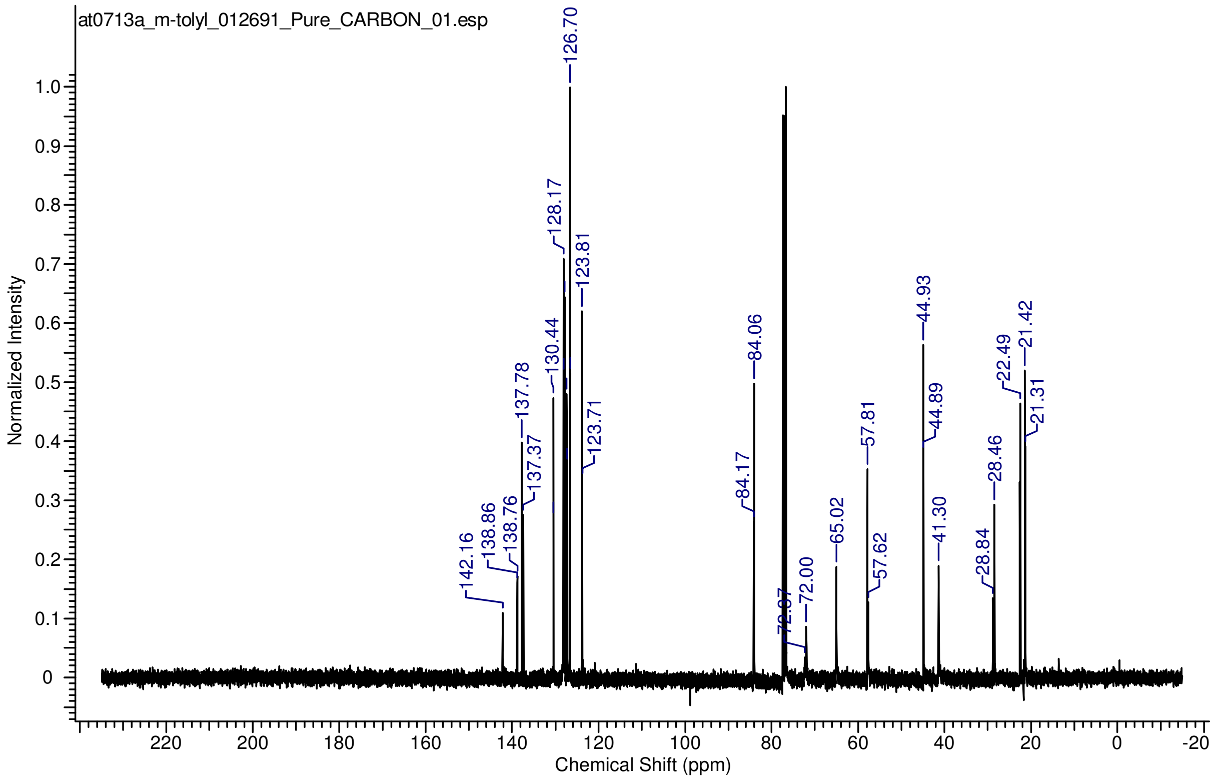
at0713a_m-tolyl_012691_Pure_CARBON_01.esp
142.16
138.86
138.76
137.78
137.37
130.44
128.17
126.70
123.81
123.71
84.17
84.06
72.00
65.02
57.81
57.62
44.93
44.89
41.30
28.84
28.46
22.49
21.42
21.31
Normalized Intensity
Chemical Shift (ppm)

Chromatogram Plot

File: m:\... \at053 chiral heck-su\avslutning\gc\is_000653_mch3.sms
Sample: IS_000653_pCH3 Operator: Operator
Scan Range: 1 - 1190 Time Range: 0.00 - 11.15 min. Date: 04/22/2010 21:55
Sample Notes: ROUTINE

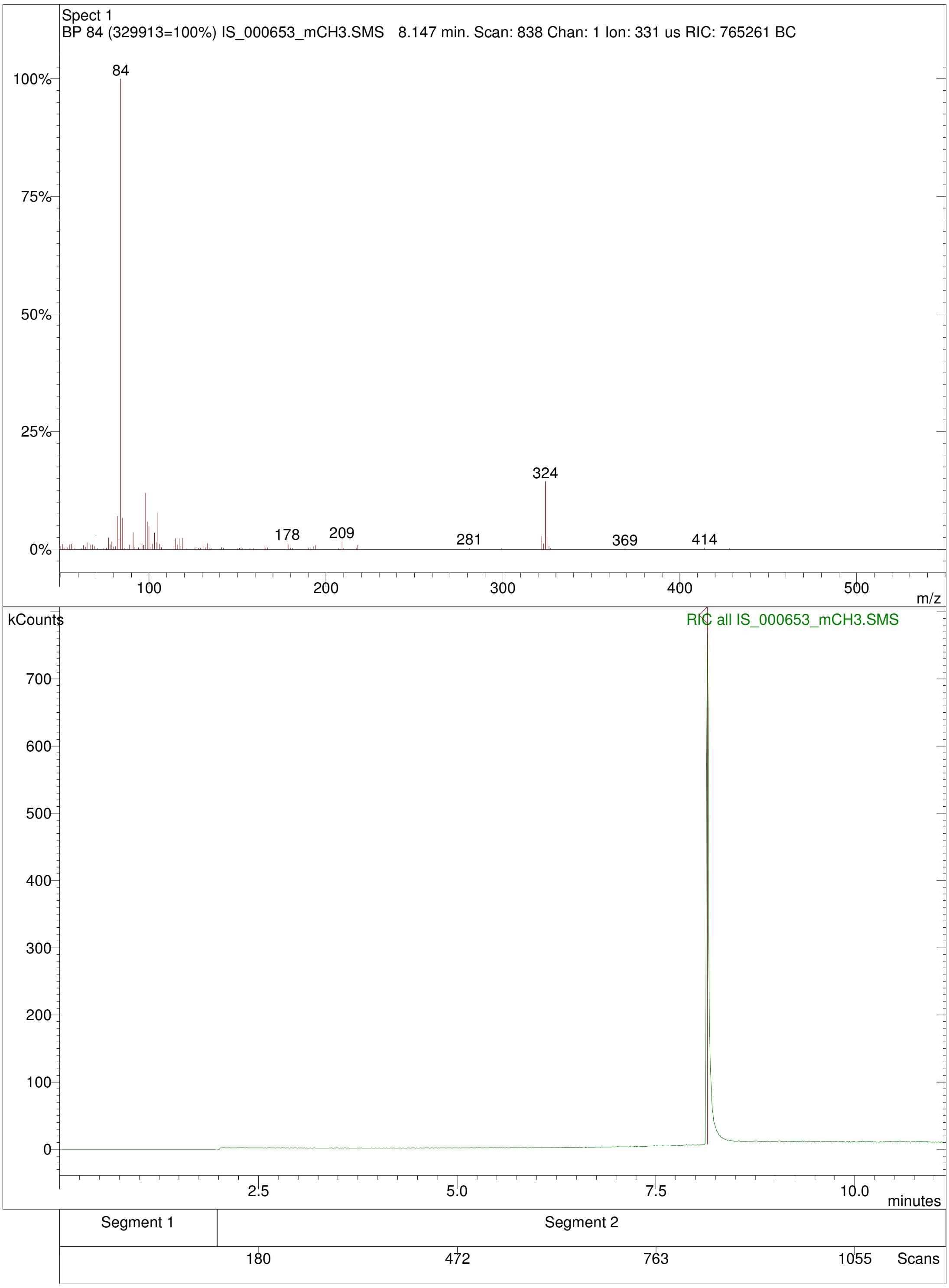

(2S)-2-((1,2-Di(naphthalen-2-yl)ethoxy)methyl)-1-methylpyrrolidine, product 5h

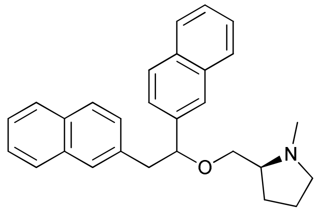

As described in the general procedure for synthesis of diarylated products **5**, **6** and **7(rac)**, but using 2-naphthaleneboronic acid (**4h**) as the arylating agent. The reaction was stirred for 24 hours and purification by DCVC afforded **5h** in 47% yield (40 mg) as a bright-yellow oil; 2.6:1 d.r.; R_f=0.3 (*i*-hexane/EtOAc/Et_3N 70:26:4); $[\alpha]_D^{22}$= 8.4° (c=14.0 in $CHCl_3$); ^1H NMR ($CDCl_3$, major diastereomer, 20°C, TMS): δ=7.86-7.67 (m, 7H), 7.64 (s, 1H), 7.51-7.39 (m, 5H), 7.30 (dd, *J*=1.2, 8.2 Hz, 1H), 4.74 (dd, *J*=5.4, 8.2 Hz, 1H), 3.39 (dd, *J*=7.8, 13.7 Hz, 1H), 3.37-3.26 (m, 2H), 3.17 (dd, *J*=5.8, 14.1 Hz, 1H), 3.14-3.04 (m, 1H), 2.52-2.40 (m, 1H), 2.29 (s, 3H), 2.26-2.16 (m, 1H), 1.88-1.59 (m, 3H), 1.53-1.43 (m, 1H); ^{13}C NMR ($CDCl_3$, major diastereomer, 20°C, TMS): $\delta_{=c}$ 139.5, 136.2, 133.4, 133.2, 133.1, 132.1, 128.19, 128.17, 128.0 , 127.8, 127.7, 127.50, 127.48, 127.4, 125.6, 125.7, 125.72, 125.7, 125.1, 124.5, 84.1, 72.2, 64.9, 57.8, 44.9, 41.5, 28.5, 22.5; ^1H NMR ($CDCl_3$, minor diastereomer, 20°C, TMS): δ=7.88-7.28 (m, 14H; the signals are hidden by major isomer), 4.73-4.68 (m, 1H; signal is hidden by major isomer), 3.45 (ddd, *J*=1.13, 5.1, 9.4 Hz, 1H), 3.37-3.27 (m, 1H), 3.22-3.11 (m, 1H), 3.04-2.96 (m, 1H), 2.39-2.30 (m, 1H), 2.34 (s, 3H), 2.19-2.10 (m, 1H), 1.88-1.79 (m, 1H), 1.75-1.56 (m, 3H), 1.54-1.43 (m, 1H); ^{13}C NMR ($CDCl_3$, minor diastereomer, 20°C, TMS): δ=139.6, 136.3, 133.4, 133.2, 133.1, 132.1, 129.0, 128.4, 128.3, 128.21, 128.17, 128.0, 127.9, 127.7, 127.5, 127.3, 125.9, 125.7, 125.1, 124.6, 84.2, 72.5, 65.1, 57.7, 44.9, 41.5, 28.9, 22.6; MS (70 eV): *m/z* (%): 396 (25) [M^+], 98 (10) [$C_6H_{12}N^+$], 84 (100) [$C_5H_{10}N^+$]; HRMS (ESI): *m/z* calcd for $C_{28}H_{29}NO$ (*M* + H^+): 396.2327; Found: 396.2329.

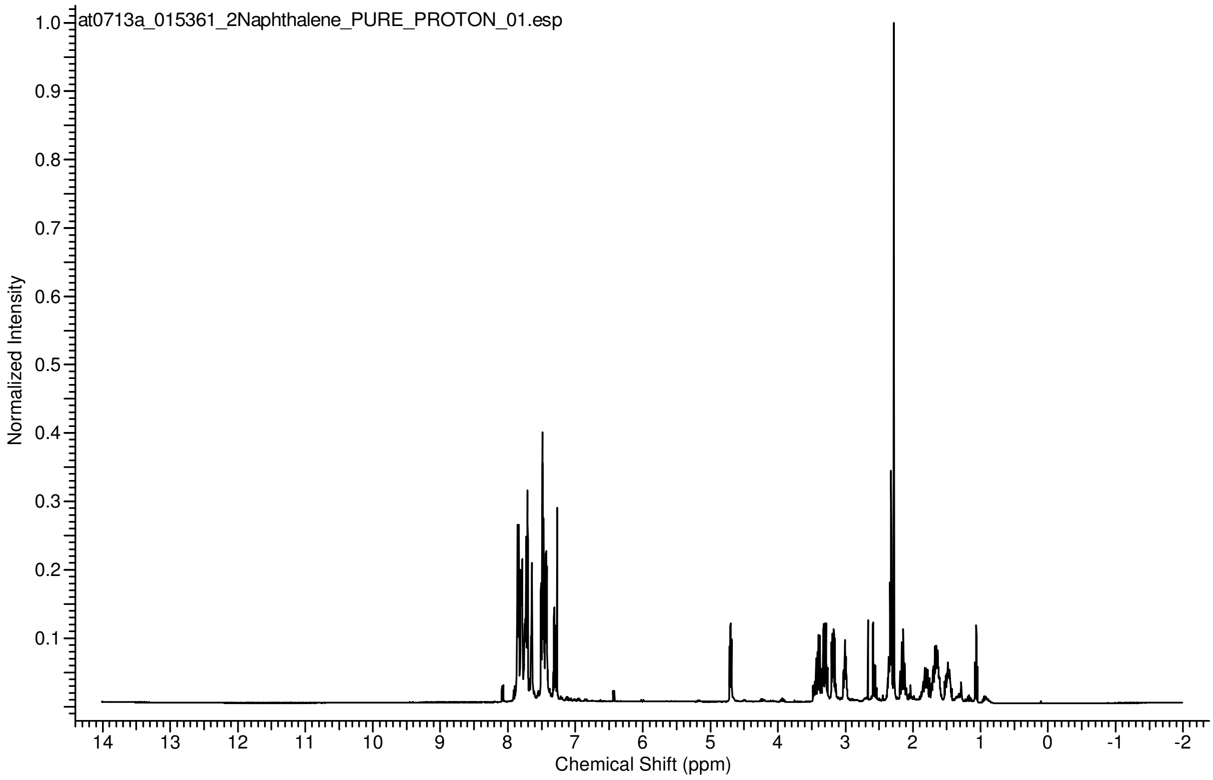
at0713a_015361_2Naphthalene_PURE_PROTON_01.esp
Normalized Intensity
Chemical Shift (ppm)

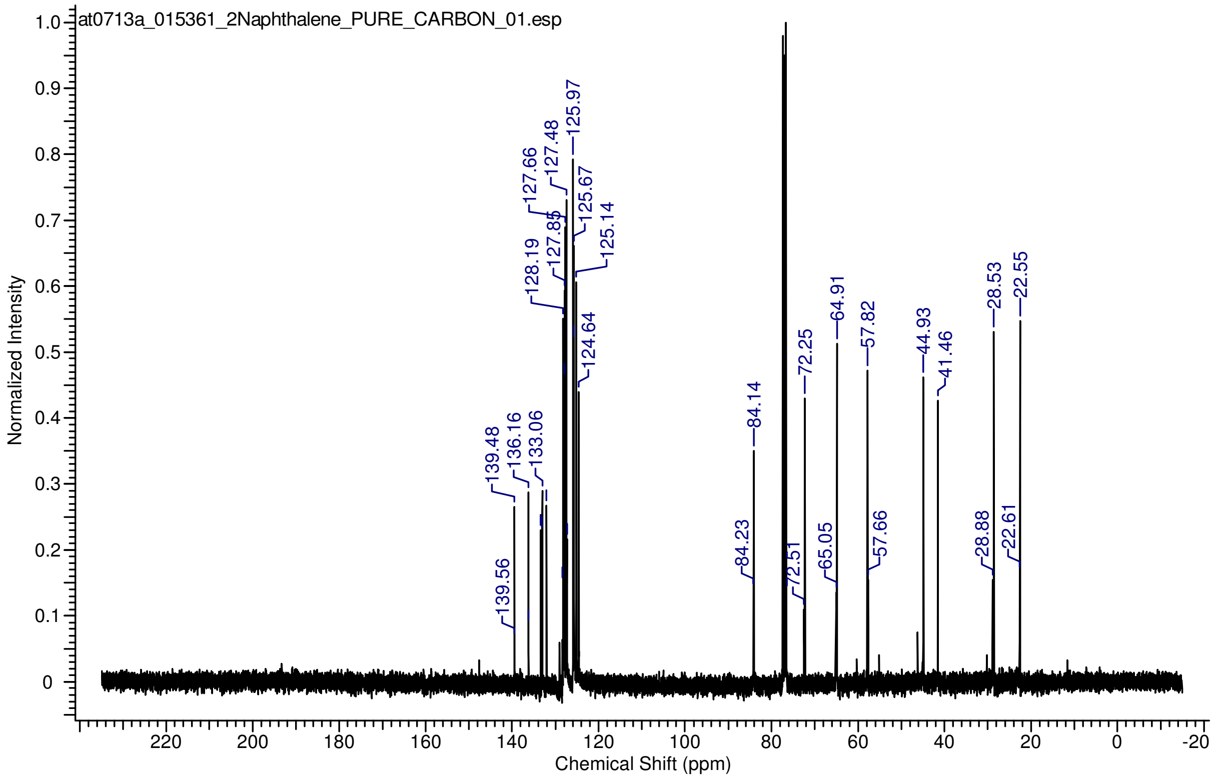
at0713a_015361_2Naphthalene_PURE_CARBON_01.esp
Normalized Intensity
Chemical Shift (ppm)
139.56
139.48
136.16
133.06
128.19
127.85
127.66
127.48
125.97
125.67
125.14
124.64
84.23
84.14
72.51
72.25
65.05
64.91
57.82
57.66
44.93
41.46
28.88
28.53
22.61
22.55

Chromatogram Plot

File: m:\... \at053 chiral heck-su\avslutning\gc\is_000662_2-naf001.sms
Sample: IS_000662_2-naf Operator: Operator
Scan Range: 1 - 1223 Time Range: 0.00 - 11.56 min. Date: 04/23/2010 12:53
Sample Notes: ROUTINE

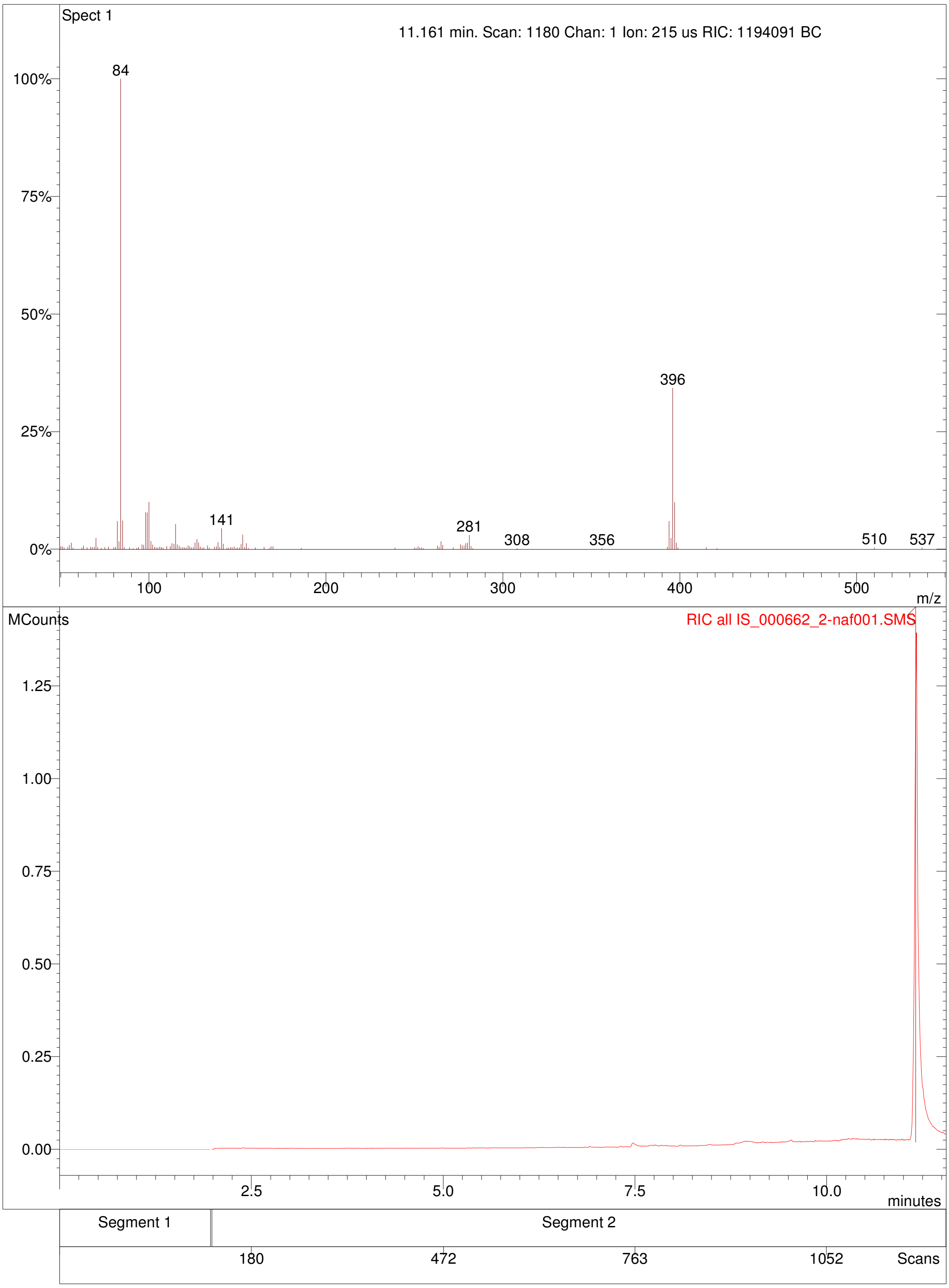

(2S)-2-((1,2-Bis(4-bromophenyl)ethoxy)methyl)-1-methylpyrrolidine, product 5i

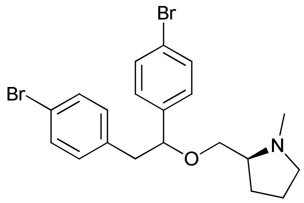

As described in the general procedure for synthesis of diarylated products **5**, **6** and **7(rac)**, but using 4-bromophenylboronic acid (**4i**) as the arylating agent. The reaction was stirred for 36 hours and purification by DCVC afforded **5i** in 52% yield (50 mg) as a pale-yellow oil; 3.4:1 d.r.; R_f=0.3 (*i*-hexane/EtOAc/Et_3N 70:26:4); $[\alpha]_D^{22}$= -7.3 (c=14.3 in $CHCl_3$); ^{1}H NMR ($CDCl_3$, major isomer, 20°C, TMS): δ=7.43 (d, *J*=8.4 Hz, 2H), 7.34 (d, *J*=8.5 Hz, 2H), 7.08 (d, *J*=8.4 Hz, 2H), 6.94 (d, *J*=8.5 Hz, 2H), 4.34 (dd, *J*=5.9, 7.4 Hz, 1H), 3.25-3.18 (m, 1H), 3.22 (dd, *J*=1.3, 5.4 Hz, 1H), 3.07-2.97 (m, 2H), 2.28 (dd, *J*=5.9, 13.7 Hz, 1H), 2.34-2.26 (m, 1H), 2.30 (m, 3H), 1.88-1.60 (m, 4H), 1.49-1.38 (m, 1H); ($CDCl_3$, major isomer, 20°C, TMS): δ=140.6, 136.9, 131.4, 131.3, 131.1, 128.5, 121.4, 120.1, 83.0, 72.4, 64.8, 57.8, 44.0, 41.6, 28.6, 22.6; HRMS (ESI): *m/z* calcd for $C_{20}H_{23}Br_2NO$ ($M + H^+$): 452.0225; Found: 452.0226.

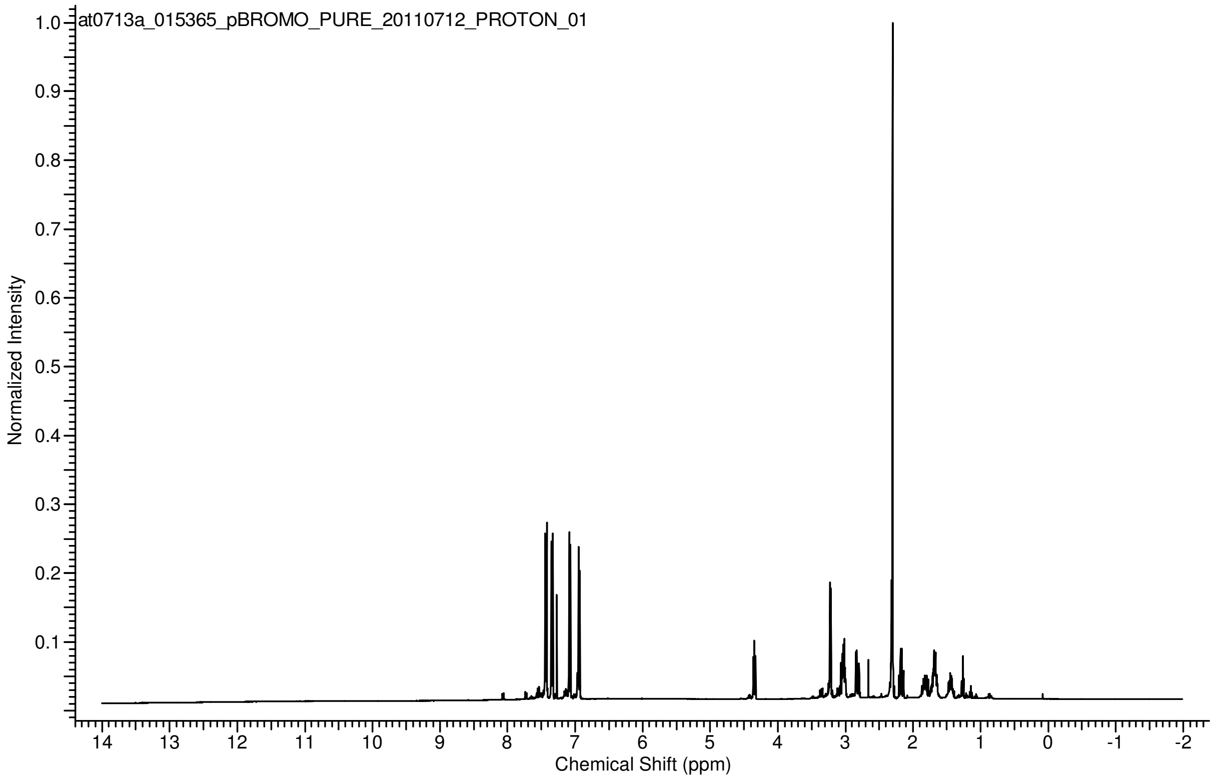
at0713a_015365_pBROMO_PURE_20110712_PROTON_01
Normalized Intensity
Chemical Shift (ppm)

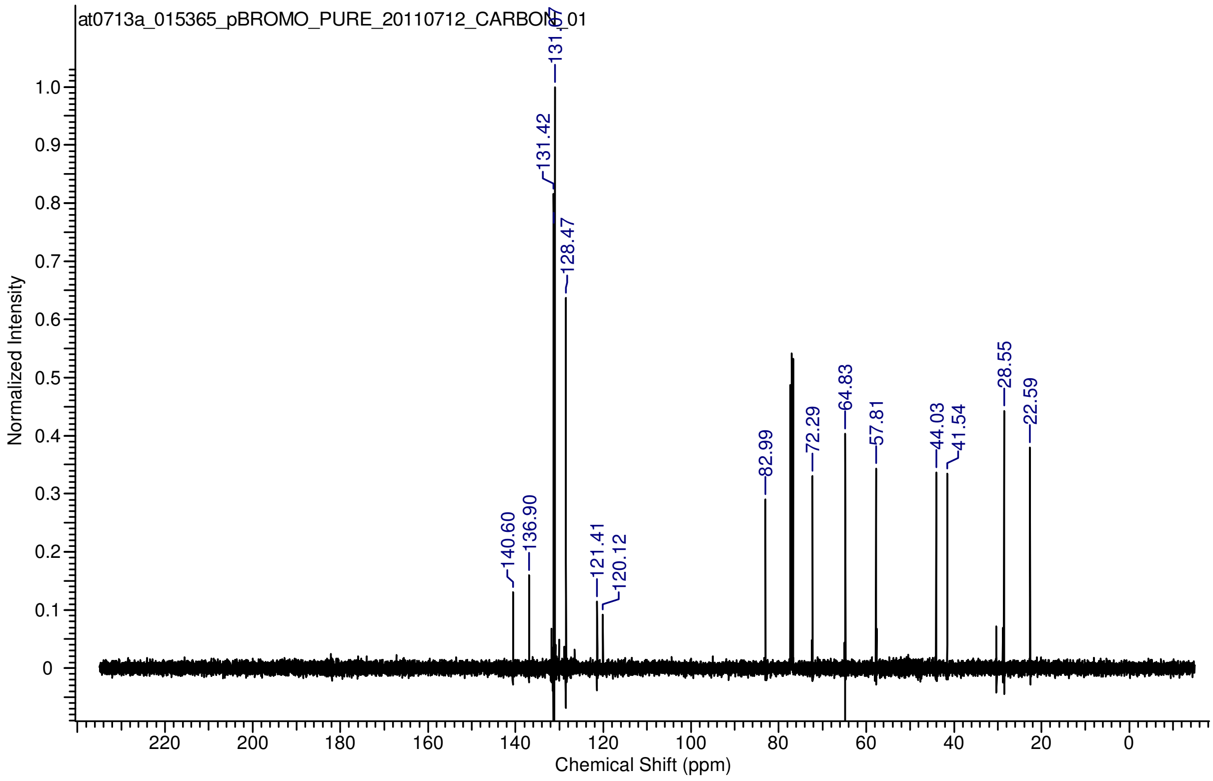
at0713a_015365_pBROMO_PURE_20110712_CARBON_01
Normalized Intensity
Chemical Shift (ppm)
140.60
136.90
131.42
128.47
121.41
120.12
82.99
72.29
64.83
57.81
44.03
41.54
28.55
22.59

4,4'-(1-((S)-1-Methylpyrrolidin-2-yl)methoxy)ethane-1,2-diyl)dibenzaldehyde, product 5j

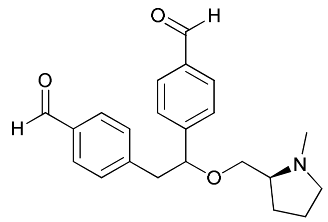

As described in the general procedure for synthesis of diarylated products **5**, **6** and **7(rac)**, but using 4-formylphenylboronic acid (**4j**) as the arylating agent. The reaction was stirred for 24 hours and purification by DCVC afforded **5j** in 64% yield (48 mg) as pale orange oil; R_f=0.2 (*i*-hexane/EtOAc/Et_3N 40:46:4);3.0:1 d.r.; $[\alpha]_D^{22}$= -13.5 (c=10.5 in $CHCl_3$); ^1H NMR ($CDCl_3$, major diastereomer, 20°C, TMS): δ=10.00 (s, 1H), 9.97 (s, 1H), 7.86-7.82 (m, 2H), 7.77-7.73 (m, 2H), 7.741-7.37 (m, 2H), 7.29-7.24 (m, 2H), 4.59 (dd, *J*=5.6, 7.2 Hz, 1H), 3.34 (dd, *J*=5.5, 9.6 Hz, 1H), 3.23-3.13 (m, 1H), 3.12-2.96 (m, 3H), 2.46-2.34 (m, 1H), 2.33 (s, 3H), 2.27-2.17 (m, 1H), 1.90-1.78 (m, 1H), 1.78-1.62 (m, 2H), 1.51-1.41 (m, 1H); ^{13}C NMR ($CDCl_3$, major diastereomer, 20°C, TMS): δ=191.9, 191.8, 148.4, 145.0, 144.9, 136.0, 134.9, 130.31, 129.9, 129.6, 127.3, 82.9, 72.3, 65.1, 57.7, 44.7, 41.5, 28.4, 22.6; ^1H NMR ($CDCl_3$, minor diastereomer, 20°C, TMS): δ=10.00 (s, 1H), 9.98 (s, 1H), 7.86-7.82 (m, 2H), 7.77-7.73 (m, 2H), 7.741-7.37 (m, 2H), 7.29-7.24 (m, 2H), 4.56 (dd, *J*=5.2, 7.6 Hz, 1H), 3.42 (dd, *J*=5.3, 9.5 Hz, 1H),), 3.28 (dd, *J*=5.1, 9.6 Hz, 1H), 3.23-3.13 (m, 1H), 3.12-2.96 (m, 2H), 2.46-2.34 (m, 1H), 2.34 (s, 3H), 2.27-2.17 (m, 1H), 1.90-1.78 (m, 1H), 1.78-1.62 (m, 2H), 1.51-1.41 (m, 1H); ^{13}C NMR ($CDCl_3$, minor diastereomer, 20°C, TMS): δ=192.0, 191.8, 148.5, 145.0, 136.0, 134.9, 130.3, 129.9, 129.5, 127.3, 83.0, 72.6, 65.1, 57.6, 44.7, 41.5, 28.7, 22.7; MS (70 eV): *m/z* (%): 352 (3) [M^+], 98 (9) [$C_6H_{12}N^+$], 84 (100) [$C_5H_{10}N^+$]; HRMS (ESI): *m/z* calcd for $C_{22}H_{25}NO$ (M + H^+): 352.1913; Found: 352.1910.

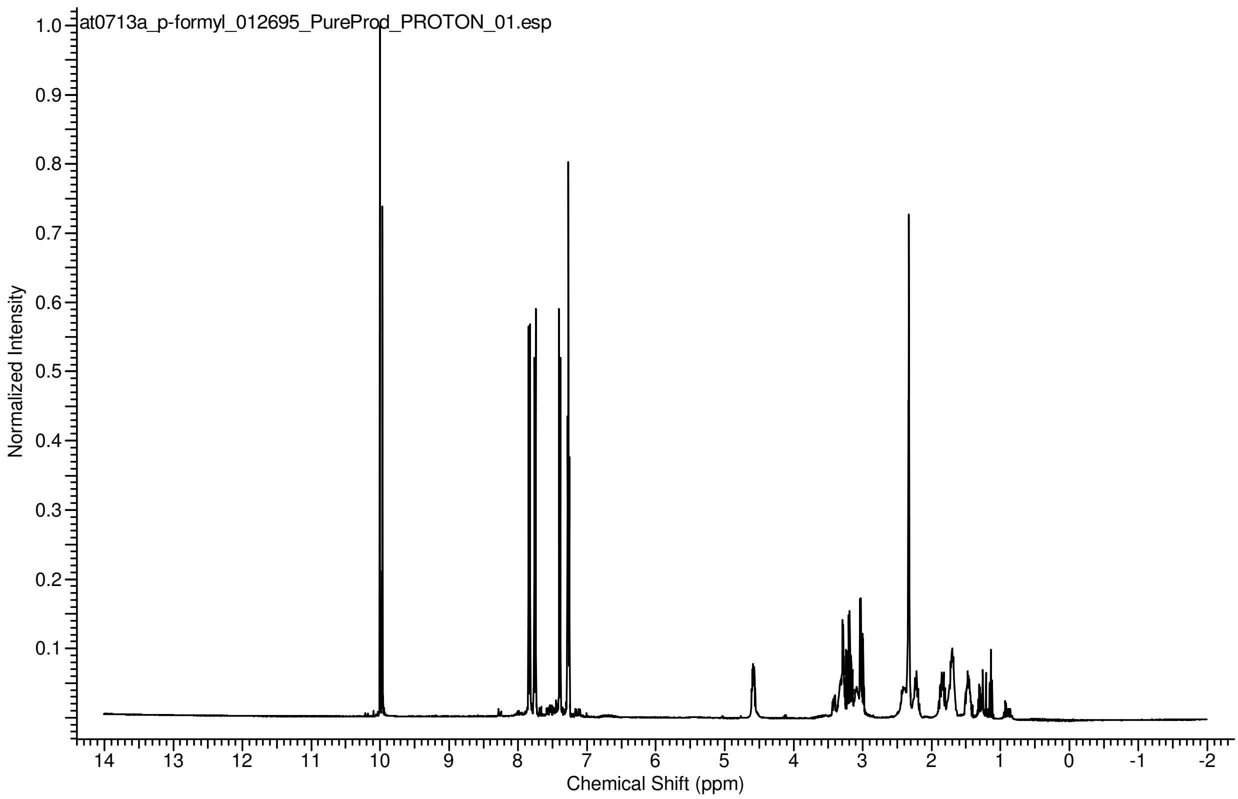
at0713a_p-formyl_012695_PureProd_PROTON_01.esp
Normalized Intensity
Chemical Shift (ppm)

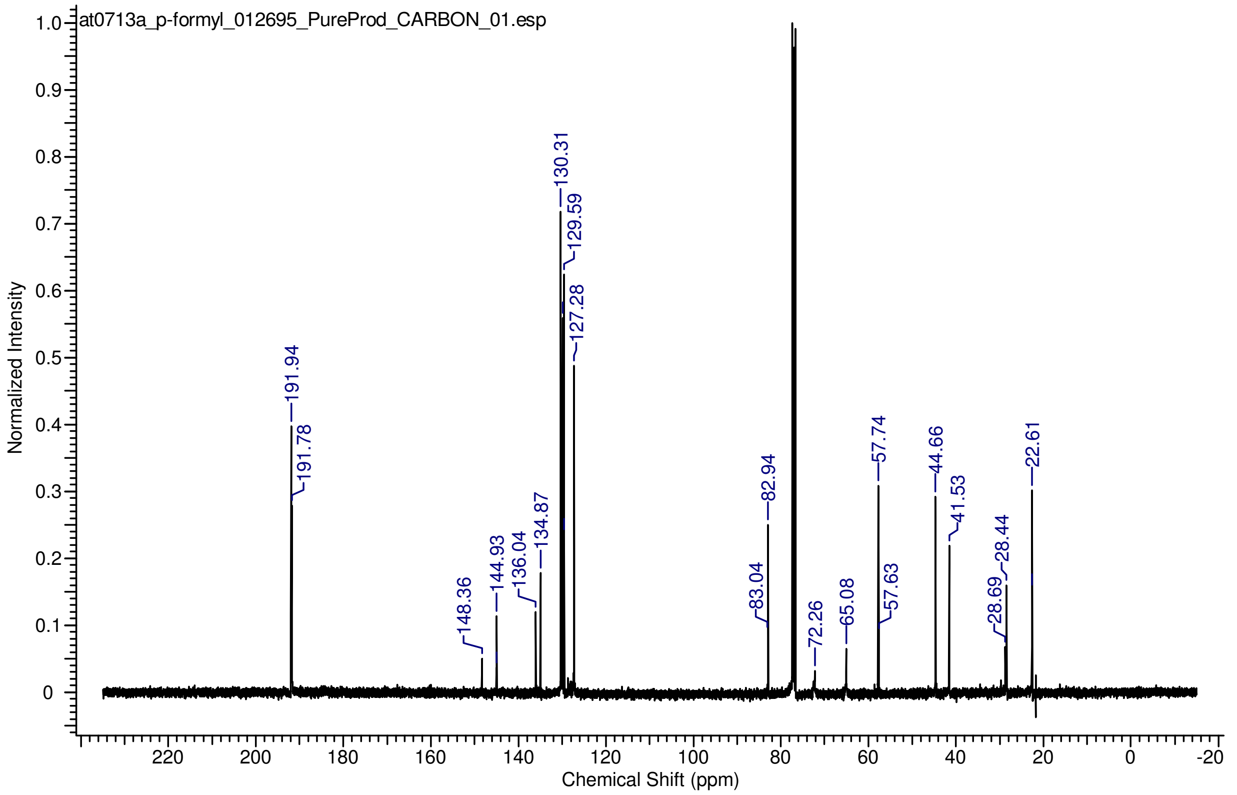
at0713a_p-formyl_012695_PureProd_CARBON_01.esp
Normalized Intensity
Chemical Shift (ppm)
191.94
191.78
148.36
144.93
136.04
134.87
130.31
129.59
127.28
83.04
82.94
72.26
65.08
57.74
57.63
44.66
41.53
28.69
28.44
22.61

Chromatogram Plot

File: m:\... \synthesis\at053 chiral heck-su\avslutning\gc\is_000667_cho.sms
Sample: IS_000667_CHO
Operator: Operator
Scan Range: 1 - 1190 Time Range: 0.00 - 11.16 min.
Date: 04/22/2010 22:25
Sample Notes: ROUTINE

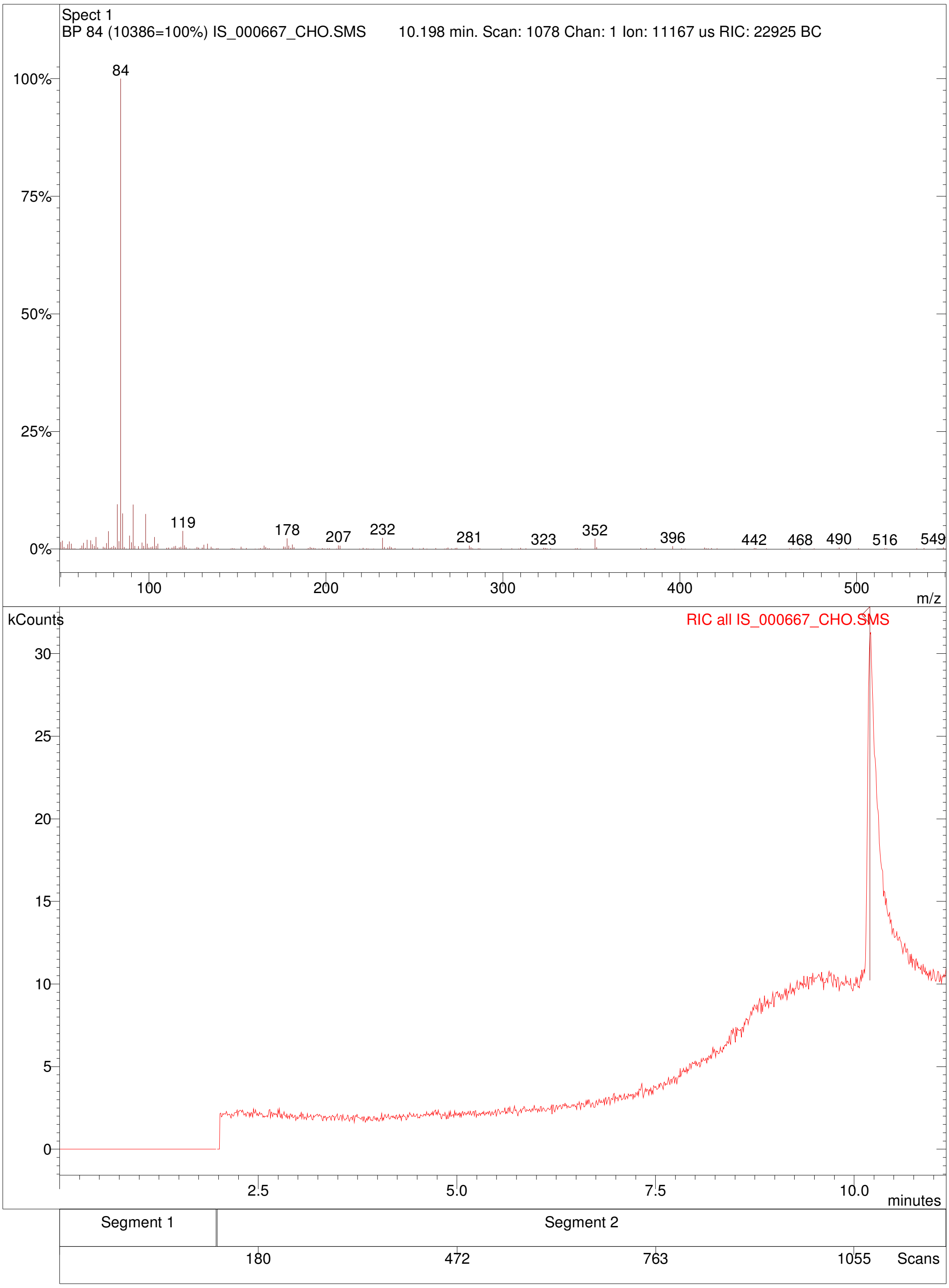

(2S)-2-((1,2-Bis(4-(trifluoromethyl)phenyl)ethoxy)methyl)-1-methylpyrrolidine, product 5k

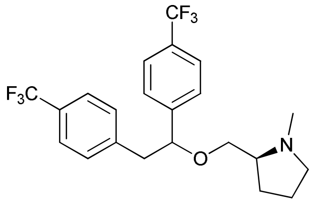

As described in the general procedure for synthesis of diarylated products **5**, **6** and **7(rac)**, but using 4-(trifluoromethyl)phenylboronic acid (**4k**) as the arylating agent. The reaction was stirred for 24 hours and purification by DCVC afforded **5k** in 41% yield (37 mg) as a bright yellow oil; 4.7 d.r.; R_f=0.3 (*i*-hexane/EtOAc/Et_3N 70:26:4); $[\alpha]_D^{22}$= -29.0° (c=10.7 in $CHCl_3$); ^1H NMR ($CDCl_3$, major isomer, 25°C, TMS): δ=7.59 (d, *J*=7.8 Hz, 2H), 7.50 (d, *J*=7.8 Hz, 2H), 7.36 (d, *J*=7.8 Hz, 2H), 7.23 (d, *J*=7.8 Hz, 2H), 4.53 (dd, *J*=5.3, 7.4 Hz, 1H), 3.31-3.22 (m, 2H), 3.16 (dd, *J*=7.9, 13.7 Hz, 1H), 3.09-3.00 (m, 1H), 2.97 (dd, *J*=5.6, 13.7 Hz, 1H), 2.42-2.32 (m, 1H), 2.29 (s, 3H), 2.25-2.15 (m, 1H), 1.90-1.77 (m, 1H), 1.77-1.63 (m, 2H), 1.55-1.39 (m, 1H); ^{13}C NMR ($CDCl_3$, major isomer, 25°C, TMS): δ=145.5, 141.9, 130.1 (q, *J*=32.2 Hz), 129.9, 128.7 (q, *J*=33.0 Hz), 127.0, 125.4 (q, *J*=3.8 Hz), 125.0 (q, *J*=3.8 Hz), 124.3 (q, *J*=271.9 Hz), 124.1 (q, *J*=273.0 Hz), 82.9, 72.3, 65.1, 57.8, 44.5, 41.5, 28.5, 22.6; ^1H NMR ($CDCl_3$, minor isomer, 25°C, TMS): δ=7.63-7.21 (m, 8H; signals are hidden by major isomer), 4.51 (dd, *J*=5.3, 7.6 Hz, 1H), 3.39 (dd, *J*=5.3, 9.5 Hz, 1H), 3.31-3.22 (m, 1H; the split is hidden by major isomer), 3.13 (dd, *J*=5.7, 9.5 Hz, 1H), 3.09-3.00 (m, 1H), 2.97-2.92 (m, 1H; the signal is hidden by major isomer), 2.42-2.32 (m, 1H), 2.33 (s, 3H), 2.25-2.15 (m, 1H), 1.90-1.77 (m, 1H), 1.77-1.63 (m, 2H), 1.55-1.39 (m, 1H); ^{13}C NMR ($CDCl_3$, minor isomer, 25°C, TMS): δ_C= 145.7, 142.0, 130.0, 126.9, [the split signals were too weak to distinguish, therefore these are not reported], 83.0, 72.6, 65.1, 57.7, 44.5, 41.5, 28.6, 22.6; MS (70 eV): *m/z* (%): 432 (4) [M^+], 98 (13) [$C_6H_{12}N^+$], 84 (100) [$C_5H_{10}N^+$]; HRMS (ESI): *m/z* calcd for $C_{22}H_{23}F_6NO$ (M + H^+): 432.1762; Found: 432.1760.

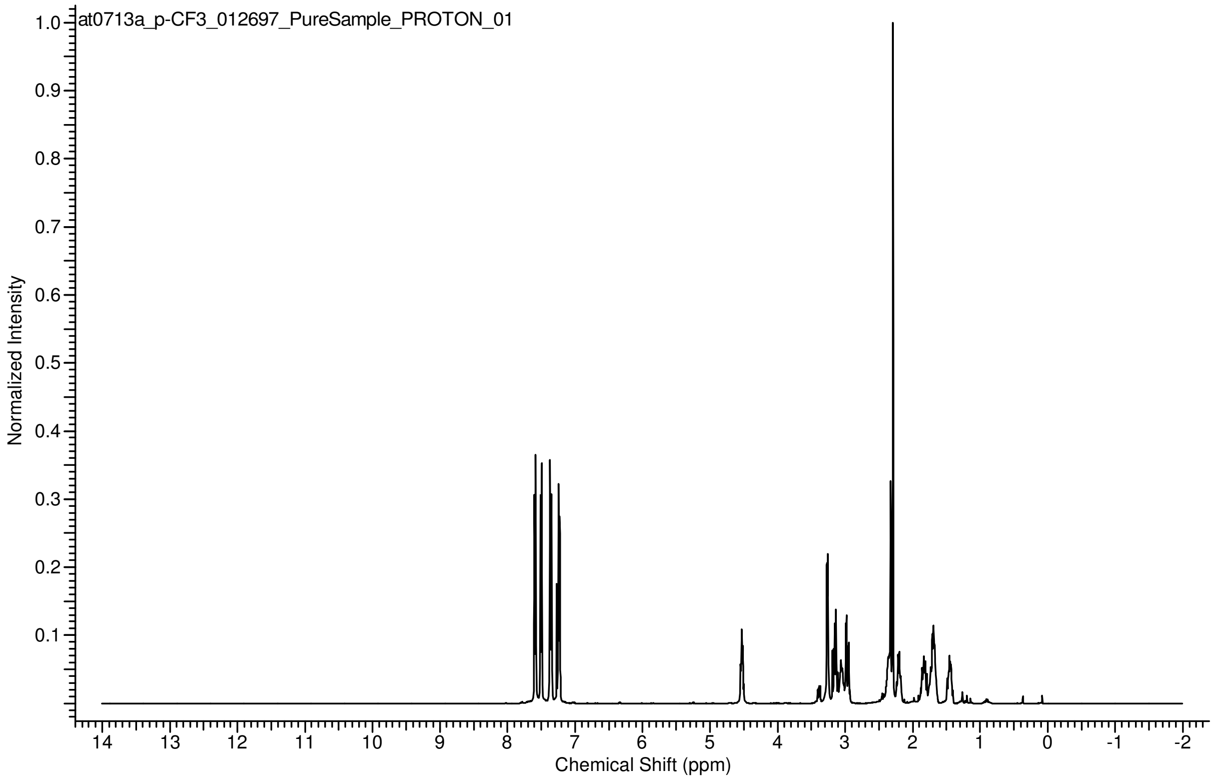
at0713a_p-CF3_012697_PureSample_PROTON_01
Normalized Intensity
Chemical Shift (ppm)

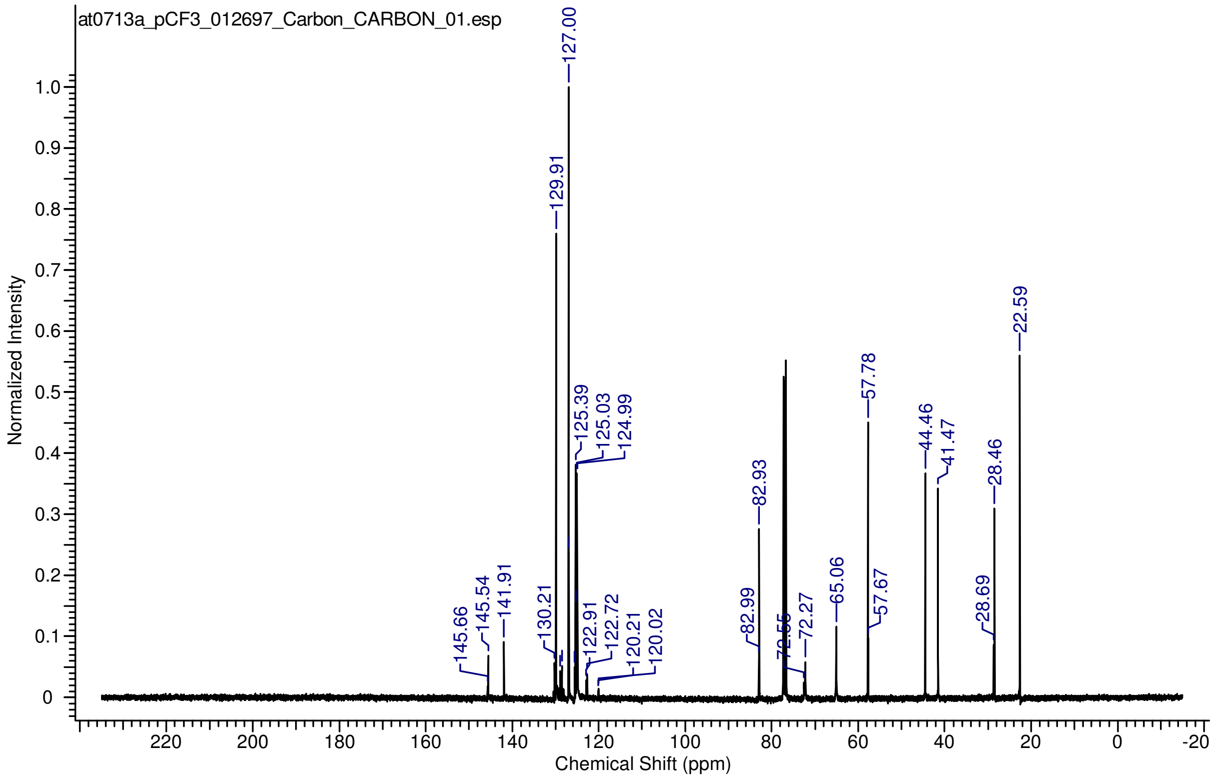
at0713a_pCF3_012697_Carbon_CARBON_01.esp
Normalized Intensity
Chemical Shift (ppm)
145.66
145.54
141.91
130.21
129.91
127.00
125.39
125.03
124.99
122.91
122.72
120.21
120.02
82.99
82.93
72.27
65.06
57.78
57.67
44.46
41.47
28.69
28.46
22.59

Chromatogram Plot

File: m:\... \synthesis\at053 chiral heck-su\avslutning\gc\is_000659_cf3.sms
Sample: IS_000659_CF3 Operator: Operator
Scan Range: 1 - 1188 Time Range: 0.00 - 11.15 min. Date: 04/22/2010 23:11
Sample Notes: ROUTINE

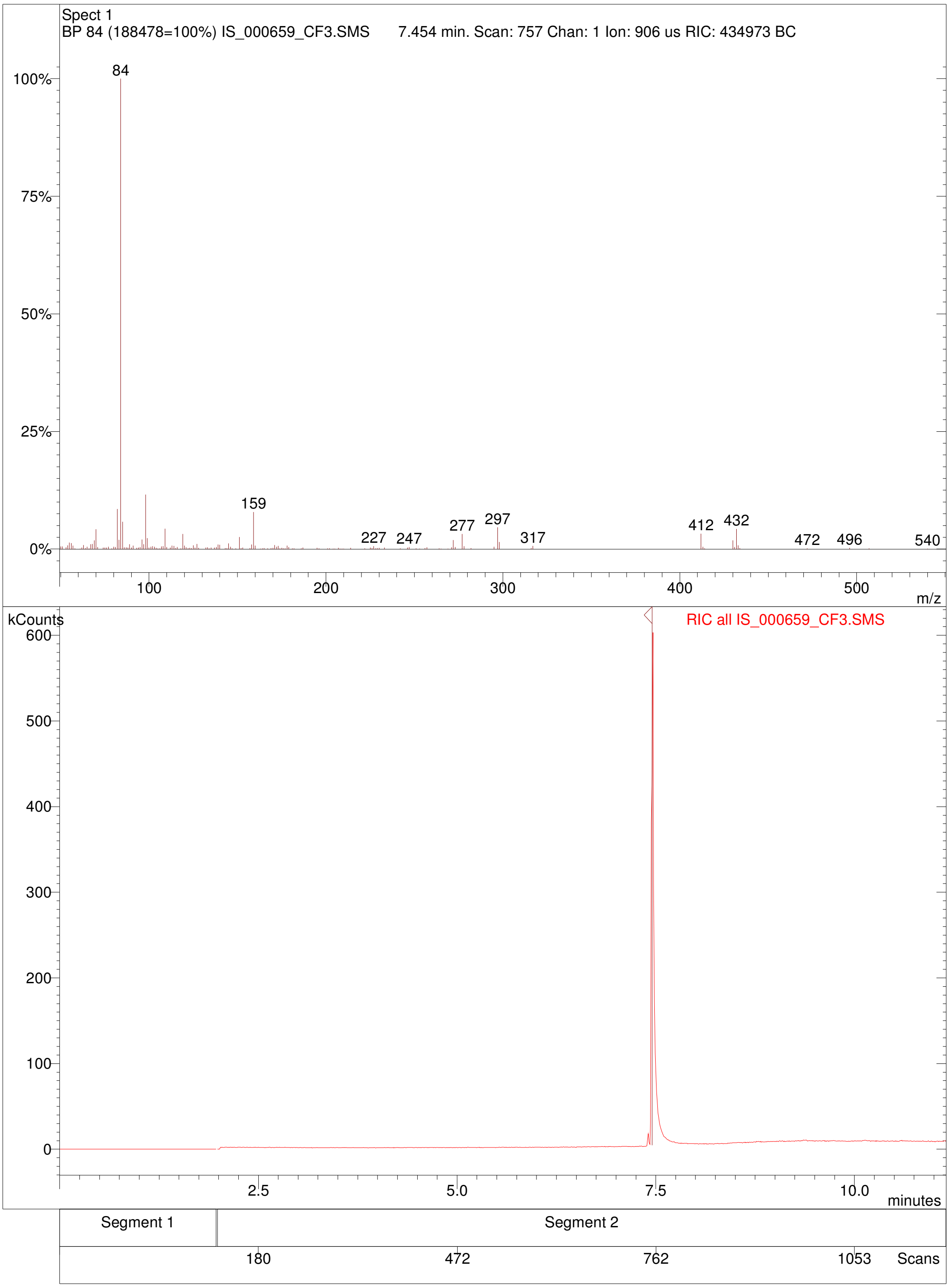

(3S)-3-(1,2-Bis(4-methoxyphenyl)ethoxy)-1-methylpiperidine, product 6a

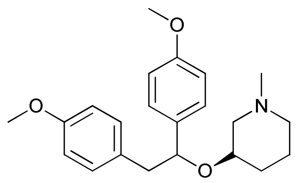

As described in the general procedure for synthesis of diarylated products **5**, **6** and **7(rac)**, but using 4-methoxyphenylboronic acid (**4a**) as the arylating agent. The reaction was stirred for 36 hours and purification by DCVC afforded **6a** in 12% yield (8.9 mg) as a bright-yellow oil; 2.2:1 d.r.; R_f=0.3 (*i*-hexane/EtOAc/Et_3N 60:36:4); $[\alpha]_D^{22}$= 4.5 (c=2.2 in $CHCl_3$); ^{1}H NMR ($CDCl_3$, major isomer, 20°C, TMS): δ= 7.98 (d, *J*=8.4 Hz, 2H), 7.91 (d, *J*=8.2 Hz, 2H), 7.32 (d, *J*=8.4 Hz, 2H), 7.19 (d, *J*=8.2 Hz, 2H), 4.61 (dd, *J*=5.4, 7.9 Hz, 1H), 3.92 (s, 3H), 3.91 (s, 3H), 3.29-3.21 (m, 1H), 3.11 (dd, *J*=7.9, 13.5 Hz, 1H), 2.91 (dd, *J*=5.4, 13.5 Hz, 1H), 2.64-2.55 (m, 1H), 2.52-2.44 (m, 1H), 2.17 (s, 3H), 1.98-1.84 (m, 2H), 1.76-1.58 (m, 2H), 1.41-1.24 (m, 1H), 1.10-0.98 (m, 1H); ^{13}C NMR ($CDCl_3$, major isomer, 20°C, TMS): δ= 167.1, 166.9, 147.8, 143.7, 129.7, 129.6, 129.4, 126.5, 80.6, 73.5, 61.1, 55.4, 52.1, 52.0, 46.3, 45.1, 30.3, 28.9, 23.1; HRMS (ESI): *m/z* calcd for $C_{20}H_{25}NO$ (M + H^+): 356.2226; Found: 356.2223

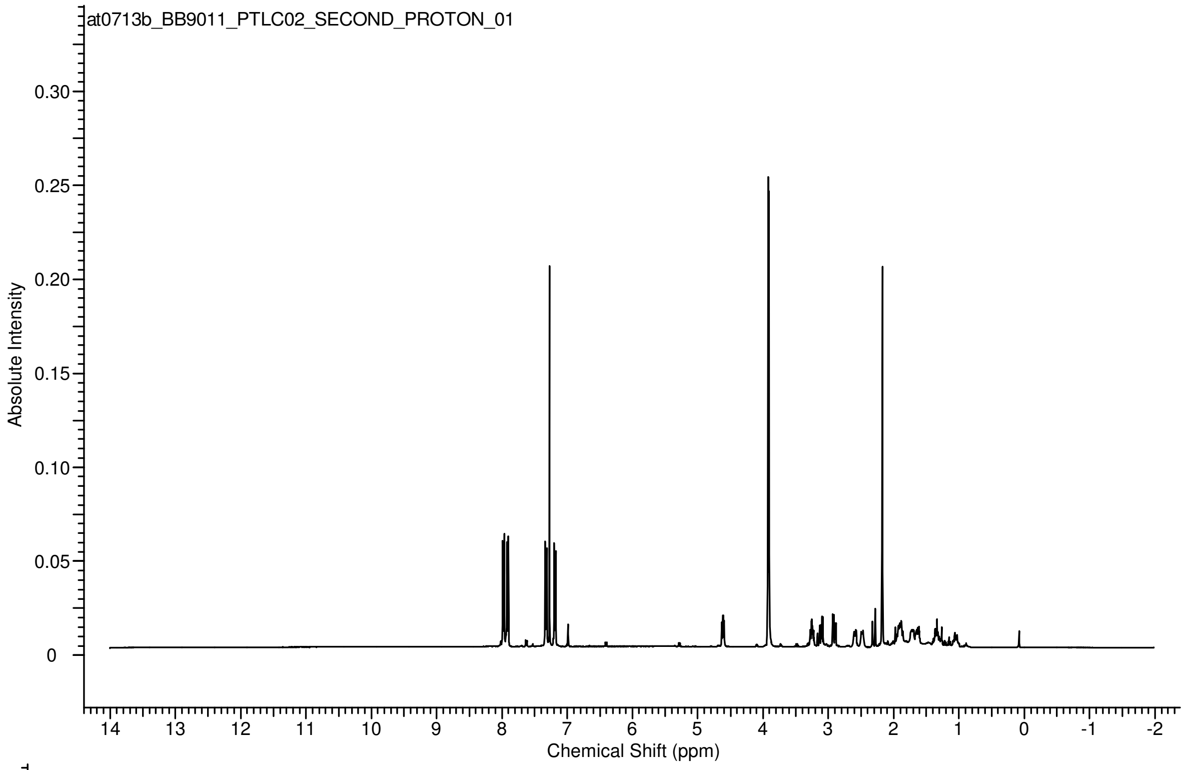
at0713b_BB9011_PTLC02_SECOND_PROTON_01
Absolute Intensity
Chemical Shift (ppm)

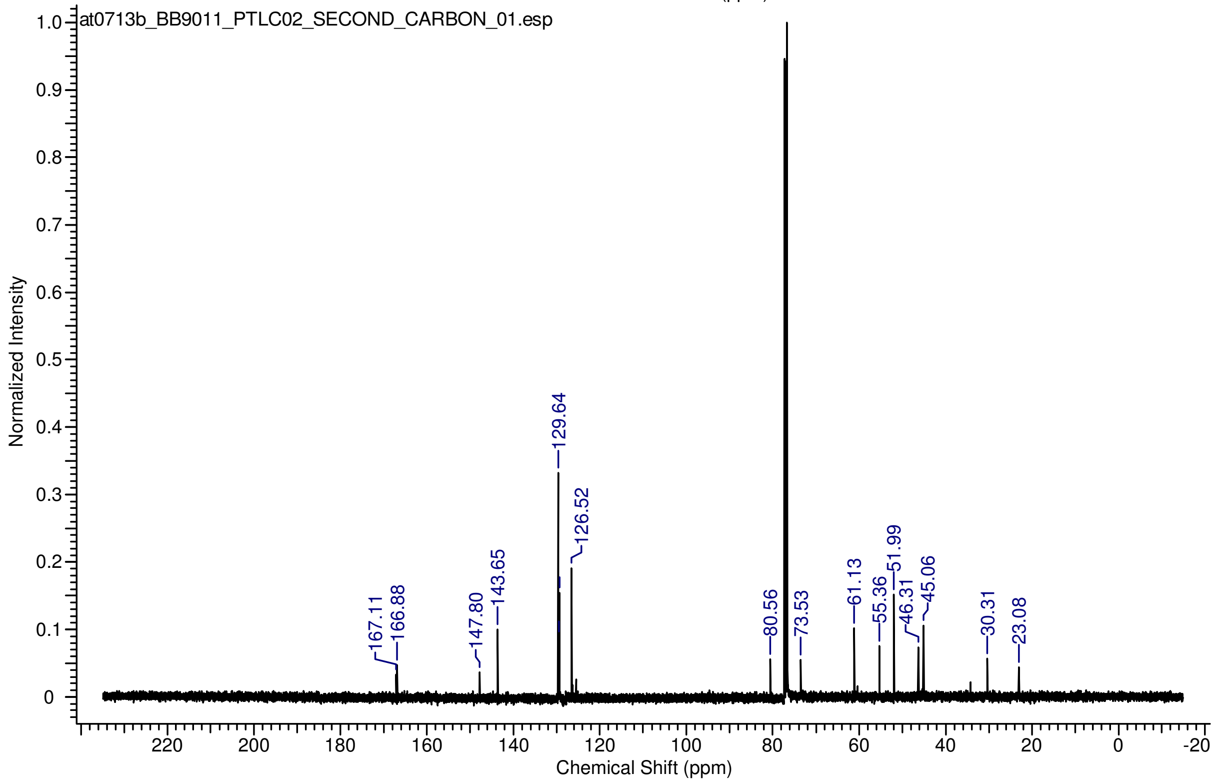
at0713b_BB9011_PTLC02_SECOND_CARBON_01.esp
Normalized Intensity
Chemical Shift (ppm)
167.11
166.88
147.80
143.65
129.64
126.52
80.56
73.53
61.13
55.36
51.99
46.31
45.06
30.31
23.08

LC-MS Analysis Report

General Information

Sample ID:	at0713b_BB9011b_crude
Date & Time:	8/16/2011 10:18:23 AM
Data File:	D:\BMC\Users\alejandro\results\at07\11 v34\at0713b_BB9011b_crude_11199.D
Data Processing:	Mass spectrum report with integrated +/-TIC, UV chromatograms displayed

Chromatogram

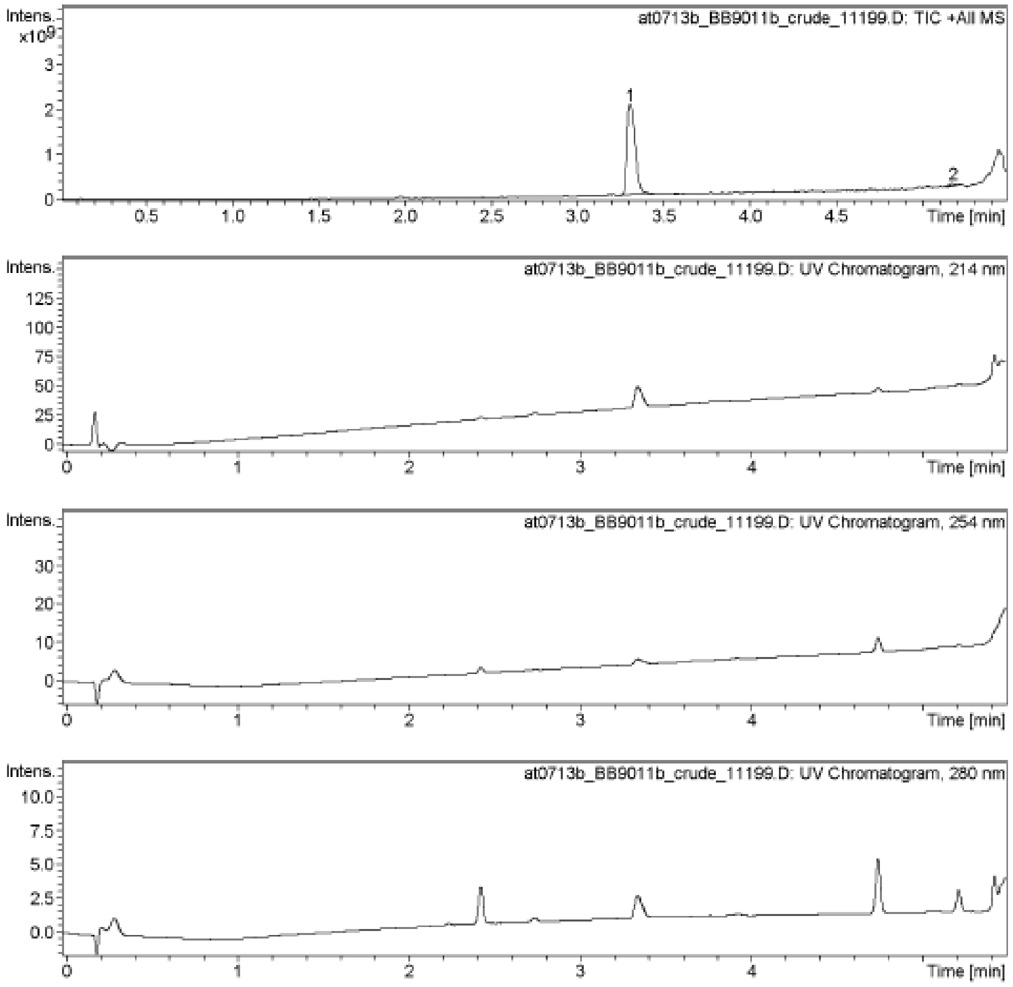

Cmpd 1, 3.31 min

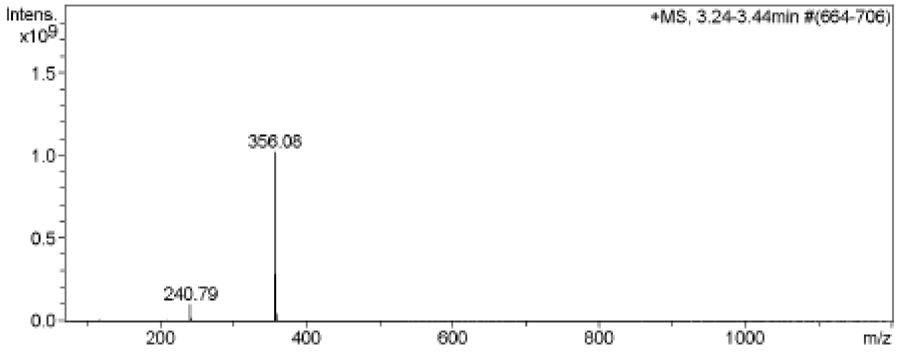

#	m/z
1	240.79
2	356.08
3	357.06

(3S)-3-(1,2-Diphenylethoxy)-1-methylpiperidine, product 6c

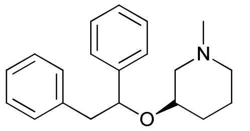

As described in the general procedure for synthesis of diarylated products **5**, **6** and **7(rac)**, but using phenylboronic acid (**4c**) as the arylating agent. The reaction was stirred for 36 hours and purification by DCVC afforded **6c** in 63% yield (39 mg) as a tan oil; 4.7:1 d.r.; R_f=0.3 (*i*-hexane/EtOAc/Et_3N 70:26:4); $[\propto]_{D}^{22}$= -2.7 (c=5.5 in $CHCl_3$); ^1H NMR ($CDCl_3$, major isomer, 20°C, TMS): *δ*=7.34-7.10 (m, 10H), 4.54 (dd, *J*=5.5, 8.0 Hz, 1H), 3.33-3.25 (m, 1H), 3.08 (dd, *J*=8.0, 13.6 Hz, 1H), 2.86 (dd, *J*=5.5, 13.6 Hz, 1H), 2.66-2.46 (m, 2H), 2.19 (s, 3H), 2.00-1.82 (m, 2H), 1.79-1.56 (m, 2H), 1.44-1.29 (m, 1H), 1.12-1.00 (m, 1H); ^{13}C NMR ($CDCl_3$, major isomer, 20°C, TMS): *δ*=142.9, 138.8, 129.6, 128.2, 127.9, 127.4, 126.6, 126.0, 81.4, 72.84, 61.2, 55.4, 46.3, 45.6, 28.9, 23.0; MS (70 eV): *m/z* (%): 296 (30) [M^+], 98 (100) [$C_6H_{12}N^+$], 71 (80) [$C_4H_9N^+$]; HRMS (ESI): *m/z* calcd for $C_{20}H_{25}NO$ (M + H^+): 296.2014; Found: 296.2017.

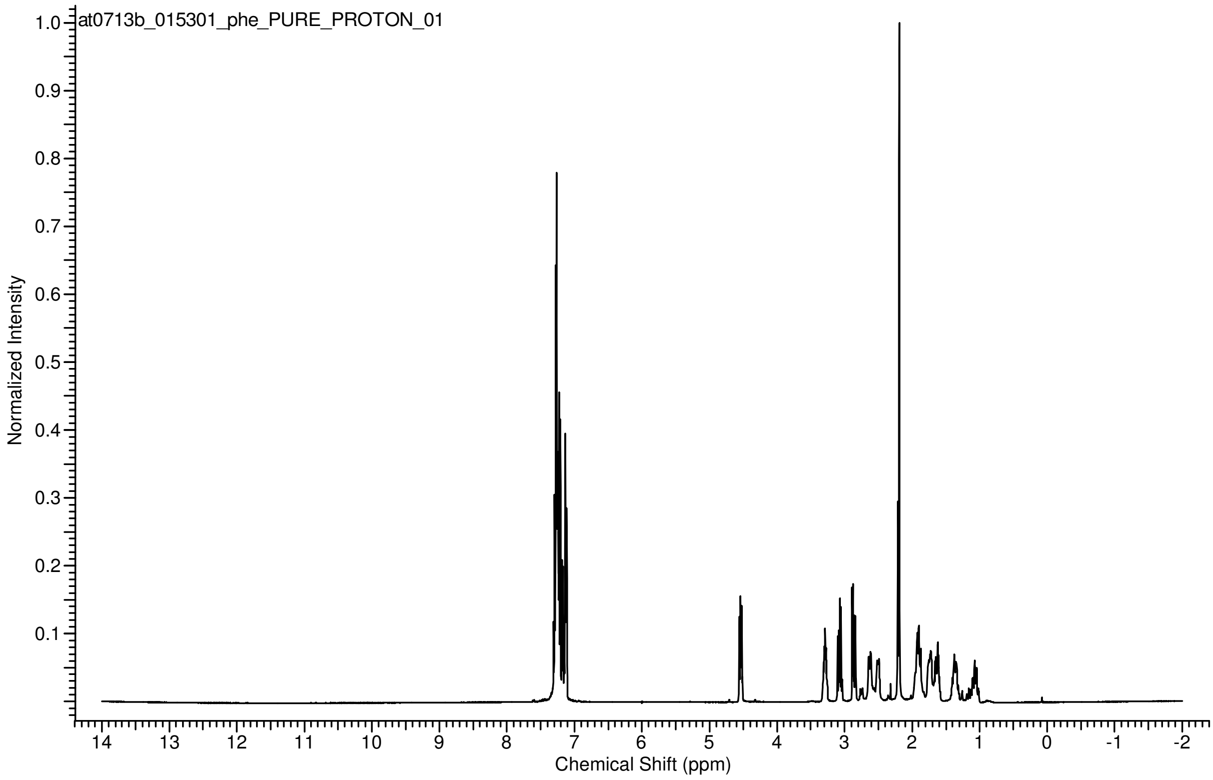

at0713b_015301_phe_PURE_PROTON_01
Normalized Intensity
Chemical Shift (ppm)

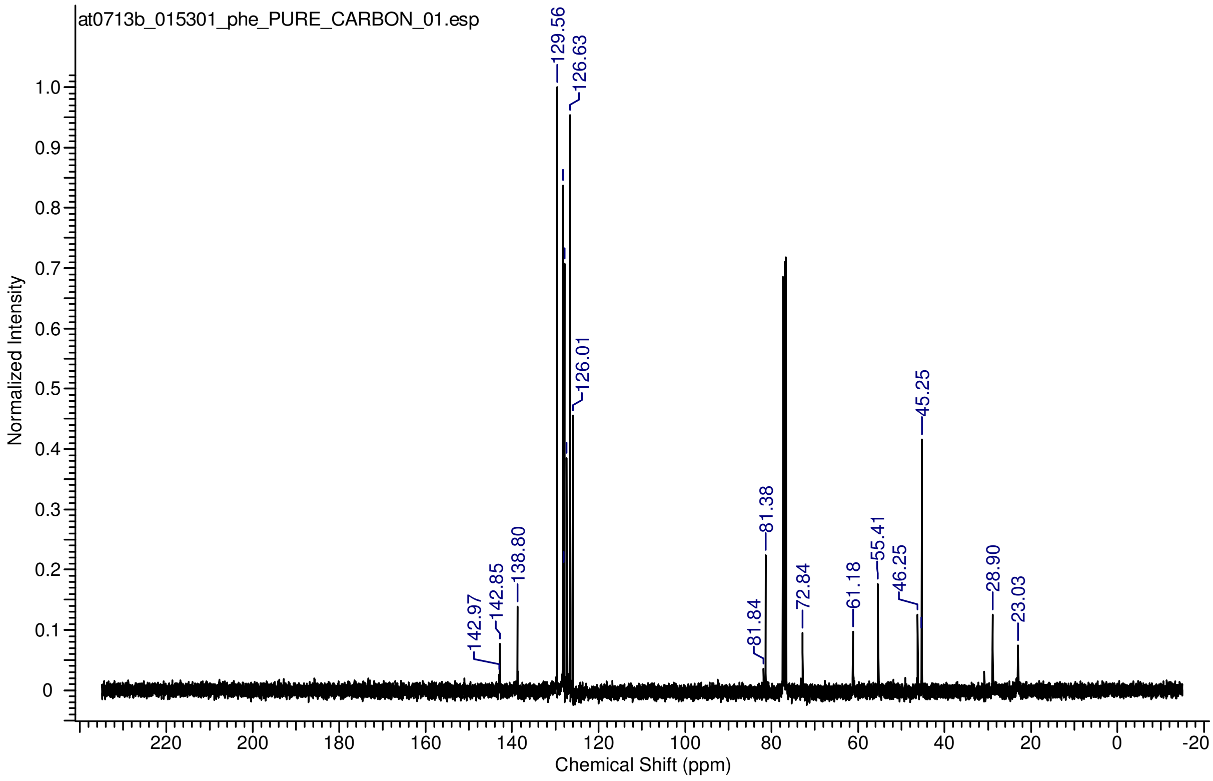

at0713b_015301_phe_PURE_CARBON_01.esp
129.56
126.63
126.01
142.97
142.85
138.80
81.84
81.38
72.84
61.18
55.41
46.25
45.25
28.90
23.03
Normalized Intensity
Chemical Shift (ppm)

Chromatogram Plot

File: m:\... \pek\resterande gc_lc\at0713bphe_015301\at0713b_015301_fr15.sms
Sample: at0713b_015301_fr15 Operator: Org Farm Kemi
Scan Range: 1 - 1117 Time Range: 0.00 - 11.16 min. Date: 2011-03-30 14:50
Sample Notes: Routine

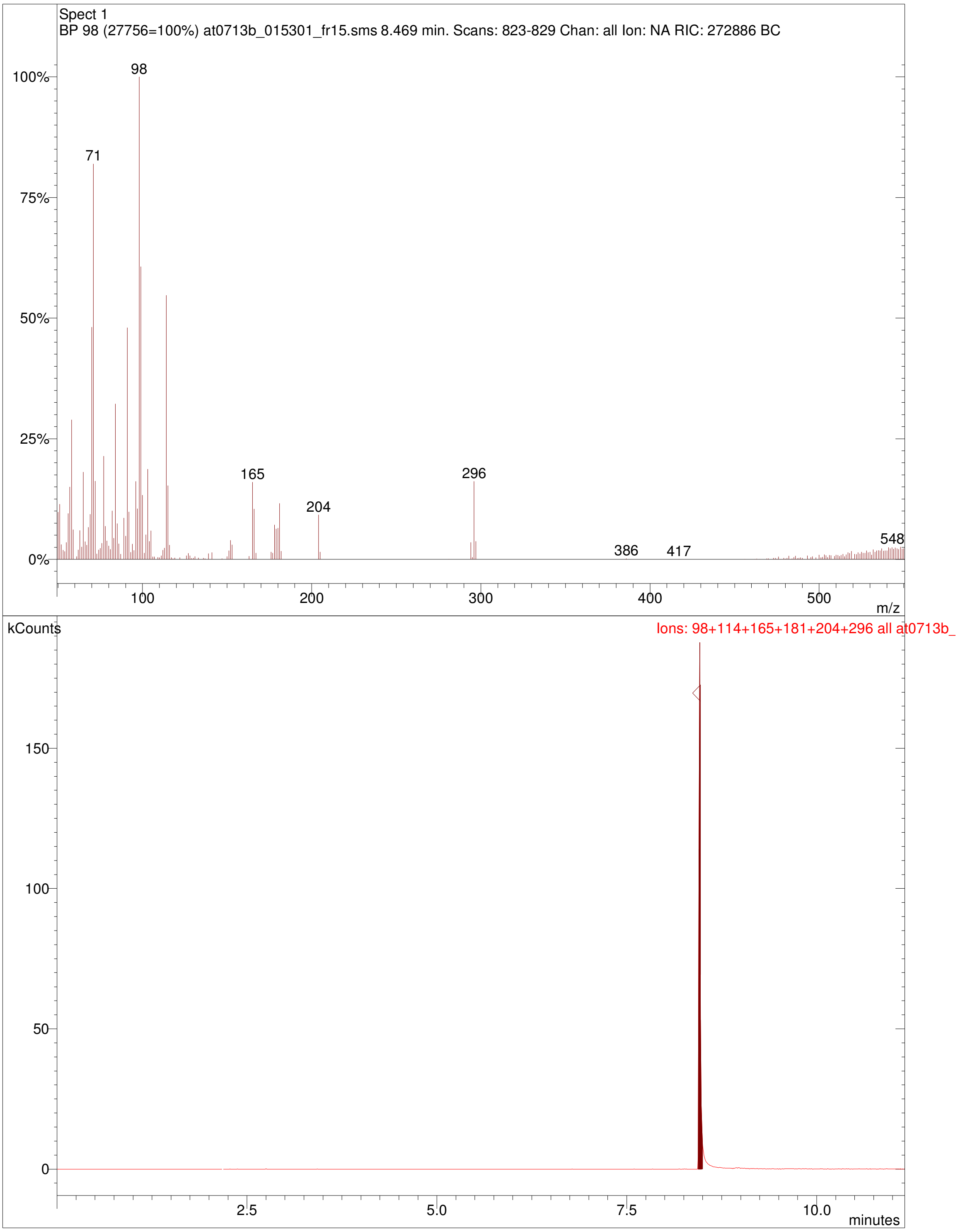

(3S)-3-(1,2-Di-p-tolylethoxy)-1-methylpiperidine, product 6e

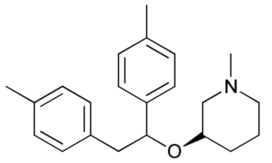

As described in the general procedure for synthesis of diarylated products **5**, **6** and **7(rac)**, but using *p*-tolylboronic acid (**4e**) as the arylating agent. The reaction was stirred for 36 hours and purification by DCVC afforded **6e** in 63% yield (43 mg) as a bright-yellow oil; 3.0:1 d.r.; R_f=0.3 (*i*-hexane/EtOAc/Et_3N 70:26:4); $[\alpha]^{22}_{D}$= 7.1 (c=13.0 in $CHCl_3$); ^1H NMR ($CDCl_3$, major isomer, 20°C, TMS): δ=7.16 (d, *J*=8.1 Hz, 2H), 7.11 (d, *J*=8.0 Hz, 2H), 7.06-7.00 (m, 4H), 4.50 (dd, *J*=5.5, 7.9 Hz, 1H), 3.32-3.24 (m, 1H), 3.04 (dd, *J*=7.8, 13.7 Hz, 1H), 2.82 (dd, *J*=5.6, 13.7 Hz, 1H), 2.66-2.46 (m, 2H), 2.34 (s, 3H), 2.31 (s, 3H), 2.19 (s, 3H), 1.99-1.81 (m, 2H), 1.80-1.57 (m, 2H), 1.43-1.30 (m, 1H), 1.13-1.02 (m, 1H); ^{13}C NMR ($CDCl_3$, major isomer, 20°C, TMS): δ=139.9, 136.9, 135.8, 135.3, 129.4, 128.9, 128.6, 126.6, 81.2, 72.7, 61.2, 55.4, 55.4, 46.3, 44.8, 29.0, 23.1, 21.0; ^{13}C NMR ($CDCl_3$, minor isomer, 20°C, TMS): δ=140.1, 136.9, 135.8, 135.4, 129.4, 128.8, 128.6, 126.6, 81.8, 73.3, 60.0, 55.4, 46.3, 44.9, 30.9, 29.0, 23.5, 21.1; MS (70 eV): *m/z* (%): 324 (100) [M^+], 114 (25) [$C_6H_{12}NO^+$], 98 (60) [$C_6H_{12}N^+$]; HRMS (ESI): *m/z* calcd for $C_{22}H_{29}NO$ ($M + H^+$): 324.2327; Found: 324.2330.

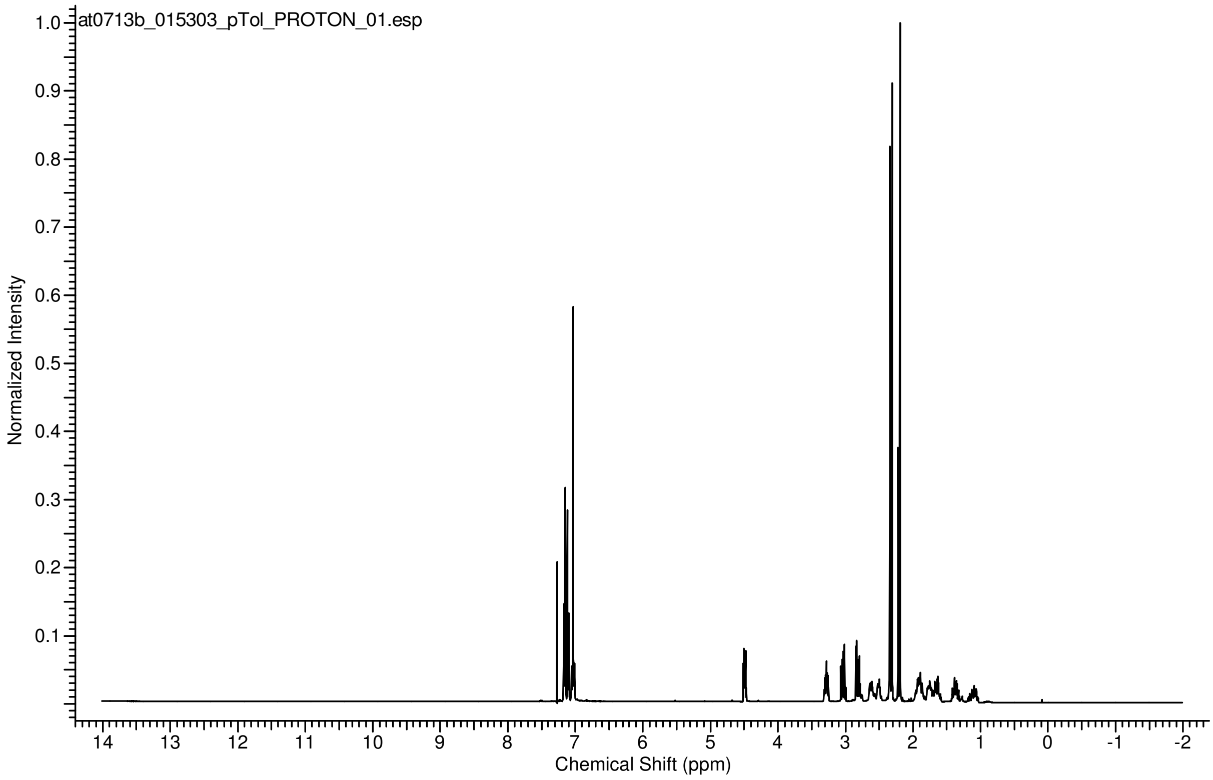
at0713b_015303_pTol_PROTON_01.esp
Normalized Intensity
Chemical Shift (ppm)

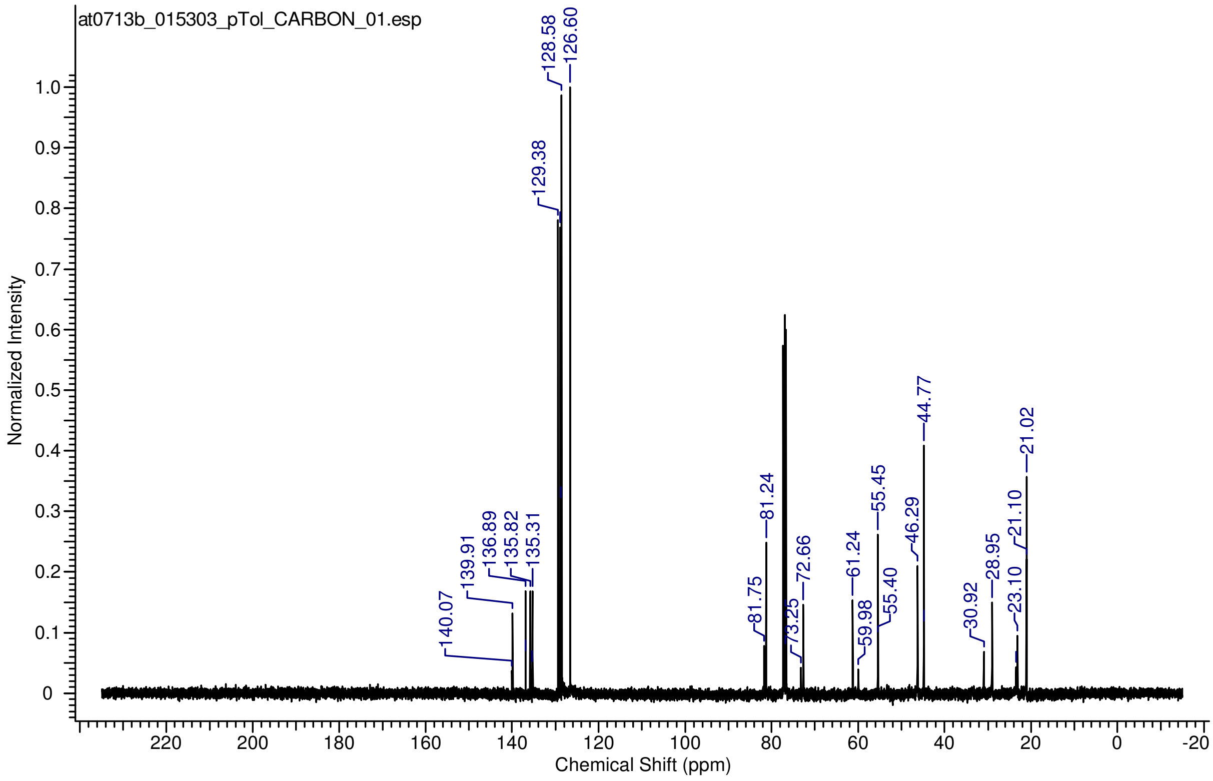
at0713b_015303_pTol_CARBON_01.esp
128.58
126.60
129.38
140.07
139.91
136.89
135.82
135.31
81.75
81.24
73.25
72.66
61.24
59.98
55.45
55.40
46.29
44.77
30.92
28.95
23.10
21.10
21.02
Normalized Intensity
Chemical Shift (ppm)

Chromatogram Plot

File: m:\... \pek\resterande gc_lc\at0713b_015303\at0713b_015303_fr24.sms
Sample: at0713b_015303_fr24 Operator: Operator
Scan Range: 1 - 1102 Time Range: 0.00 - 11.16 min. Date: 2011-04-26 13:17
Sample Notes: ROUTINE

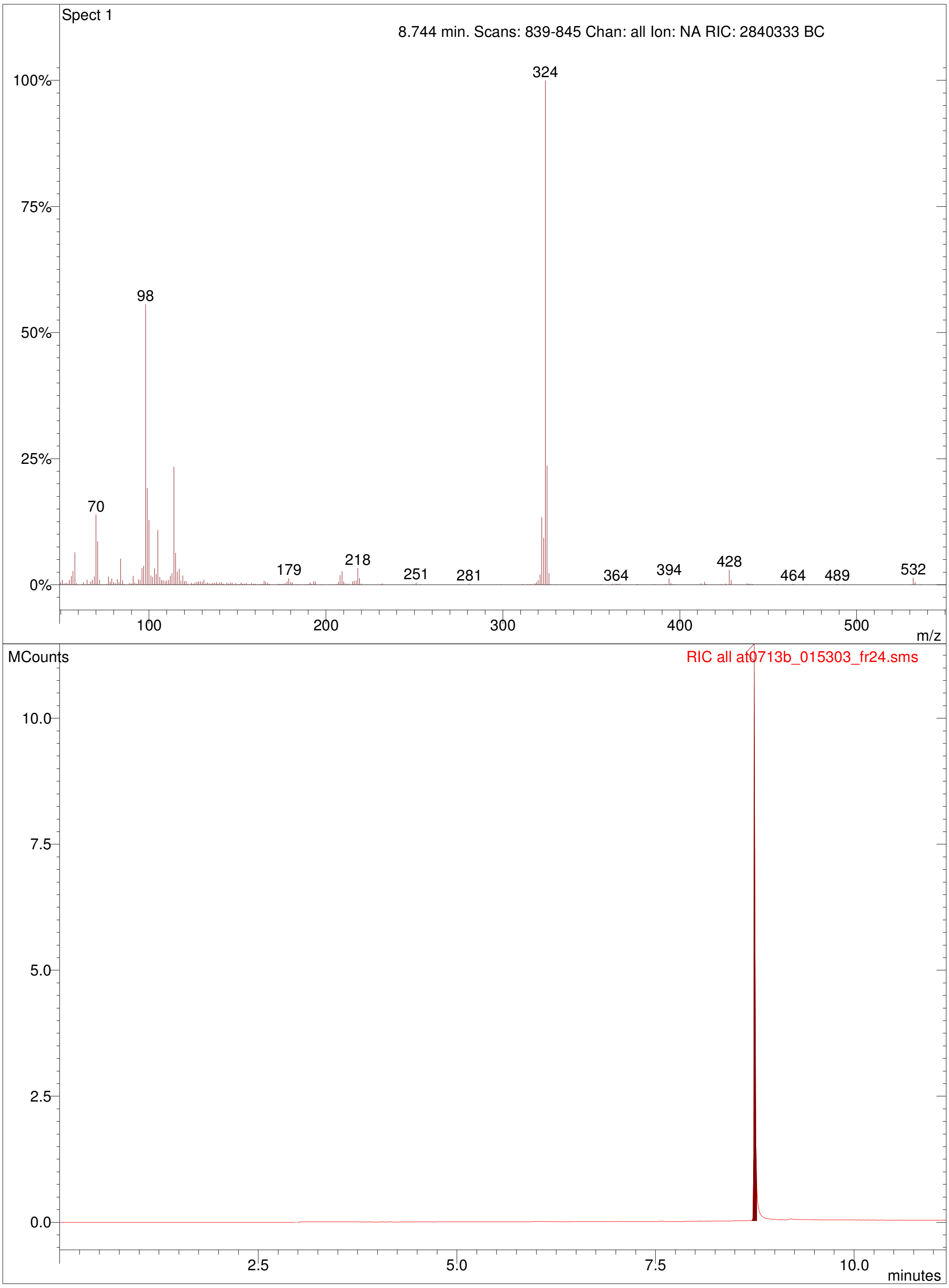

(3S)-3-(1,2-Di-m-tolylethoxy)-1-methylpiperidine, product 6f

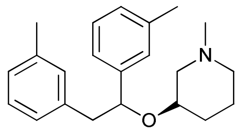

As described in the general procedure for synthesis of diarylated products **5**, **6** and **7(rac)**, but using *m*-tolylboronic acid (**4f**) as the arylating agent. The reaction was stirred for 36 hours and purification by DCVC afforded **6f** in 52% yield (36 mg) as a bright-yellow oil; 3.4:1 d.r. $[\alpha]_D^{22}$= -2.5 (c=13.8 in $CHCl_3$); R_f=0.3 (*i*-hexane/EtOAc/Et_3N 70:26:4); ^1H NMR ($CDCl_3$, major isomer, 20°C, TMS): δ=7.20 (dt, *J*=7.6 Hz, 1H), 7.14 (dt, *J*=7.5 Hz, 1H), 7.11-7.05 (m, 3H), 7.02-6.93 (m, 3H), 4.50 (dd, *J*=5.1, 8.2 Hz, 1H), 3.33-3.24 (m, 1H), 3.03 (dd, *J*=8.2 Hz, 13.7 Hz, 1H), 2.82 (dd, *J*=5.1, 13.7 Hz, 1H), 2.67-2.46 (m, 2H), 2.35 (s, 3H), 2.32 (s, 3H), 2.20 (s, 3H), 1.99-1.80 (m, 2H), 1.78-1.58 (m, 2H), 1.44-1.30 (m, 1H), .1.12-1.00 (m, 1H); ^{13}C NMR ($CDCl_3$, major isomer, 20°C, TMS): δ=143.0, 138.9, 137.7, 137.3, 130.4, 128.1, 128.1, 127.8, 127.2, 126.7, 126.5, 123.7, 81.5, 72.8, 61.1, 55.4, 46.2, 45.2, 28.8, 22.9, 21.4, 21.3; 324; MS (70 eV): *m/z* (%): 324 (47) [M^+], 114 (55) [$C_6H_{12}NO^+$], 98 (100) [$C_4H_9N^+$]; HRMS (ESI): *m/z* calcd for $C_{22}H_{29}NO$ ($M + H^+$): 324.2327; Found: 324.2326.

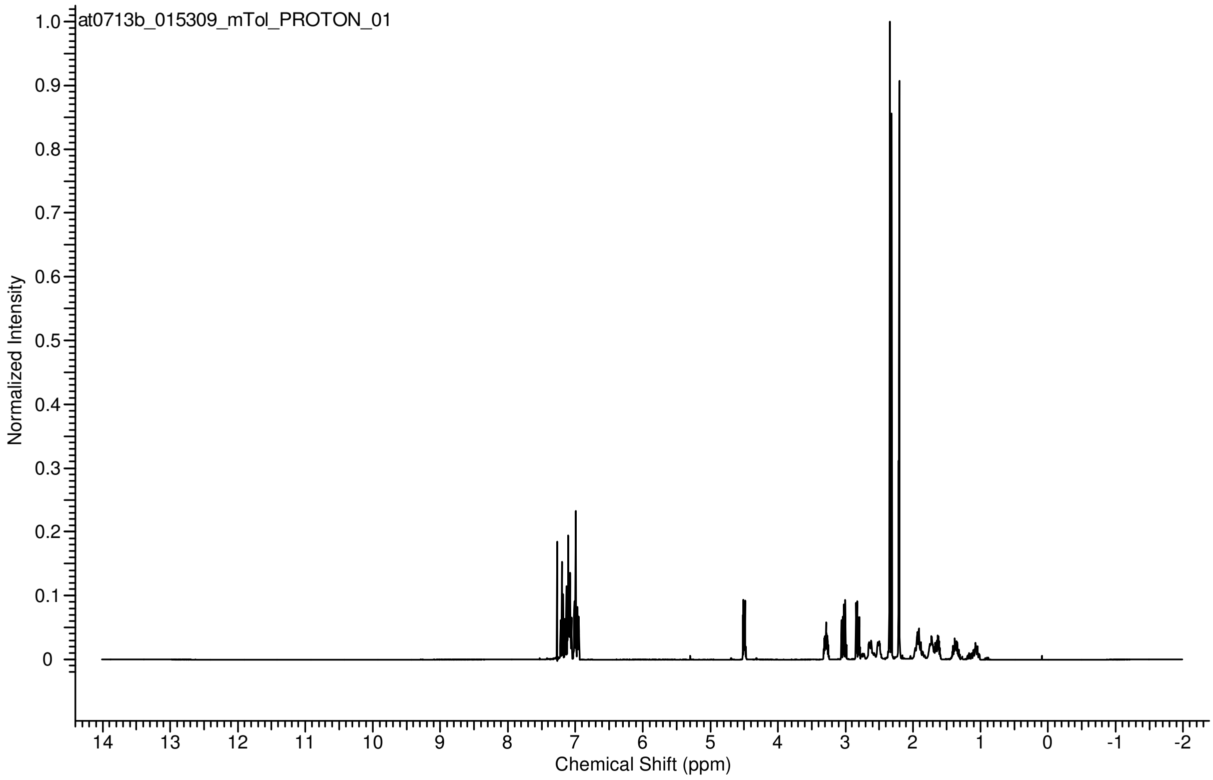
at0713b_015309_mTol_PROTON_01
Normalized Intensity
Chemical Shift (ppm)

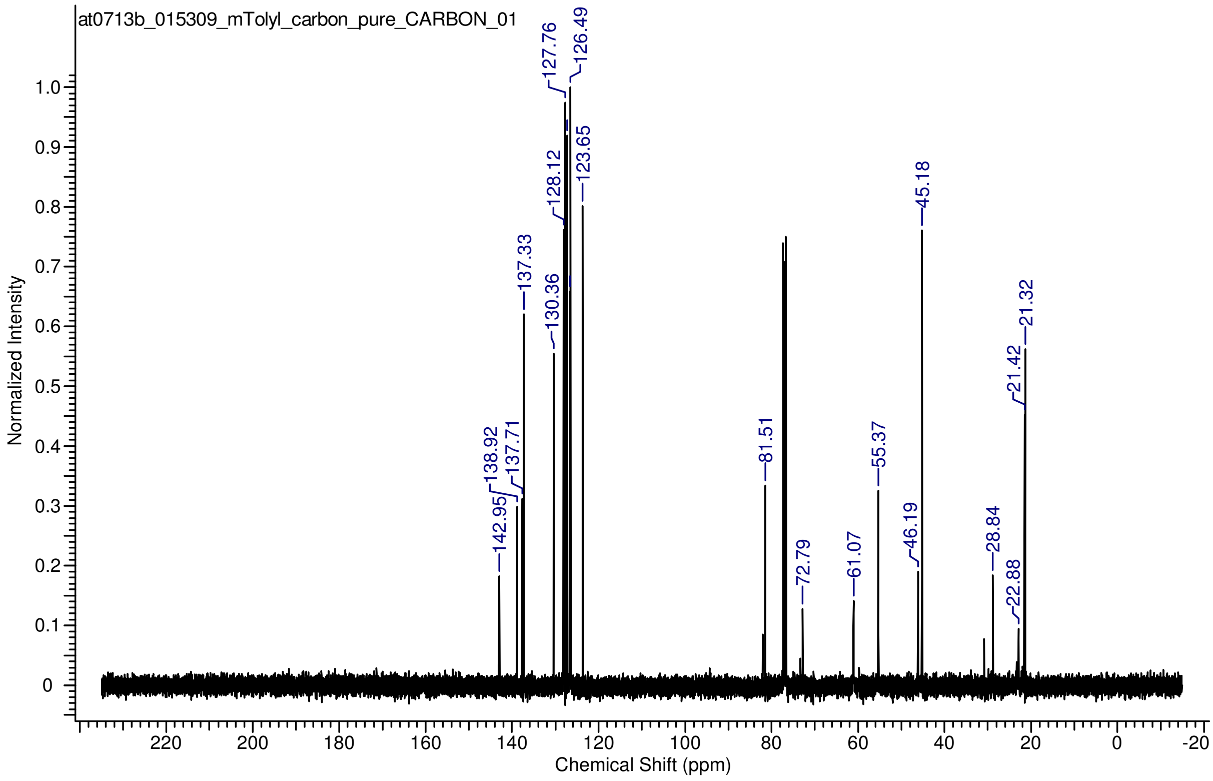
at0713b_015309_mTolyl_carbon_pure_CARBON_01
142.95
138.92
137.71
137.33
130.36
128.12
127.76
126.49
123.65
81.51
72.79
61.07
55.37
46.19
45.18
28.84
22.88
21.42
21.32
Normalized Intensity
Chemical Shift (ppm)

Chromatogram Plot

File: m:\... \pek\resterande gc_lc\at0713b_015309\at0713b_015309_fr23.sms
Sample: at0713b_015309_fr23 Operator: Operator
Scan Range: 1 - 1099 Time Range: 0.00 - 11.15 min. Date: 2011-04-22 09:49
Sample Notes: ROUTINE

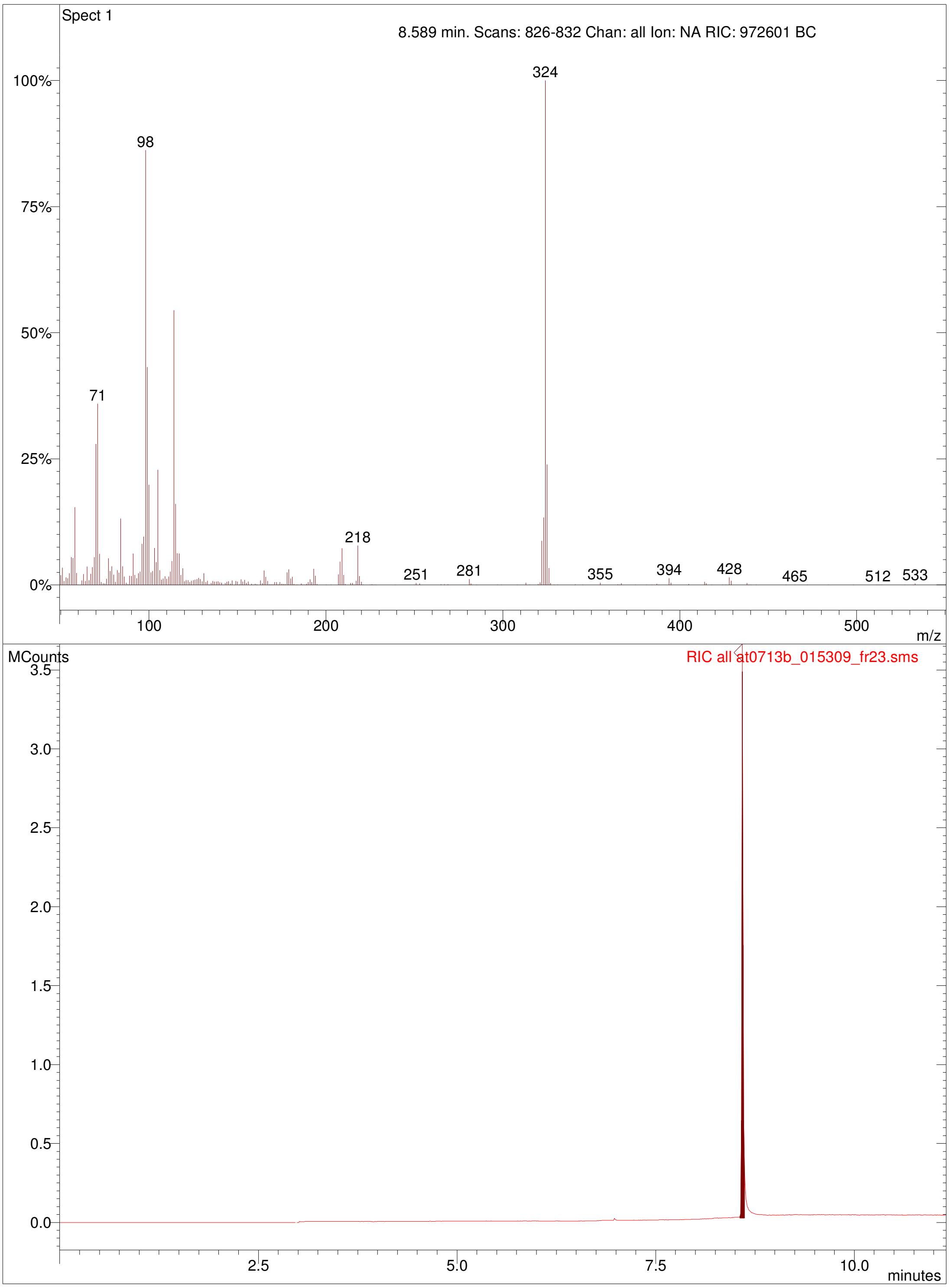

(3S)-3-(1,2-Di-o-tolylethoxy)-1-methylpiperidine, product 6g

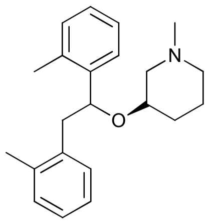

As described in the general procedure for synthesis of diarylated products **5**, **6** and **7(rac)**, but using *o*-tolylboronic acid (**4g**) as the arylating agent. The reaction was stirred for 36 hours and purification by DCVC afforded **6g** in 49% yield (33 mg) as a brown oil; 1.7:1 d.r.; R_f=0.3 (*i*-hexane/EtOAc/Et_3N 70:26:4); $[\alpha]_D^{22}$= 8.7 (c=16.1 in $CHCl_3$); ^1H NMR ($CDCl_3$, major isomer, 20°C, TMS): δ=7.51 (dd, *J*=1.7, 6.1 Hz, 1H), 7.26-7.19 (m, 1H), 7.14 (dt, *J*=1.5, 7.4 Hz, 1H), 7.12-7.09 (m,2H), 7.09-7.00 (m, 3H), 4.83 (dd, *J*=6.0, 7.4 Hz, 1H), 3.27-3.18 (m, 1H), 3.11 (dd, *J*=7.5, 13.7 Hz, 1H), 2.90-2.81 (m, 1H), 2.78-2.50 (m, 2H), 2.26 (s, 3H), 2.20 (s, 3H), 2.09 (s, 3H), 1.98-1.57 (m, 4H), 1.45-1.32 (m, 1H), 1.12-1.03 (m, 1H); ^{13}C NMR ($CDCl_3$, major isomer, 20°C, TMS): δ=141.2, 136.9, 136.7, 135.0, 130.4, 130.0 129.8, 127.0, 126.5, 126.1, 126.0, 125.5, 76.5, 72.8, 61.2, 55.4, 46.2, 41.6, 29.0, 23.2, 19.6, 18.9; ^1H NMR ($CDCl_3$, minor isome, 20°C, TMS): δ=7.53 (dd, *J*=1.7, 6.2 Hz, 1H), 7.26-7.19 (m, 1H), 7.15 (dt; *J*=1.6, 7.5 Hz, 1H), 7.12-7.09 (m,2H), 7.09-7.00 (m, 3H), 4.81 (dd, *J*=5.7, 7.3 Hz, 1H), 3.33-3.26 (m, 1H), 3.08 (dd, *J*=7.6, 13.7 Hz, 1H), 2.90-2.81 (m, 1H), 2.78-2.50 (m, 2H), 2.26 (s, 3H), 2.22 (s, 3H), 2.11 (s, 3H), 1.98-1.57 (m, 4H), 1.45-1.32 (m, 1H), 1.19-1.10 (m, 1H); ^{13}C NMR ($CDCl_3$, minor isomer, 20°C, TMS): δ=141.4, 136.9, 136.7, 135.0, 130.4, 130.0, 129.9, 126.9, 126.5, 126.1, 126.0, 125.5, 77.2, 73.4, 60.0, 55.3, 46.2, 41.6, 30.9, 23.5, 19.6, 18.9; MS (70 eV): *m/z* (%): 324 (83) [M^+], 114 (48) [$C_6H_{12}NO^+$], 98 (100) [$C_4H_9N^+$]; HRMS (ESI): *m/z* calcd for $C_{22}H_{29}NO$ ($M + H^+$): 324.2327; Found: 324.2324.

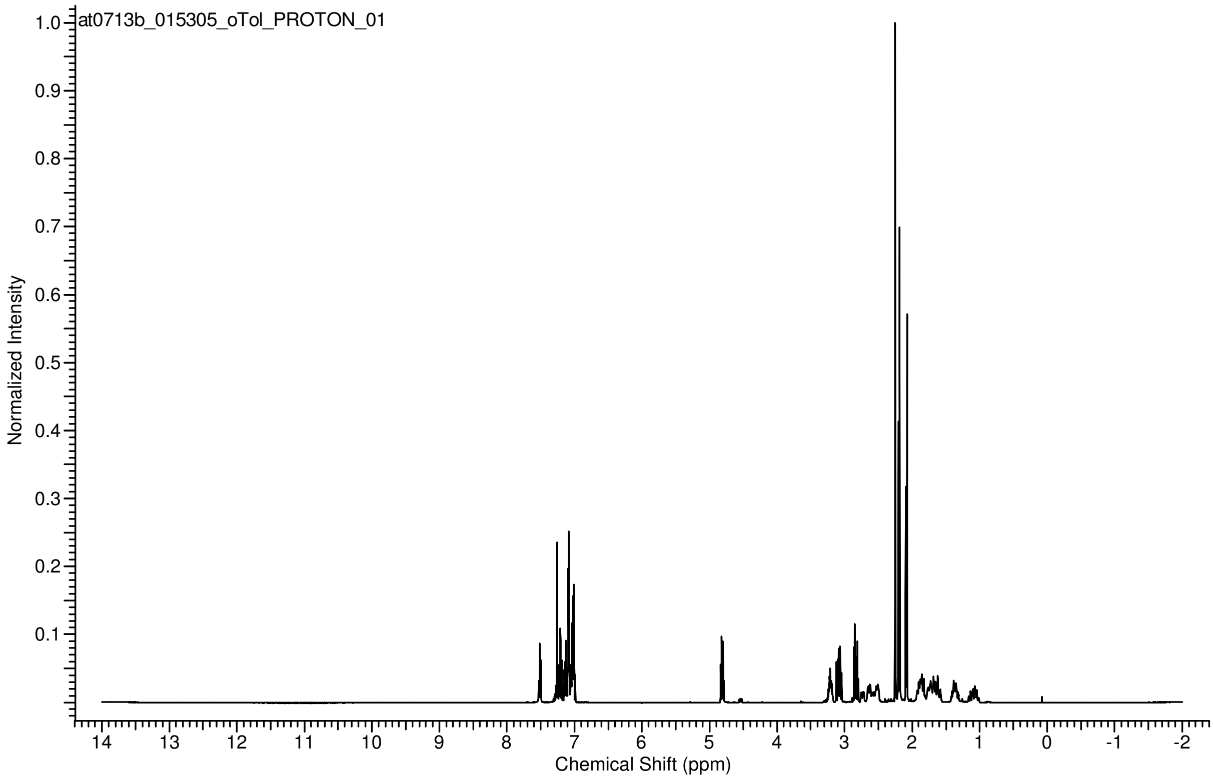
at0713b_015305_oTol_PROTON_01
Normalized Intensity
Chemical Shift (ppm)

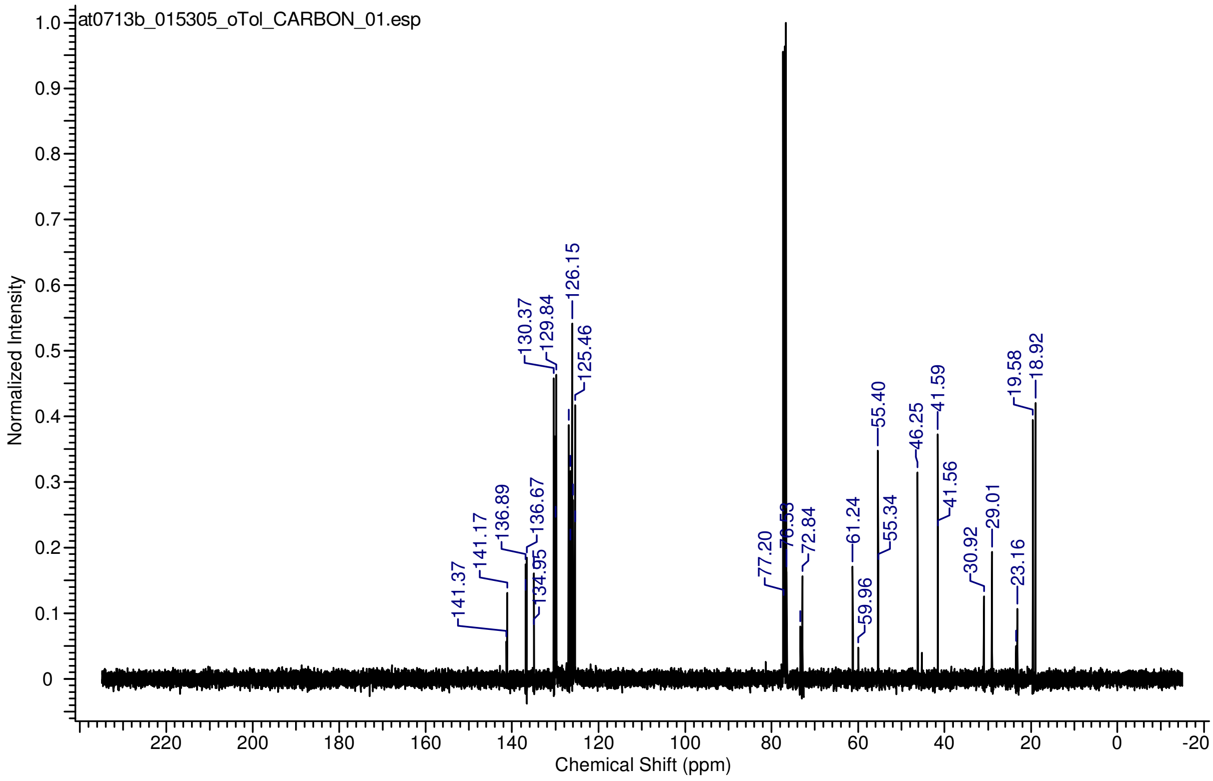
at0713b_015305_oTol_CARBON_01.esp
Normalized Intensity
Chemical Shift (ppm)
141.37
141.17
136.89
136.67
134.95
130.37
129.84
126.15
125.46
77.20
72.84
61.24
59.96
55.40
55.34
46.25
41.59
41.56
30.92
29.01
23.16
19.58
18.92

Chromatogram Plot

File: m:\... \pek\resterande gc_lc\at0713b_015305\st0713b_015305_fr23.sms
Sample: st0713b_015305_fr23 Operator: Operator
Scan Range: 1 - 1100 Time Range: 0.00 - 11.15 min. Date: 2011-04-25 11:29
Sample Notes: ROUTINE

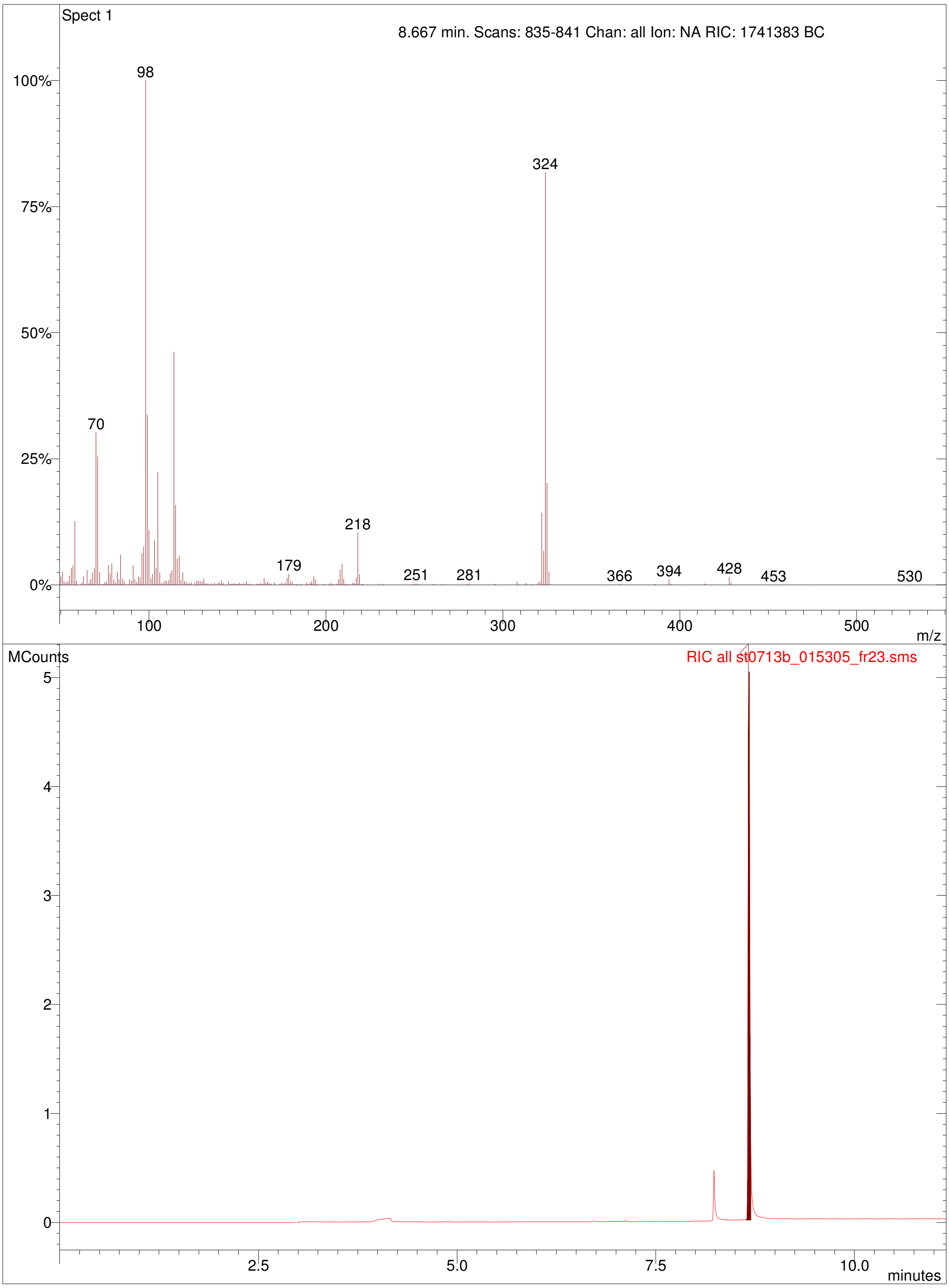

(3S)-3-(1,2-Di(naphthalen-2-yl)ethoxy)-1-methylpiperidine, product 6h

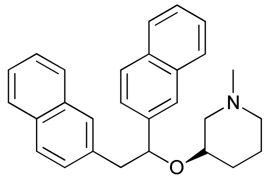

As described in the general procedure for synthesis of diarylated products **5**, **6** and **7(rac)**, but using 2-naphthaleneboronic acid (**4h**) as the arylating agent. The reaction was stirred for 36 hours and purification by DCVC afforded **6h** in 30% yield (25 mg) as a pale white paste; 4.7:1 d.r.; R_f=0.4 (*i*-hexane/EtOAc/Et_3N 70:26:4); $[\alpha]^{22}_{D}$= 15.6 (c=4.2 in $CHCl_3$); ^{1}H NMR ($CDCl_3$, major isomer, 20°C, TMS): δ=.78-7.61 (m, 7H), 7.57 (s, 1H), 7.43 (dd, *J*=1.6, 8.4 Hz, 1H), 7.41-7.33 (m, 4H), 7.23 (dd, *J*=1.7, 8.4 Hz, 1H), 4.73 (dd, *J*=5.5, 8.2 Hz, 1H), 3.29-3.20 (m, 2H), 3.04 (dd, *J*=5.4, 13.7 Hz, 1H), 2.57-2.50 (m, 1H), 2.41-2.33 (m, 1H), 2.07 (s, 3H), 1.89-1.79 (m, 2H), 1.71-1.63 (m, 1H), 1.59-1.50 (m, 1H), 1.29-1.17 (m, 1H), 1.10-1.00 (m, 1H); ^{13}C NMR ($CDCl_3$, major isomer, 20°C, TMS): δ=140.4, 136, 133.4, 133.2, 133.1, 132.1, 128.2, 128.1, 128.0, 127.9, 127.8, 127.6, 127.5, 127.4, 126.0, 125.8, 125.7, 125.6, 125.2, 124.6, 81.6, 73.2, 61.3, 55.4, 46.3, 45.3, 29.0, 23.1; (*M* + H^+): 396.2327; Found: 396.2333.

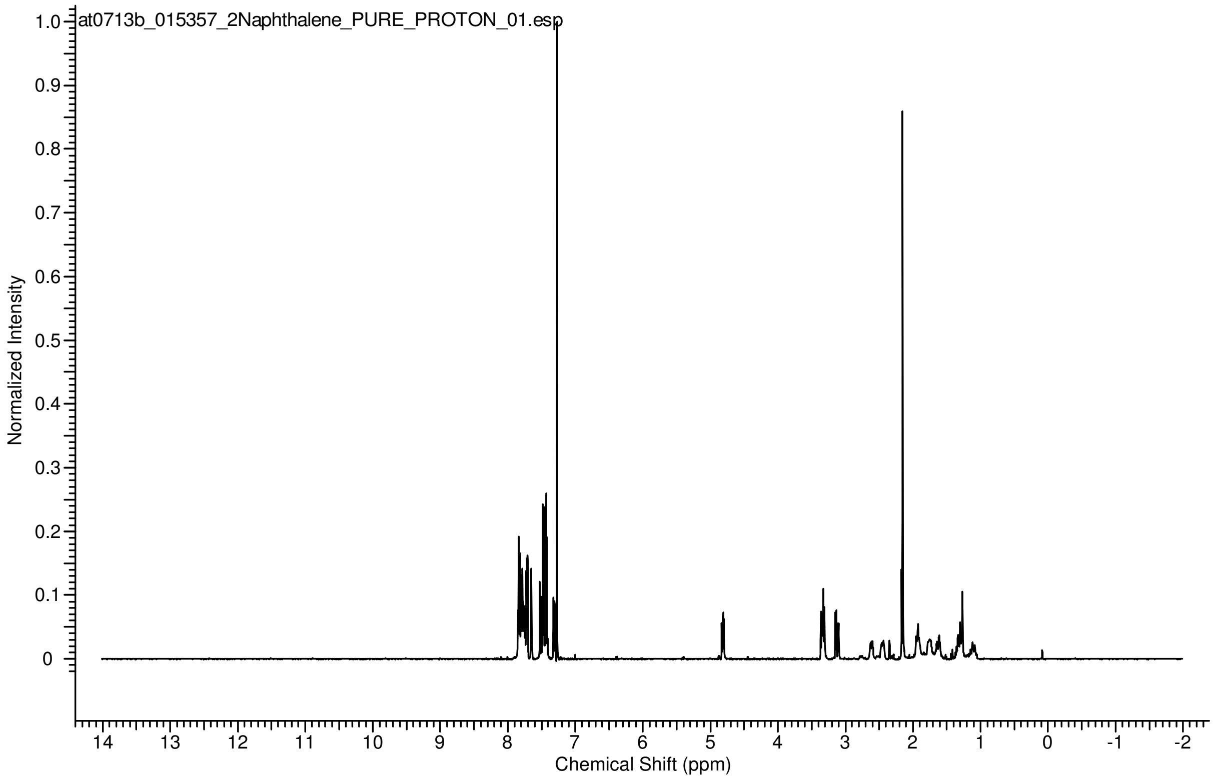
at0713b_015357_2Naphthalene_PURE_PROTON_01.esp
Normalized Intensity
Chemical Shift (ppm)

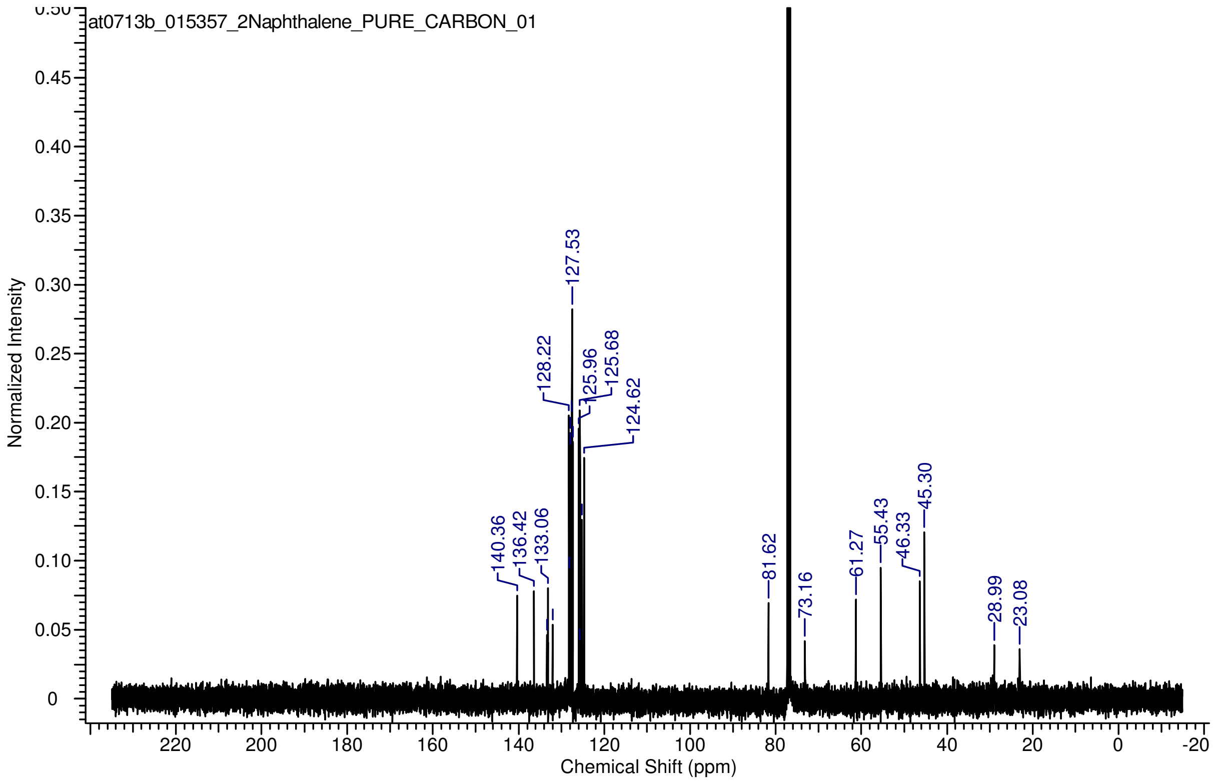
at0713b_015357_2Naphthalene_PURE_CARBON_01
Normalized Intensity
Chemical Shift (ppm)
140.36
136.42
133.06
128.22
127.53
125.96
125.68
124.62
81.62
73.16
61.27
55.43
46.33
45.30
28.99
23.08

Data File: z:\results\2011\v11-20\v19\v19.040\v19.gdt
Date acquired: Fri May 13 2011 12:44:53
Control Method: D:\BMC\METHODS\HIGHFL~1\POSITIVE\UV_H_P10.GCT
Sample name: at0713b_015357_2naph_fr14

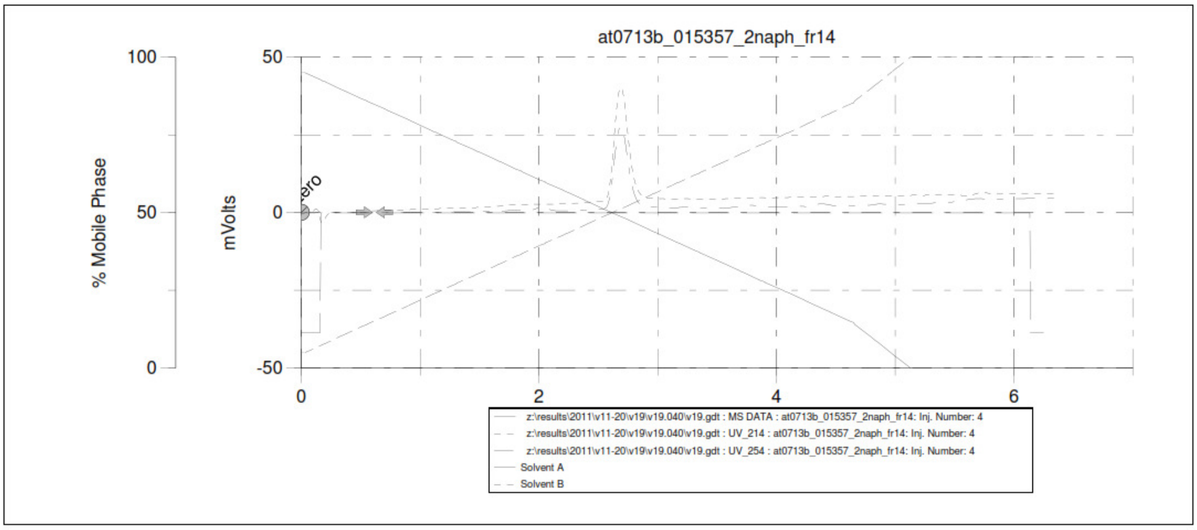

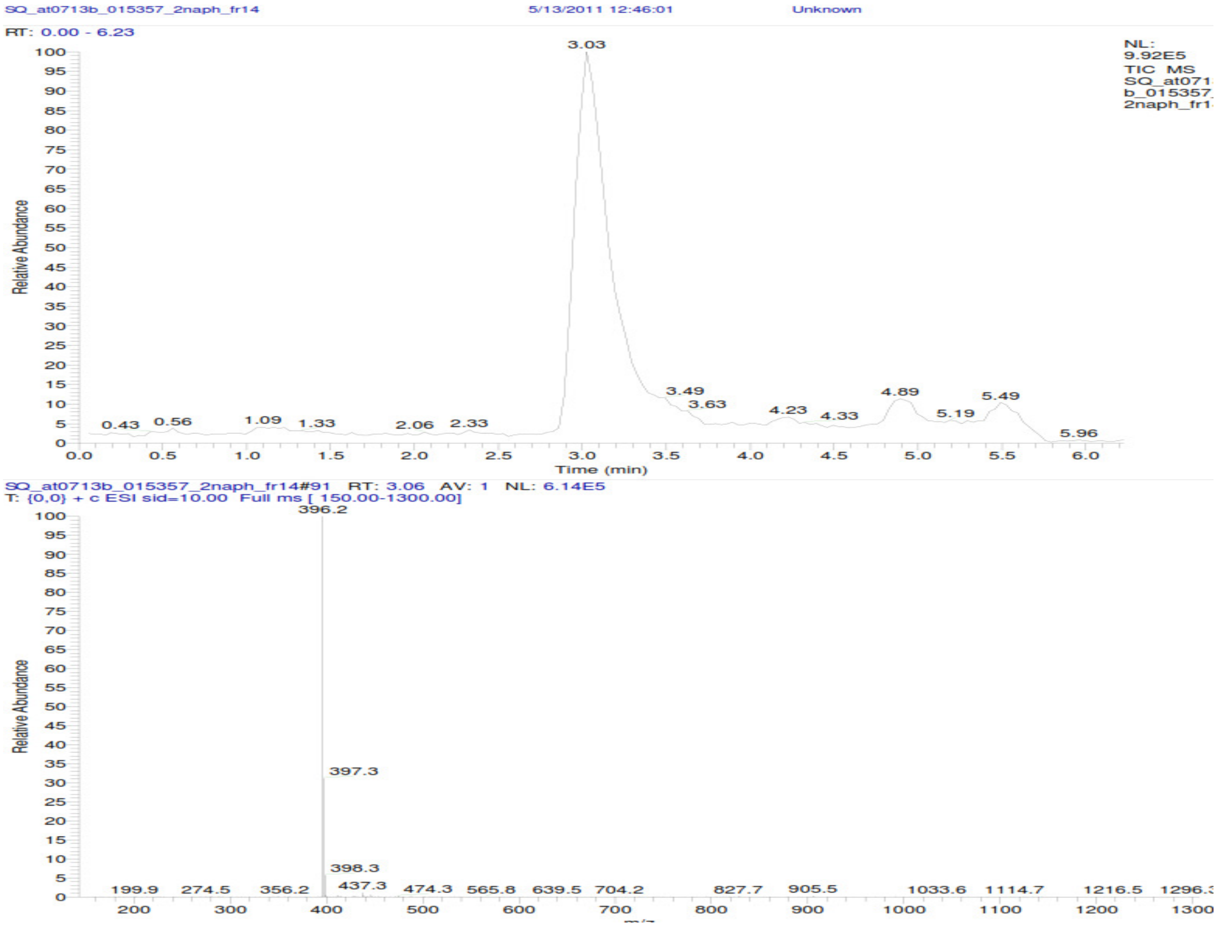

(3S)-3-(1,2-Bis(4-bromophenyl)ethoxy)-1-methylpiperidine, product 6i

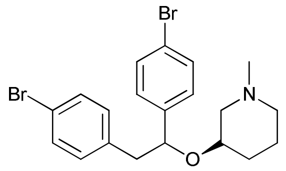

As described in the general procedure for synthesis of diarylated products **5**, **6** and **7(rac)**, but using 4-bromophenylboronic acid (**4i**) as the arylating agent. The reaction was stirred for 36 hours and purification by DCVC afforded **6i** in 35% yield (33 mg) as a pale-yellow oil; 3:1 d.r.; $[\alpha]^{22}_{D}$= 15.8 (c =9.2 in $CHCl_3$); R_f=0.3 (*i*-hexane/EtOAc/Et_3N 60:36:4); ^1H NMR ($CDCl_3$, major isomer, 20°C, TMS): δ=7.43 (d, *J*=8.4, 2H), 7.35 (d, *J*=8.4 Hz, 2H), 7.11 (d, *J*=8.4 Hz, 2H), 6.97 (d, *J*=8.4 Hz, 2H), 4.46 (dd, *J*=5.6, 7.6 Hz, 1H), 3.27-3.19 (m, 1H), 2.99 (dd, *J*=7.6, 13.7 Hz, 1H), 2.77 (dd, *J*=5.6 Hz, 1H), 2.63-2.46 (m, 2H), 2.18 (s, 3H), 197-1.82 (m, 2H), 1.78-1.59 (m, 2H), 1.42-1.29 (m, 1H), 1.12-1.01 (m, 1H); ^{13}C NMR ($CDCl_3$, major isomer, 20°C, TMS): δ=141.6, 137.2, 131.4, 131.3, 131.1, 128.3, 121.3, 120.1, 80.3, 73.2, 61.2, 55.4, 46.4, 44.5, 29.0, 23.1; HRMS (ESI): *m/z* calcd for $C_{20}H_{23}Br_2NO$ ($M + H^+$): 452.0225; Found: 452.0219.

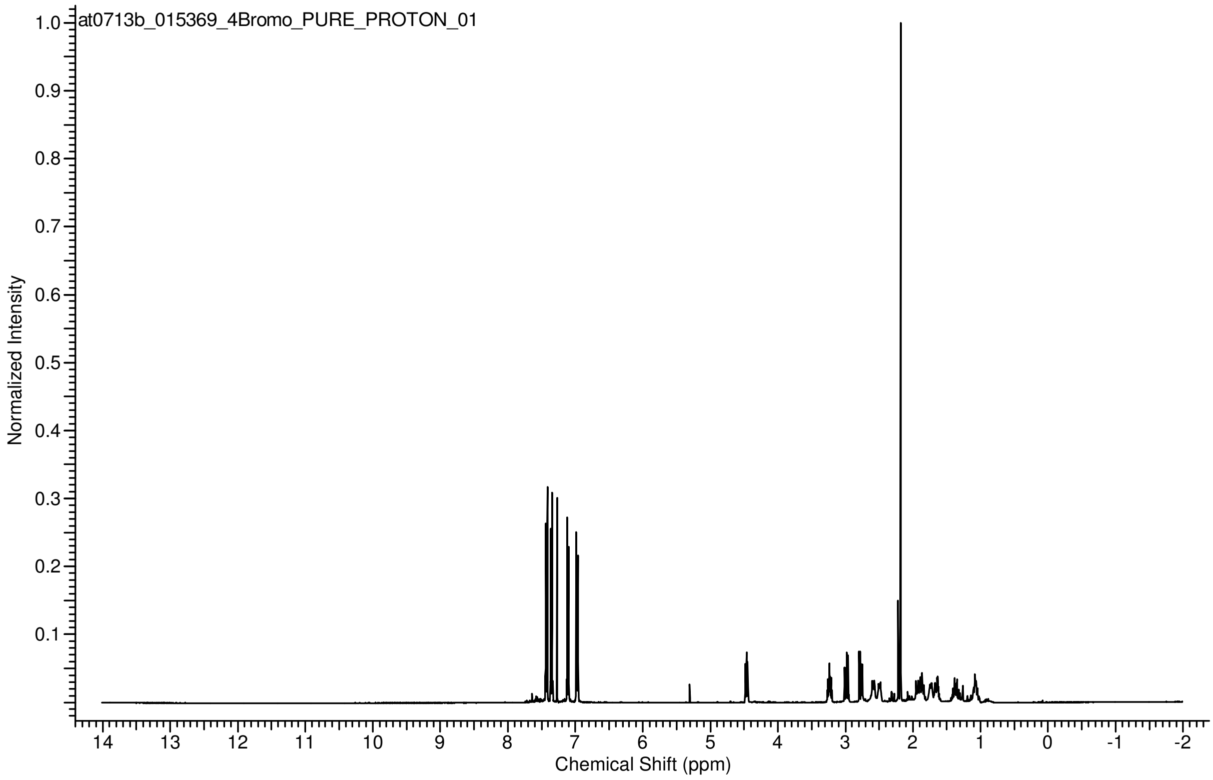
at0713b_015369_4Bromo_PURE_PROTON_01
Normalized Intensity
Chemical Shift (ppm)

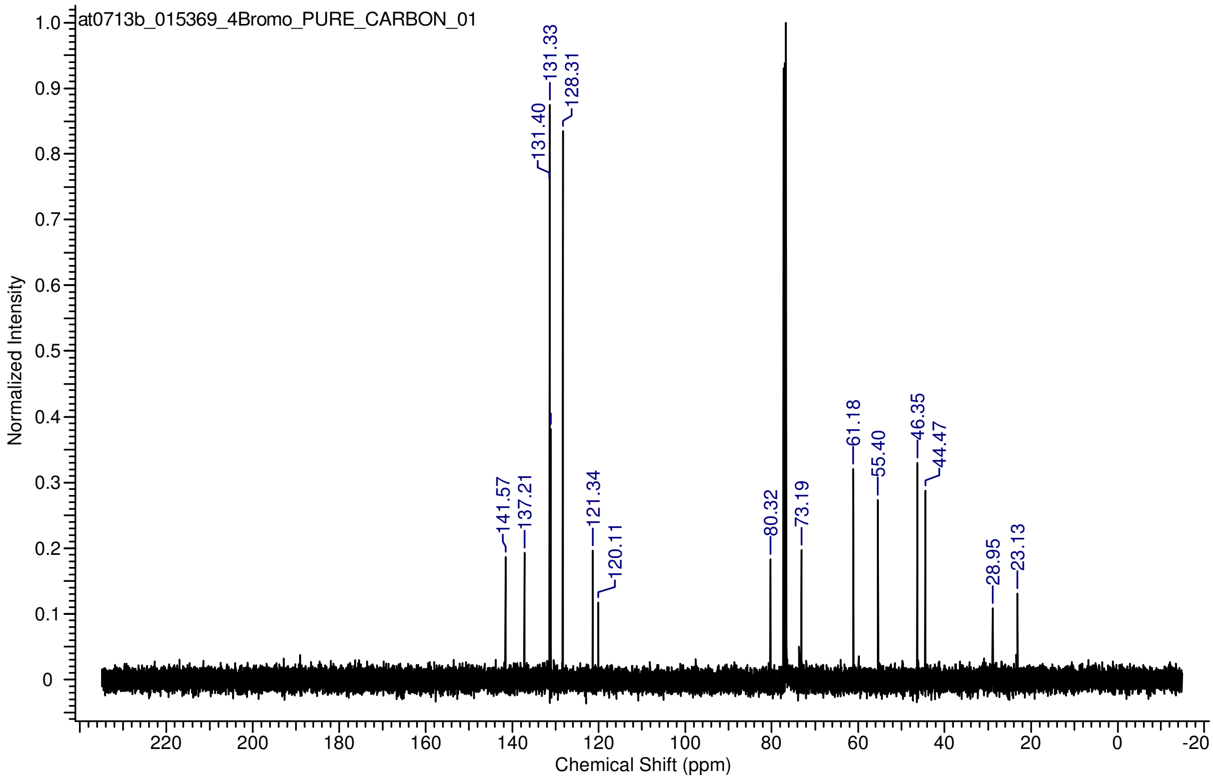
at0713b_015369_4Bromo_PURE_CARBON_01
131.33
128.31
131.40
141.57
137.21
121.34
120.11
80.32
73.19
61.18
55.40
46.35
44.47
28.95
23.13
Normalized Intensity
Chemical Shift (ppm)

Data File: z:\results\2011\v11-20\v19\v19.024\v19.gdt
Date acquired: Thu May 12 2011 12:43:39
Control Method: D:\BMC\METHODS\HIGHFL~1\POSITIVE\UV_H_P10.GCT
Sample name: at0713b_015369_4bromo_fr20

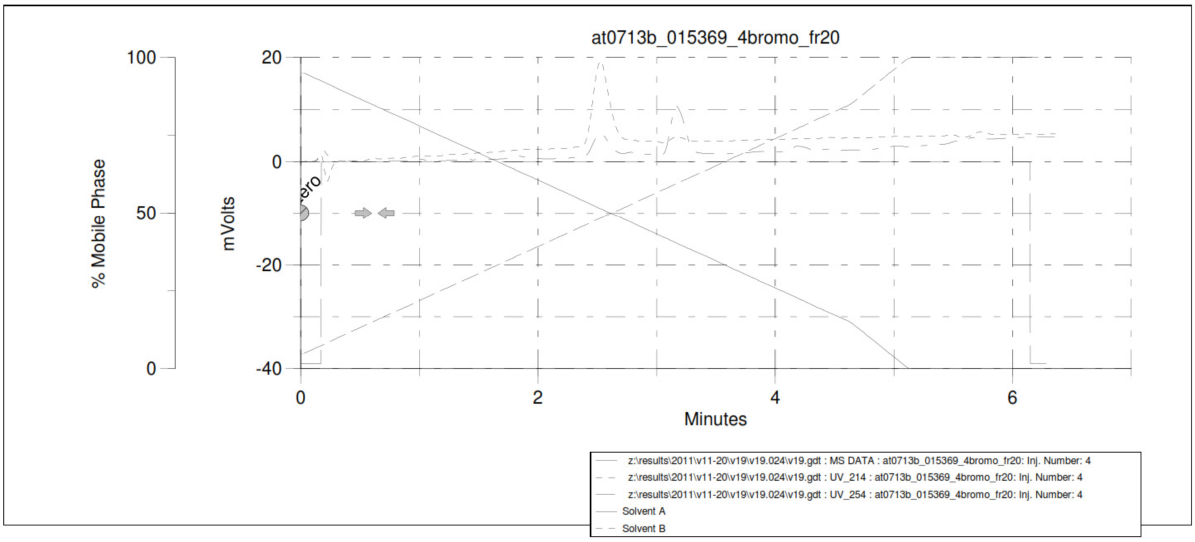

SQ_at0713b_015369_4bromo_fr20 5/12/2011 12:44:48 Unknown

RT: 0.00 - 6.23

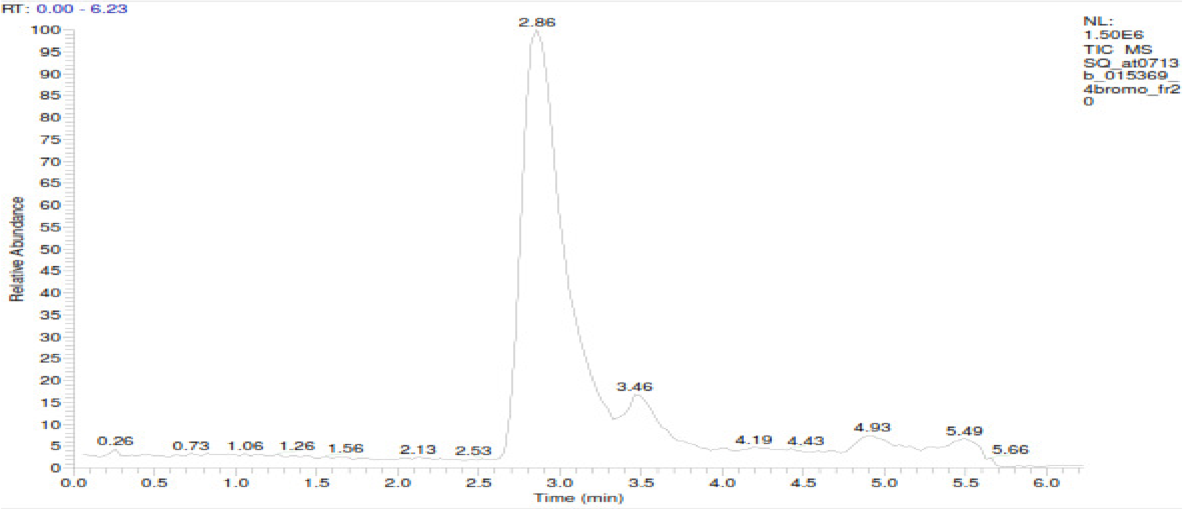

SQ_at0713b_015369_4bromo_fr20#86 RT: 2.89 AV: 1 NL: 5.22E5
T: {0,0} + c ESI sid=10.00 Full ms [150.00-1300.00]

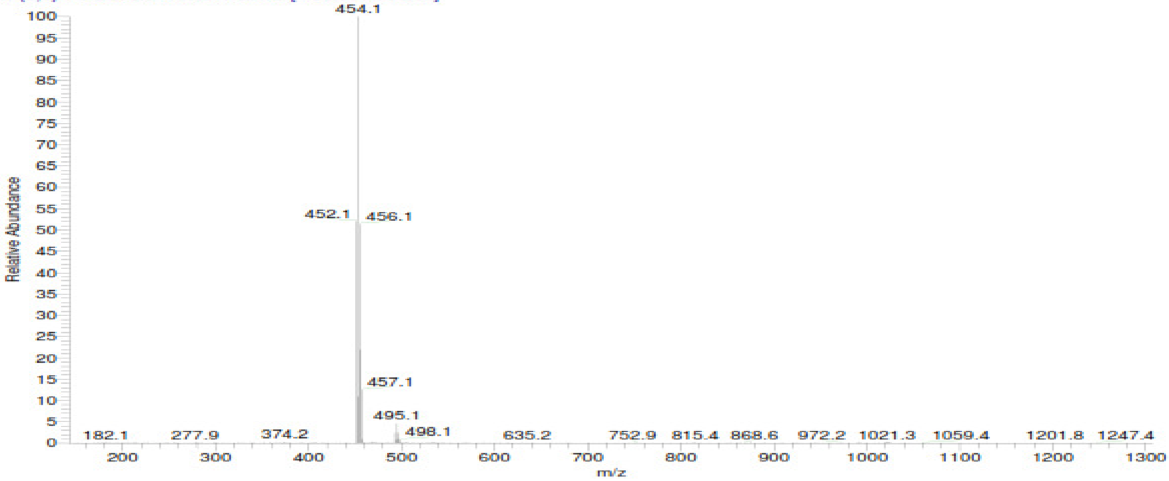

(3S)-3-(1,2-Bis(4-(trifluoromethyl)phenyl)ethoxy)-1-methylpiperidine, product 6k

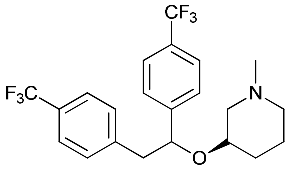

As described in the general procedure for synthesis of diarylated products **5**, **6** and **7(rac)**, but using 4-(trifluoromethyl)phenylboronic acid (**4k**) as the arylating agent. The reaction was stirred for 36 hours and purification by DCVC afforded **6k** 48% yield (44 mg) as a bright-yellow oil; 4.7:1 d.r.; R_f=0.3 (*i*-hexane/EtOAc/Et_3N 70:26:4); $[\alpha]_D^{22}$= -13.7 (c=13.2 in $CHCl_3$); ^{1}H NMR ($CDCl_3$, major isomer, 20°C, TMS): δ=7.58 (d, *J*=8.6 Hz, 2H), 7.51 (d, *J*=8.6 Hz, 2H), 7.39 (d, *J*=8.0 Hz, 2H), 7.26 (d, *J*=8.0 Hz, 2H), 4.62 (dd, *J*=5.0, 8.2 Hz, 1H), 3.30-3.22 (m, 1H), 3.10 (dd, *J*=8.2, 13.7 Hz, 1H), 2.91 (dd, *J*=5.0 Hz, 13.7 Hz, 1H), 2.66-2.48 (m, 2H), 2.21 (s, 3H), 2.03-1.87 (m, 2H), 1.78-1.60 (m, 2H), 1.44-1.32 (m, 1H), 1.11-0.99 (m, 1H); ^{13}C NMR ($CDCl_3$, major isomer, 20°C, TMS): δ=146.5, 142.2, 130.0 (q, *J*=32.7 Hz), 129.9, 128.7(q, *J*=32.3 Hz), 126.8, 125.3 (q, *J*=3.8 Hz), 124.9 (q, *J*=3.8 Hz), 121.6 (q, *J*=272.1 Hz), 121.4 (q, *J*=272.0 Hz), 80.3, 73.3, 60.9, 55.3, 46.2, 44.8, 28.8, 22.9; MS (70 eV): *m/z* (%): 432 (70) [M^+], 114 (100) [$C_6H_{12}NO^+$], 98 (72) [$C_4H_9N^+$]; HRMS (ESI): *m/z* calcd for $C_{22}H_{23}F_6NO$ ($M + H^+$): 432.1762; Found: 432.1761.

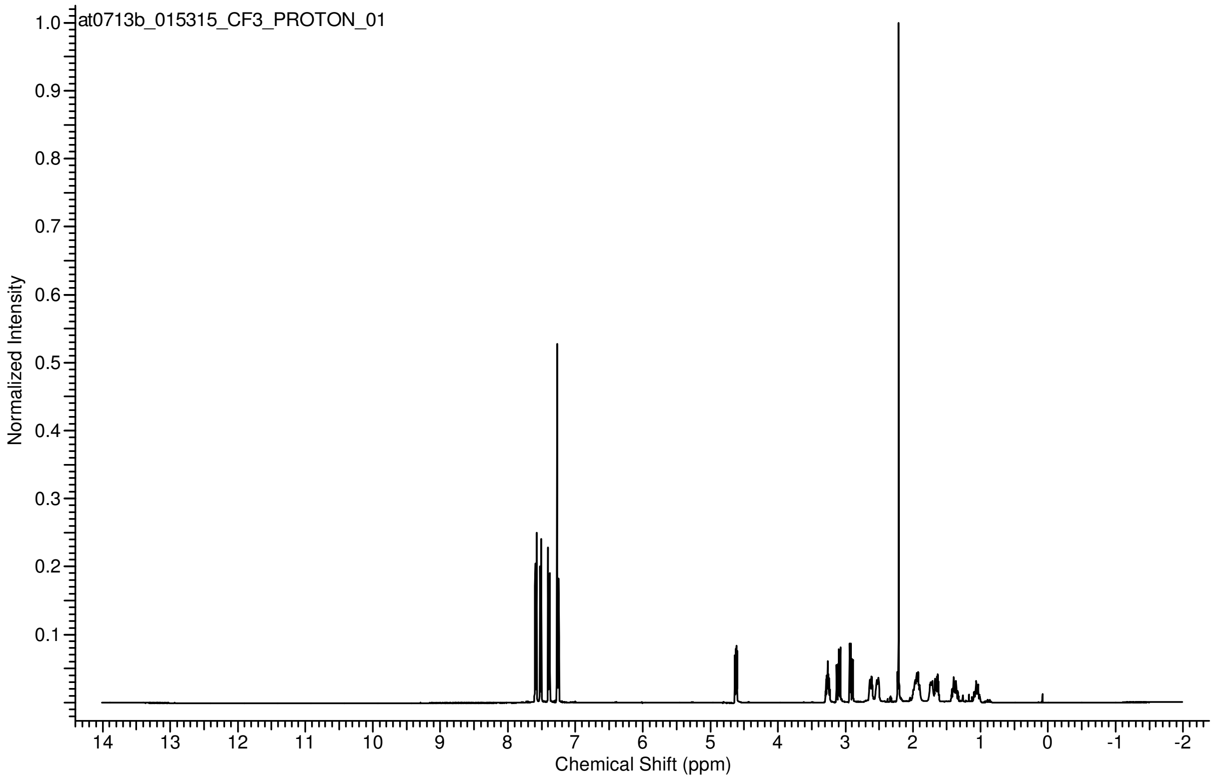
at0713b_015315_CF3_PROTON_01
Normalized Intensity
Chemical Shift (ppm)

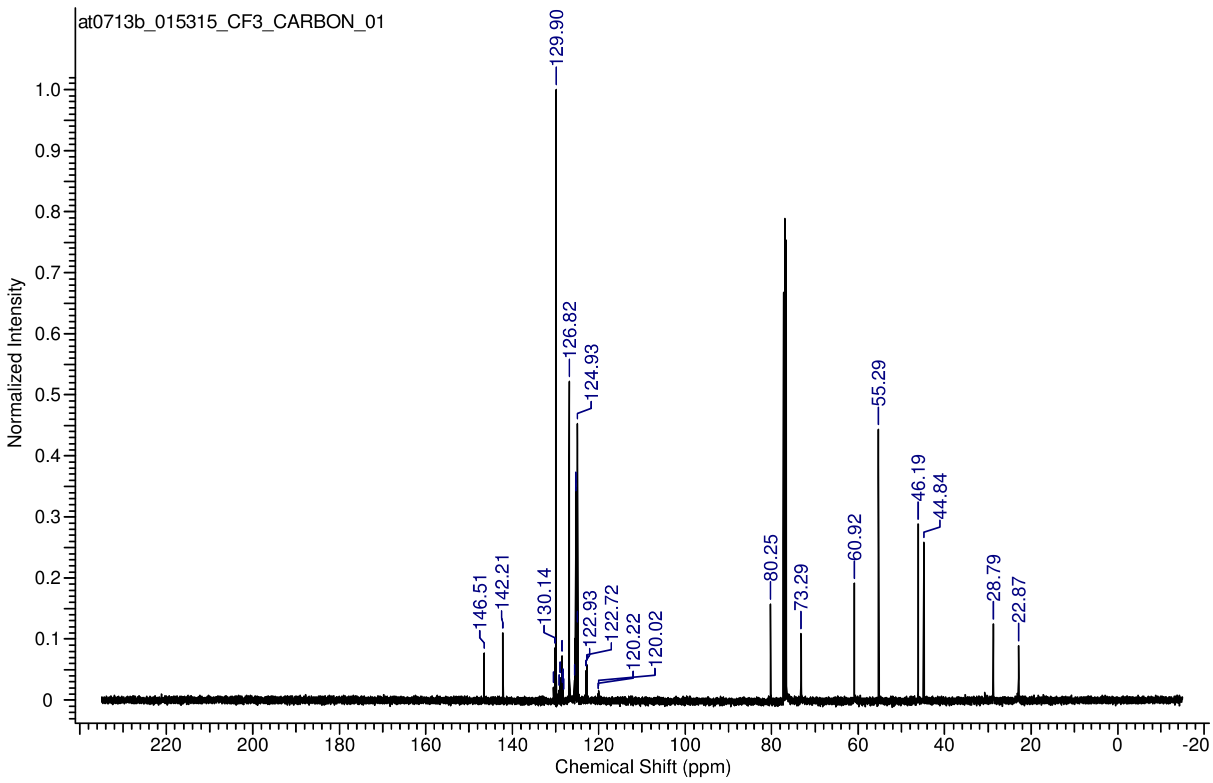
at0713b_015315_CF3_CARBON_01
129.90
126.82
124.93
146.51
142.21
130.14
122.93
122.72
120.22
120.02
80.25
73.29
60.92
55.29
46.19
44.84
28.79
22.87
Normalized Intensity
Chemical Shift (ppm)

Chromatogram Plot

File: m:\... \pek\resterande gc_lc\at0713bpcf_015315\at0713bpcf_015315_c.sms
Sample: at0713bpCF_015315_c Operator: Operator
Scan Range: 1 - 1266 Time Range: 0.00 - 11.99 min. Date: 2011-03-10 12:25
Sample Notes: ROUTINE

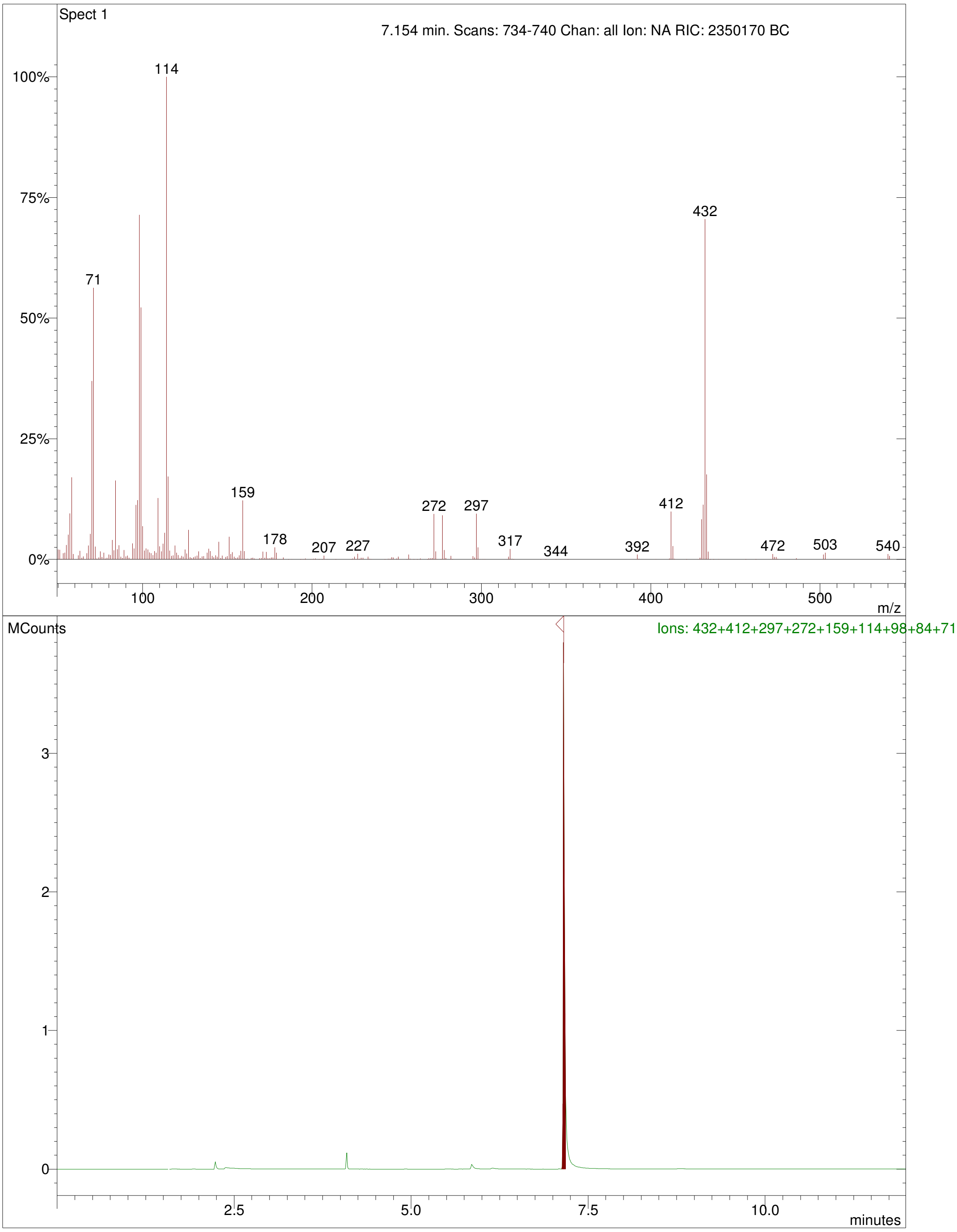

trans-2-(1,2-Diphenylethoxy)-N,N-dimethylcyclohexanamine, product 7(rac)

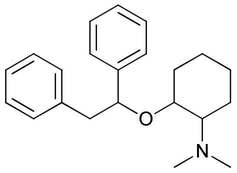

As described in the general procedure for synthesis of diarylated products **5**, **6** and **7(rac)**, but using phenylboronic acid (**4c**) as the arylating agent. The reaction was stirred for 36 hours and purification by DCVC afforded **7(rac)** in 40% yield (23 mg) as a bright-yellow oil; 33% d.r. The diastereoisomers were separated by preparative TLC (isohexane:diethyl ether:triethylamine 60:36:4);

Major Isomer 26% yield (15 mg); R_f=0.3 (*i*-hexane/EtOAc/Et_3N 60:36:4); $[\alpha]_D^{22}$= -0.6 (c=3.2 in $CHCl_3$); ^1H NMR ($CDCl_3$, major isomer, 20°C, TMS): δ= 7.38-7.13 (m, 10H), 4.59 (dd, *J*=5.6, 8.0 Hz, 3.21-3.16 (m, 1H), 3.15 (dd, *J*=8.0, 13.5 Hz, 1H), 2.91 (dd, *J*=5.6, 13.5 Hz, 1H), 2.36-2.19 (m, 2H), 2.17 (s, 6H), 1.94-1.87 (m, 1H), 1.77-1.70 (m, 1H), 1.61-1.52 (m, 1H), 1.50-1.43 (m, 1H), 1.20-1.10 (m, 2H), 1.05-0.95 (m, 2H); ^{13}C NMR ($CDCl_3$, major isomer, 20°C, TMS): δ=142.3, 139.1, 129.6, 128.0, 127.8, 127.4, 127.4, 125.9, 79.4, 74.2, 66.7, 45.1, 41.5, 28.9, 24.7, 24.0, 23.4; MS (70 eV): *m/z* (%): 324 (20) [M^+], 142 (100) [$C_8H_{16}NO^+$], 58 (15) [$C_3H_8N^+$]; HRMS (ESI): *m/z* calcd for $C_{22}H_{29}NO$ (M + H^+): 324.237; Found: 324.2328.

Minor isomer: 13% yield (7.5 mg); R_f=0.4 (*i*-hexane/EtOAc/Et_3N 60:36:4); $[\alpha]_D^{22}$= 0.6 (c=7.3 in $CHCl_3$); ^1H NMR ($CDCl_3$, 20°C, TMS): δ=7.31-7.19 (m, 7H), 7.17-7.12 (m, 3H), 4.90 (dd, *J*=6.2, 7.5 Hz, 1H), 3.23 (ddd, *J*=4.3, 8.3, 9.2 Hz, 1H), 3.16 (dd, *J*=7.5, 13.6 Hz, 1H), 2.85 (dd, *J*=6.2, 13.6 Hz, 1H), 2.33-2.26 (m, 1H), 2.23 (s, 6H), 1.80-1.63 (m, 3H), 1.60-1.49 (m, 1H), 1.24-0.95 (m, 4H); ^{13}C NMR ($CDCl_3$, 20°C, TMS): δ=144.0, 138.9, 129.6, 128.0, 127.9, 127.1, 126.8, 125.8, 82.4, 75.6, 67.5, 45.6, 41.4, 32.3, 30.3, 24.0, 23.5; MS (70 eV): *m/z* (%): 324 (20) [M^+], 142 (100) [$C_8H_{16}NO^+$], 58 (15) [$C_3H_8N^+$]; HRMS (ESI): *m/z* calcd for $C_{22}H_{29}NO$ (M + H^+): 324.2327; Found: 324.3232.

MAJOR
DIASTEREOMER

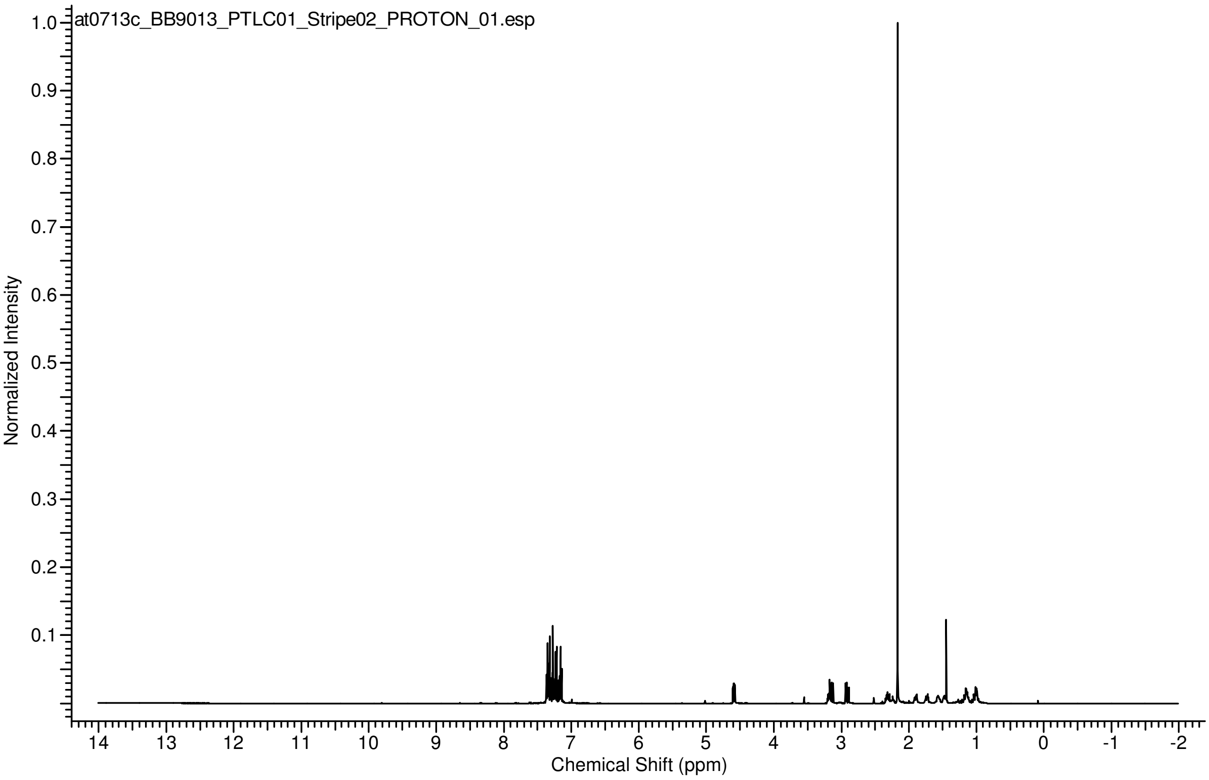

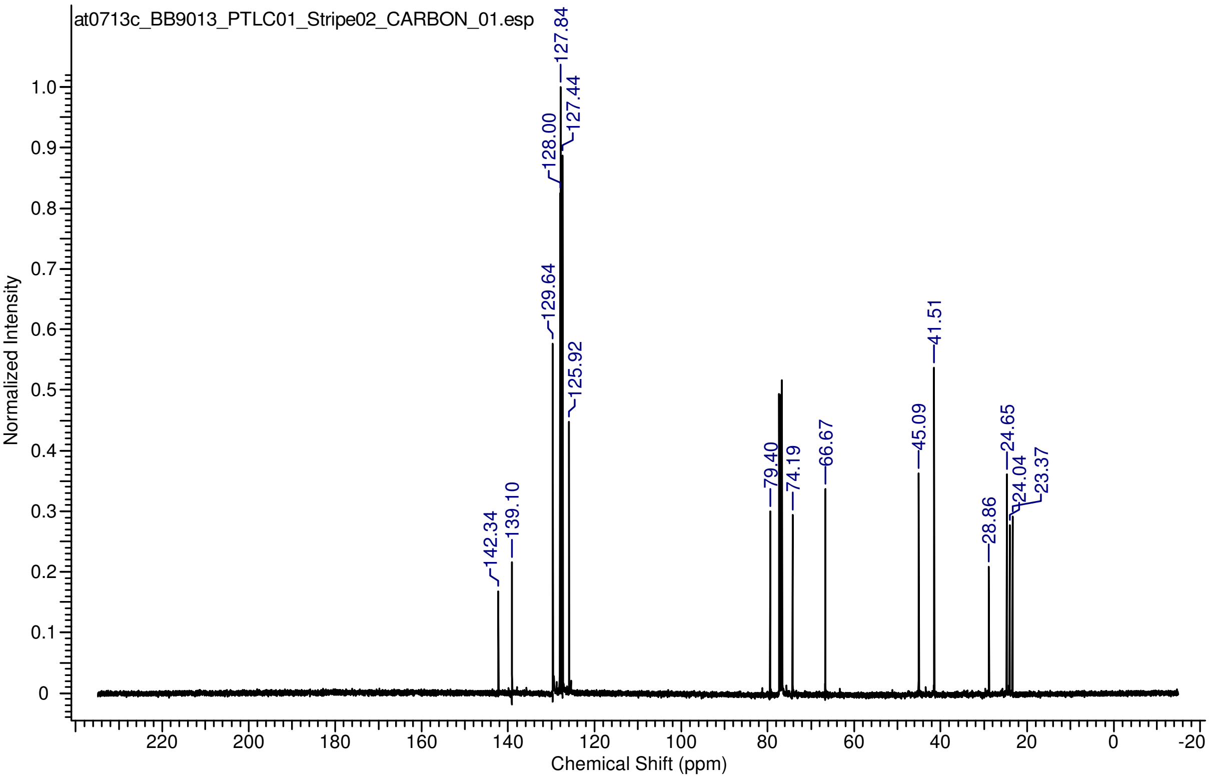

MINOR
DIASTEREOMER

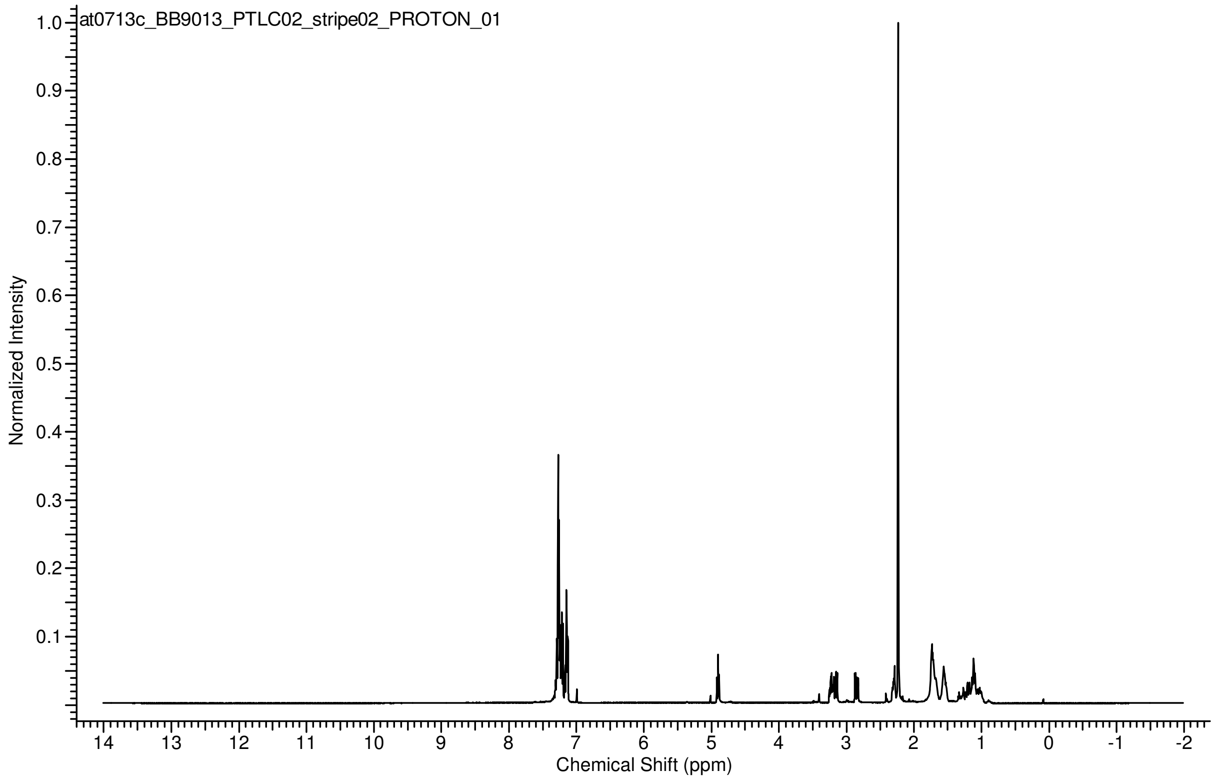

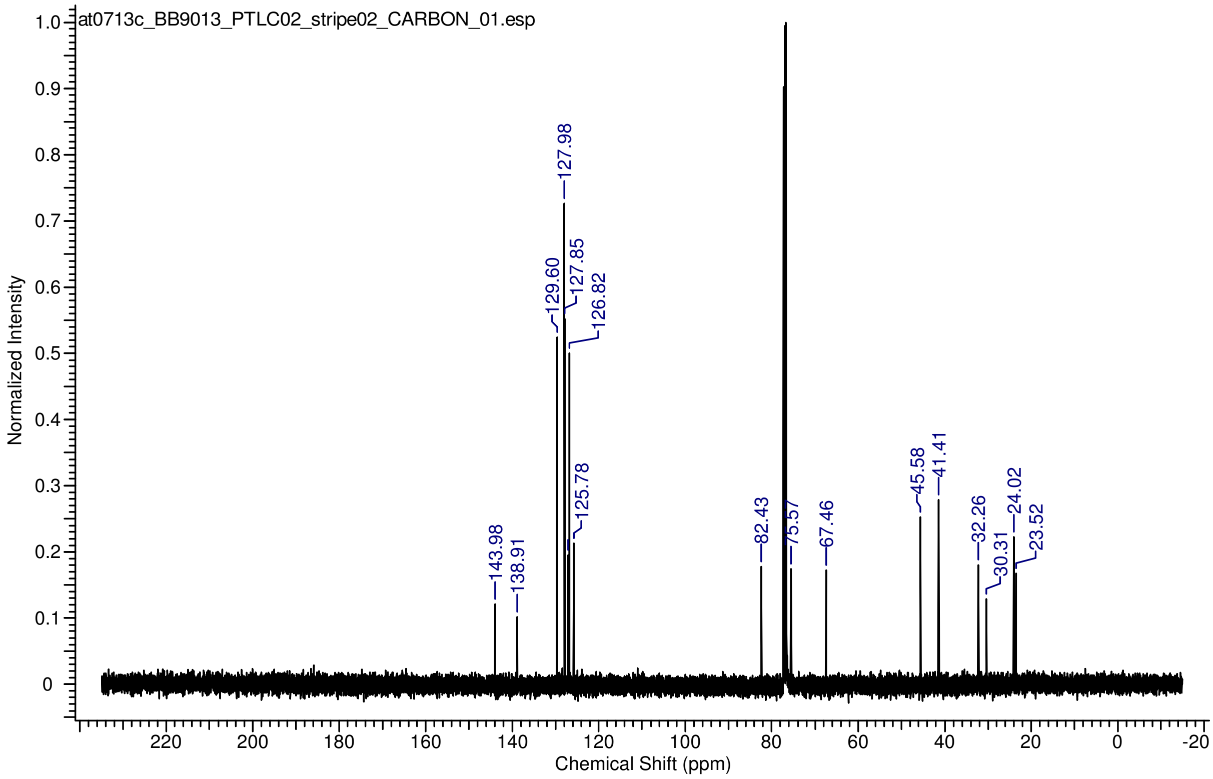

Chromatogram Plots

Plot 1: m:\... \at0713c_bb9013_p01001.sms RIC all
Plot 2: m:\... \at0713c_bb9013_p02002.sms RIC all

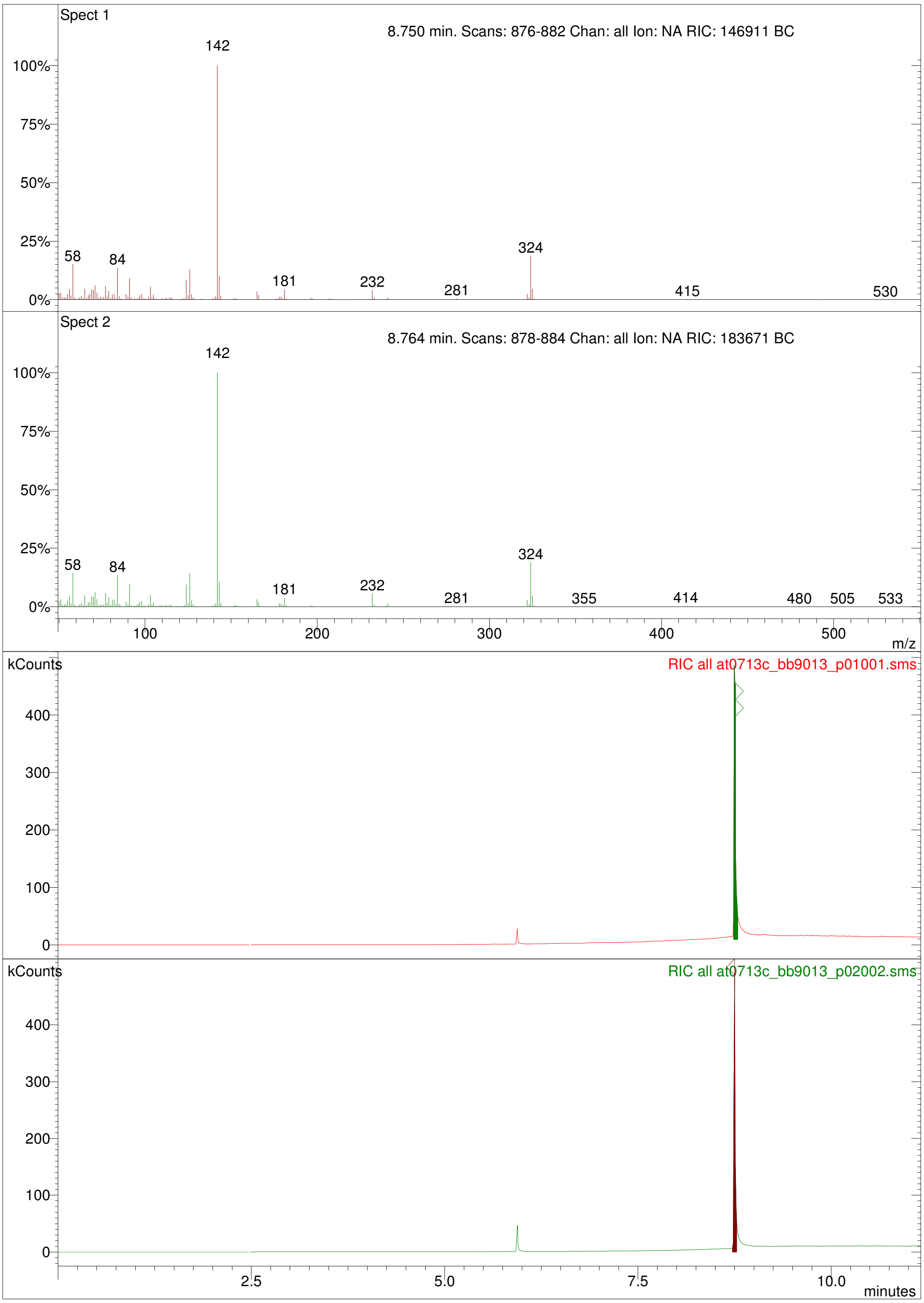

(S)-2-(((S)-1,2-Bis(4-methoxyphenyl)ethoxy)methyl)-1,1-dimethylpyrrolidin-1-ium, product 10

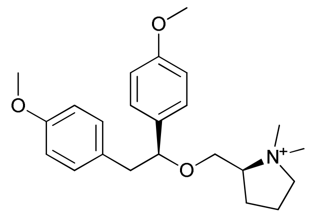

To a 4 mL glas vial containing (2*S*)-2-((1,2-bis(4-methoxyphenyl)ethoxy)methyl)-1-methylpyrrolidine (**5a**, 26.8 mg, 75 µmol) was added methyl iodide (0.5 mL). The mixture was stirred for 3 h at 40°C. When LC-MS showed total consumption of the starting material, methyl iodide was evaporated leaving a yellow solution. The product was crystallized by vapor crystallization: hot isobutanol was used as the main solvent (approx. 0.6 mL) and some droplets hot EtOAc were added. The vial was secured in a glass container partially filled with diethyl ether. The container was capped and left standing over night. The next day, crystals were identified and the container was transferred to the refrigerator were it was stored for 2 additional days. When a satisfactory number of crystals were formed, the solvent was filtered and the crystals washed with diethyl ether and dried under vacuum to obtain **11** in 50% yield (14 mg pure product).

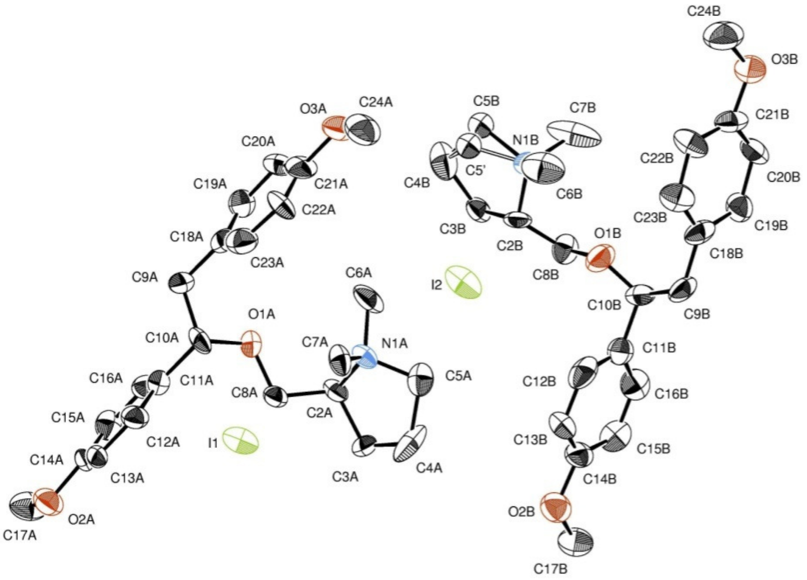

Explanation. For the compound there are two molecules (two cations and anions) in the independent part of the unit cell. The structure disorder occurs for one of the molecules. The occupation factors of the disordered atoms (C5B and C5’) are equal 0.5.

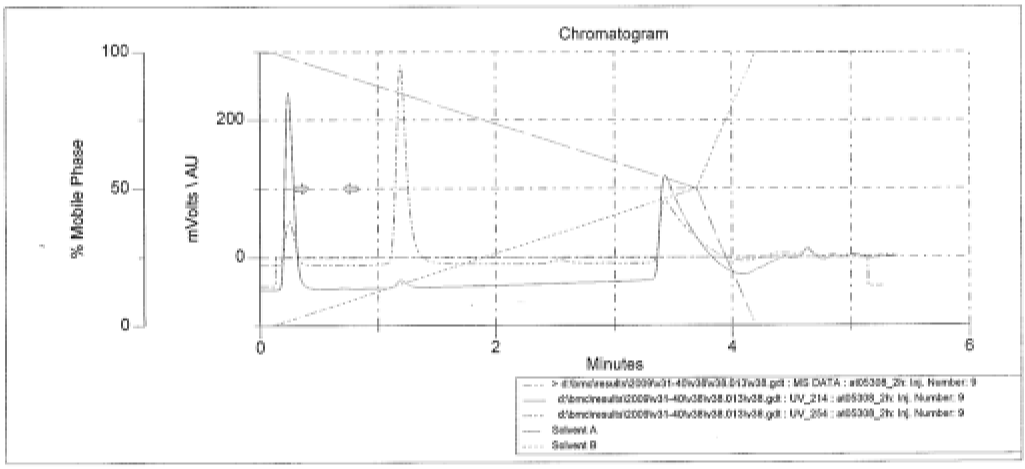

Chromatogram
% Mobile Phase
mVolts \ AU
Minutes

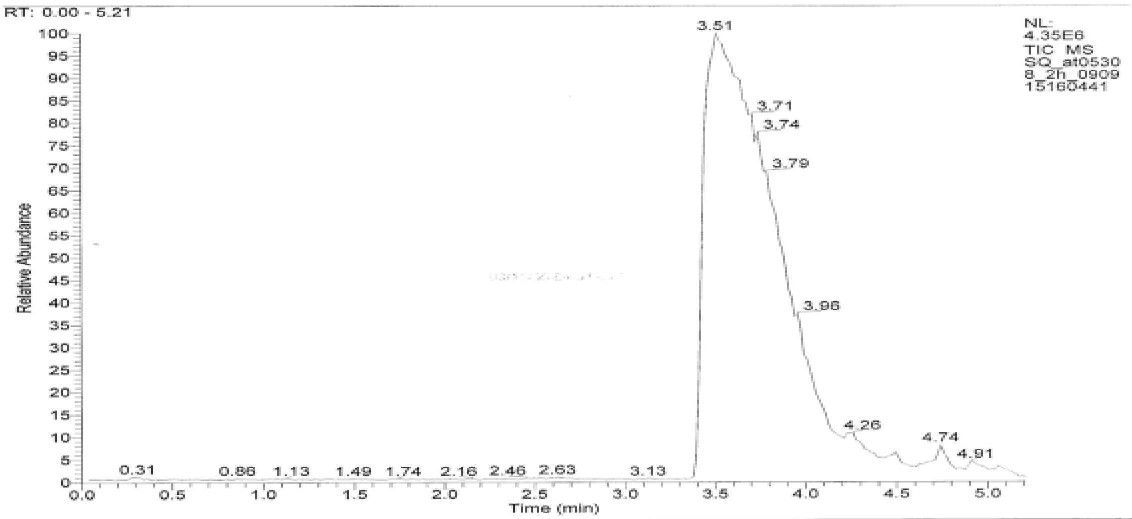

RT: 0.00 - 5.21
NL:
4.35E6
TIC MS
SQ_at0530
8_2h_0909
15160441
Relative Abundance
Time (min)
3.51
3.71
3.74
3.79
3.96
4.26
4.74
4.91
0.31
0.86
1.13
1.49
1.74
2.16
2.46
2.63
3.13

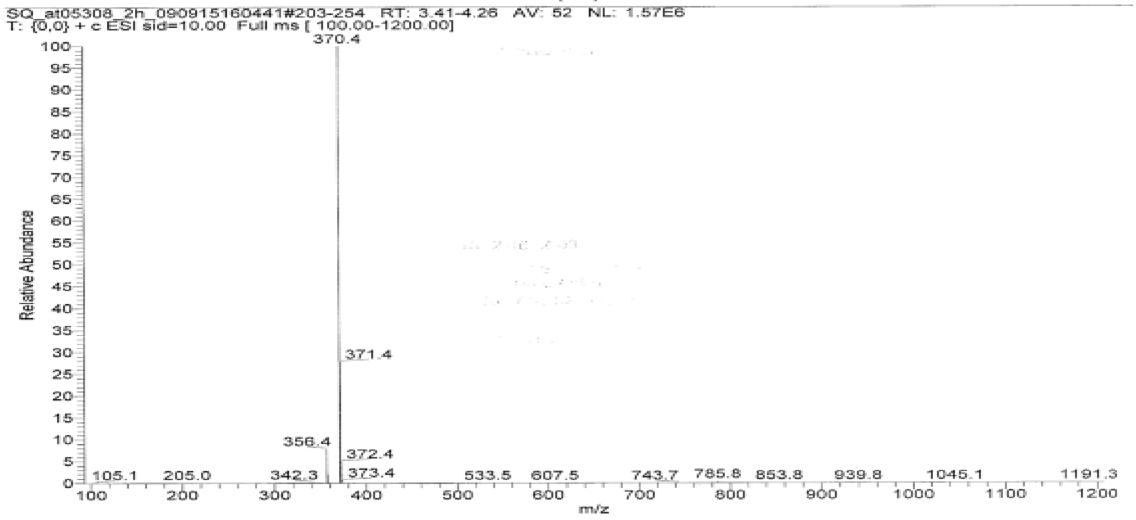

SQ_at05308_2h_090915160441#203-254 RT: 3.41-4.26 AV: 52 NL: 1.57E6
T: {0,0} + c ESI sid=10.00 Full ms [100.00-1200.00]
Relative Abundance
m/z
370.4
371.4
356.4
372.4
373.4
105.1
205.0
342.3
533.5
607.5
743.7
785.8
853.8
939.8
1045.1
1191.3

Experiments performed with addition of equimolar amounts of 4e and 4j

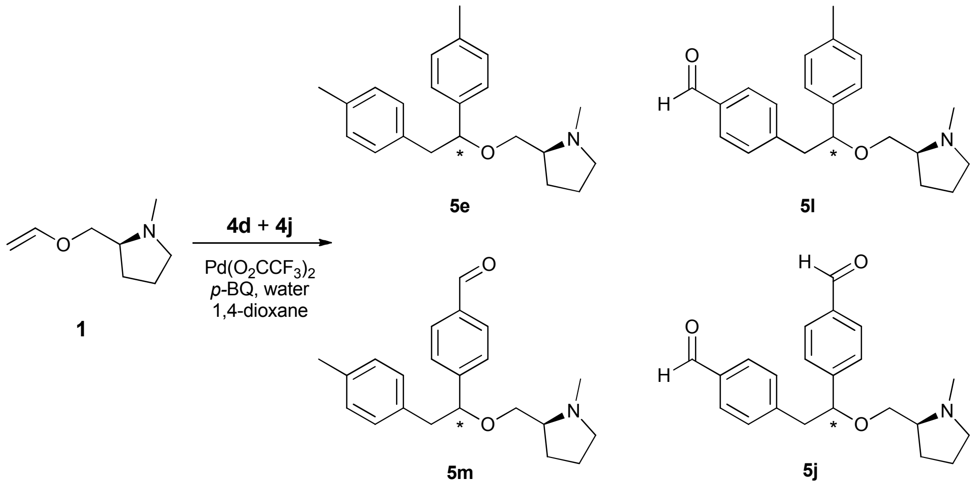

As described in the general procedure for synthesis of diarylated products **5**, **6** and **7(rac)**, but using 4-tolylboronic acid **4e** and 4-formylboronic acid **4j** in equimolar amounts (4 equiv each) as the arylating agents. The reaction was stirred for 24 hours and the crude was filtrated through a plug of silica. The acquired oily mixture was submitted to GC-MS and NMR-analysis were the ratio **5e**:**5l**:**5m**:**5j** was determined to 2:2:2:1 in 68% NMR yield (using DMF as intern standard) showing that the reaction is slightly sensitive for electronic effects.

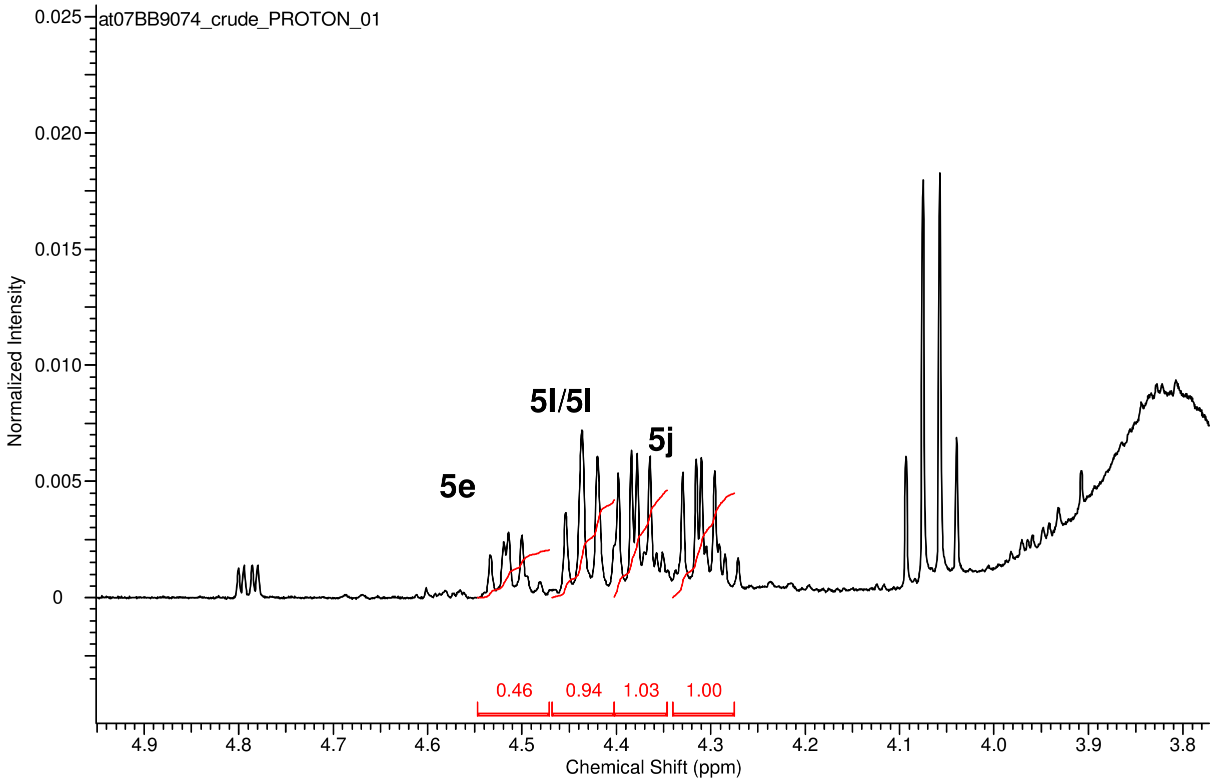

Chromatogram Plot

File: m:\... \referee comments\kompletterande exp\gcn\at07bb9075_crude.sms
Sample: at07BB9075_crude Operator: Operator
Scan Range: 1 - 2777 Time Range: 0.00 - 25.99 min. Date: 2012-01-11 15:47
Sample Notes: ROUTINE

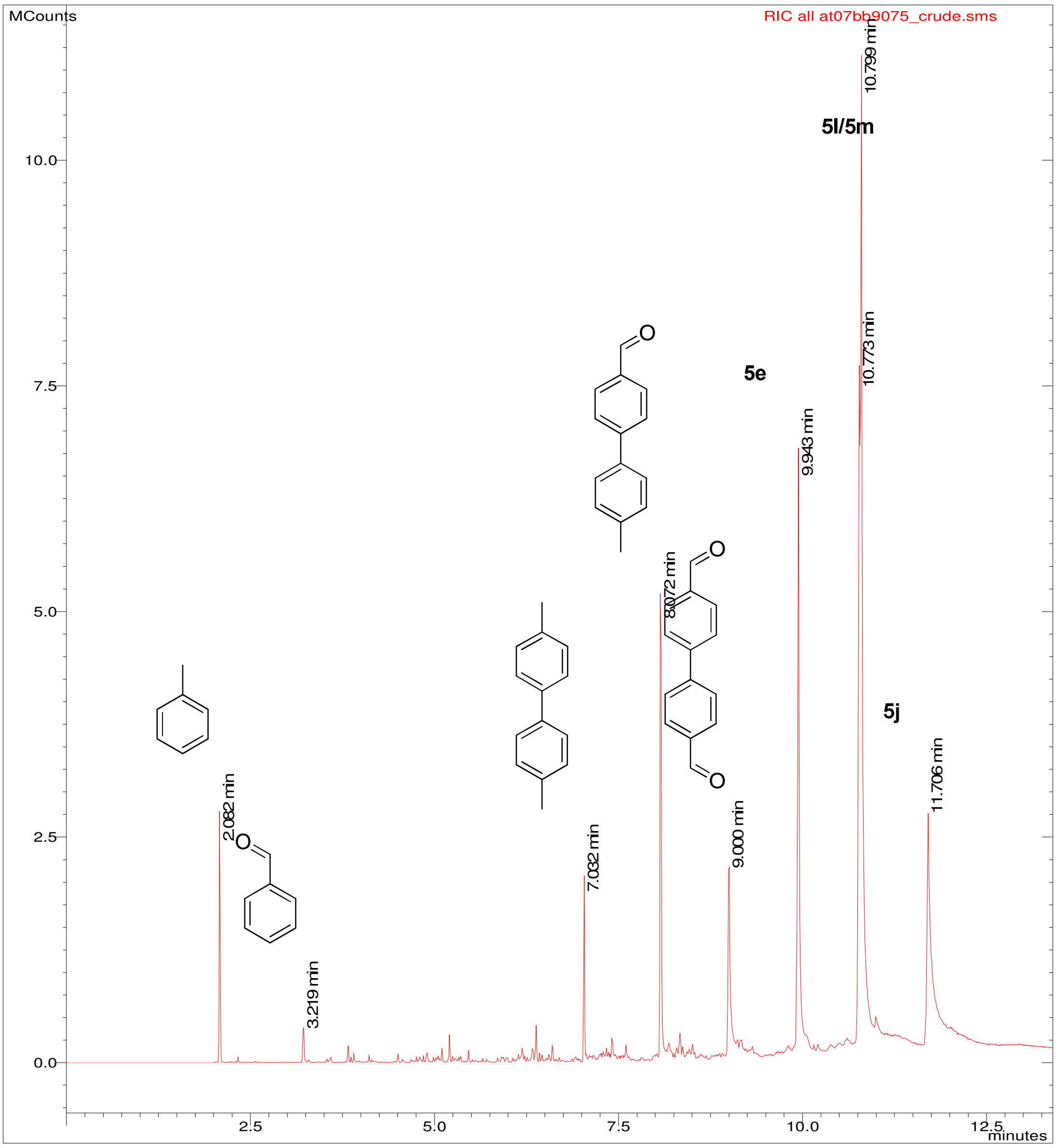

Experiments performed with addition of the chiral ligand (R)-BINAP

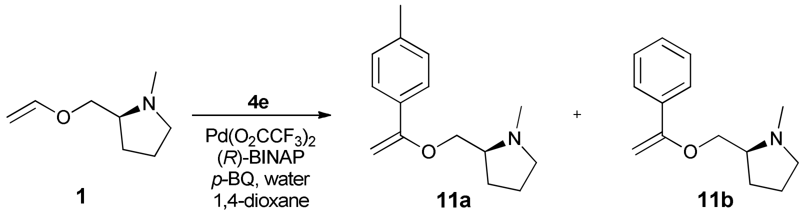

As described in the general procedure for synthesis of diarylated products **5**, **6** and **7(rac)**, but using 4-tolylboronic acid (**4e**) and adding (*R*)-BINAP (0.075 equiv) prior to $Pd(O_2CCF_3)_2$ (0.05 equiv) under nitrogen. The reaction was stirred for 24 hours and the crude was filtrated through a plug of silica. The acquired oily mixture was submitted to GC-MS and NMR-analysis were P-aryl/aryl exchange was detected.[4] The ratio **11a**:**11b** was determined to 3.2:1 and the total yield (**11a** + **11b** = **11**) was determined to 48% by crude NMR-analysis. Formation of diarylated product **5e** was also detected in very small amounts (<10%) and the ratio **11**:**5e** was determined to 8:1.

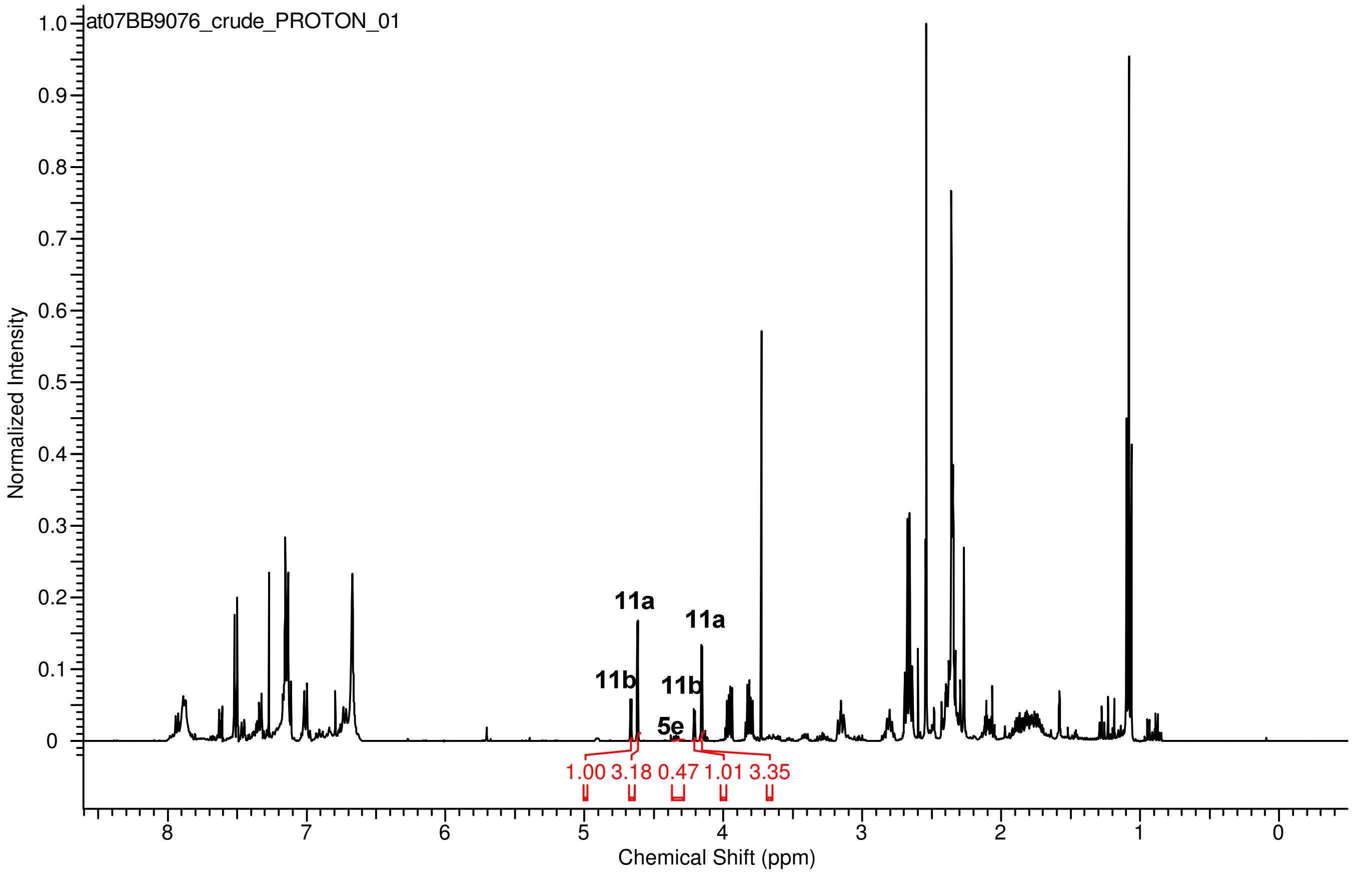

Chromatogram Plot

File: m:\... \referee comments\kompletterande exp\gcn\at07_bb9076_crude.sms
Sample: at07_BB9076_crude Operator: Operator
Scan Range: 1 - 1426 Time Range: 0.00 - 13.49 min. Date: 2012-01-10 10:05
Sample Notes: ROUTINE

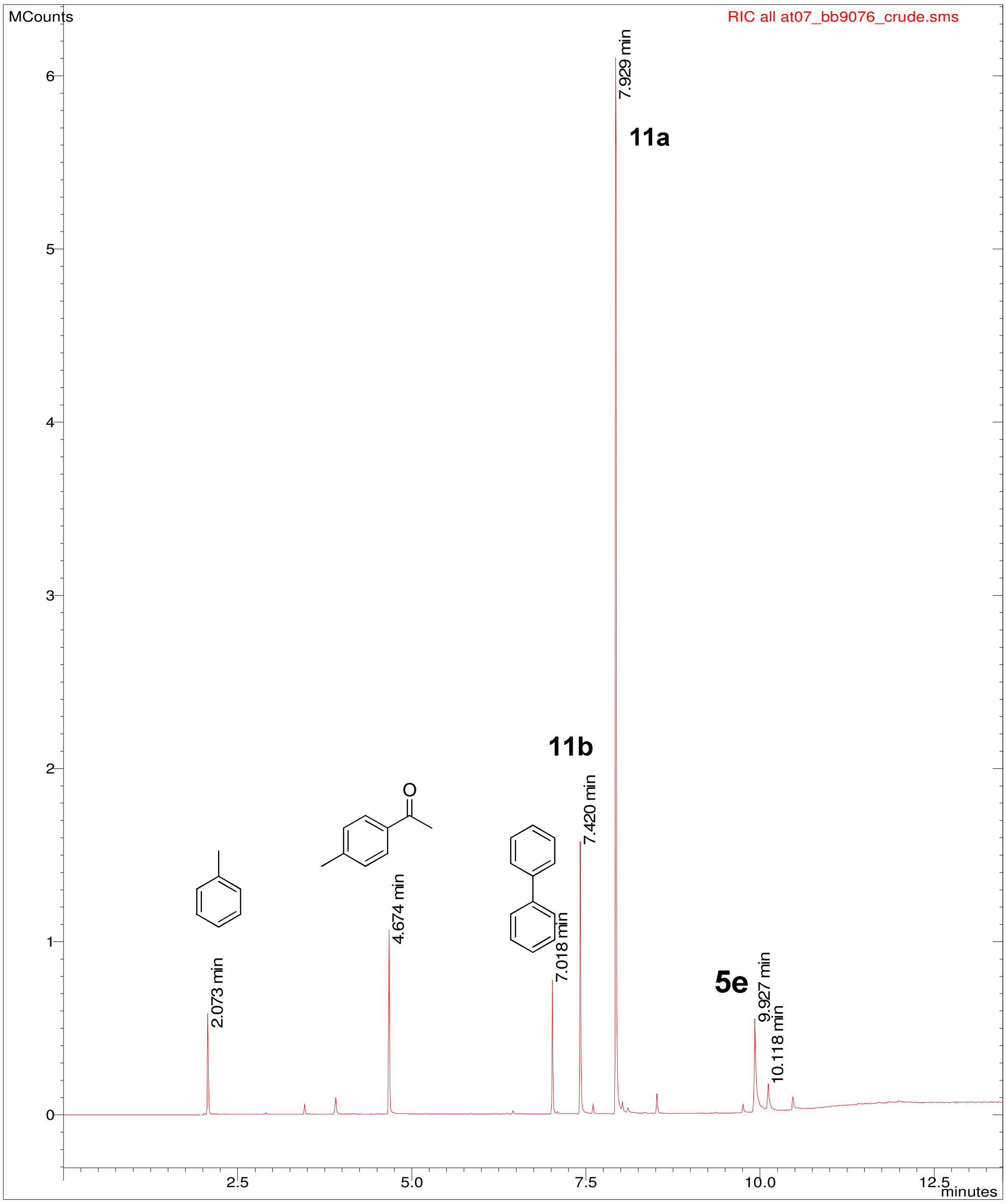

References

[1] D. S. Pedersen, C. Rosenbohm, *Synthesis* **2001**, *2001*, 2431.

[2] M. Bosch, M. Schlaf, *J. Org. Chem.* **2003**, *68*, 5225.

[3] a) J. Cossy, C. Dumas, D. G. Pardo, *Eur. J. Org. Chem.* **1999**, *1999*, 1693; b) T.-X. Métro, D. G. Pardo, J. Cossy, *J. Org. Chem.* **2007**, *72*, 6556.

[4] a) F. E. Goodson, T. I. Wallow, B. M. Novak, *J. Am. Chem. Soc.* **1997**, *119*, 12441; b) S. A. Macgregor, *Chem. Soc. Rev.* **2007**, *36*, 67.
